# Planar bismuth triamides: a tunable platform for main group Lewis acidity and polymerization catalysis†

**DOI:** 10.1039/d3sc00917c

**Published:** 2023-04-06

**Authors:** Tyler J. Hannah, W. Michael McCarvell, Tamina Kirsch, Joseph Bedard, Toren Hynes, Jacqueline Mayho, Karlee L. Bamford, Cyler W. Vos, Christopher M. Kozak, Tanner George, Jason D. Masuda, S. S. Chitnis

**Affiliations:** a Chemistry Department, Dalhousie University 6274 Coburg Rd Halifax NS B3H 4R2 Canada saurabh.chitnis@dal.ca; b Department of Chemistry, Memorial University of Newfoundland St. John's NL A1B 3X7 Canada; c Department of Chemistry, Saint Mary's University 923 Robie St. Halifax NS B3H 3C3 Canada

## Abstract

Geometric deformation in main group compounds can be used to elicit unique properties including strong Lewis acidity. Here we report on a family of *planar* bismuth(iii) complexes (*cf.* typically pyramidal structure for such compounds), which show a geometric Lewis acidity that can be further tuned by varying the steric and electronic features of the triamide ligand employed. The structural dynamism of the planar bismuth complexes was probed in both the solid and solution phase, revealing at least three distinct modes of intermolecular association. A modified Gutmann–Beckett method was used to assess their electrophilicity by employing trimethylphosphine sulfide in addition to triethylphosphine oxide as probes, providing insights into the preference for binding hard or soft substrates. Experimental binding studies were complemented by a computational assessment of the affinities and dissection of the latter into their intrinsic bond strength and deformation energy components. The results show comparable Lewis acidity to triarylboranes, with the added ability to bind two bases simultaneously, and reduced discrimination against soft substrates. We also study the catalytic efficacy of these complexes in the ring opening polymerization of cyclic esters ε-caprolactone and *rac*-lactide. The polymers obtained show excellent dispersity values and high molecular weights with low catalyst loadings used. The complexes retain their performance under industrially relevant conditions, suggesting they may be useful as less toxic alternatives to tin catalysts in the production of medical grade materials. Collectively, these results establish planar bismuth complexes as not only a novel neutral platform for main group Lewis acidity, but also a potentially valuable one for catalysis.

## Abstract 2

Planar bismuth compounds exhibit tunable Lewis acidity and high catalytic activity for lactone polymerization.

## Graphical abstract

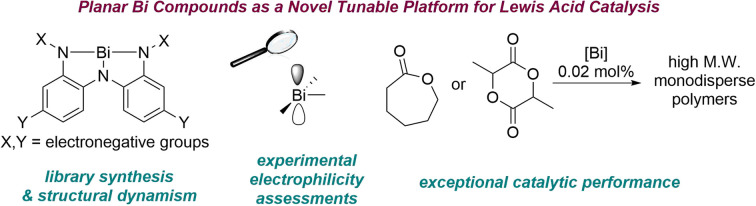

## 1 Introduction

The reactivity of main group elements can be tuned by variation of their steric or electronic environment – bulky substituents can stabilize low-valent compounds against oligomerization, allowing an examination of their unimolecular chemistry,^1–5^ while very electronegative substituents can engender high Lewis acidity that can be harnessed for catalytic reactions.^6–9^ Alongside such steric and electronic tuning, geometric deformation has emerged in recent years as a strategy to rationally control the behaviour of main group centres.^10,11^ Perturbing the geometry at a p-block element away from that predicted by valence shell electron pair repulsion (VSEPR) theory changes the frontier molecular orbital manifold with consequences for the molecule's spectroscopic profile and reactivity. Recent examples include the synthesis of pre-pyramidalized boranes,^12–14^ planar group 14 compounds,^15–18^ and unusual phosphorus, arsenic, and antimony compounds^19–22^ that deviate from their respective classical (VSEPR-predicted) geometries. These distorted compounds can exhibit high Lewis acidity even without an accompanying molecular charge or electron-withdrawing substituents. In some cases, they also show unprecedented small molecule activation chemistry and catalysis, making them important targets for synthetic chemistry.^16,23–30^

**Fig. 1:**
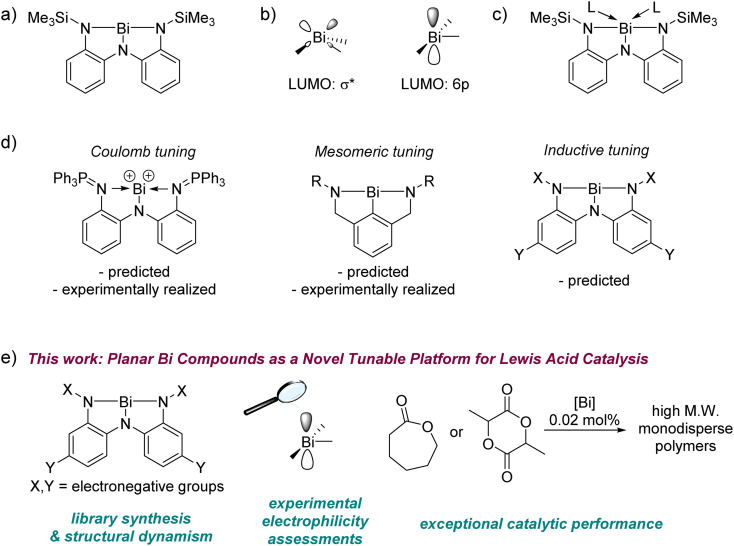
(a) The first planar bismuth triamide. (b) LUMOs of a generic pyramidal (left) and planar (right) Bi(iii) compound. (c) Ligand coordination to planar bismuth triamides. (d) Coulomb, mesomeric, and inductive tuning of planar bismuth complexes. (e) Establishing planar bismuth compounds as a novel inductively tunable platform for Lewis acidity and polymerization catalysis.

In this context, we have systematically probed the chemistry of *planar* bismuth(iii) triamides, whose T-shaped structures represent a gross departure from the VSEPR-predicted *pyramidal* arrangement of substituents around the metal (Fig. 1a).^31,32^ This deformation was enabled by a pincer triamide ligand with suitable steric protection to preclude attachment of multiple ligand equivalents to the metal.^33^ Planar bismuth triamides feature a vacant Bi 6p orbital as the lowest unoccupied molecular orbital (LUMO), whose orthogonal disposition to the molecular plane (Fig. 1b)^31,34^ is reminiscent of the electronic structure at planar trivalent boranes. Indeed, experimental studies have confirmed that it is possible to coordinate ligands to the metal centre in planar bismuth compounds, identifying them as a new platform for main group Lewis acid chemistry (Fig. 1c).^31,32^

Three approaches for tuning the properties of this platform may be envisioned (Fig. 1d). In a *Coulombic* approach, cationic charge was used to boost electrophilicity by using a bis-phosphinimino-amide ligand instead of a triamide ligand.^35^ Analogous bismuth cations and polycations have been reported to be strong Lewis acids with applications in catalysis.^36–42,95–98^ In a *mesomeric* tuning approach, one of the adjacent π-donor groups was removed to boost the metal-centred electrophilicity by precluding one of the three N(lp) → Bi(6p) interactions.^43^ A third, *inductive* approach, has been computationally predicted for tuning the electrophilicity in these complexes over a very wide range,^34,44^ but this approach remains experimentally unrealized, despite being the most convenient due to its modular nature. Conceptually, this inductive tuning method is analogous to that used fruitfully in the chemistry of triarylboranes, where the extent of aryl ring halogenation controls the Lewis acidity at boron.^45,46^

Here for the first time we experimentally demonstrate inductive tuning of electrophilicity at planar bismuth compounds by systematic alteration of a triamide ligand manifold. Our study covers the structural and spectroscopic consequences of the applied electronic perturbation, its effect upon metal-centred electrophilicity, and the discovery of exceptional catalytic performance in the polymerization of cyclic esters (Fig. 1e). The latter constitutes the first example of polymerization catalysis with geometrically deformed pnictogen compounds. Collectively, these results identify planar bismuth triamides as a novel and inductively-tunable, neutral platform for main group Lewis acid catalysis.

## 2 Experimental

### 2.1 General considerations

All manipulations were performed using standard Schlenk and glovebox techniques under an atmosphere of dry nitrogen. Solvents were distilled from Na/benzophenone (tetrahydrofuran, pentanes, hexanes, diethyl ether, toluene) or calcium hydride (dichloromethane, acetonitrile, 1,2-dichlorobenzene) and stored over sieves prior to use. Deuterated benzene was freeze–pump–thawed twice and stored over activated 3 Å sieves for at least 48 h. Reaction glassware was baked in a 150 °C oven for at least 1 h prior to use and assembled under nitrogen or pumped into a glovebox while hot.

Nuclear magnetic resonance spectra are referenced to tetramethylsilane (^1^H, ^13^C) on a Bruker AV-300 spectrometer or a Bruker AV-500 spectrometer with residual solvent used for chemical shift calibration. Unless otherwise specified, all spectra were recorded at 300 K. Samples for NMR spectroscopy were prepared and sealed inside the glovebox with Parafilm before removal into ambient atmosphere. Infrared spectra were obtained on a Bruker Tensor 27 instrument between KBr plates with the sample dropcast as a thin film. UV-vis spectra were obtained on a cordless SpectroVis Plus spectrometer using a glass cuvette. All sample preparation and analysis were performed in the glovebox. Background correction was performed using a cuvette containing the analysis solvent. Melting points were obtained for samples by sealing glass capillaries with grease and parafilm. The melting point values are uncorrected. Single crystal diffraction experiments were performed on a Bruker APEX-II CCD diffractometer or D8 Venture diffractometer. Reflections were integrated using the APEX 3 or 4 software^47^ and solved using SHELXT^48^ and refined using SHELXL^49^ with the Olex2 software GUI.^50^ Electro-spray ionization (ESI) and atmospheric pressure chemical ionization (APCI) mass spectra were obtained on a Bruker micrOTOF instrument. Elemental analyses were preformed using samples packaged in tin boats inside a glovebox. Combustion analysis was performed using an Elementar Unicube instrument in CHN/S mode. Note that the journal requirements of ±0.40% accuracy for all elements have recently been critically re-evaluated.^51^ Gel permeation chromatography (GPC) to determine polymer molecular weights and dispersity values were performed by triple detection using an Agilent Technologies 1260 Infinity HPLC equipped with Phenogel 10^3^ Å, 300 × 4.60 mm and 10^4^ Å, 300 × 4.60 mm columns (covering mass ranges of 1000–75 000 and 5000–500 000 g mol^−1^, respectively). THF was used as the eluent at a flow rate of 0.30 mL min^−1^ running at 25 °C. The HPLC was coupled to Wyatt Technologies multiangle light scattering, viscometry, and refractive index detectors and processed using the Astra 6 software package. Chromatograms are provided in the ESI.† MALDI-TOF mass spectrometry of polymers was performed using a Bruker ultrafleXtreme MALDI-TOF/TOF analyzer with a Bruker smartbeam-II laser (up to 2 kHz, operating at 355 nm) in reflectron mode. Mass spectra of 1000 shots were accumulated. For polymer analysis 2,5-dihydroxybenzoic acid (DHBA) was used as a matrix and was dissolved in THF at a concentration of 15 mg mL^−1^. The polymer samples were dissolved in THF at concentrations of 10 mg mL^−1^ and sodium trifluoroacetate (NaTFA) cationizing agent was dissolved in THF at a concentration of 0.1 mol L^−1^. Solutions of matrix, polymer sample, and sodium salt were mixed in a volume ratio of 20 : 3 : 1, respectively. The resulting solution was hand-spotted on a stainless steel MALDI target plate using aliquots of 0.5 μL and the solvent was allowed to evaporate. MALDI-TOF MS data were processed, and images were prepared using MestReNova software with the mass analysis plug-in. Representative spectra are provided in the ESI.†

Bismuth(iii) chloride was purchased from Oakwood Chemicals and purified by vacuum sublimation (10^−2^ mbar, 200 °C) prior to use. Chlorotrimethylsilane (TMSCl) and chlorotriisopropylsilane (TIPSCl) were purchased from TCI America and used as received. Silver trifluoromethanesulfonate, and 1,3,5-tri-isopropylphenyl sulfonyl chloride was obtained from Oakwood and used as received. ε-Caprolactone and *rac*-lactide were obtained from Millipore Sigma and purified as per the procedures described below prior to usage. All other reagents were purchased from Millipore Sigma and used as received. Additional synthetic details are included in the ESI.†

### 2.2 Selected procedures & characterization data

#### 2.2.1 Compound 1b

Bis(2-amino-4-bromophenyl)amine (5.00 g, 14.0 mmol) and triethylamine (3.5 g, 35 mmol) were dissolved in THF (120 mL). Trimethylchlorosilane (3.20 g, 29.5 mmol) was added to this slowly and the mixture was stirred for 3 days at room temperature. This solution was evacuated to dryness and redissolved in pentane (100 mL), filtered, and concentrated then cooled to −30 °C for 2 days resulting in pale yellow crystals of 1b. Yield: 80%, 5.65 g; melting point 78–81 °C; elemental analysis: found: C, 43.43; H, 5.48; N, 8.47. Calc. for C_18_H_27_Br_2_N_3_Si_2_: C, 43.12; H, 5.43; N, 8.38; ^1^H NMR: *δ*_H_ (500 MHz, C_6_D_6_) 7.16 (2H, s, Ar-H), 6.82 (2H, d, *J* 8.3, Ar-H), 6.27 (2H, d, *J* 8.3, Ar-H), 4.07 (1H, s, N-H), 3.38 (2H, s, N-H), 0.03 (18H, s, Si(CH_3_)_3_); ^13^C NMR: *δ*_C_ (126 MHz, C_6_D_6_) 141.92 (C_Ar_), 132.02 (C_Ar_), 123.12 (C_Ar_), 121.93 (C_Ar_), 120.06 (C_Ar_), 117.20 (C_Ar_), −0.20 (Si(CH_3_)_3_).

#### 2.2.2 Compound 1c

Bis(2-amino-4-bromophenyl)amine (357 mg, 1.0 mmol) and triethylamine (253 mg, 2.5 mmol) were dissolved in THF (15 mL). Triisopropylsilyl trifluoromethanesulfonate (644 mg, 2.1 mmol) was added to this slowly and the mixture was stirred for 3 days at room temperature. Solution was evacuated to dryness and redissolved in pentane (25 mL), then filtered and evacuated to dryness collecting 1c as a pale brown solid. This product was of suitable purity for use further but could be purified by recrystallization from pentane. Yield: 72%, 480 mg; elemental analysis: found: C, 53.63; H, 7.77; N, 6.18. Calc. for C_30_H_51_Br_2_N_3_Si_2_: C, 53.80; H, 7.68; N, 6.27; ^1^H NMR: *δ*_H_ (300 MHz, C_6_D_6_) 7.24 (2H, d, *J* 2.2, Ar-H), 6.80 (2H, dd, *J* 8.3, 2.2, Ar-H), 6.22 (2H, d, *J* 8.2, Ar-H), 4.23 (1H, s, N-H), 3.60 (2H, s, NH_2_), 1.12–1.08 (6H, m, *CH*(CH_3_)_2_), 0.98 (36H, d, *J* 6.7, CH(*CH*_3_)_2_); ^13^C NMR: *δ*_C_ (75 MHz, C_6_D_6_) 142.61 (C_Ar_), 130.67 (C_Ar_), 123.07 (C_Ar_), 121.37 (C_Ar_), 119.49 (C_Ar_), 117.64 (C_Ar_), 18.53 (*CH*(CH_3_)_2_), 12.63 (CH(*CH*_3_)_2_); APCI-HRMS (positive ion mode): calculated for [C_30_H_51_Br_2_N_3_Si_2_]^+^ = 667.1983 *m*/*z*, observed = 667.1986 *m*/*z*.

#### 2.2.3 Compound 1e

Silver trifluoromethanesulfonate (2.157 g, 8.4 mmol) and 2,4,6-triisopropylbenzenesulfonyl chloride (2.68 g, 8.8 mmol) were dissolved in a mixture of acetonitrile (25 mL) and diethyl ether (50 mL) and stirred in the dark for 2 hours. Separately, L2 (1.5 g, 4.2 mmol) and pyridine (0.994 g, 12.6 mmol) were dissolved in acetonitrile (50 mL). The sulfonyl chloride solution was added slowly to the amine resulting in a colour change initially to green followed by purple, and this mixture was refluxed for 3 days. This was cooled to room temperature and evacuated to dryness giving a purple foam. This was redissolved in DCM (100 mL) and washed with HCl (1 M, 6 × 100 mL) followed by water (100 mL) and brine (100 mL). The organic layer was dried over magnesium sulfate, filtered, and evacuated to dryness giving a purple solid. This was washed with pentane (6 × 20 mL) giving 1e as a pale pink solid. Yield: 57%, 2.13 g; melting point 94–103 °C; elemental analysis: found: C, 57.20; H, 6.31; N, 4.51. Calc. for C_42_H_55_Br_2_N_3_O_4_S_2_: C, 56.69; H, 6.23; N, 4.72; ^1^H NMR: *δ*_H_ (500 MHz, CDCl_3_) 7.19 (4H, s, Ar-H), 7.12 (2H, dd, *J* 8.6, 2.2, Ar-H), 6.81 (2H, d, *J* 2.2, Ar-H), 6.72 (2H, s, N-H), 6.64 (2H, d, *J* 8.6, Ar-H), 4.04 (4H, p, *J* 6.7, *CH*(CH_3_)_2_), 2.92 (2H, p, *J* 6.9, *CH*(CH_3_)_2_), 1.27 (12H, d, *J* 6.9, CH(*CH*_3_)_2_), 1.22 (24H, d, *J* 6.7, CH(*CH*_3_)_2_); ^13^C NMR: *δ*_C_ (126 MHz, CDCl_3_) 153.96 (C_Ar_), 150.88 (C_Ar_), 136.32 (C_Ar_), 131.80 (C_Ar_), 129.97 (C_Ar_), 129.59 (C_Ar_), 126.71 (C_Ar_), 124.31 (C_Ar_), 122.16 (C_Ar_), 115.37 (C_Ar_), 34.38 (*CH*(CH_3_)_2_), 30.13 (*CH*(CH_3_)_2_), 24.88 (CH(*CH*_3_)_2_), 23.69 (CH(*CH*_3_)_2_); ESI-HRMS (negative ion mode): calculated for [C_42_H_54_Br_2_N_3_O_4_S_2_]^−^ = 886.1899 *m*/*z*, observed = 886.1928 *m*/*z*.

#### 2.2.4 Compound 2d

Bi(NMe_2_)_3_ (72 mg, 0.21 mmol) and 1d (154 mg, 0.21 mmol) were weighed out and dissolved in toluene (10 mL) separately, then cooled to −30 °C. The Bi solution was added slowly to the ligand while stirring. During this time a colour change to orange was observed. Solution was filtered and evaporated to dryness giving an orange solid which was recrystallized from pentane at −30 °C. Yield: 67%, 133 mg; elemental analysis (including 1 molecule of pentane): found: C, 55.31; H, 6.49; N, 6.40; S, 5.72. Calc. for C_51_H_80_BiN_5_O_4_S_2_: 55.67; H, 7.33; N, 6.36; S, 5.83.; ^1^H NMR: *δ*_H_ (500 MHz, C_6_D_6_) 7.56 (2H, dd, *J* 8.2, 1.4, Ar-H), 7.32 (4H, s, Ar-H), 7.12 (2H, dd, *J* 8.0, 1.5, Ar-H), 6.74 (2H, ddd, *J* 8.5, 7.3, 1.5, Ar-H), 6.51 (2H, ddd, *J* 8.4, 7.3, 1.3, Ar-H), 5.01 (4H, hept, *J* 6.8, *CH*(CH_3_)_2_), 3.55 (2H, s, *HN*(CH_3_)_2_), 2.72 (2H, hept, *J* 6.9, *CH*(CH_3_)_2_), 2.33 (12H, s, HN(*CH*_3_)_2_), 1.37 (24H, d, *J* 6.8, CH(*CH*_3_)_2_), 1.16 (12H, d, *J* 6.9, CH(*CH*_3_)_2_).; ^13^C NMR: *δ*_C_ (126 MHz, C_6_D_6_) 152.46 (C_Ar_), 150.26 (C_Ar_), 148.51 (C_Ar_), 139.11 (C_Ar_), 137.84 (C_Ar_), 121.20 (C_Ar_), 120.53 (C_Ar_), 120.29 (C_Ar_), 119.42 (C_Ar_), 37.31 (HN(CH_3_)_2_), 34.45 (*CH*(CH_3_)_2_), 29.93 (*CH*(CH_3_)_2_), 25.23 (CH(*CH*_3_)_2_), 23.77 (CH(*CH*_3_)_2_). Crystal data for 2d (plus solvent) C_49_H_75_BiN_5_O_4_S_2_: orthorhombic, space group *Pbcn* (no. 60), *a* = 33.0206(10) Å, *b* = 10.3986(3) Å, *c* = 29.7870(9) Å, *V* = 10 227.9(5) Å^3^, *Z* = 8, *T* = 150.00 K, *μ*(CuKα) = 7.882 mm^−1^, *D*_calc_ = 1.391 g cm^−3^, 317 789 reflections measured (5.352° ≤ 2*Θ* ≤ 149.334°), 10 464 unique (*R*_int_ = 0.0542, *R*_sigma_ = 0.0139) which were used in all calculations. The final *R*_1_ was 0.0319 (I > 2*σ*(*I*)) and w*R*_2_ was 0.0755 (all data). CCDC no. 2217278.

#### 2.2.5 Compound 3b

Triamine 1b (1.01 g, 2.0 mmol) and Bi(NMe_2_)_3_ (0.68 g, 2.0 mmol) were dissolved in hexanes (50 mL) separately and cooled to −30 °C. The Bi(NMe_2_)_3_ solution was added to the ligand dropwise resulting in a colour change initially to red followed by blue. This solution was allowed to warm to room temperature and stirred overnight. Solution was filtered and concentrated to dryness giving analytically pure 3b as a blue solid. X-ray quality crystals were grown from a concentrated pentane solution at −30 °C. Yield: 95%, 1.35 g; melting point: 173–179 °C (decomposition); elemental analysis (including 1 molecule of THF): found: C, 34.31; H, 4.15; N, 5.46. Calc. for C_22_H_32_BiBr_2_N_3_OSi_2_: 33.90; H, 4.14; N, 5.39; ^1^H NMR (300 MHz, C_6_D_6_): *δ* 7.70–7.65 (4H, m, Ar-H), 6.82 (2H, dd, J 9.0, 2.2, Ar-H), 0.32 (18H, s, Si(CH_3_)_3_). ^13^C NMR: *δ*_C_ (75 MHz, C_6_D_6_) 154.04 (C_Ar_), 150.98 (C_Ar_), 125.62 (C_Ar_), 123.20 (C_Ar_), 120.03 (C_Ar_), 119.16 (C_Ar_), 1.63 Si(CH_3_)_3_. ESI-HRMS (positive ion mode): calculated for [C_18_H_25_BiBr_2_N_3_Si_2_]^+^ = 705.975 225 *m*/*z*, observed = 705.974 871 *m*/*z*. Crystal data for dimeric 3b (C_36_H_48_Bi_2_Br_4_N_6_Si_4_): triclinic, space group *P*1̄ (no. 2), *a* = 9.6987(8) Å, *b* = 11.0671(9) Å, *c* = 13.5493(12) Å, *α* = 109.747(3)°, *β* = 92.089(3)°, *γ* = 104.352(3)°, *V* = 1314.52(19) Å^3^, *Z* = 1, *T* = 150.00 K, *μ*(CuKα) = 17.648 mm^−1^, *D*_calc_ = 1.787 g cm^−3^, 24 255 reflections measured (6.992° ≤ 2*Θ* ≤ 140.118°), 4889 unique (*R*_int_ = 0.0683, *R*_sigma_ = 0.0462) which were used in all calculations. The final *R*_1_ was 0.0660 (*I* > 2*σ*(*I*)) and w*R*_2_ was 0.1840 (all data). CCDC no. 2217277.

#### 2.2.6 Compound 3c

Triamine 1c (665 mg, 1.0 mmol) and Bi(NMe_2_)_3_ (340 mg, 1.0 mmol) were dissolved in hexanes (25 mL) separately and cooled to −30 °C. The Bi(NMe_2_)_3_ solution was added to the ligand dropwise resulting in a colour change to blue. This solution was allowed to warm to room temperature and stirred overnight. Solution was filtered and concentrated to dryness giving analytically pure 3c as a blue solid. X-ray quality crystals were grown from a concentrated pentane solution at −30 °C. Yield: 94%, 840 mg; melting point 119–123 °C (decomposition); elemental analysis: found: C, 41.07; H, 5.52; N, 4.99. Calc. for C_30_H_48_BiBr_2_N_3_Si_2_: 41.15; H, 5.52; N, 4.80; ^1^H NMR: *δ*_H_ (300 MHz, C_6_D_6_) 7.84 (2H, d, J 2.2, Ar-H), 7.67 (2H, d, J 9.1, Ar-H), 6.72 (2H, dd, J 9.1, 2.2, Ar-H), 1.56 (6H, p, J 7.3, *CH*(CH_3_)_2_), 1.13 (36H, d, J 7.5, CH(*CH*_3_)_2_); ^13^C NMR: *δ*_C_ (75 MHz, C_6_D_6_) 155.23 (C_Ar_), 151.21 (C_Ar_), 126.99 (C_Ar_), 123.29 (C_Ar_), 120.13 (C_Ar_), 119.65 (C_Ar_), 19.00 (*CH*(CH_3_)_2_), 14.91 (CH(*CH*_3_)_2_); ESI-HRMS (positive ion mode): calculated for [C_30_H_49_BiBr_2_N_3_Si_2_]^+^ = 874.1630 *m*/*z*, observed = 874.1635 *m*/*z*. Crystal data for 3c (C_30_H_48_BiBr_2_N_3_Si_2_, suitable for connectivity only): monoclinic, space group *C*2/*c* (no. 15), *a* = 22.2542(5) Å, *b* = 13.9368(4) Å, *c* = 11.6414(3) Å, *β* = 108.4650(10)°, *V* = 3424.72(15) Å^3^, *Z* = 4, *T* = 150.00 K, *μ*(CuKα) = 13.676 mm^−1^, *D*_calc_ = 1.698 g cm^−3^, 19 519 reflections measured (7.6° ≤ 2*Θ* ≤ 140.124°), 3213 unique (*R*_int_ = 0.0733, *R*_sigma_ = 0.0513) which were used in all calculations. The final *R*_1_ was 0.1511 (*I* > 2*σ*(*I*)) and w*R*_2_ was 0.4076 (all data). CCDC no. 2217276.

#### 2.2.7 Compound 3d

Triamine 1d (184 mg, 0.25 mmol), and Bi(N(SiMe_3_)_2_)_3_ (173 mg, 0.25 mmol) were weighed out and dissolved in toluene (10 mL) separately. The Bi solution was added slowly to the ligand while stirring. Pyridine (10 mol%) was added to the reaction mixture, then the vessel was sealed and stirred at 60 °C for 4 days. During this time a colour change initially to red followed by purple was observed. Solution was evaporated to dryness and washed with pentane (4 × 3 mL) giving 3d as a violet solid in high purity. X-ray quality crystals were grown from a solution in benzene layered with pentane. Yield: 55%, 157 mg; melting point 161–167 °C (decomposition); elemental analysis (including 0.5 molecules of benzene): found: C, 55.15; H, 5.90; N, 4.05; S, 6.07. Calc. for C_45_H_57_BiN_3_O_4_S_2_: C, 55.32; H, 5.88; N, 4.30; S, 6.56; ^1^H NMR: *δ*_H_ (500 MHz, C_6_D_6_) 7.65 (2H, dd, *J* 8.5, 1.4, C_Ar_-H), 7.25 (2H, dd, *J* 8.7, 1.3, C_Ar_-H), 7.23 (4H, s, C_Ar_-H), 6.58 (2H, ddd, *J* 8.5, 7.1, 1.4, C_Ar_-H), 5.91 (2H, ddd, *J* 8.4, 7.0, 1.2, C_Ar_-H), 5.02 (4H, p, *J* 6.8, *CH*(CH_3_)_2_), 2.63 (2H, p, *J* 6.9, *CH*(CH_3_)_2_), 1.23 (24H, d, *J* 6.8, CH(*CH*_3_)_2_), 1.09 (12H, d, *J* 6.9, CH(*CH*_3_)_2_); ^13^C NMR: *δ*_C_ (126 MHz, C_6_D_6_) 153.37 (C_Ar_), 150.79 (C_Ar_), 148.76 (C_Ar_), 146.55 (C_Ar_), 136.75 (C_Ar_), 128.59 (C_Ar_), 124.50 (C_Ar_), 124.33 (C_Ar_), 118.86 (C_Ar_), 117.24 (C_Ar_), 34.41 (*CH*(CH_3_)_2_), 30.34 (*CH*(CH_3_)_2_), 24.82 (CH(*CH*_3_)_2_), 23.65 (CH(*CH*_3_)_2_); APCI-HRMS (positive ion mode): calculated for [C_42_H_55_BiN_3_O_4_S_2_]^+^ = 938.3432 *m*/*z*, observed = 938.3416 *m*/*z*. Crystal data for dimeric 3d·0.5C_6_H_6_ (C_45_H_57_BiN_3_O_4_S_2_): triclinic, space group *P*1̄ (no. 2), *a* = 11.0336(14) Å, *b* = 12.7111(18) Å, *c* = 17.108(2) Å, *α* = 81.082(6)°, *β* = 79.522(5)°, *γ* = 65.058(5)°, *V* = 2131.0(5) Å^3^, *Z* = 2, *T* = 150.00 K, *μ*(MoKα) = 4.280 mm^−1^, *D*_calc_ = 1.523 g cm^−3^, 56 400 reflections measured (3.548° ≤ 2*Θ* ≤ 53.462°), 9027 unique (*R*_int_ = 0.0660, *R*_sigma_ = 0.0410) which were used in all calculations. The final *R*_1_ was 0.0503 (*I* > 2*σ*(*I*)) and w*R*_2_ was 0.1335 (all data). CCDC no. 2217453.

#### 2.2.8 Compound 3e

Triamine 1e (445 mg, 0.5 mmol), and Bi(N(SiMe_3_)_2_)_3_ (346 mg, 0.5 mmol) were weighed out and dissolved in toluene (10 mL) separately. The Bi solution was added slowly to the ligand while stirring. Pyridine (10 mol%) was added to the reaction mixture, then the vessel was sealed and stirred at 80 °C for 4 days. During this time a colour change initially to red followed by purple was observed. Solution was evaporated to dryness and washed with pentane (4 × 5 mL) giving 3e as a violet solid. Yield: 45%, 247 mg; melting point 172–176 °C (decomposition); elemental analysis (including 1 molecule of benzene): found: C, 49.02; H, 5.28; N, 3.69; S, 4.93. Calc. for C_48_H_58_BiBr_2_N_3_O_4_S_2_: C, 49.11; H, 4.98; N, 3.58; S, 5.46; ^1^H NMR: *δ*_H_ (500 MHz, CD_3_CN) 7.33 (4H, s, C_Ar_-H), 7.21–7.10 (2H, m, C_Ar_-H), 6.87–6.81 (2H, m, C_Ar_-H), 6.70 (2H, s, C_Ar_-H), 4.61–4.55 (4H, m, *CH*(CH_3_)_2_), 3.03–2.94 (2H, m, *CH*(CH_3_)_2_), 1.28 (12H, d, *J* 7.0, (CH(*CH*_3_)_2_)), 1.23 (24H, s, (CH(*CH*_3_)_2_)).; ^13^C NMR: *δ*_C_ (126 MHz, CD_3_CN) 153.57 (C_Ar_), 151.22 (C_Ar_), 150.98 (C_Ar_), 137.61 (C_Ar_), 125.87 (C_Ar_), 125.07 (C_Ar_), 122.43 (C_Ar_), 120.79 (C_Ar_), 120.27 (C_Ar_), 118.77 (C_Ar_), 34.86 (*CH*(CH_3_)_2_), 30.40 (*CH*(CH_3_)_2_), 25.10 (CH(*CH*_3_)_2_), 23.88 (CH(*CH*_3_)_2_).; APCI-HRMS (positive ion mode): calculated for [C_42_H_53_BiBr_2_N_3_O_4_S_2_]^+^ = 1094.1643 *m*/*z*, observed = 1094.1598 *m*/*z*. Crystal data for dimeric 3e·(NC_6_H_5_)(C_6_H_6_) (Bi_2_Br_4_C_112_H_132_N_8_O_8_S_4_): triclinic, space group *P*1̄ (no. 2), *a* = 16.4085(16) Å, *b* = 18.9240(19) Å, *c* = 19.8569(19) Å, *α* = 83.424(4)°, *β* = 89.804(4)°, *γ* = 66.363(4)°, *V* = 5605.3(10) Å^3^, *Z* = 2, *T* = 150.00 K, *μ*(MoKα) = 4.693 mm^−1^, *D*_calc_ = 1.531 g cm^−3^, 292 086 reflections measured (3.486° ≤ 2*Θ* ≤ 53.464°), 23 818 unique (*R*_int_ = 0.0541, *R*_sigma_ = 0.0264) which were used in all calculations. The final *R*_1_ was 0.0528 (*I* > 2*σ*(*I*)) and w*R*_2_ was 0.1674 (all data). CCDC no. 2217454.

#### 2.2.9 Preparation of Gutmann–Beckett experiments

The Lewis acid (0.025 mmol, 5 eq.) and the corresponding phosphine oxide or sulfide (0.005 mmol, 1 eq.) were dissolved in 0.6 mL of benzene and immediately analyzed *via* NMR spectroscopy.

#### 2.2.10 Purification of ε-caprolactone

To remove residual water, technical grade ε-caprolactone was stirred with CaH_2_ under dry nitrogen at 50 °C for 48 h and then vacuum distilled into a Teflon-valved glass ampoule. The monomer was subsequently dispensed for polymerization studies inside a glovebox.

#### 2.2.11 Polymerization of ε-caprolactone

The bismuth catalyst (1 equivalent) was added to ε-caprolactone (100–5000 equivalents) in 0.3 mL of a given solvent. The reaction mixture was heated in an NMR tube, and progress was monitored by ^1^H NMR. Once complete, the polymer was isolated by dissolving the formed gel in DCM or THF (2–3 mL), precipitating the resulting solution into rapidly stirring methanol (20 mL) at 0 °C, and centrifuging this suspension to obtain a solid product. This precipitation procedure was repeated up to three times and the product thus obtained was dried under vacuum. Isolated yields were typically between 50–70%.

#### 2.2.12 Purification of *rac*-lactide

To remove residual water, *rac*-lactide was exposed to dynamic vacuum (1 × 10^−3^ mbar) in a Schlenk flask at ambient temperature for 48 h. The monomer was subsequently dispensed for polymerization studies inside a glovebox.

#### 2.2.13 Polymerization of *rac*-lactide

*rac*-Lactide (100–5000 equivalents) and the bismuth catalyst (1 equivalent) were transferred into an NMR tube and dissolved/suspended in the desired solvent (*ca.* 0.5 mL). The reaction mixture was heated in an NMR tube, and progress was monitored by ^1^H NMR. Once complete, the polymer was isolated by dissolving the formed gel in DCM or THF (2–3 mL), precipitating the resulting solution into rapidly stirring methanol (20 mL) at 0 °C, and centrifuging this suspension to obtain a solid product. This precipitation procedure was repeated up to three times and the product thus obtained was dried under vacuum. Isolated yields were typically between 50–70%.

### 2.3 Computational methods

All calculations were carried out using Gaussian 16. The PBE0 functional with D3BJ dispersion correction was used in all cases.^54,55^ Most structures were optimized using the def2-TZVP basis set, except for the dimeric species and the Me_3_PO and Me_3_PS adducts of 3d and 3e which were optimized using the def2-SVP basis set for practical reasons.^52–55^ The reaction energies for formation of Me_3_PO and Me_3_PS adducts correspond to uncorrected single point energies calculated using the def2-TZVP basis set. FIA calculations were carried out using the def2-TZVP basis set and benchmarked to the Me_3_SiF/Me3Si^+^ couple for higher accuracy.^56^ UV-Vis calculations were carried out using TD-DFT with the def2-TZVP basis set from the optimized geometries or crystal structure geometries where appropriate.

## 3 Results & discussion

### 3.1 Syntheses

**Fig. 2:**
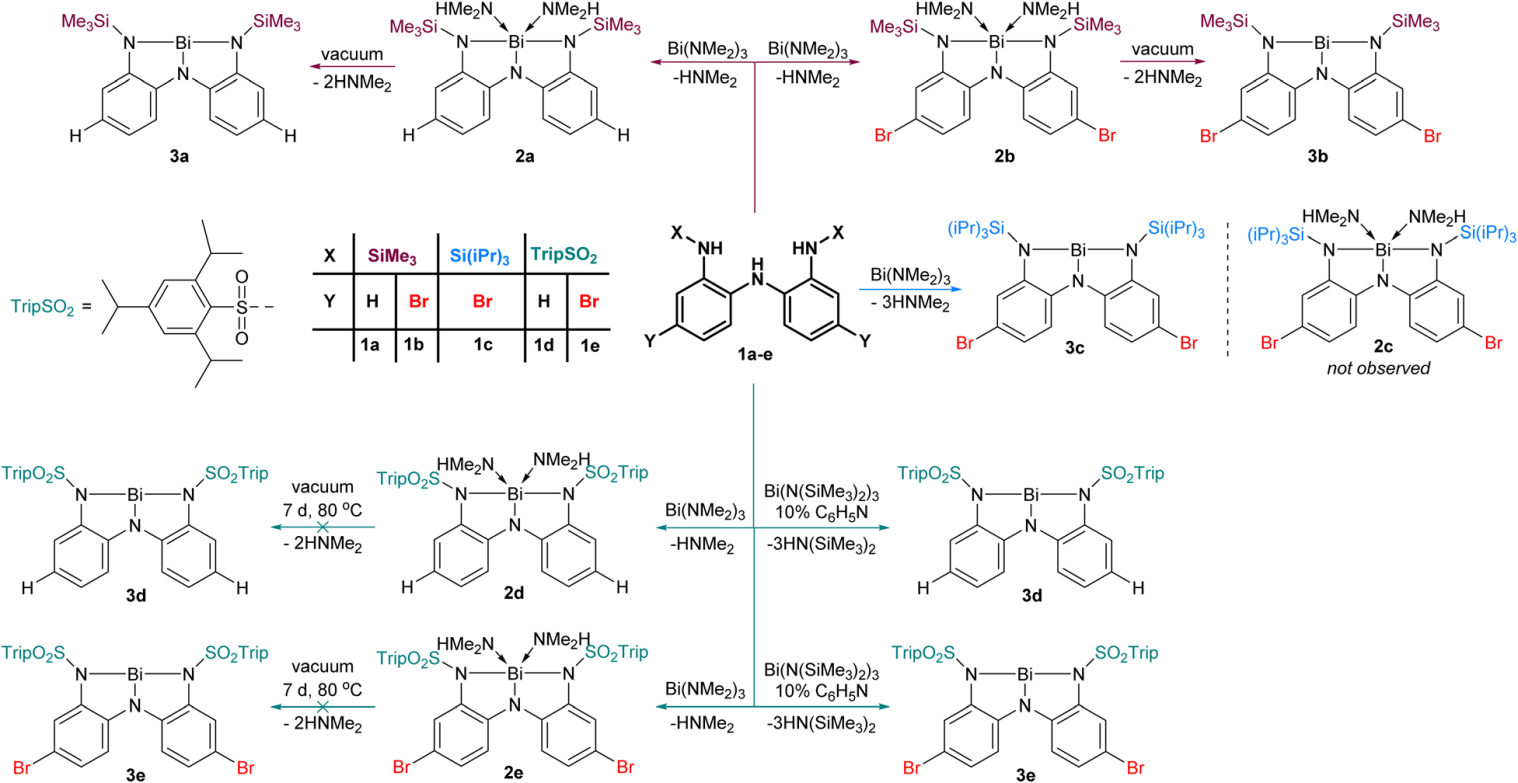
Metallation of ligands 1a–e to give amine adducts 2a, 2b, 2d, 2e or amine-free Lewis acids 3a–e.

We prepared five triamines (1a–e, Fig. 2), where the substituent X and Y were systematically varied to probe the effect of steric and electronic parameters. The conversion of 1a to 2a and subsequently to 3a upon removal of coordinated dimethylamine under vacuum was communicated previously.^31^ Adopting this approach, the reaction of 1b with Bi(NMe_2_)_3_ led to the formation first of 2b and then 3b upon application of vacuum. As we discuss later, 3b exists as a dimer in the solid state (3b_dim_) and as a monomer in solution. Replacement of the small SiMe_3_ groups with Si(^i^Pr)_3_ groups yielded 3c, which is monomeric in both solution and solid state. The steric bulk at nitrogen in ligand 1c is apparently sufficiently high that we detected no evidence of intermediate dimethylamine-bound adduct 2c*en route* to 3c (Fig. 2).

To exploit the electron-withdrawing nature of sulfonamide substituents, we reacted triamine 1d with Bi(NMe_2_)_3_. While this reaction proceeded smoothly to yield the amine adduct 2d, application of vacuum (10^−3^ mbar, 7 d, 80 °C) did not effect conversion to 3d. Presumably, the reduced steric protection and greater electrophilicity at the metal centre prevent dissociation of the volatile amines. We postulated that using a bulkier bismuth triamide might circumvent formation of amine adducts altogether. Thus, 1d–e was combined with Bi(N(SiMe_3_)_2_)_3_, and although evidence of HN(SiMe_3_)_2_ was detected, the reaction was found to be very slow, likely due to the steric bulk of the hexamethylsilazide fragment. We speculated that a small basic molecule could serve as a proton transfer catalyst to accelerate the transfer of protons from 1d–e to the hexamethylsilazide groups. After screening several bases, pyridine was found to be a convenient catalyst for this reaction. Using 10 mol% pyridine resulted in smooth conversion to 3d–e without the intermediacy of amine adducts because the HN(SiMe_3_)_2_ generated is too bulky to act as a ligand in this context.

Compounds 2d and 3a–e have been isolated and comprehensively characterized using melting points, elemental analysis, NMR, Infrared, and UV-Vis spectroscopies, as well as X-ray crystallography and high resolution mass-spectrometry. The amine adducts 2a–b are metastable entities that continually lose volatile Me_2_NH at variable rates (depending upon temperature, pressure, and particle size) and therefore could not be isolated. They have been spectroscopically characterized after being freshly generated in sealed NMR tubes (see ESI†).

### 3.2 Solid phase structures

**Fig. 3:**
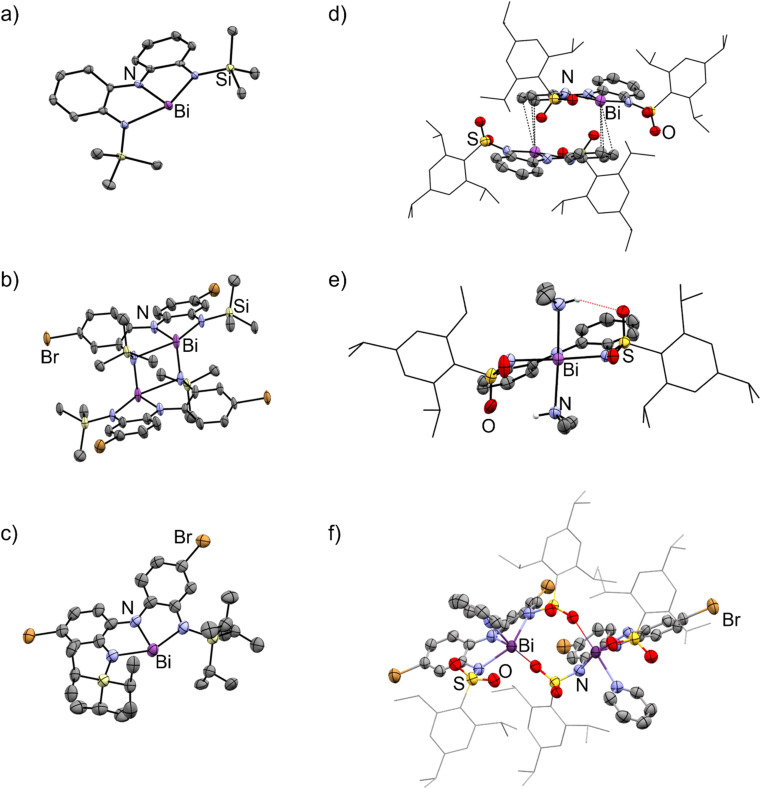
Molecular structures of (a) 3a, (b) 3b, (c) 3c, (d) 3d, (e) 2d, and (f) 3e·py in the solid state. Ellipsoids have been drawn at the 50% probability level. Hydrogen atoms and solvent molecules have been removed for clarity. The trip group has been shown in wireframe mode for clarity. Purple = bismuth, blue = nitrogen, red = oxygen, light yellow = silicon, yellow = sulfur, brown = bromine. See Fig. S115–S120† for additional views.

**Fig. 4:**
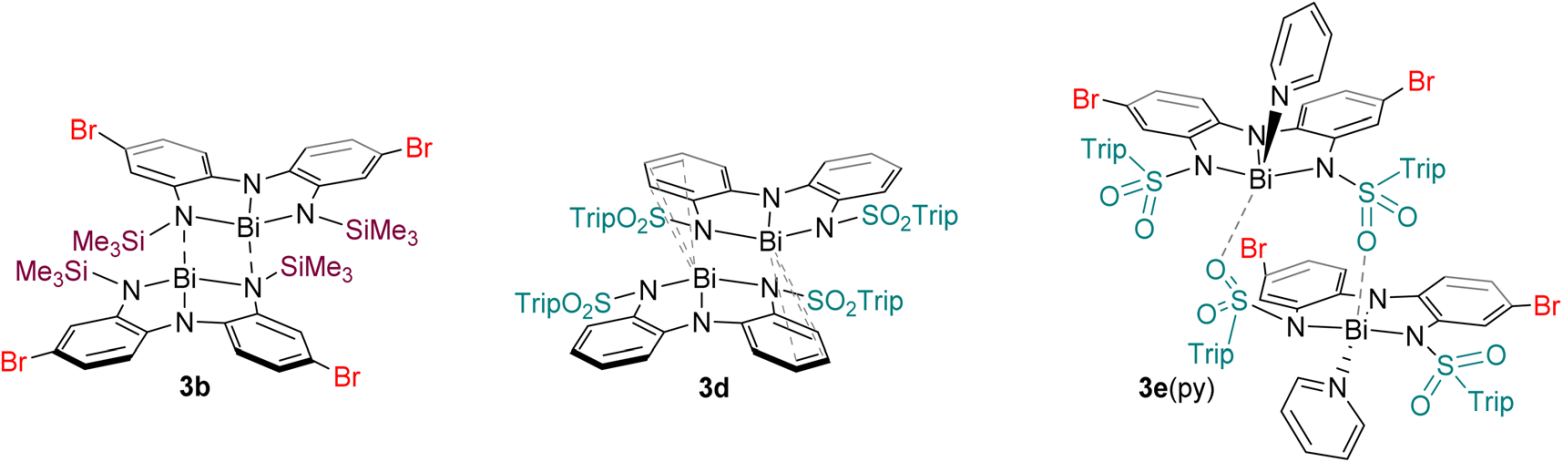
Intermolecular association modes observed for 3b, 3d, and 3e·py in the solid state.

The solid-state structures of 3a–e showed remarkable diversity (Fig. 3 and 4). Whereas 3a is monomeric in the solid state, 3b is dimeric due to the presence of Bi–N interaction between two units (Fig. 3a and b). We interpret this dimerization as evidence of enhanced Lewis acidity in monomeric 3b relative to 3a, in line with our expectations for a ligand appended with the electron-withdrawing bromine substituent. Interestingly, the Bi–N bonds between the two units are shorter (2.29 Å) than one of the Bi–N bonds within each unit of 3b (2.60 Å). On this basis, the geometry at each bismuth atom can be best described as being see-saw shaped, with three covalent interactions in a pyramidal arrangement and a fourth dative interaction trans to one of the covalent Bi–N bonds. This is similar to dimerization we have previously observed in related bismuth and antimony compounds.^32,43^ Data obtained for 3c is only suitable for connectivity, but it confirms that the compound is monomeric in the solid-state (Fig. 3c), with the large tri-isopropylsilyl substituents effectively thwarting coordination of both Me_2_NH (no 2c was detected) and dimerization. DFT calculations investigating the percent buried volume (% *V*_bur_)^57,58^ for 3c help to confirm this, finding a very large % *V*_bur_ of 70.2%, compared to the corresponding value of only 55.1% calculated for monomeric 3b (Table S3, ESI†).

The structure of 3d is dimeric, but here the assembly is held together by two η^3^ π-interactions involving the backbone arenes and the metal centres [Bi⋯C: 3.513(6)–3.695(7) Å]. While donor–acceptor interactions between heavy elements and arenes are well-known,^59^ it is unusual to see such interactions persist when heteroatoms are present in the system as the latter tend to be stronger donors than arenes. In the present case, we hypothesize that the dimerization does not occur *via* nitrogen atoms (as it does in 3b) because the electron-withdrawing sulfonyl group renders the nitrogen atoms less basic than the arenes, enabling the η^3^ arene-bismuth interactions to persist. Despite the numerous interactions, the geometry around the metal atom remains very close to planar, with a N–Bi–N–N dihedral angle of 177.54(1)°, suggesting these intermolecular interactions are quite weak. Indeed, the structure of compound 2d (Fig. 3e), provides a reasonable model for putative monomeric 3d. Although compound 3e was obtained in pure form, we were unable to grow crystals that were large enough for diffraction. Instead, a select few crystals of its pyridine adduct were fortuitously obtained, presumably from the catalytic amount of pyridine employed during the synthesis. The structure shows that one molecule of pyridine is bound to each metal centre, which is further coordinated to the oxygen atom from the sulfonamide fragment of a second molecule. Thus, at least three different intermolecular association modes are possible in the planar bismuth triamides prepared here, as summarized in Fig. 4.

### 3.3 Solution phase structures

The Lewis acid behaviour of the molecules studied would be influenced by the persistence of intermolecular interactions in the solution phase. The ^1^H NMR spectra of all derivatives show only one set of resonances for both silyl or sulfonamide substituents, which is consistent with a planar monomeric geometry. However, the timescale of the NMR experiment (milliseconds) is slow enough that rapid side-to-side motion that renders the groups equivalent and averages out their chemical shifts cannot be discounted.^60^ Since nuclear motions are much slower than electronic excitations, we envisioned that UV-Vis spectroscopy would probe the ‘instantaneous’ solution phase geometry, an approach we have used previously to study such dynamic processes.^60^ In this system, UV-Vis spectroscopy provides insights into the availability of the 6p atomic orbital for excitation. In the planar, monomeric geometry, the vacant low-energy 6p orbital participates in HOMO → LUMO and HOMO−1 → LUMO excitations, exhibiting absorption bands in the 500–700 nm range. In the dimeric structures, higher energy σ* antibonding MOs are involved in the first excited state, resulting in absorptions in the 375–475 nm range.

**Fig. 5:**
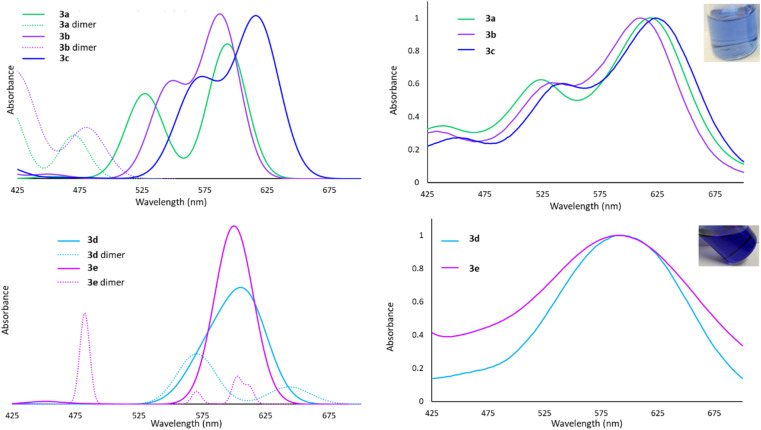
Top left: Calculated UV-Vis spectra for 3a–c and 3a, 3b dimers. Top right: Experimental UV-Vis spectra for 3a–c (absorbance normalized to 1.0), inset showing solution colour of 3b. Bottom left: Calculated UV-Vis spectra of 3d, 3e and their dimers. Bottom right: Experimental UV-Vis spectra of 3d (absorbance normalized to 1.0), 3e, inset showing solution colour of 3d.

The experimental spectra for 3a–c in toluene are shown in Fig. 5 and, in each case, two bands are observed at long wavelengths and their relative intensities approximates the calculated oscillator strengths for the associated transitions for the monomeric forms. We conclude that 3a–c exist primarily as monomers in solution, despite the dimeric structure being detected in the solid state for 3b. The spectral bands for 3d and 3e are very broad, but nevertheless centered very prominently at 600 nm, where TD-DFT calculations predict the monomeric entities absorb. However, due to the calculated maxima for the dimers also being in the 500–650 nm range, we cannot definitively rule out the presence of a small population of dimers in equilibrium with the monomeric forms.

This analysis is supported by molecular weight estimates derived from diffusion ordered NMR spectroscopy (DOSY). Diffusion coefficients for 3b–d measured by DOSY NMR in C_6_D_6_ were fitted to Grubbs' calibration curve^61^ to give molecular weight estimates. Dilute solutions of 3b–d were calculated to have molecular weights corresponding to monomer units (Table S1†). Only a concentrated solution of 3d gave a DOSY-estimated molecular weight that exceeded the theoretical molecular weight of the monomer, suggesting formation of dimers in the high concentration regime, but a predominantly monomeric formulation at low concentrations, corroborating the UV-Vis analysis.

We therefore conclude that despite the presence of at least three different dimerization modes possible in the solid phase, compounds 3a–e are primarily monomeric even in weakly coordinating solvents (*e.g.* toluene). For 3d and 3e a small percentage of dimers may be present in solution.

### 3.4 Electrophilicity assessments *via* Gutmann–Beckett experiments

As a well-benchmarked and simple experimental measure of Lewis acidity, the Gutmann–Beckett method was chosen to assess the Lewis acidity of 3a–e.^62^ We have previously shown that up to two O-atom donors can be accommodated within the coordination sphere of bismuth in such compounds.^60^ To favour 1 : 1 adducts for comparison with literature data, a five-fold excess of the Lewis acid was employed based on titration experiments suggesting asymptotic behaviour was reached at this ratio (see Fig. S8–S16†). Although these titration experiments suggest 1 : 1 adducts are predominant, the persistence of a small amount of 2 : 1 adducts cannot be ruled out definitively. In order to further assess the hard/soft character of these acids an extension to the Gutmann–Beckett method was also employed whereby trimethylphosphine sulfide was used in place of triethylphosphine oxide.^63^

As shown in Table 1, silane substituted complexes displayed modest Lewis acidity with Et_3_PO acceptor numbers of 18, 33 and 18 for 3a–c, respectively. The increase in value from 3a to 3b is consistent with the enhanced Lewis acidity of the latter arising from the dibromination of the backbone. The decreased acceptor number for 3c is surprising at first glance but may be a consequence of the increased steric shielding of the metal centre by the bulky Si(^i^Pr)_3_ groups, which might disfavour coordination of Et_3_PO.^64^ Indeed, a conspicuously broad ^31^P NMR resonance was observed for this derivative, consistent with an association–dissociation equilibrium (Fig. S1, ESI†). Significantly enhanced Lewis acidity was observed for 3d and 3e, which showed acceptor numbers of 65 and 69, respectively. These values evidence the strong electron-withdrawing effect of the sulfonamide group and reiterate the electrophilicity boost imparted by bromide substitution in the backbone.

**Table 1:** Gutmann–Beckett acceptor numbers for 3a–3e and B(C_6_F_5_)_3_ with Et_3_PO and Me_3_PS

Compound	Et_3_PO	Me_3_PS
3a	18	1.0
3b	33	4.3
3c	18	1.2
3d	65	68
3e	69	72
3b + py	33	3.2
3b + 2py	30	0.9
3d + py	56	7.5
3d + 2py	55	3.0
B(C_6_F_5_)_3_	76	51

Complexes 3a–c showed very low acceptor numbers (less than 5) in the Me_3_PS system, indicating these are likely weak soft Lewis acids. For complexes 3d and 3e the Me_3_PS acceptor numbers observed of 68 and 72, respectively, are very high and representative of strong soft Lewis acids. Notably, the acceptor numbers observed with Me_3_PS for 3d and 3e exceed the value of the very strong Lewis acid B(C_6_F_5_)_3_.^63^ To ensure that sterics were not playing a significant role, these experiments were repeated using Et_3_PS, revealing very similar results (see Fig. S3, ESI†), thereby suggesting that slight variations in steric bulk of the probe molecule do not alter the overall trends.

To further probe the hard/soft character of 3a–e pyridine was added to observe the effect on the Et_3_PO and Me_3_PS acceptor numbers.^63^ For Me_3_PS a large decrease in acceptor number was observed with the addition of 1 or 2 equivalents of pyridine to both 3b and 3d, as representatives of the silyl and sulfonamide classes (Fig. S4, S5, ESI†). Preferential binding to pyridine over Me_3_PS further suggests a preference for binding hard substrates, despite the high acceptor number of 3d with Me_3_PS. When pyridine was added to the Et_3_PO bound Lewis acids, however, only a small decrease in acceptor number was observed for both bismuth complexes. Taken together these results suggest that compounds 3a–e prefer binding to oxygen or nitrogen bases over sulfur, which is in contrast to expectations for a large, neutral, and polarizable 6^th^-row element centre.

We also attempted to use the recently developed fluorescent Lewis acid-base adduct method^65^ to probe Lewis acidity in solution, but these efforts were thwarted by the complex photophysical properties of compounds 3a–e.

### 3.5 Electrophilicity assessments *via* calculated Me_3_PO and Me_3_PS affinities

**Fig. 6:**
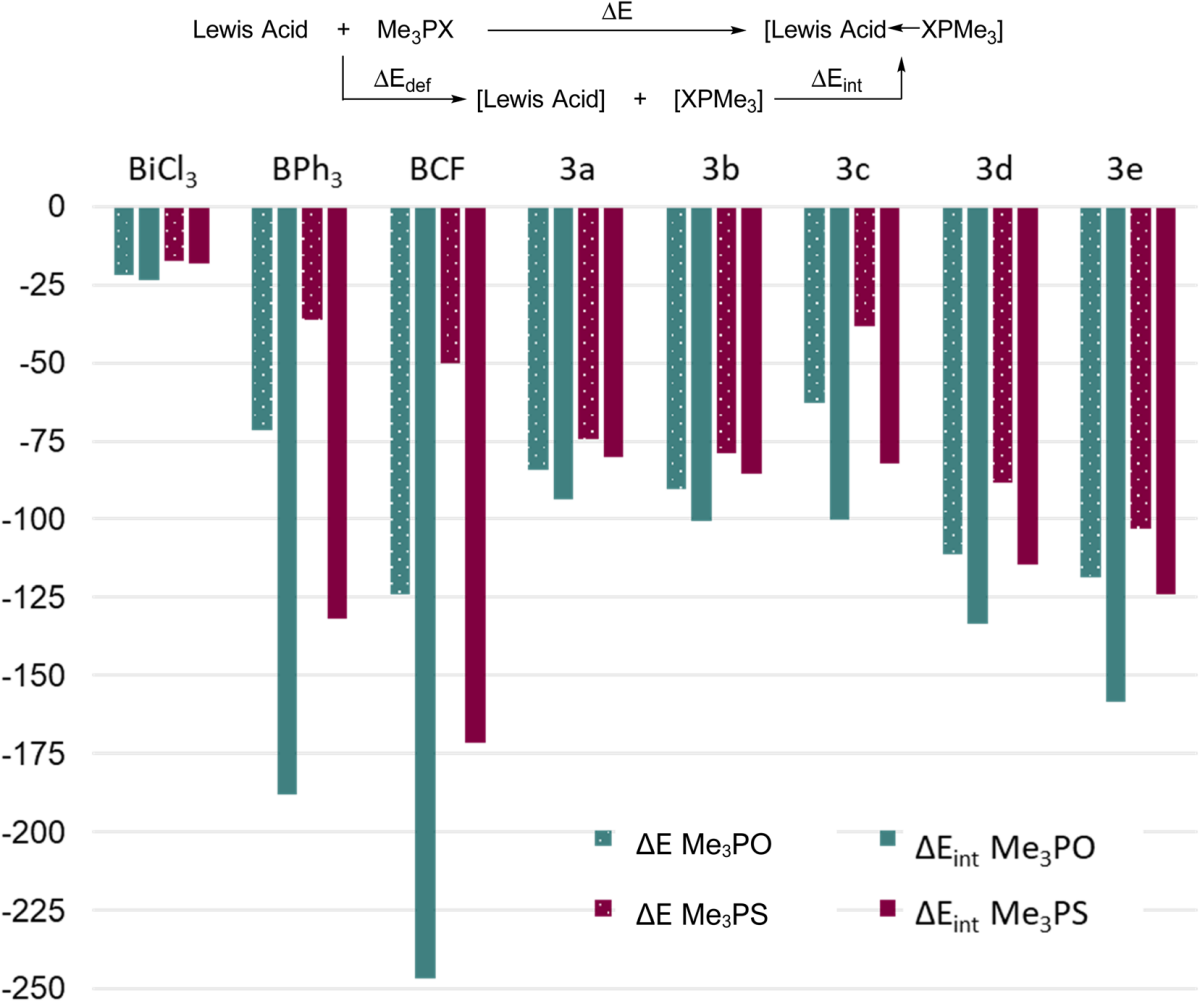
Calculated binding affinities of B(C_6_F_5_)_3_ (BCF), BPh_3_, BiCl_3_ and 3a–e with Me_3_PO and Me_3_PS.

We note that recently it has been reasonably argued that acceptor numbers may not necessarily reflect binding affinities.^64^ DFT calculations (PBE0/def2-TZVP, see experimental) were therefore performed to explicitly determine the reaction energies for the formation of 1 : 1 adducts between Me_3_PO or Me_3_PS and some boron and bismuth Lewis acids, thereby providing insights about interaction energies towards prototypical hard or soft donors (Fig. 6). We calculated the reaction energy (Δ*E*) as well as the intrinsic bond energy (Δ*E*_int_) for adduct formation in each case (see definition in Fig. 6). The quantity Δ*E* represents the energy change associated with free reactants starting from their equilibrium geometries and assembling to give the respective adduct, whereas Δ*E*_int_ represents a process starting from reactants that are pre-distorted to the geometry found in the adduct. Thus, Δ*E*_int_ quantifies only the strength of the bond formed, while disregarding the energy cost of deforming the fragments (Δ*E*_def_) into their final geometries. The methyl substituents were chosen both for computational simplicity and to remove any steric differences.

Considering first the Δ*E* values, the results showed the same trend as experimental results for Lewis acidity, with 3d and 3e showing greater reaction energies than 3a–c. As expected, 3c showed an anomalously low value due to steric factors. Three key points become evident: (i) the Me_3_PO binding affinities of the bismuth triamides (ignoring 3c due to steric factors) are greater than the corresponding value for BPh_3_, and the values for 3d and 3e even approach that for B(C_6_F_5_)_3_ (ii) the Me_3_PS binding affinity of all bismuth acids (except 3c) is greater than the corresponding value for the borane acids and (iii) the *difference* between Me_3_PS and Me_3_PO binding affinities for a given Lewis acid is much smaller for bismuth derivatives compared to boron ones. Collectively, these results indicate that although the bismuth Lewis acids preferentially bind hard Lewis bases (consistent with our experimental findings), they show a considerably higher affinity for softer bases than boranes. Thus, planar bismuth triamides are suitable for coordination and activation of *either* soft or hard substrates, whereas boranes are best suited for activation of hard substrates.

The Δ*E*_int_ values of the bismuth triamides is comparable to their Δ*E* values, indicating that the large bismuth centre does not experience a significant geometric distortion upon coordination in these compounds. The notable exception is the sterically very encumbered derivative 3c, where comparison of Δ*E* and Δ*E*_int_ shows a significant difference due to the repulsion created by large triisopropylsilyl groups. The boron Lewis acids, in contrast, show stark differences between their Δ*E* and Δ*E*_int_ values, highlighting the relatively higher steric crowding at the small boron atoms upon coordination.

Finally, we note that all bismuth triamides are significantly stronger acceptors towards both hard and soft acids than prototypical halide BiCl_3_, which is pyramidal and expresses its Lewis acidity *via* σ* antibonding orbitals. Thus geometrically induced Lewis acidity in the planar form can exceed values obtained even when using strongly electron withdrawing groups (*e.g.* chloride) in the pyramidal form. We interpret the much greater acidity expressed by the 6p orbital in 3a–e as being a result of atomic orbitals generally lying significantly lower than antibonding orbitals – thus the geometry of the triamides is key to their enhanced electrophilicity. Additionally, fluoride ion affinities were also calculated for each complex, showing similar Lewis acidity trends (Table S2, ESI†).

### 3.6 Catalytic ring opening polymerization of ε-caprolactone and *rac*-lactide

**Fig. 7:**
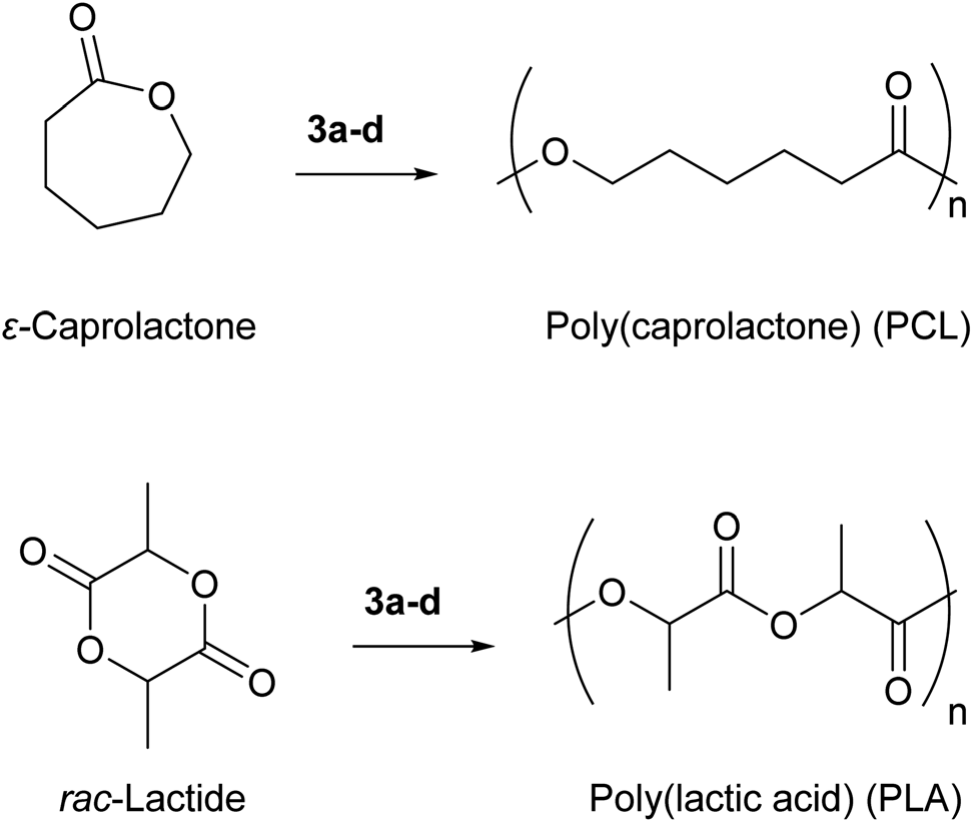
Ring opening polymerization of ε-caprolactone and *rac*-lactide by bismuth complexes 3a–d.

As the electrophilicity assessment above indicate, compounds 3a–e span a wide range in Lewis acid strengths and show an unexpectedly high (for a 6^th^-row element) affinity for oxygen-based hard donors. To exploit these features in the context of Lewis acid catalysis, we investigated their use in the polymerization of cyclic esters ε-caprolactone and lactide to give poly(caprolactone) (PCL) and poly(lactic acid) (PLA), respectively (Fig. 7). Both PCL and PLA are valuable commercial polymers with approved applications as a biodegradable scaffolds for tissue engineering and materials for construction of implanted medical devices.^66^ These polymers are also appealing from a sustainability perspective as they can be degraded in nature or on-demand to small molecules, unlike most petroleum-derived plastics which persist in the ecosphere.^67^ Their properties can also be easily tuned due to the ease of blending with other bioderived polymers.^68,69^

The most commonly used industrial catalyst for the ROP of lactone monomers is tin(ii) 2-ethylhexanoate,^70,71^ which has been FDA-approved for some years, but is now under renewed scrutiny due to toxicity concerns arising from *in vivo* accumulation and damage to marine ecosystems where end of life degradation of plastics often occurs.^72–74^ As a result, the FDA approved limit for tin is set at <15 ppm for *in vivo* usage.^75^ However, since the tin content in crude samples of PCL or PLA is typically in the 1000–2000 ppm range, significant efforts must be devoted towards removing heavy metal residue from the final product before medical-grade materials can be accessed.^76,77^ Moreover, tin(ii) 2-ethylhexanoate participates in catalytic transesterification reactions involving the formed polymer, lowering control over the molecular weight and increasing the dispersity of the material formed, thereby influencing mechanical properties.^70^

On the other hand, bismuth compounds are generally accepted as being non-toxic even in high doses, motivating their use in polymerization of cyclic esters.^78^ While even simple bismuth halides or alkoxides are able to catalyse these polymerizations, the resultant polymers are either low molecular weight materials or polydisperse, rendering them unsuitable for commercial usage.^79,80^ Bi(N(SiMe_3_)_2_)_3_, the bismuth precursor in our synthesis, has also been shown to be an effective catalyst for this polymerization, however the resulting polymers also suffer from high polydispersity.^81^ Ligand design has enabled bismuth-based systems for ROP which achieve much better results and will be discussed in more detail below. Full polymerization data for ε-caprolactone and *rac*-lactide polymerized by complexes 3a–d is presented in Table 2.

**Table 2:** Conditions and results for the ring opening polymerization (ROP) of ε-caprolactone and *rac*-lactide catalyzed by 3a–d

	Catalyst	Monomer : I ratio	Conversion^a^ (%)	Calc. *M*_n_^b^ (kg mol^−1^)	Expt. *M*_n_^c^ (kg mol^−1^)	Expt. *M*_w_^c^ (kg mol^−1^)	*Đ* c
**Monomer = ε-caprolactone**							
1^d^	3a	5000	5	27.4	62.85	82.03	1.31
2^d^	3b	1000	98	111.7	232.6	266.7	1.15
3^d^	3b	5000	86	491.3	289.6	316.0	1.09
4^e^	3b	5000	95	539.2	177.7	207.9	1.17
5^f^	3b	5000	N/A	N/A	99.24	131.6	1.33
6^d^	3c	1000	70	79.8	82.31	112.8	1.37
7^d^	3d	1000	7	8.1	N/A	N/A	N/A

**Monomer = *rac*-lactide**							
8^d^	3a	2000	98	282.2	176.2	230.3	1.31
9^d^	3b	2000	83	239.0	105.9	152.9	1.44
10^d^	3b	5000	78	561.6	55.43	63.62	1.15
11^e^	3b	2000	95	273.6	118.2	155.2	1.31
12^f^	3b	5000	N/A	N/A	228.9	500.2	2.20
13^d^	3c	2000	90	259.2	84.91	130.7	1.54
14^d^	3d	2000	22	63.4	31.33	35.69	1.14

a Determined from ^1^H NMR integration. Note that this is not equal to isolated yields which were typically in the range of 50–70%.

b Calculated by from the conversion by ^1^H NMR and the molecular weight of the corresponding monomer.

c Triple detection GPC with THF eluent, flow rate = 0.30 mL min^−1^, 25 °C; *Đ* = *M*_w_/*M*_n_ (see Experimental for full details).

d Conditions: 80 °C; C_6_H_6_; 16–40 hours.

e Conditions: 120 °C; 1,2-dichlorobenzene; 6–12 hours.

f Conditions: 190 °C; neat; 16 hours.

All complexes tested were found to be capable of catalyzing the polymerization with varying degrees of success. For ε-caprolactone, 3a and 3c showed slow conversion, likely due to the lower Lewis acidity of the former, and the high steric bulk of the latter (entries 1, 6). The resulting polymers exhibit reasonably high *M*_n_ values in the 60–80 kg mol^−1^ range and dispersity values around 1.3. In contrast, the very strongly Lewis acidic 3d showed surprisingly low polymerization activity even when used with higher catalyst loadings (entry 7). This can be attributed to either steric barriers arising from the very large triisopropyl phenyl group, or potentially due to the strong Lewis acidity precluding further reaction once the monomer is coordinated to the bismuth centre. The percent buried volume calculations support the higher degree of steric bulk in 3c (% *V*_bur_ = 70.2%) and 3d (% *V*_bur_ = 77.3%) compared with 3a (% *V*_bur_ = 55.1%). For 3b, excellent polymerization results were found under a variety of conditions. Catalyst loadings as low as 0.02 mol% could be used to effect nearly complete conversion at 80 °C. The isolated polymers showed high *M*_n_ values of nearly 300 kg mol^−1^ and, crucially, dispersities under 1.3 (entries 2–4). Attempting to accelerate the rate of polymerization by increasing the temperature to 120 °C showed improved turnover frequency further at a marginal cost to molecular weight and dispersity (entry 4).

Bismuth complexes 3a–d were also tested in the polymerization of *rac*-lactide, yielding materials in the 55–150 kg mol^−1^ range (*Đ* = 1.15–1.54) in solution phase reactions. Similar to the ε-caprolactone polymerization, the polymerization of lactide was found to proceed slowly when complexes 3c or 3d were used (entries 13 and 14). Complexes 3a and 3b were found to give the highest molecular weights with low dispersities at loadings ≤0.1 mol% (entries 8–11). With complex 3b, even lower catalysts loadings (0.02 mol%) were tolerated.

A wide array of main group catalysts have been previously reported for the ring opening polymerization of both ε-caprolactone and *rac*-lactide.^82^ Compound 3b is able to produce polyesters with dispersity and molecular weights values comparable to the best performing main group catalysts based on other biocompatible metals like indium,^83^ germanium,^84^ zinc,^85,86^ or gallium.^87^ However, the rate of polymerization found by 3b is slow (requiring several hours) when compared to the best performing systems (requiring minutes). In contrast, compared to other bismuth systems utilized for this polymerization, 3b is able to produce polymers with high molecular weights and lower dispersity.^88–94^ For example, diphenyl bismuth bromide or bismuth subsalicylate are able to proceed at greater rates and produce polymers of similar (or higher) molecular weights, the dispersities reported for these systems are higher (1.5–2) than observed here.^93,94^

**Fig. 8:**
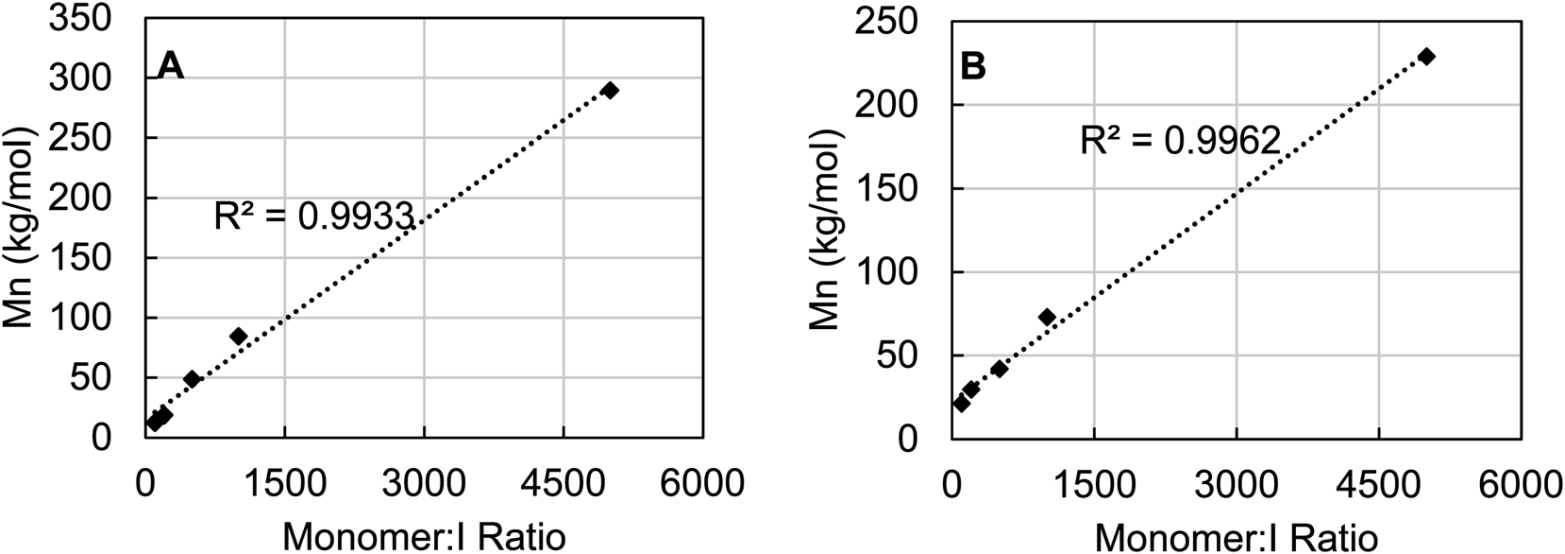
Monomer to initiator ratio plotted against the *M*_n_ of the resulting polymer for ε-caprolactone (A) and *rac*-lactide (B). All polymerizations were run at 80 °C until >80% conversion was observed by ^1^H NMR.

To probe the degree of control over molecular weight control which could be achieved using 3b, the catalyst loading of polymerizations of ε-caprolactone and *rac*-lactide were varied to observe the effect on *M*_n_. For both polymers it is clear that the molecular weight is tunable by varying the catalyst loading (Fig. 8). A linear correlation between *M*_n_ and monomer : I ratio was found in both cases across a wide range from 100–5000 equivalents of monomer. Additionally, monitoring the polymerization of ε-caprolactone by 3b revealed pseudo-first order kinetics after an induction period of about four hours (see Fig. S90, ESI†).

While a detailed mechanistic investigation of the ring opening polymerization has not been undertaken, MALDI-TOF MS results on oligomeric materials indicate end groups having a mass equal to that of (Me_3_Si)_2_NH (Fig. S112–S115, ESI†). This suggests a mechanism where trace residual bis(trimethylsilyl)amine acts as an initiator, with the methanol work-up acting as a terminating step (see ESI†). Although analytically pure 3d was used, which is expected to be free of bis(trimethylsilyl)amine, it is possible the trace amounts remaining are key to the polymerization. Interestingly, no conversion was observed when the oft-used benzyl alcohol was added to the system as an initiator.

We also tested complex 3b under solvent-free, high temperature conditions representative of existing industrial reactors, where the monomer and polymer are both present as melts to promote mass transfer and avoid material build-up. In these experiments, PCL of *ca.* 80 kg mol^−1^ (*Đ* = 1.33) and PLA of *ca.* 250 kg mol^−1^ (*Đ* = 2.2) were obtained (entries 5 and 12), identifying 3b as being effective under industrial conditions, and making it a potential drop-in tin replacement.

## 4 Conclusions

We have reported a library of planar, neutral, trivalent bismuth complexes which show inductive variation in Lewis acidity at the bismuth centre by rational tuning of substituents on the triamide ligand. Lewis acidity assessments including the Gutmann–Beckett method and calculated binding affinities have revealed values approaching those of halogenated triarylboranes. However, unlike boranes, planar bismuth compounds exhibit both hard and soft acidity while suffering a minimal energy penalty for geometric distortion, due to the large coordination sphere of the 6^th^ row metal. The Lewis acidity of complexes 3a–d was exploited to achieve the first example of catalytic polymerization with planar pnictogen compounds. Specifically, we demonstrated the ring opening polymerization of ε-caprolactone and *rac*-lactide, for which compound 3b appears to be one of the most effective main-group catalysts reported in terms of the high TON (*ca.* 5000), mild polymerization conditions (80 °C), very high molecular weight (*ca.* 300 kg mol^−1^ for PCL and 120 kg mol^−1^ for PLA), and exceptionally low dispersities (*ca.* 1.1). Complex 3b also operates at the high temperatures currently used in industrial reactors for these commodity materials. Considering the low toxicity of bismuth, these features highlight planar bismuth triamides as potential alternatives to tin catalysts in the production of medical-grade PCL and PLA. Future studies will be done to investigate the mechanism of ring opening polymerization to provide a better understanding of the catalytic behaviour.

Collectively, these results establish planar bismuth triamides as an inductively-tunable, neutral, main group platform for hard or soft Lewis acidity. Compared to previously shown examples of Coulomb or resonance tuning, such inductive tuning is synthetically easier and more modular, making it overall more accessible. Our results also demonstrate the first application of geometrically-distorted pnictogen centres in polymerization catalysis, identifying a new field of applications for a rapidly growing and fascinating class of molecules.

## Data availability

Crystallographic data has been deposited with the Cambridge Structural Database.

## Author contributions

TJH synthesized and characterized most of the compounds, carried out spectroscopic studies, completed all DFT calculations, and contributed to manuscript writing. WMM conducted the ε-caprolactone polymerization experiments. TK conducted the *rac*-lactide polymerization experiments. JB contributed DOSY NMR experiments. JM carried out the Gutmann–Beckett experiments. TH synthesized some of the ligands. KLB performed crystallography experiments. CV and CMK performed the molecular weight measurements and MALDI experiments and analyses. TG and JDM performed crystallography experiments. SSC conceived and supervised the project and contributed to manuscript writing.

## Conflicts of interest

There are no conflicts to declare.

## Supplementary Material

SC-014-D3SC00917C-s001

SC-014-D3SC00917C-s002

SC-014-D3SC00917C-s003
