# Supplementary material for: Planar bismuth triamides: a tunable platform for main group Lewis acidity and polymerization catalysis
Source: Chem Sci. 2023 Apr 6;14(17):4549–63. doi: 10.1039/d3sc00917c (PMC10155930; doi:10.1039/d3sc00917c)
Supplement: SC-014-D3SC00917C-s002 [file SC-014-D3SC00917C-s002.pdf]

Electronic Supplementary Material (ESI) for Chemical Science.
This journal is © The Royal Society of Chemistry 2023

# Planar Bismuth Triamides: A Tunable Platform for Main Group Lewis Acidity and Polymerization Catalysis

Tyler J. Hannah,[a] W. Michael McCarvell,[a] Tamina Kirsch,[a] Joseph Bedard,[a] Toren Hynes,[a] Jacqueline Mayho,[a] Karlee L. Bamford,[a] Cyler W. Vos,[b] Christopher M. Kozak,[b] Tanner George,[c] Jason D. Masuda,[c] S. S. Chitnis[a]*

[a] Chemistry Department, Dalhousie University, 6274 Coburg Rd, Halifax, NS, B3H 4R2, Canada

[b] Department of Chemistry, Memorial University of Newfoundland, St. John's, NL, A1B 3X7, Canada

[c] Department of Chemistry, St. Mary's University, 923 Robie St., Halifax, NS, B3H 3C3, Canada

* Corresponding author: saurabh.chitnis@dal.ca

# Supplementary Information

# Contents

Experimental ........ 3
Synthesis of L1: ........ 3
Synthesis of L2: ........ 4
Synthesis of 2b: ........ 4
Computational Methods ........ 4
Additional Figures Referenced in Manuscript: ........ 5
DOSY NMR Data ........ 16
Calculated Fluoride Ion affinities ........ 21
Percent Buried Volume Calculations ........ 22
Computational Data: ........ 23
Characterization Data: ........ 28
Polymerization Data: ........ 63
$^{1}$H-NMR ........ 63
Gel Permeation Chromatography Traces ........ 88
MALDI-TOF Mass Spectrometry ........ 109
Alternate X-Ray Crystal Structure Images ........ 113
References: ........ 117

# Experimental

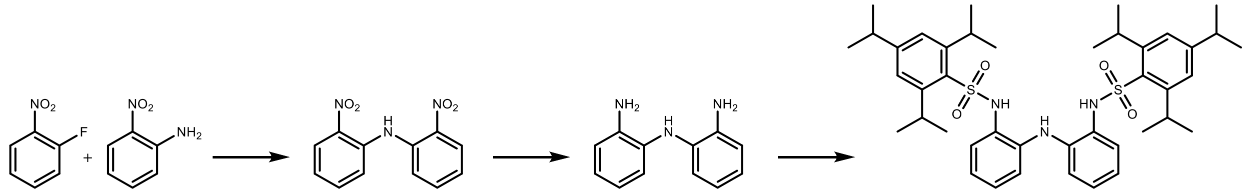

**S1** **S2** **S3**

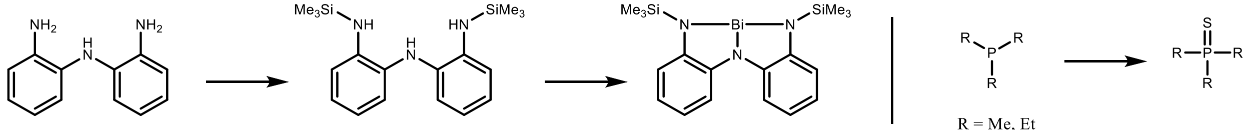

**S2** **1a** **3a**

R = Me, Et

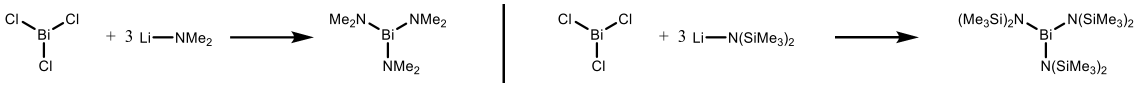

**S1**[1], **S2**[2], **S3**[3], **1a**[4], **3a**[5], $Bi(NMe_2)_3$[6], BiHMDS[7], $Me_3PS$, and $Et_3PS$[8] were prepared as previously reported.

## Synthesis of L1:

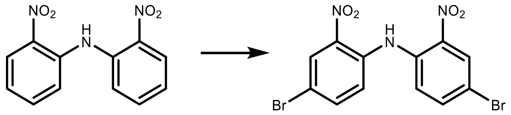

**S1** **L1**

**S1** (13.95 g, 53.8 mmol) was suspended in acetic acid (500 mL). $Br_2$ (29.7 g, 215 mmol) was added slowly, and the reaction mixture was refluxed for 16 hours. Mixture was then cooled to room temperature and distilled water (500 mL) was added. The suspension was filtered and washed with additional water (2 x 100 mL), collecting the orange solid **L1** (87 %, 19.46 g). mp > 260 °C.

Elemental Analysis: Found: C, 35.0; H, 1.85; N, 9.6. Calc. for $C_{12}H_7Br_2N_3O_4$: C, 34.6; H, 1.7; N, 10.1.

$^1$H NMR: $\delta_H$ (500 MHz, $CDCl_3$) 10.89 (1 H, s, N-H), 8.36 (2 H, d, *J* 2.4, Ar-H), 7.63 (2 H, dd, *J* 9.0, 2.3, Ar-H), 7.42 (2 H, d, *J* 8.9, Ar-H).

$^{13}$C NMR: $\delta_C$ (126 MHz, $CDCl_3$) 137.83 ($C_{Ar}$), 135.82 ($C_{Ar}$), 129.39 ($C_{Ar}$), 121.10 ($C_{Ar}$), 113.94 ($C_{Ar}$).

ESI-HRMS (negative ion mode): calculated for $[C_{12}H_6Br_2N_3O_4]^-$ = 413.8731 m/z, observed = 413.8730 m/z

## Synthesis of L2:

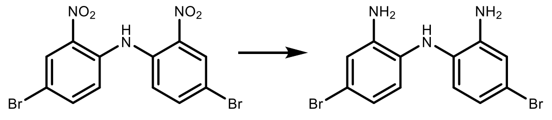


**L1** (19.2 g, 46.0 mmol), zinc dust (39.1 g, 598 mmol), and ammonium chloride (29.5 g, 552 mmol) were weighed out into a round bottom flask. THF (250 mL) was added, and the suspension was refluxed until the orange colour was no longer present (approximately 24-48 hours). The reaction mixture was cooled to room temperature and filtered through celite. The filtrate was evaporated to dryness resulting in a brown solid (71 %, 11.7 g). mp 137-140 °C.

$^{1}$H NMR: $\delta_H$ (500 MHz, $CDCl_3$) 6.91 (2 H, d, *J* 2.2, Ar-H), 6.83 (2 H, dd, *J* 8.3, 2.2, Ar-H), 6.56 (2 H, d, *J* 8.3, Ar-H), 4.85 (1 H, s, N-H), 3.67 (4 H, s, $NH_2$).
$^{13}$C NMR: $\delta_C$ (126 MHz, $CDCl_3$) 140.00 ($C_{Ar}$), 129.74 ($C_{Ar}$), 122.50 ($C_{Ar}$), 121.77 ($C_{Ar}$), 119.13 ($C_{Ar}$), 116.20 ($C_{Ar}$).
ESI-HRMS (negative ion mode): calculated for $[C_{12}H_{10}Br_2N_3]^-$ = 353.9247 m/z, observed = 353.9237 m/z

## Synthesis of 2b:

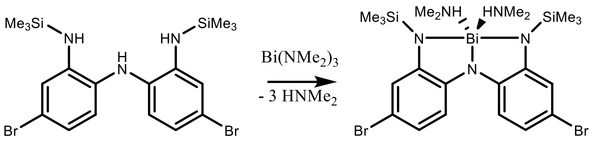


$Bi(NMe_2)_3$ (14 mg, 0.04 mmol) and **1b** (20 mg, 0.04 mmol) were dissolved in $C_6D_6$ (0.6 mL) and immediately sealed in an NMR tube, resulting in a colour change to red. After 10 minutes mixture was analyzed by NMR.

$^{1}$H NMR: $\delta_H$ (500 MHz, $C_6D_6$) 7.52 (2 H, d, *J* 8.6, Ar-H), 7.26 (2 H, d, *J* 2.3, Ar-H), 6.85 (2 H, dd, *J* 8.5, 2.3, Ar-H), 3.22 (3 H, s, **H**N($CH_3$)$_3$), 1.81 (18 H, d, *J* 6.4, HN(**CH$_3$**)$_3$), 0.29 (18 H, s, Si($CH_3$)$_3$).
$^{13}$C NMR: $\delta_C$ (126 MHz, $C_6D_6$) 149.46 ($C_{Ar}$), 148.22 ($C_{Ar}$), 122.92 ($C_{Ar}$), 118.30 ($C_{Ar}$), 117.54 ($C_{Ar}$), 112.96 ($C_{Ar}$), 37.89 (HN($CH_3$)$_2$), 2.23 (Si($CH_3$)$_3$).

## Computational Methods

All calculations were carried out using Gaussian 16. The PBE0 functional with D3BJ dispersion correction was used in all cases. FIA calculations were carried out using the def2-TZVP basis set and benchmarked to the $Me_3SiF$/$Me3Si^+$ couple for higher accuracy.[9]

# Additional Figures Referenced in Manuscript:

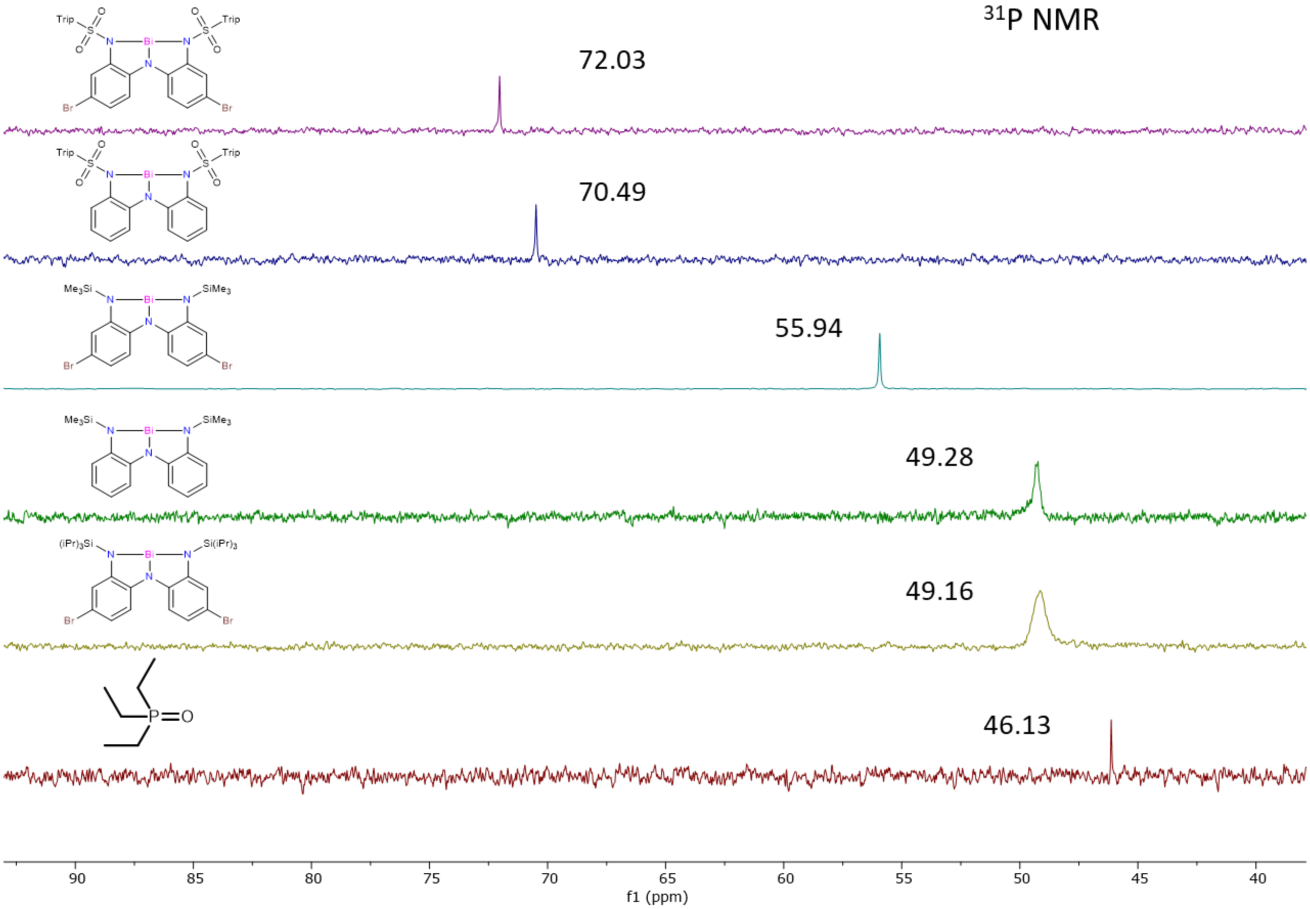


**Figure S1.** Gutmann-Beckett results of Lewis acids combined with $Et_3PO$ in a 5:1 ratio. All spectra are recorded in $C_6H_6$ and referenced to the residual solvent peak in $^{1}$H NMR, corrected for $^{31}$P. $^{31}$P {$^{1}$H} NMR (121 MHz, $C_6H_6$).

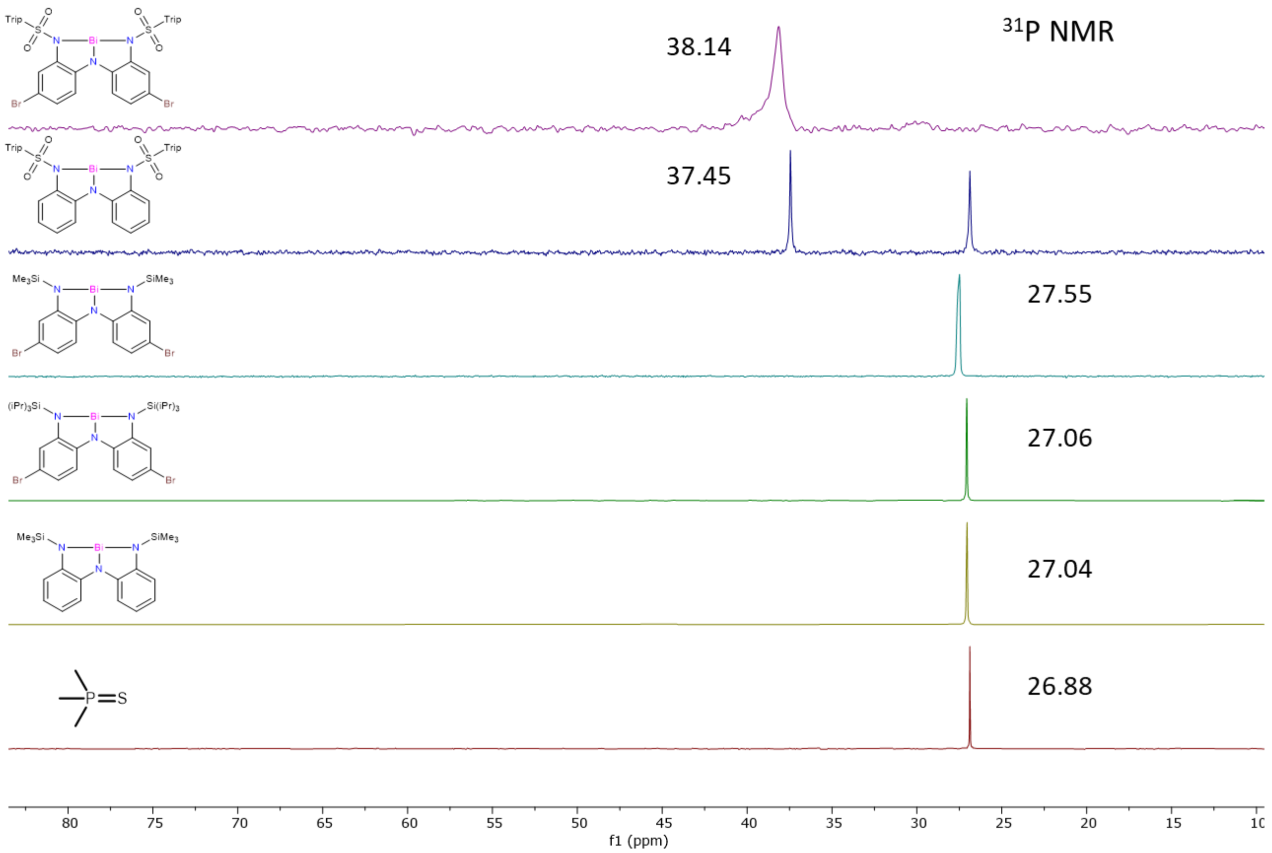


**Figure S2.** Gutmann-Beckett results of Lewis acids combined with $Me_3PS$ in a 5:1 ratio. All spectra are recorded in $C_6H_6$ and referenced to the residual solvent peak in $^1H$ NMR, corrected for $^{31}P$. $^{31}P\{^1H\}$ NMR (121 MHz, $C_6H_6$).

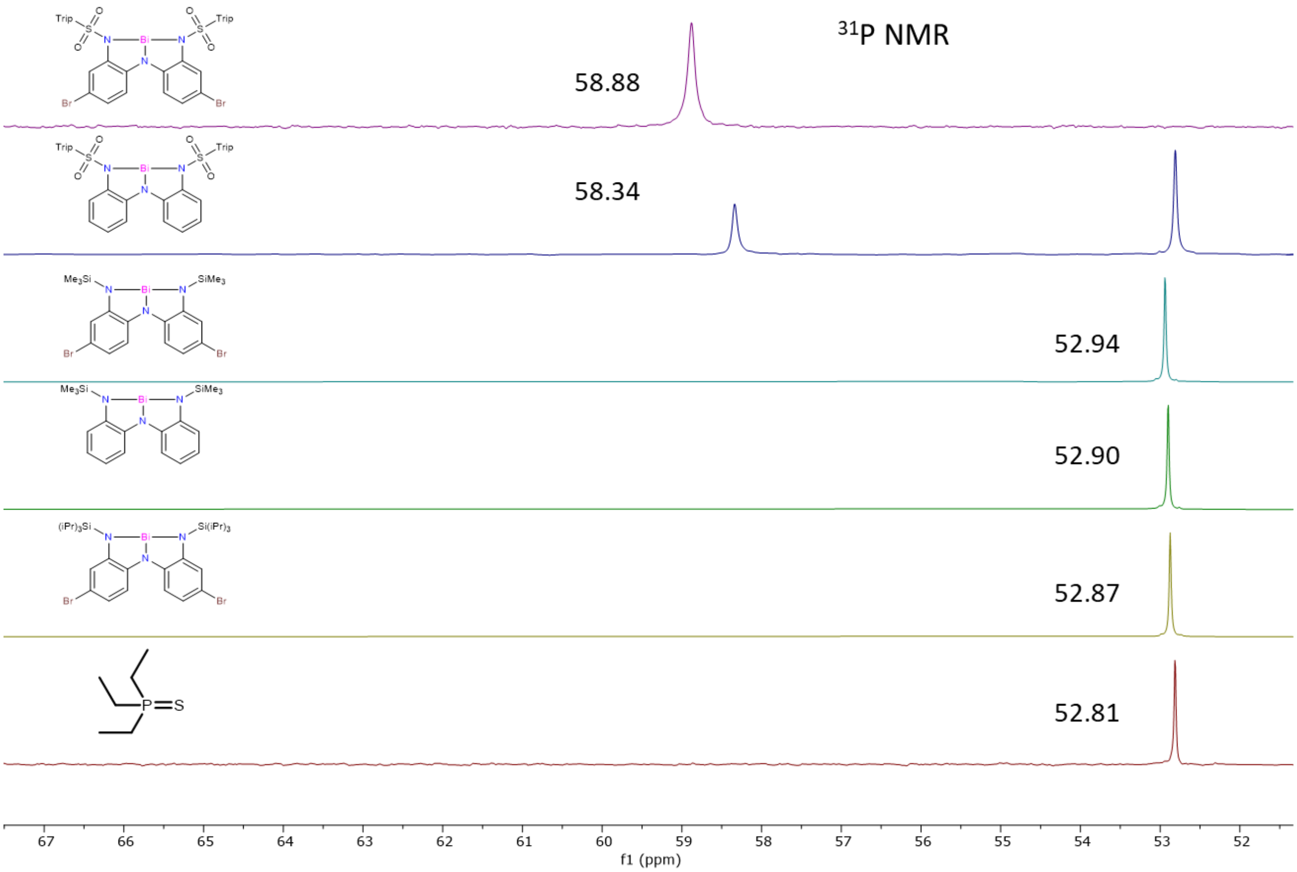


**Figure S3.** Gutmann-Beckett results of Lewis acids combined with $Et_3PS$ in a 5:1 ratio. All spectra are recorded in $C_6H_6$ and referenced to the residual solvent peak in $^1H$ NMR, corrected for $^{31}P$. $^{31}P$ {$^1H$} NMR (121 MHz, $C_6H_6$).

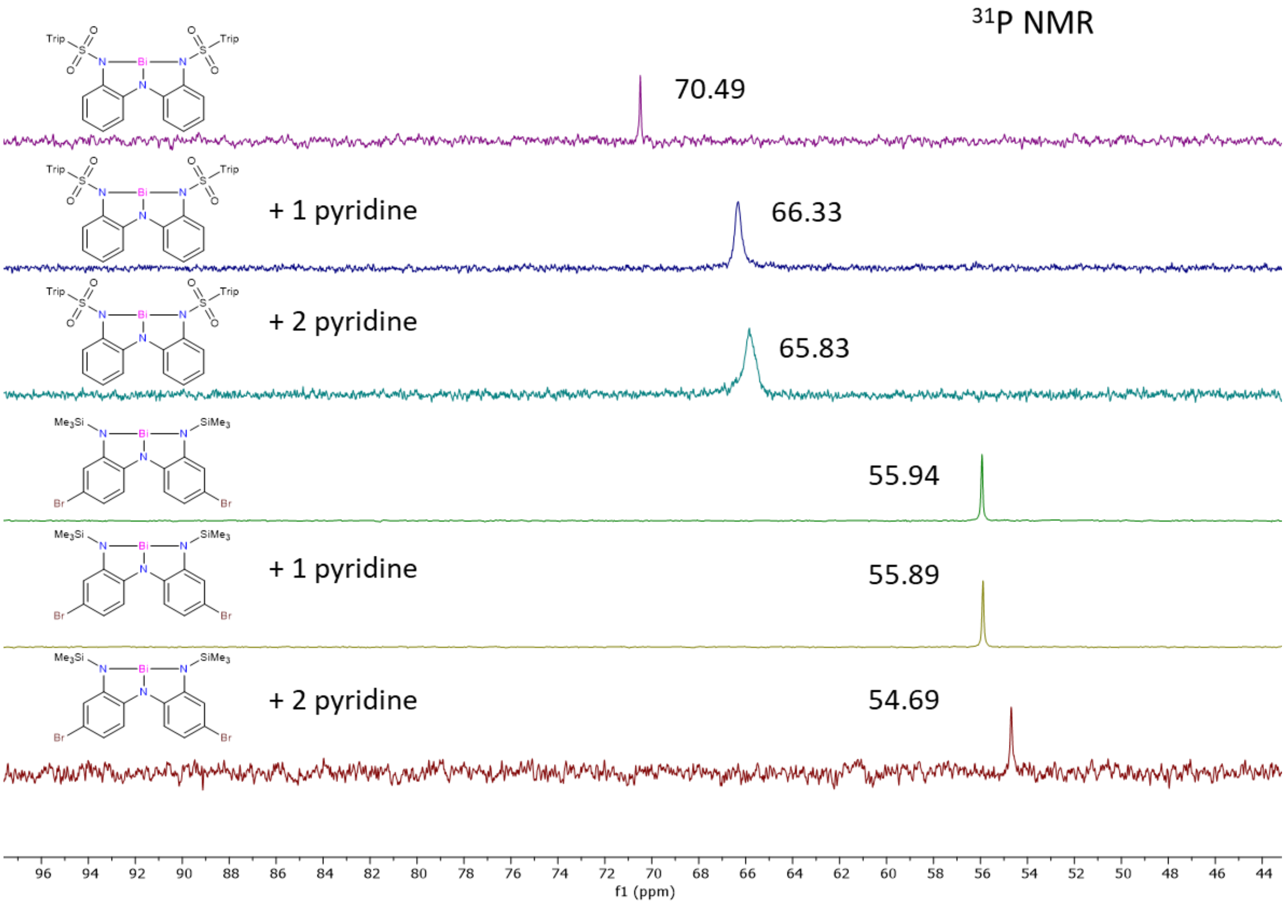


**Figure S4.** Gutmann-Beckett results of Lewis acids combined with $Et_3PO$ in a 5:1 ratio. Results showing change in chemical shift upon addition of 1 and 2 equivalents of pyridine in an attempt to displace the phosphine. All spectra are recorded in $C_6H_6$ and referenced to the residual solvent peak in $^1H$ NMR, corrected for $^{31}P$. $^{31}P$ {$^1H$} NMR (121 MHz, $C_6H_6$).

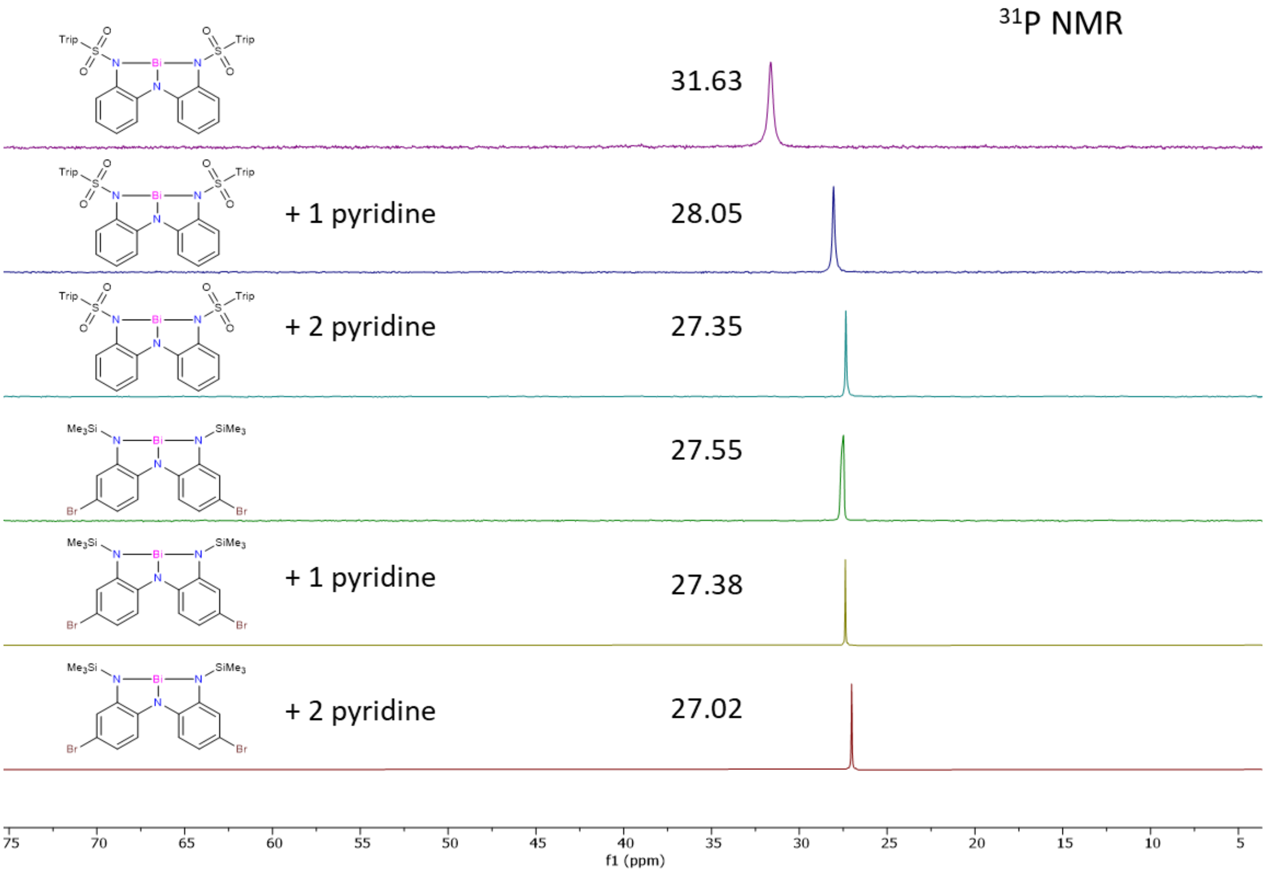


**Figure S5.** Gutmann-Beckett results of Lewis acids combined with $Me_3PS$ in a 5:1 ratio. Results showing change in chemical shift upon addition of 1 and 2 equivalents of pyridine in an attempt to displace the phosphine. All spectra are recorded in $C_6H_6$ and referenced to the residual solvent peak in $^1H$ NMR, corrected for $^{31}P$. $^{31}P$ {$^1H$} NMR (121 MHz, $C_6H_6$).

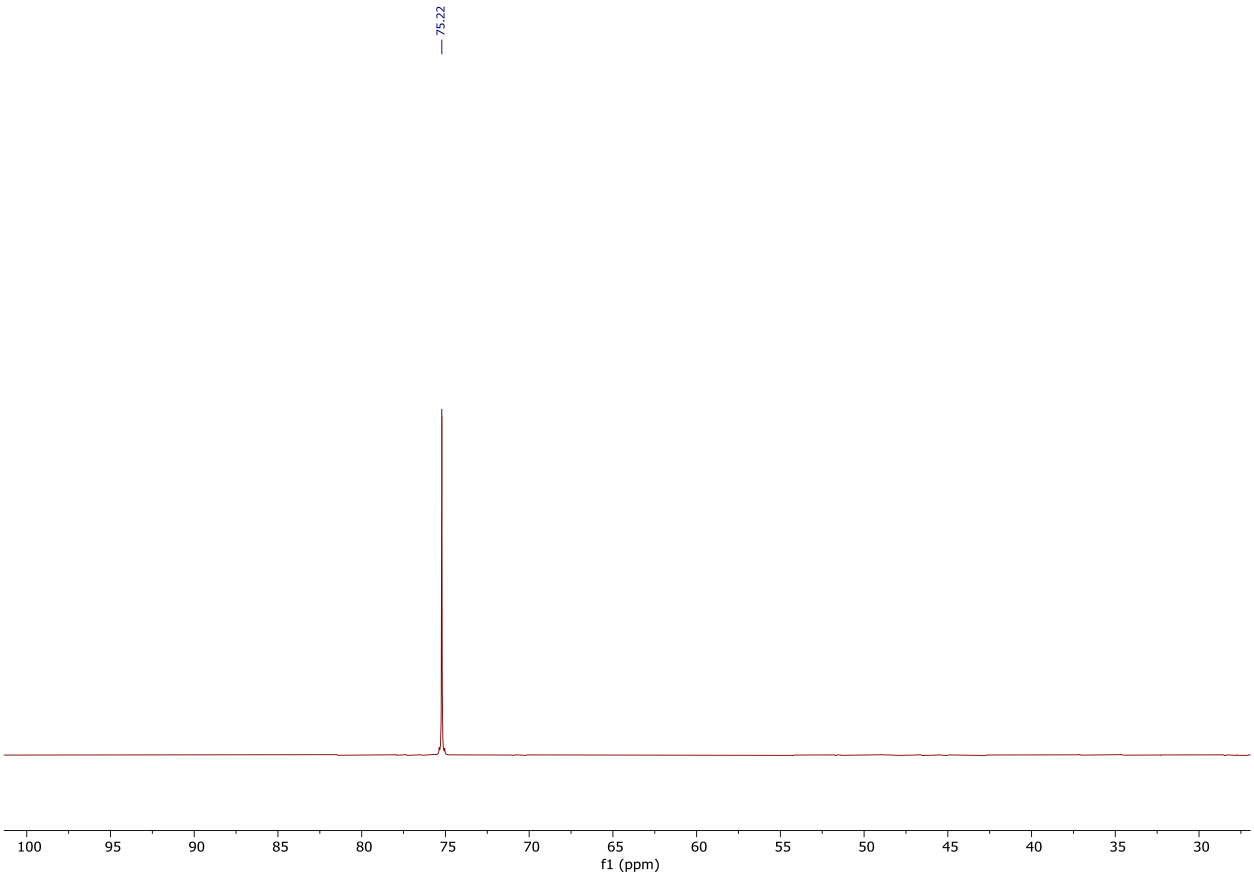


**Figure S6.** Gutmann-Beckett result of $B(C_6F_5)_3$ combined with $Et_3PO$ in a 5:1 ratio. Spectrum is recorded in $C_6H_6$ and referenced to the residual solvent peak in $^1H$ NMR, corrected for $^{31}P$. $^{31}P$ {$^1H$} NMR (121 MHz, $C_6H_6$).

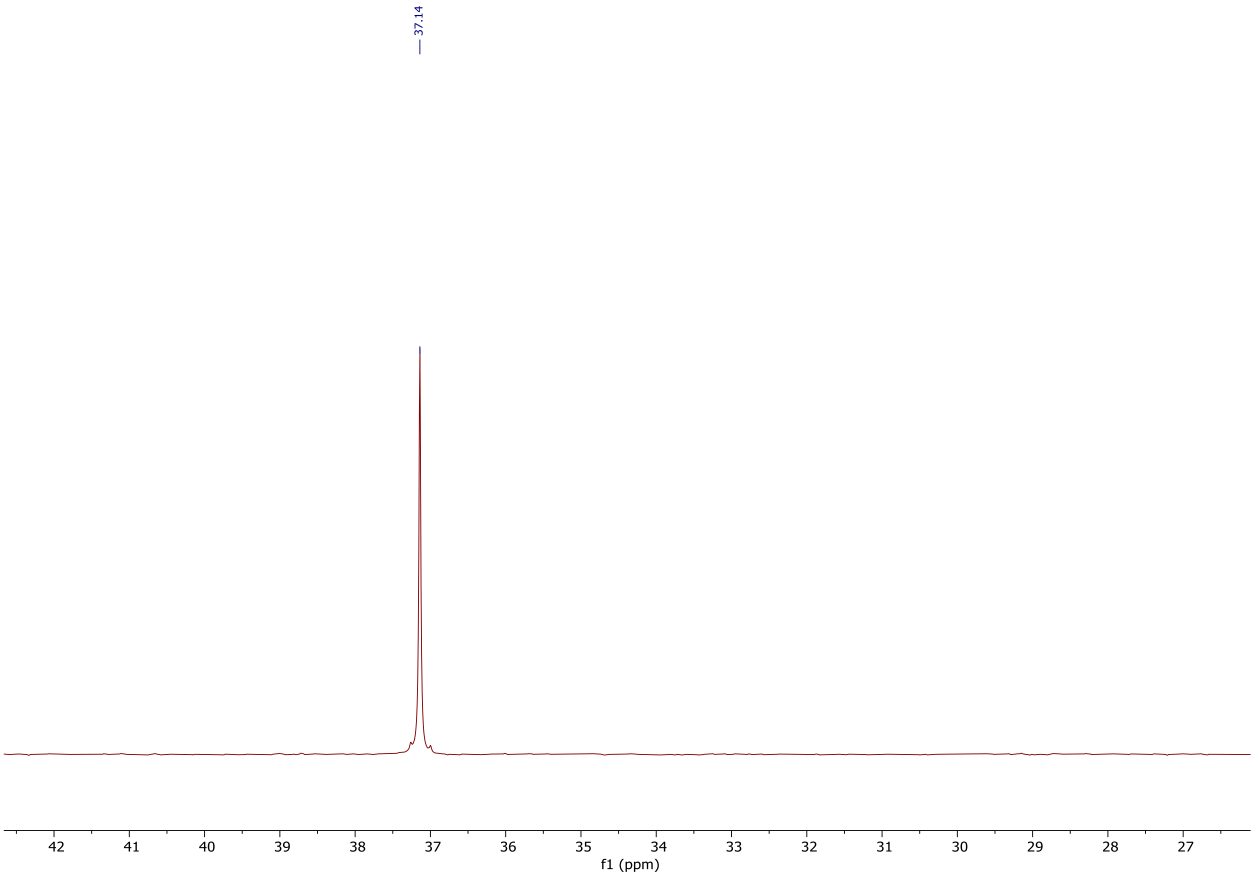


**Figure S7.** Gutmann-Beckett result of $B(C_6F_5)_3$ combined with $Me_3PS$ in a 5:1 ratio. Spectrum is recorded in $C_6H_6$ and referenced to the residual solvent peak in $^1H$ NMR, corrected for $^{31}P$. $^{31}P$ {$^1H$} NMR (121 MHz, $C_6H_6$).

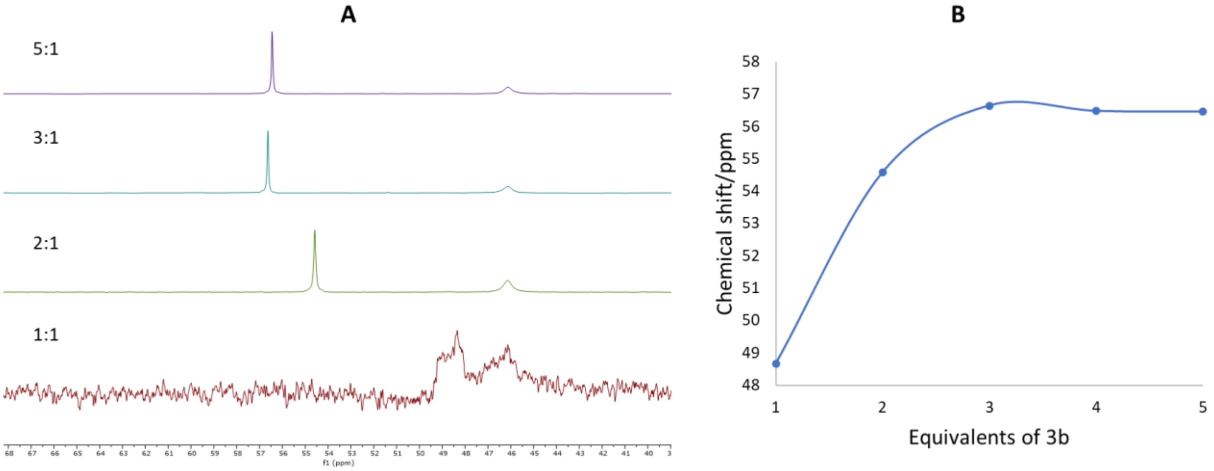


**Figure S8.** Gutmann-Beckett results of **3b** combined with $Et_3PO$ while varying the amount of Lewis acid. All spectra are recorded in toluene and referenced to an internal standard of the phosphine in a sealed capillary. A shows stacked spectra while B is a plot of chemical shifts showing trend. $^{31}P\{^1H\}$ NMR (121 MHz, $C_6H_6$).

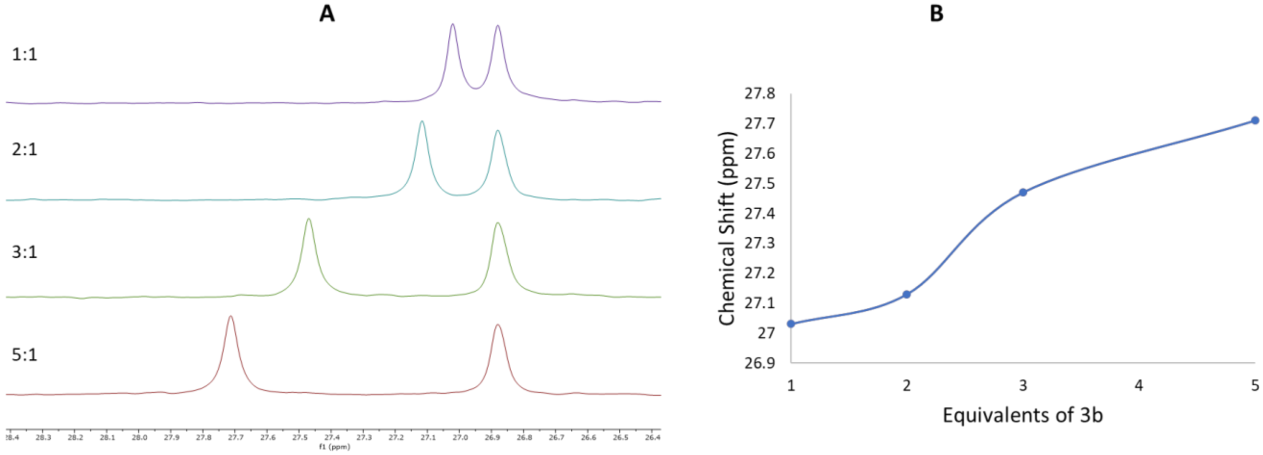


**Figure S9.** Gutmann-Beckett results of **3b** combined with $Me_3PS$ while varying the amount of Lewis acid. All spectra are recorded in toluene and referenced to an internal standard of the phosphine in a sealed capillary. A shows stacked spectra while B is a plot of chemical shifts showing trend. $^{31}P\{^1H\}$ NMR (121 MHz, $C_6H_6$).

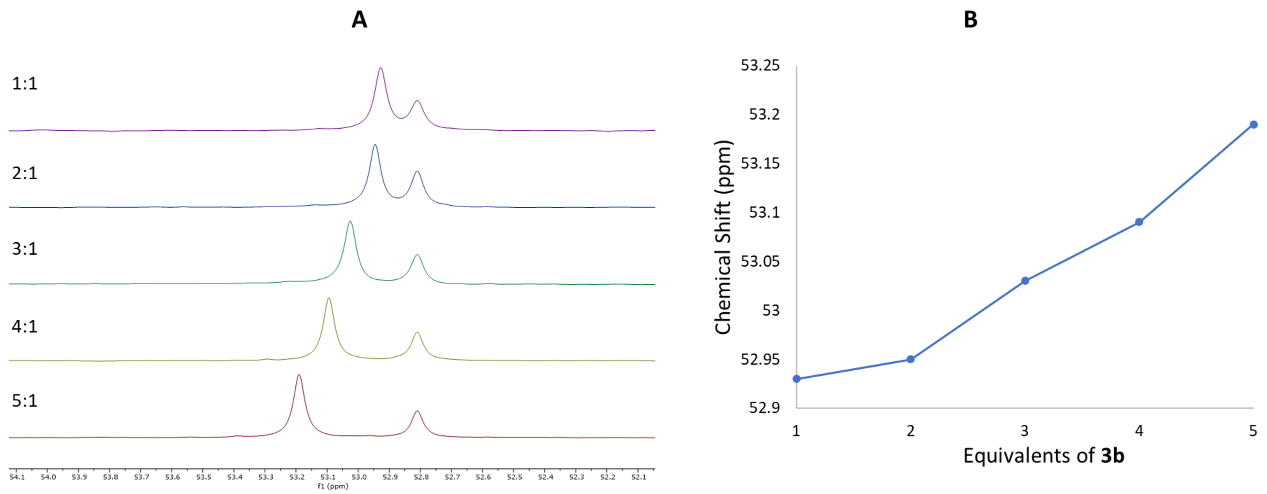


**Figure S10.** Gutmann-Beckett results of **3b** combined with $Et_3PS$ while varying the amount of Lewis acid. All spectra are recorded in toluene and referenced to an internal standard of the phosphine in a sealed capillary. A shows stacked spectra while B is a plot of chemical shifts showing trend. $^{31}P$ {$^{1}H$} NMR (121 MHz, $C_6H_6$).

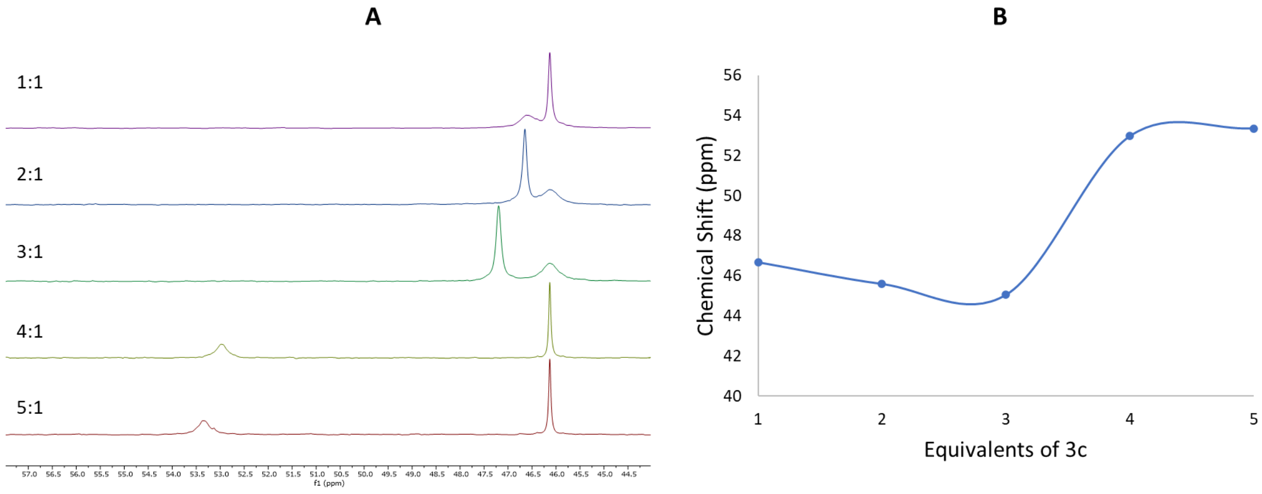


**Figure S11.** Gutmann-Beckett results of **3c** combined with $Et_3PO$ while varying the amount of Lewis acid. All spectra are recorded in toluene and referenced to an internal standard of the phosphine in a sealed capillary. A shows stacked spectra while B is a plot of chemical shifts showing trend. $^{31}P$ {$^{1}H$} NMR (121 MHz, $C_6H_6$).

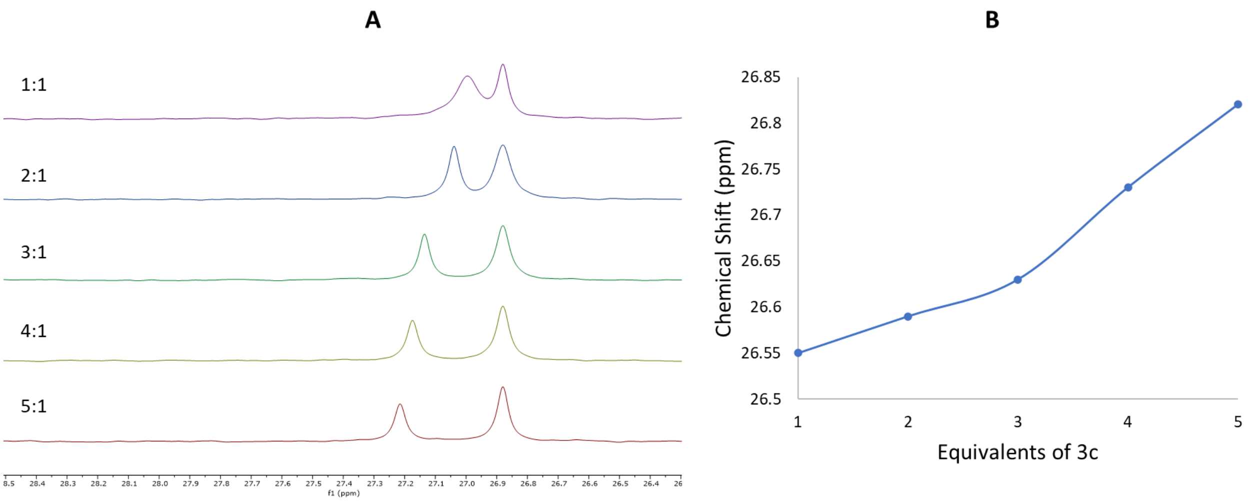


**Figure S12.** Gutmann-Beckett results of **3c** combined with $Me_3PS$ while varying the amount of Lewis acid. All spectra are recorded in toluene and referenced to an internal standard of the phosphine in a sealed capillary. A shows stacked spectra while B is a plot of chemical shifts showing trend. $^{31}P\ \{^1H\}$ NMR (121 MHz, $C_6H_6$).

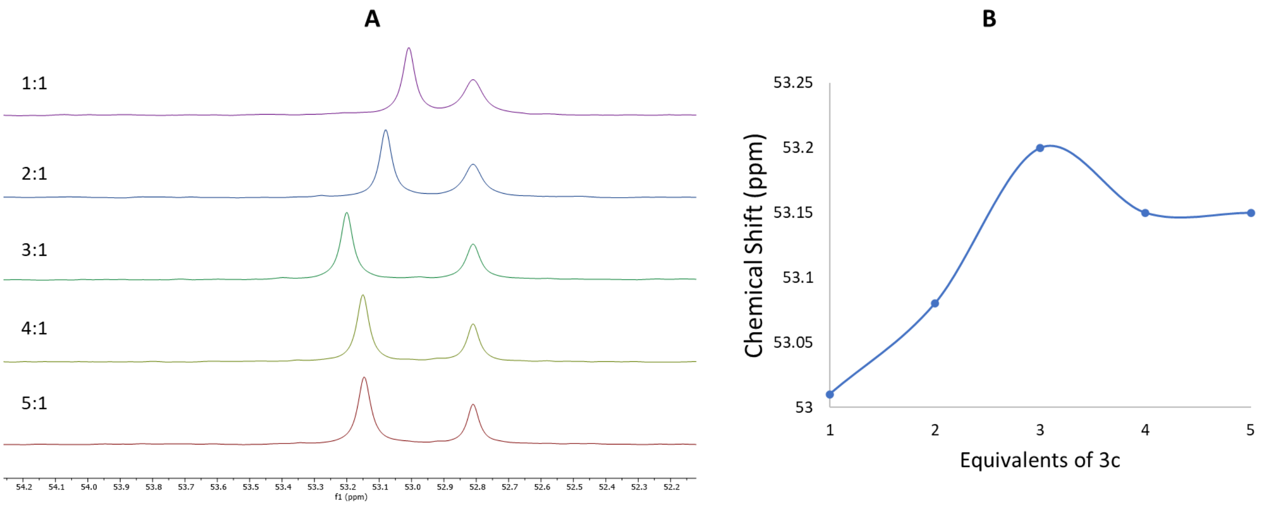


**Figure S13.** Gutmann-Beckett results of **3c** combined with $Et_3PS$ while varying the amount of Lewis acid. All spectra are recorded in toluene and referenced to an internal standard of the phosphine in a sealed capillary. A shows stacked spectra while B is a plot of chemical shifts showing trend. $^{31}P\ \{^1H\}$ NMR (121 MHz, $C_6H_6$).

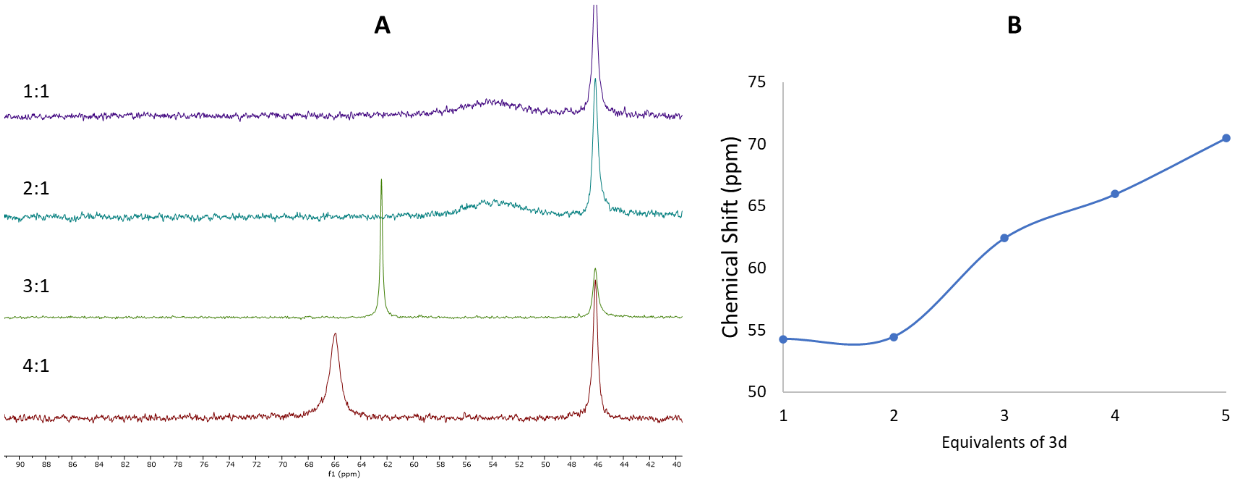


**Figure S14.** Gutmann-Beckett results of **3d** combined with $Et_3PO$ while varying the amount of Lewis acid. All spectra are recorded in toluene and referenced to an internal standard of the phosphine in a sealed capillary. A shows stacked spectra while B is a plot of chemical shifts showing trend. $^{31}P\ \{^{1}H\}$ NMR (121 MHz, $C_6H_6$).

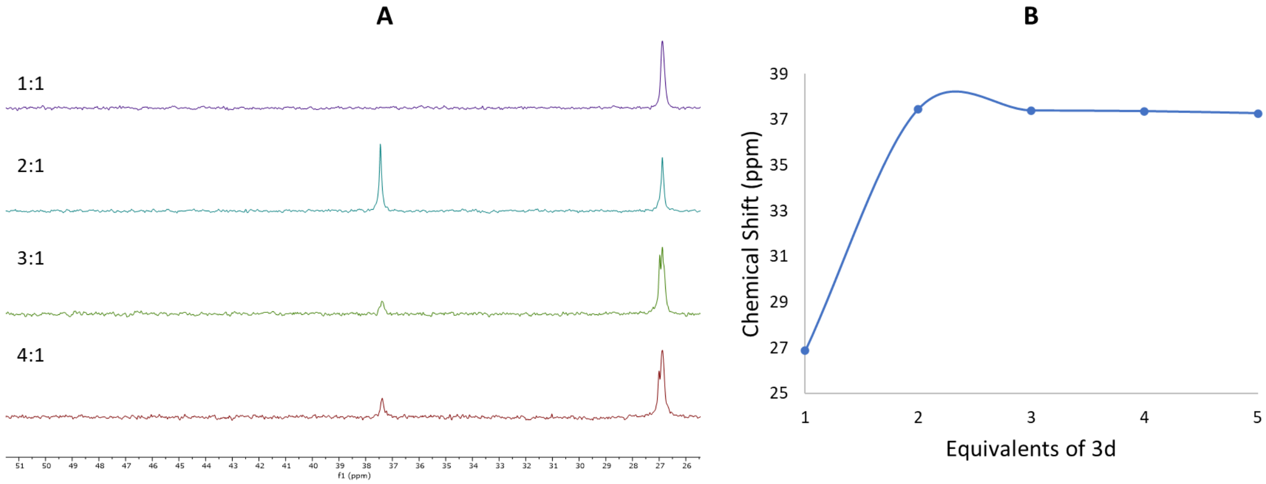


**Figure S15.** Gutmann-Beckett results of **3d** combined with $Me_3PS$ while varying the amount of Lewis acid. All spectra are recorded in toluene and referenced to an internal standard of the phosphine in a sealed capillary. A shows stacked spectra while B is a plot of chemical shifts showing trend. $^{31}P\ \{^{1}H\}$ NMR (121 MHz, $C_6H_6$).

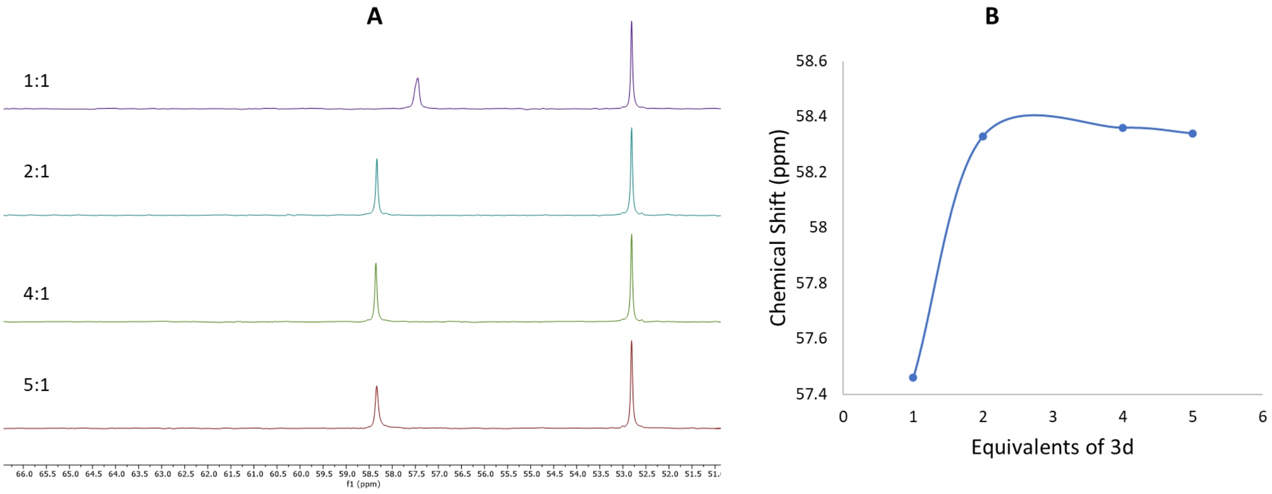


**Figure S16.** Gutmann-Beckett results of **3d** combined with $Et_3PS$ while varying the amount of Lewis acid. All spectra are recorded in toluene and referenced to an internal standard of the phosphine in a sealed capillary. A shows stacked spectra while B is a plot of chemical shifts showing trend. $^{31}P$ {$^{1}H$} NMR (121 MHz, $C_6H_6$).

## DOSY NMR Data

**Table S1.** Monomer-dimer solution phase analysis by DOSY NMR

| **Compound (conc)** | **Diffusion coefficient (D, $m^2s^{-1}$)** | **Molecular weight (g $mol^{-1}$)$^a$** | **Theoretical molecular weight (g $mol^{-1}$)** |
|---|---|---|---|
| **3b** (0.01 M) | $6.01 \times 10^{-10}$ | 712 | 707.37 |
| **3c** (0.01 M) | $5.85 \times 10^{-10}$ | 750 | 875.69 |
| **3d** (0.01 M) | $5.84 \times 10^{-10}$ | 752 | 769.69 |
| **3d** (0.05 M) | $5.16 \times 10^{-10}$ | 945 | 769.69 |

$^a$Conversions to molecular weight were done using Grubbs' DOSY calibration curve.

SIMFIT RESULTS
==============
AREA fit : Diffusion : Variable Gradient :
I=I[0]*exp(-D*SQR(2*PI*gamma*Gi*LD)*(BD-LD/3)*1e4)
16 points for Integral 1, Integral Region from 7.694 to 7.373 ppm
Converged after 40 iterations!
Results Comp. 1
I[0] = 1.002e+00
Diff Con. = 6.013e-10 m2/s
Gamma = 4.258e+03 Hz/G
Little Delta = 2.200m
Big Delta = 100.000m
RSS = 2.155e-05
SD = 1.160e-03

| Point | Gradient | Expt | Calc | Difference |
|---|---|---|---|---|
| 1 | 9.630e-01 | 1.000e+00 | 9.999e-01 | -1.019e-04 |
| 2 | 3.948e+00 | 9.678e-01 | 9.700e-01 | 2.264e-03 |
| 3 | 6.934e+00 | 9.076e-01 | 9.070e-01 | -5.339e-04 |
| 4 | 9.919e+00 | 8.178e-01 | 8.174e-01 | -3.437e-04 |
| 5 | 1.290e+01 | 7.122e-01 | 7.100e-01 | -2.128e-03 |
| 6 | 1.589e+01 | 5.944e-01 | 5.944e-01 | 5.803e-05 |
| 7 | 1.888e+01 | 4.789e-01 | 4.796e-01 | 7.559e-04 |
| 8 | 2.186e+01 | 3.742e-01 | 3.730e-01 | -1.158e-03 |
| 9 | 2.484e+01 | 2.795e-01 | 2.796e-01 | 1.010e-04 |
| 10 | 2.783e+01 | 2.029e-01 | 2.020e-01 | -8.907e-04 |
| 11 | 3.082e+01 | 1.402e-01 | 1.407e-01 | 4.983e-04 |
| 12 | 3.380e+01 | 9.279e-02 | 9.441e-02 | 1.615e-03 |
| 13 | 3.679e+01 | 5.973e-02 | 6.106e-02 | 1.331e-03 |
| 14 | 3.977e+01 | 3.637e-02 | 3.807e-02 | 1.697e-03 |
| 15 | 4.276e+01 | 2.216e-02 | 2.288e-02 | 7.135e-04 |
| 16 | 4.574e+01 | 1.411e-02 | 1.325e-02 | -8.625e-04 |

==========================================================

**Figure S17.** DOSY report for **3b** (0.01 M).

SIMFIT RESULTS
==============
AREA fit : Diffusion : Variable Gradient :
I=I[0]*exp(-D*SQR(2*PI*gamma*Gi*LD)*(BD-LD/3)*1e4)
16 points for Integral 1, Integral Region from 7.873 to 7.774 ppm
Converged after 35 iterations!
Results Comp. 1
I[0] = 1.013e+00
Diff Con. = 7.332e-10 m2/s
Gamma = 4.258e+03 Hz/G
Little Delta = 2.200m
Big Delta = 100.000m
RSS = 1.261e-03
SD = 8.876e-03

| Point | Gradient | Expt | Calc | Difference |
|---|---|---|---|---|
| 1 | 9.630e-01 | 1.000e+00 | 1.011e+00 | 1.060e-02 |
| 2 | 3.467e+00 | 9.761e-01 | 9.827e-01 | 6.682e-03 |
| 3 | 5.971e+00 | 9.309e-01 | 9.259e-01 | -5.049e-03 |
| 4 | 8.474e+00 | 8.598e-01 | 8.452e-01 | -1.453e-02 |
| 5 | 1.098e+01 | 7.517e-01 | 7.476e-01 | -4.109e-03 |
| 6 | 1.348e+01 | 6.486e-01 | 6.406e-01 | -7.969e-03 |
| 7 | 1.599e+01 | 5.292e-01 | 5.319e-01 | 2.702e-03 |
| 8 | 1.849e+01 | 4.221e-01 | 4.278e-01 | 5.751e-03 |
| 9 | 2.099e+01 | 3.124e-01 | 3.335e-01 | 2.109e-02 |
| 10 | 2.350e+01 | 2.528e-01 | 2.518e-01 | -9.863e-04 |
| 11 | 2.600e+01 | 1.935e-01 | 1.842e-01 | -9.295e-03 |
| 12 | 2.850e+01 | 1.385e-01 | 1.306e-01 | -7.907e-03 |
| 13 | 3.101e+01 | 9.770e-02 | 8.970e-02 | -8.005e-03 |
| 14 | 3.351e+01 | 5.435e-02 | 5.970e-02 | 5.349e-03 |
| 15 | 3.602e+01 | 3.505e-02 | 3.849e-02 | 3.438e-03 |
| 16 | 3.852e+01 | 1.720e-02 | 2.404e-02 | 6.848e-03 |

==============================================================

**Figure S18.** DOSY report for **3c** (0.01 M).

SIMFIT RESULTS

==============

AREA fit : Diffusion : Variable Gradient :

I=I[0]*exp(-D*SQR(2*PI*gamma*Gi*LD)*(BD-LD/3)*1e4)

16 points for Integral 1, Integral Region from 7.680 to 7.595 ppm

Converged after 38 iterations!

Results Comp. 1

I[0] = 9.826e-01

Diff Con. = 5.838e-10 m2/s

Gamma = 4.258e+03 Hz/G

Little Delta = 1.800m

Big Delta = 100.000m

RSS = 3.877e-03

SD = 1.557e-02

| Point | Gradient | Expt | Calc | Difference |
|---|---|---|---|---|
| 1 | 9.630e-01 | 1.000e+00 | 9.813e-01 | -1.865e-02 |
| 2 | 3.788e+00 | 9.680e-01 | 9.638e-01 | -4.185e-03 |
| 3 | 6.613e+00 | 9.333e-01 | 9.264e-01 | -6.836e-03 |
| 4 | 9.437e+00 | 8.942e-01 | 8.716e-01 | -2.254e-02 |
| 5 | 1.226e+01 | 7.752e-01 | 8.026e-01 | 2.745e-02 |
| 6 | 1.509e+01 | 7.040e-01 | 7.234e-01 | 1.941e-02 |
| 7 | 1.791e+01 | 6.119e-01 | 6.381e-01 | 2.618e-02 |
| 8 | 2.074e+01 | 5.463e-01 | 5.509e-01 | 4.645e-03 |
| 9 | 2.356e+01 | 4.576e-01 | 4.656e-01 | 7.957e-03 |
| 10 | 2.639e+01 | 3.920e-01 | 3.851e-01 | -6.896e-03 |
| 11 | 2.921e+01 | 3.085e-01 | 3.117e-01 | 3.251e-03 |
| 12 | 3.204e+01 | 2.552e-01 | 2.470e-01 | -8.191e-03 |
| 13 | 3.486e+01 | 2.131e-01 | 1.915e-01 | -2.156e-02 |
| 14 | 3.769e+01 | 1.465e-01 | 1.454e-01 | -1.091e-03 |
| 15 | 4.051e+01 | 1.285e-01 | 1.080e-01 | -2.048e-02 |
| 16 | 4.333e+01 | 8.533e-02 | 7.852e-02 | -6.807e-03 |

==========================================================

**Figure S19.** DOSY report for **3d** (0.01 M).

SIMFIT RESULTS
==============
AREA fit : Diffusion : Variable Gradient :
I=I[0]*exp(-D*SQR(2*PI*gamma*Gi*LD)*(BD-LD/3)*1e4)
16 points for Integral 1, Integral Region from 7.713 to 7.538 ppm
Converged after 36 iterations!
Results Comp. 1
I[0] = 1.007e+00
Diff Con. = 5.162e-10 m2/s
Gamma = 4.258e+03 Hz/G
Little Delta = 2.200m
Big Delta = 100.000m
RSS = 5.618e-04
SD = 5.925e-03

| Point | Gradient | Expt | Calc | Difference |
|---|---|---|---|---|
| 1 | 9.630e-01 | 1.000e+00 | 1.005e+00 | 5.305e-03 |
| 2 | 3.788e+00 | 9.801e-01 | 9.816e-01 | 1.506e-03 |
| 3 | 6.613e+00 | 9.294e-01 | 9.318e-01 | 2.394e-03 |
| 4 | 9.437e+00 | 8.618e-01 | 8.597e-01 | -2.049e-03 |
| 5 | 1.226e+01 | 7.714e-01 | 7.711e-01 | -3.440e-04 |
| 6 | 1.509e+01 | 6.752e-01 | 6.723e-01 | -2.894e-03 |
| 7 | 1.791e+01 | 5.816e-01 | 5.698e-01 | -1.184e-02 |
| 8 | 2.074e+01 | 4.736e-01 | 4.694e-01 | -4.208e-03 |
| 9 | 2.356e+01 | 3.782e-01 | 3.759e-01 | -2.283e-03 |
| 10 | 2.639e+01 | 2.880e-01 | 2.926e-01 | 4.646e-03 |
| 11 | 2.921e+01 | 2.191e-01 | 2.214e-01 | 2.354e-03 |
| 12 | 3.204e+01 | 1.470e-01 | 1.629e-01 | 1.587e-02 |
| 13 | 3.486e+01 | 1.124e-01 | 1.165e-01 | 4.034e-03 |
| 14 | 3.769e+01 | 8.764e-02 | 8.096e-02 | -6.678e-03 |
| 15 | 4.051e+01 | 5.191e-02 | 5.470e-02 | 2.794e-03 |
| 16 | 4.333e+01 | 3.738e-02 | 3.593e-02 | -1.456e-03 |

==========================================================

**Figure S20.** DOSY report for **3d** (0.05 M).

## Calculated Fluoride Ion affinities

**Table S2**. Calculated FIAs

| Compound | FIA (kJ/mol) |
|---|---|
| **3a** | 304 |
| **3b** | 336 |
| **3c** | 327 |
| **3d** | 378 |
| **3e** | 395 |
| $BiCl_3$ | 333 |
| $BPh_3$ | 324 |
| $B(C_6F_5)_3$ | 440 |

**Table S3**. Reaction Energies of Various Compounds with 1 and 2 Equivalents of $Me_3PO$ and $Me_3PS$, all values are given in kJ/mol

| | **$BiCl_3$** | **$BPh_3$** | **BCF** | **3a** | **3b** | **3c** | **3d** | **3e** |
|---|---|---|---|---|---|---|---|---|
| **$Me_3PO$** | -22.0 | -71.6 | -123.8 | -84.1 | -90.4 | -62.8 | -111.2 | -118.6 |
| **$\Delta E_{int}$ $Me_3PO$** | -23.4 | -188.2 | -246.6 | -93.8 | -100.6 | -100.3 | -133.5 | -158.4 |
| **$Me_3PS$** | -17.2 | -36.4 | -50.2 | -74.2 | -78.9 | -38.1 | -88.4 | -103 |
| **$\Delta E_{int}$ $Me_3PS$** | -18.3 | -131.7 | -171.5 | -79.9 | -85.4 | -82 | -114.7 | -124.2 |
| **2 $Me_3PO$** | | | | -183.1 | -193.8 | -105.8 | -255.1 | -205.6 |
| **2 $Me_3PS$** | | | | -143.5 | -159.6 | -94.3 | -176.9 | -195.7 |

# Percent Buried Volume Calculations

**Table S4.** Percent buried volume calculations for **3b-d**.

| Complex | $V_{buried}$ | |
|---|---|---|
| **3b** | 55.1% | 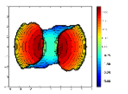 |
| **3c** | 70.2% | 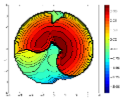 |
| **3d** (Anti conformation) | 77.3% | 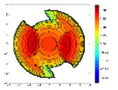 |
| **3d** (Syn conformation | 61.7% | 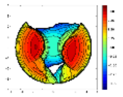 |

## Computational Data:

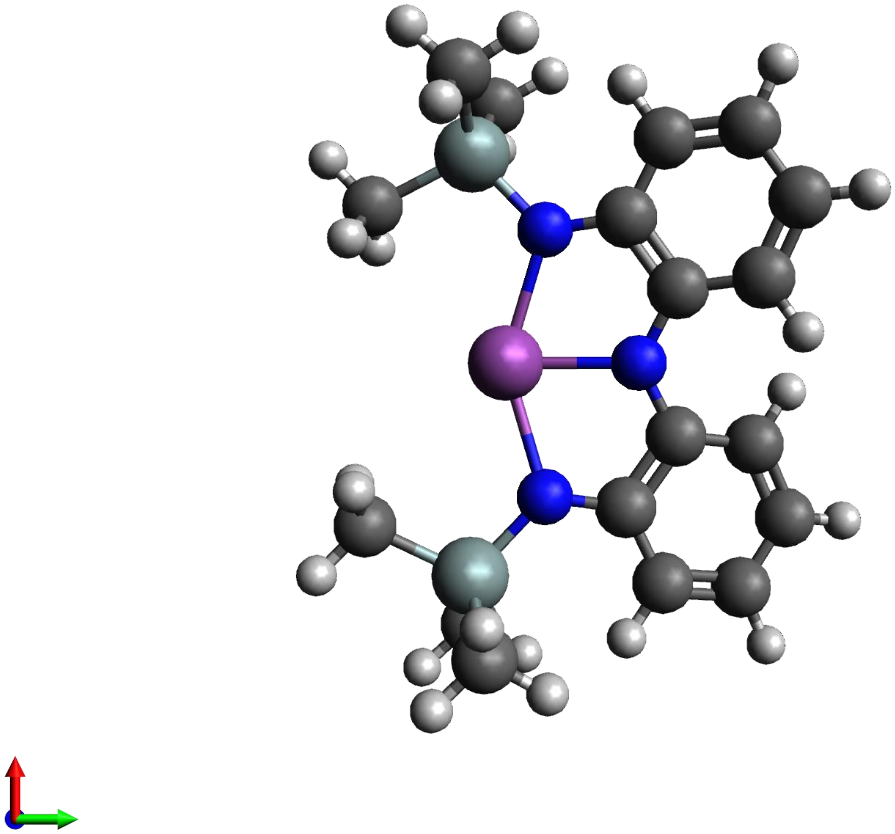

**Figure S21.** Optimized Structure of **3a** at the PBE1PBE/def2-TZVP level.

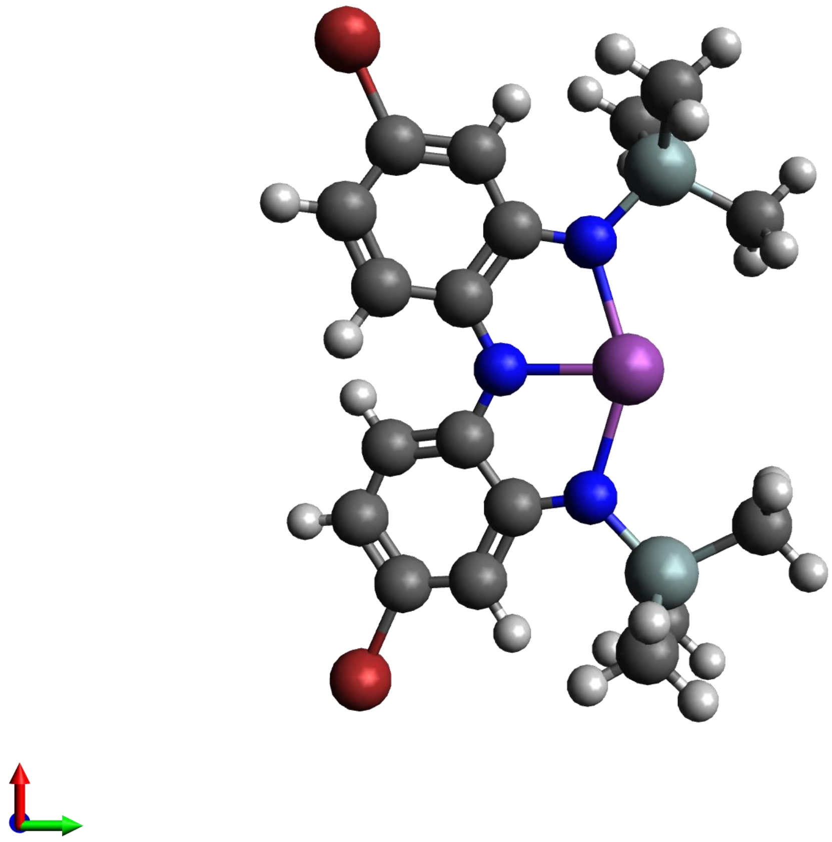

**Figure S22.** Optimized Structure of **3b** at the PBE1PBE/def2-TZVP level.

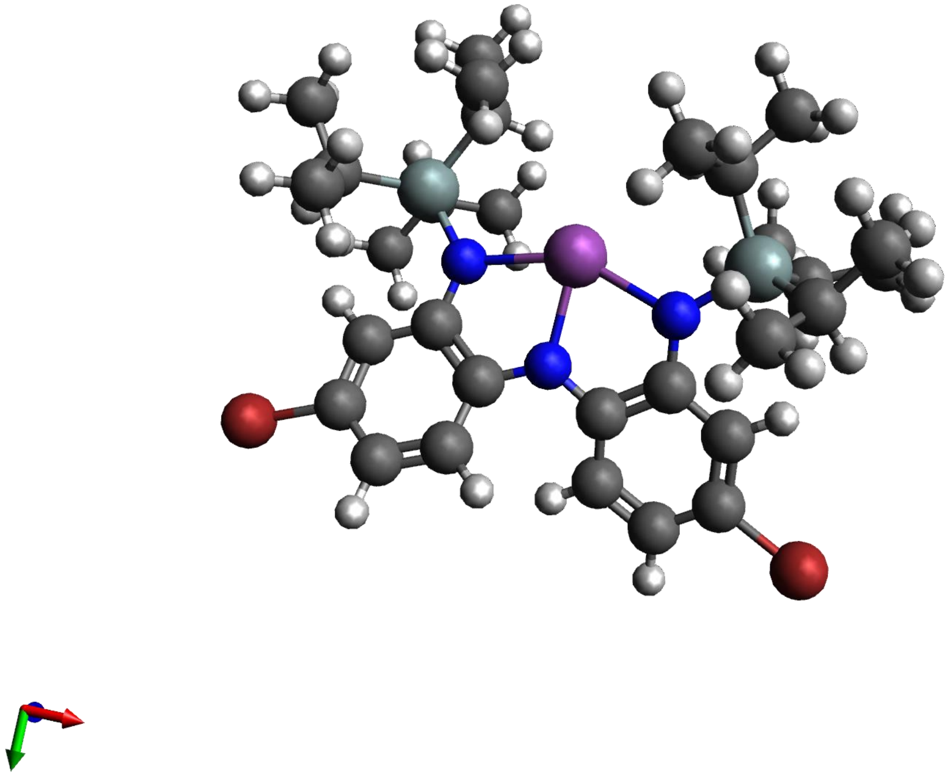

**Figure S23.** Optimized Structure of **3c** at the PBE1PBE/def2-TZVP level.

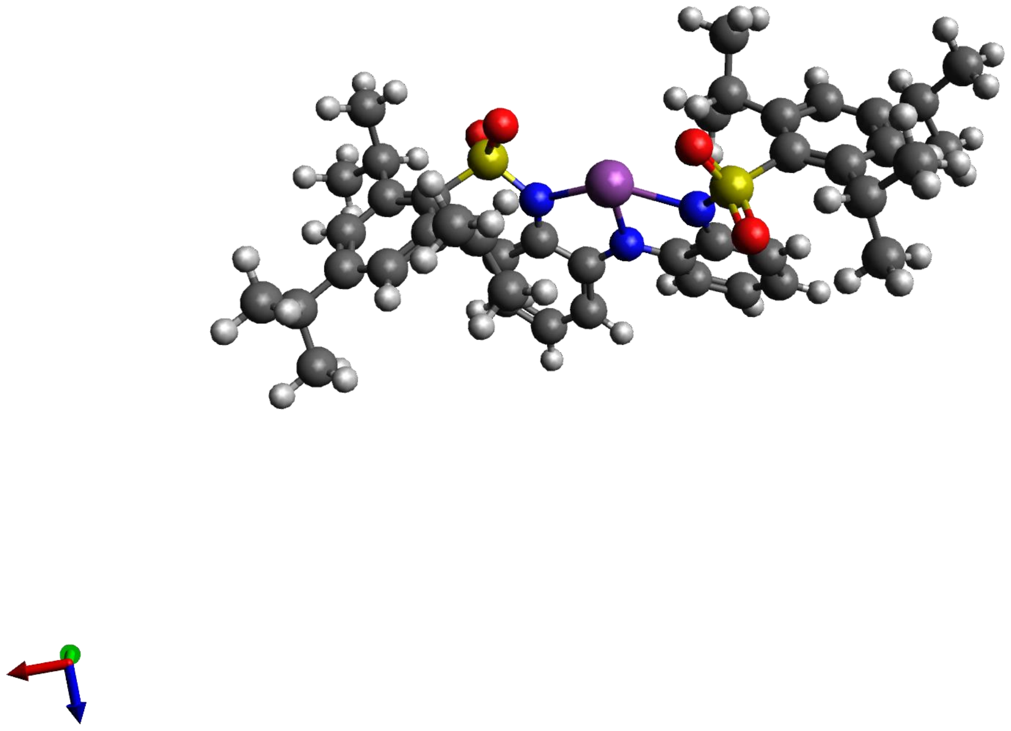

**Figure S24.** Optimized Structure of **3d** at the PBE1PBE/def2-TZVP level.

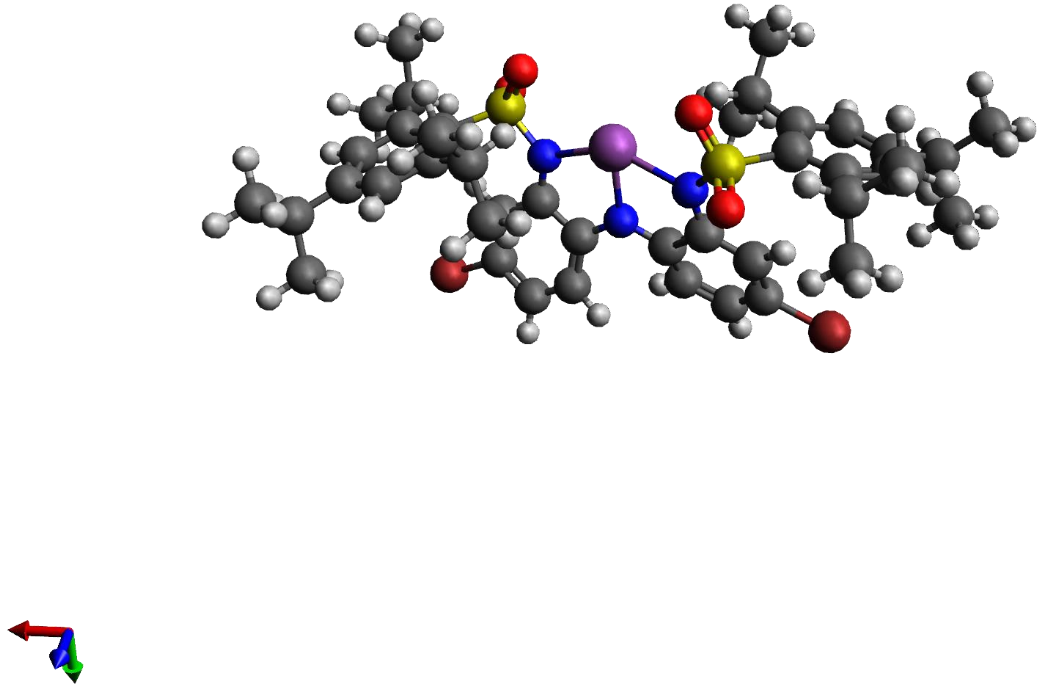

**Figure S25.** Optimized Structure of **3e** at the PBE1PBE/def2-TZVP level.

**Reference for Gaussian**: Gaussian 16, Revision A.02, M. J. Frisch, G. W. Trucks, H. B. Schlegel, G. E. Scuseria, M. A. Robb, J. R. Cheeseman, G. Scalmani, V. Barone, G. A. Petersson, H. Nakatsuji, X. Li, M. Caricato, A. Marenich, J. Bloino, B. G. Janesko, R. Gomperts, B. Mennucci, H. P. Hratchian, J. V. Ortiz, A. F. Izmaylov, J. L. Sonnenberg, D. Williams-Young, F. Ding, F. Lipparini, F. Egidi, J. Goings, B. Peng, A. Petrone, T. Henderson, D. Ranasinghe, V. G. Zakrzewski, J. Gao, N. Rega, G. Zheng, W. Liang, M. Hada, M. Ehara, K. Toyota, R. Fukuda, J. Hasegawa, M. Ishida, T. Nakajima, Y. Honda, O. Kitao, H. Nakai, T. Vreven, K. Throssell, J. A. Montgomery, Jr., J. E. Peralta, F. Ogliaro, M. Bearpark, J. J. Heyd, E. Brothers, K. N. Kudin, V. N. Staroverov, T. Keith, R. Kobayashi, J. Normand, K. Raghavachari, A. Rendell, J. C. Burant, S. S. Iyengar, J. Tomasi, M. Cossi, J. M. Millam, M. Klene, C. Adamo, R. Cammi, J. W. Ochterski, R. L. Martin, K. Morokuma, O. Farkas, J. B. Foresman, and D. J. Fox, Gaussian, Inc., Wallingford CT, 2016.

## Characterization Data:

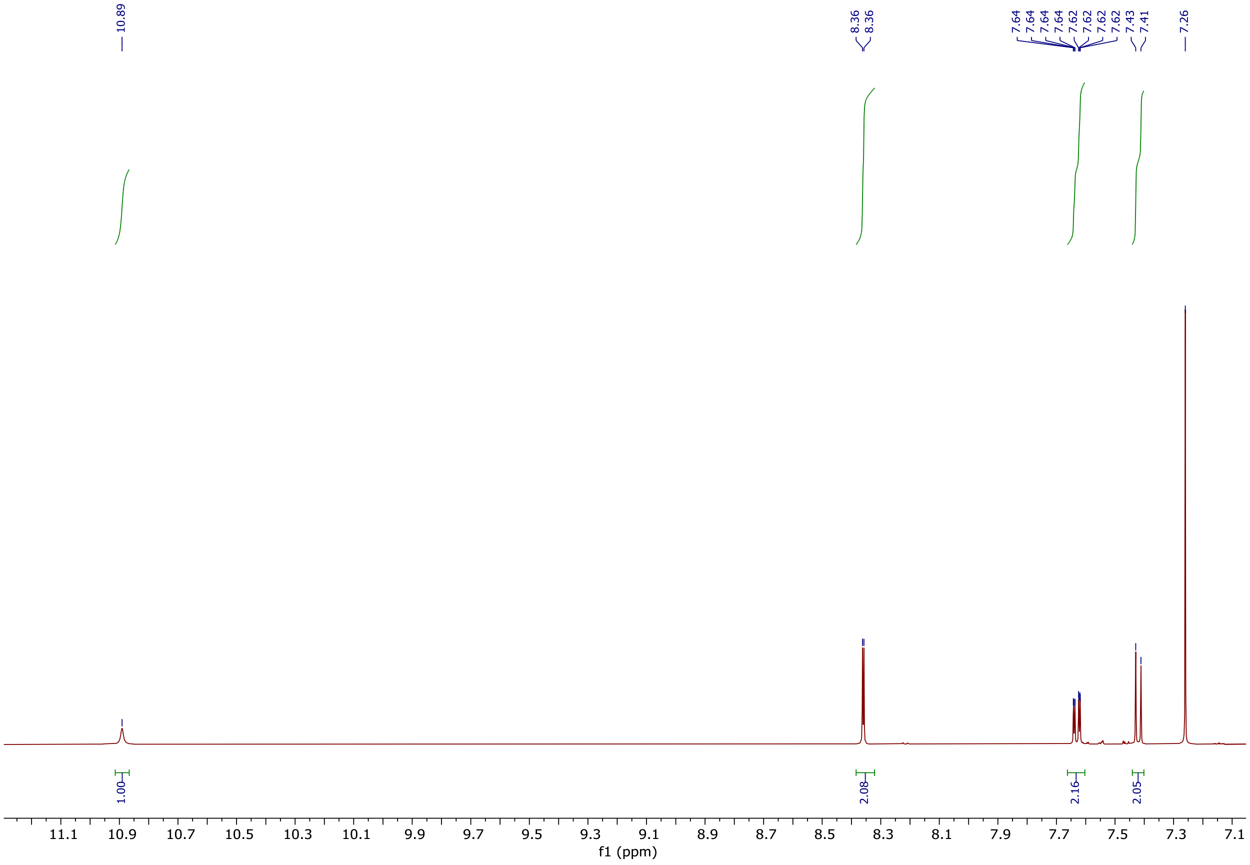


**Figure S26.** $^{1}H$-NMR (500 MHz, $CDCl_3$) of **L1**

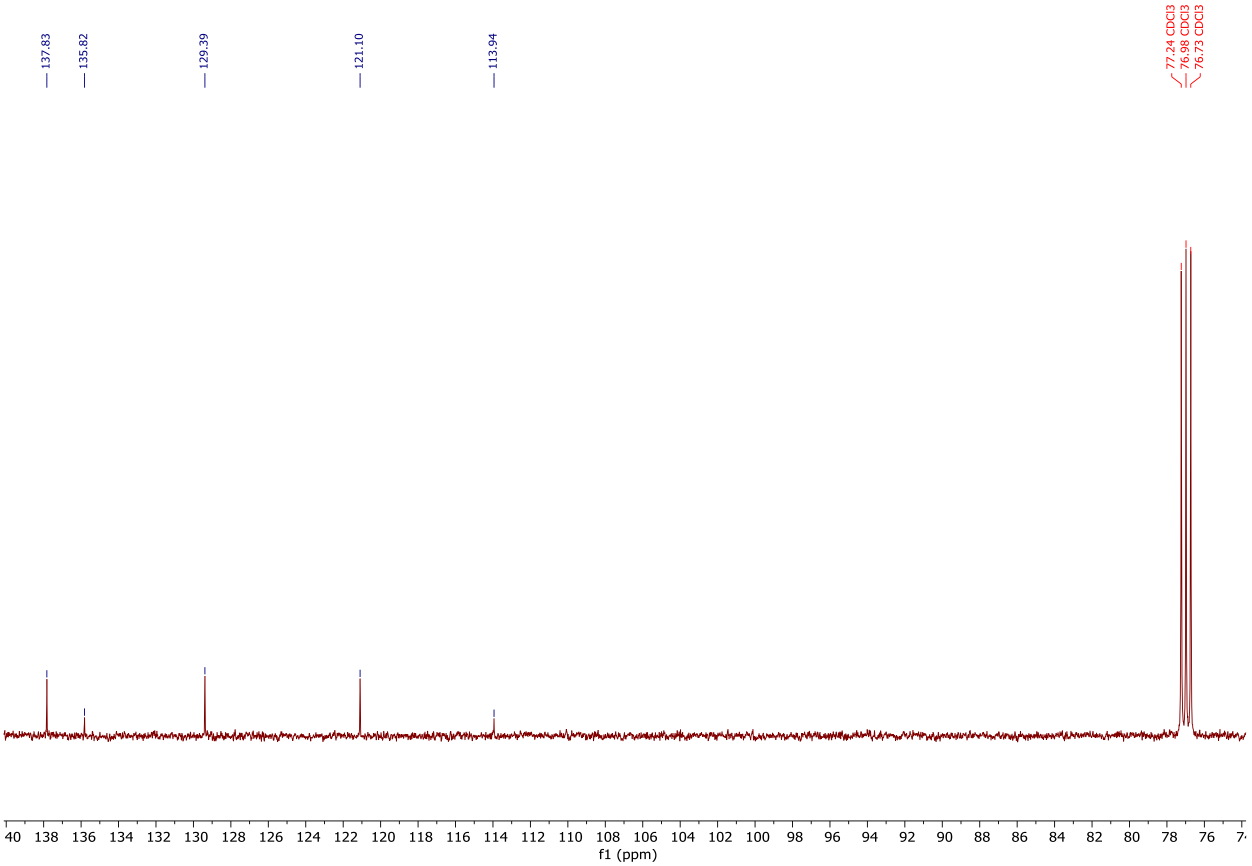


**Figure S27.** $^{13}C$ {$^{1}H$} NMR (126 MHz, $CDCl_3$) of **L1**

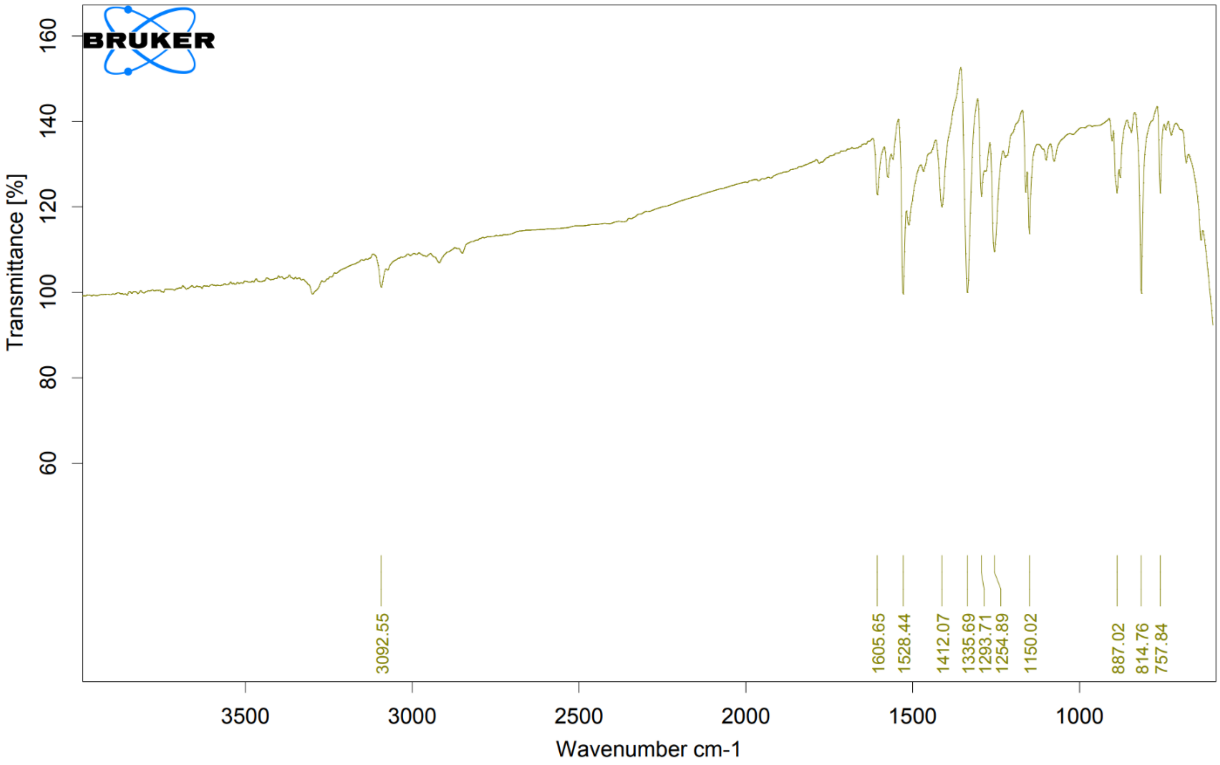


**Figure S28.** Infrared spectrum of **L1**

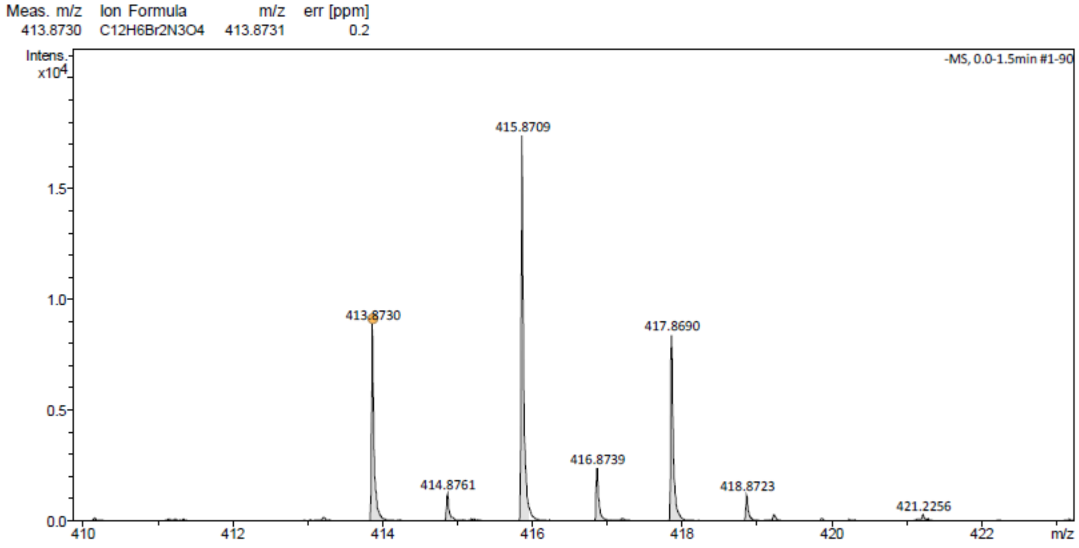


**Figure S29.** Mass spectrum of **L1**

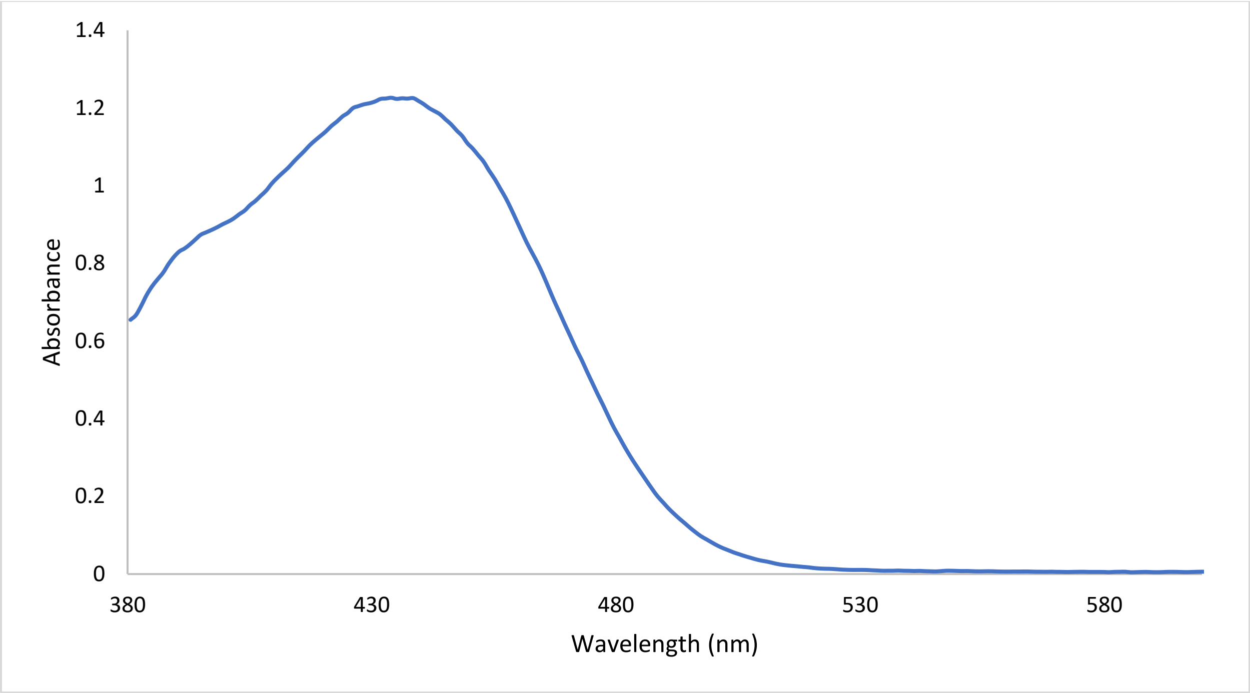


**Figure S30.** UV-Vis absorption spectrum of **L1**

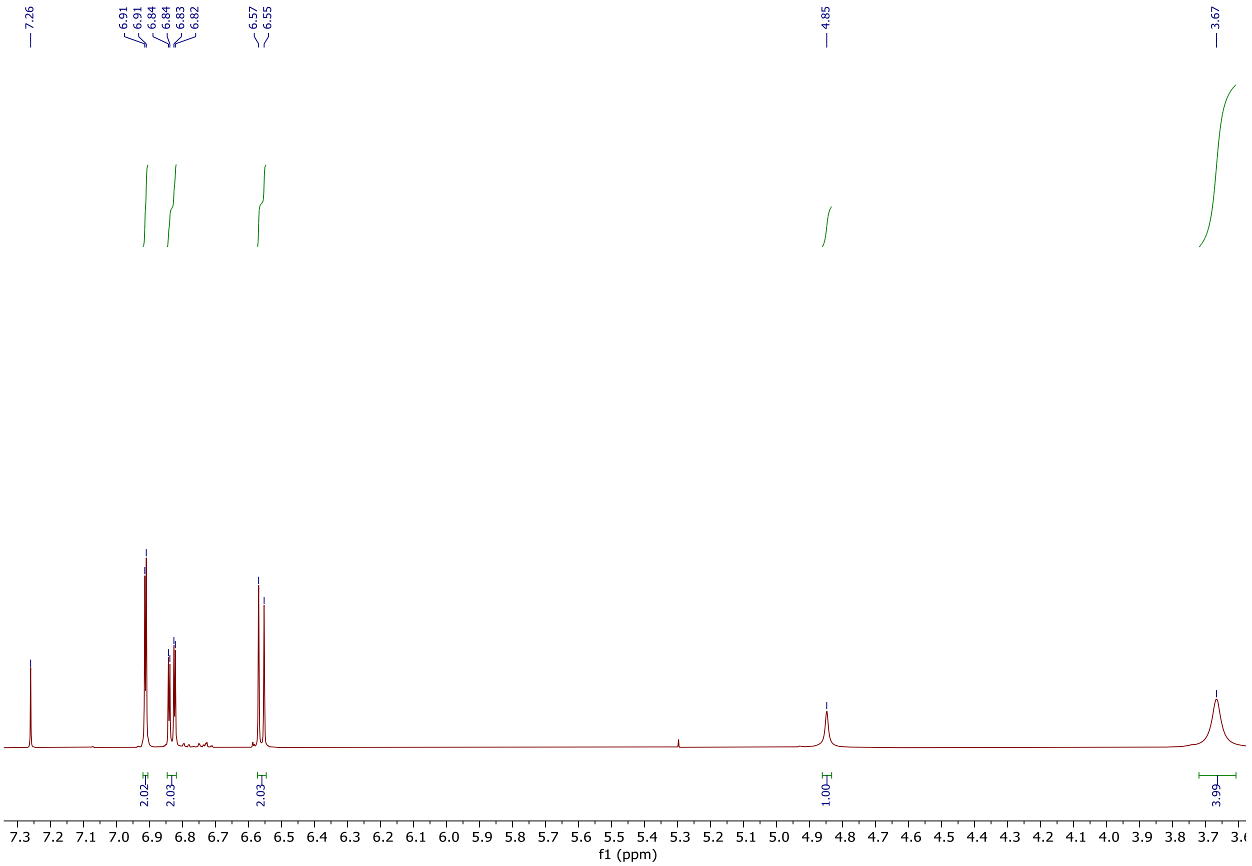


**Figure S31.** $^{1}$H NMR (500 MHz, $CDCl_3$) of **L2**

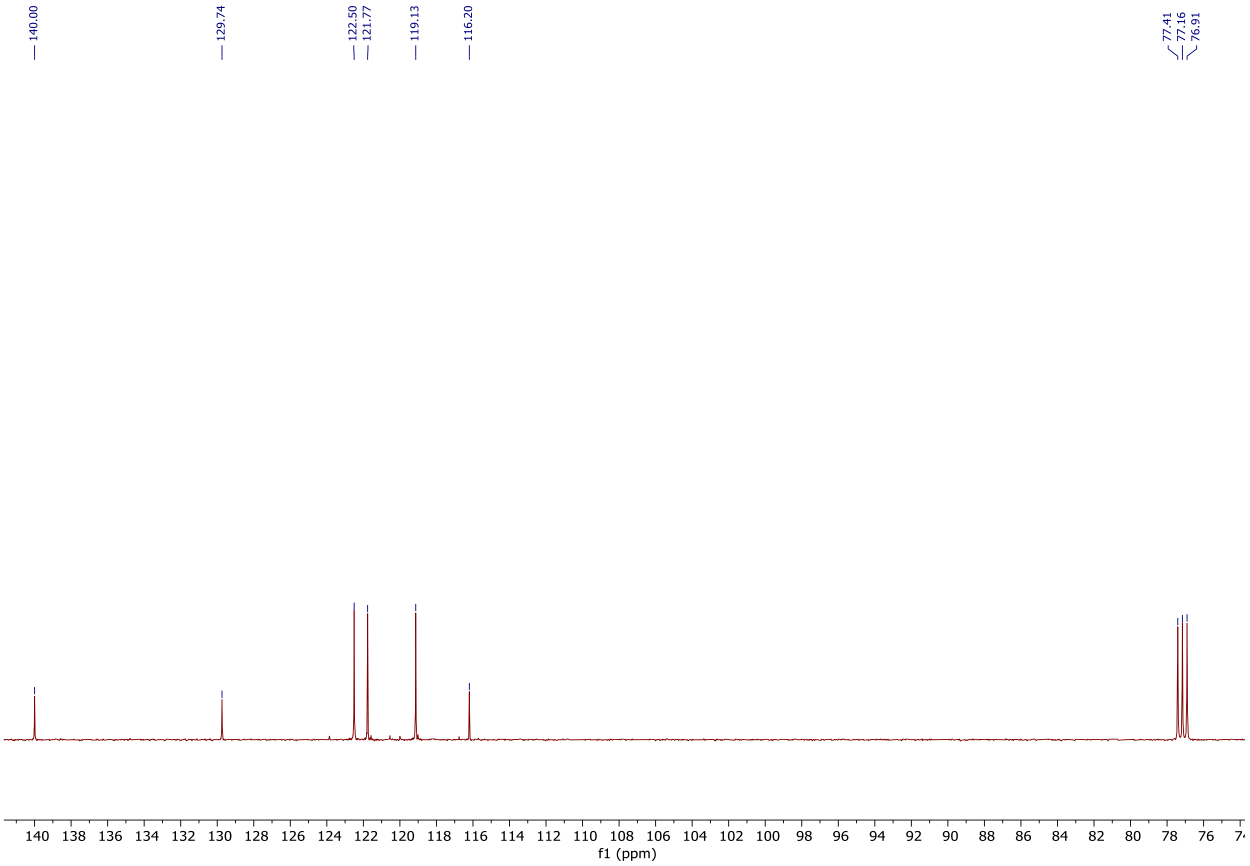


**Figure S32**. $^{13}C\{^{1}H\}$ NMR (126 MHz, $CDCl_3$) of **L2**

| Meas. m/z | Ion Formula | m/z | err [ppm] |
|---|---|---|---|
| 353.9237 | C12H10Br2N3 | 353.9247 | 2.9 |

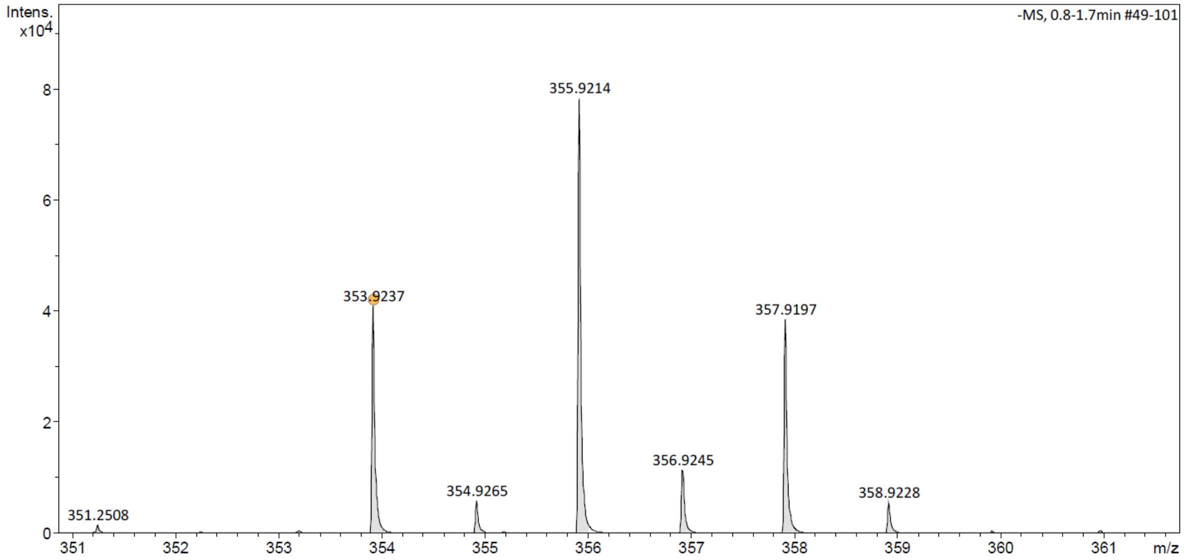


**Figure S33**. Mass spectrum of **L2**

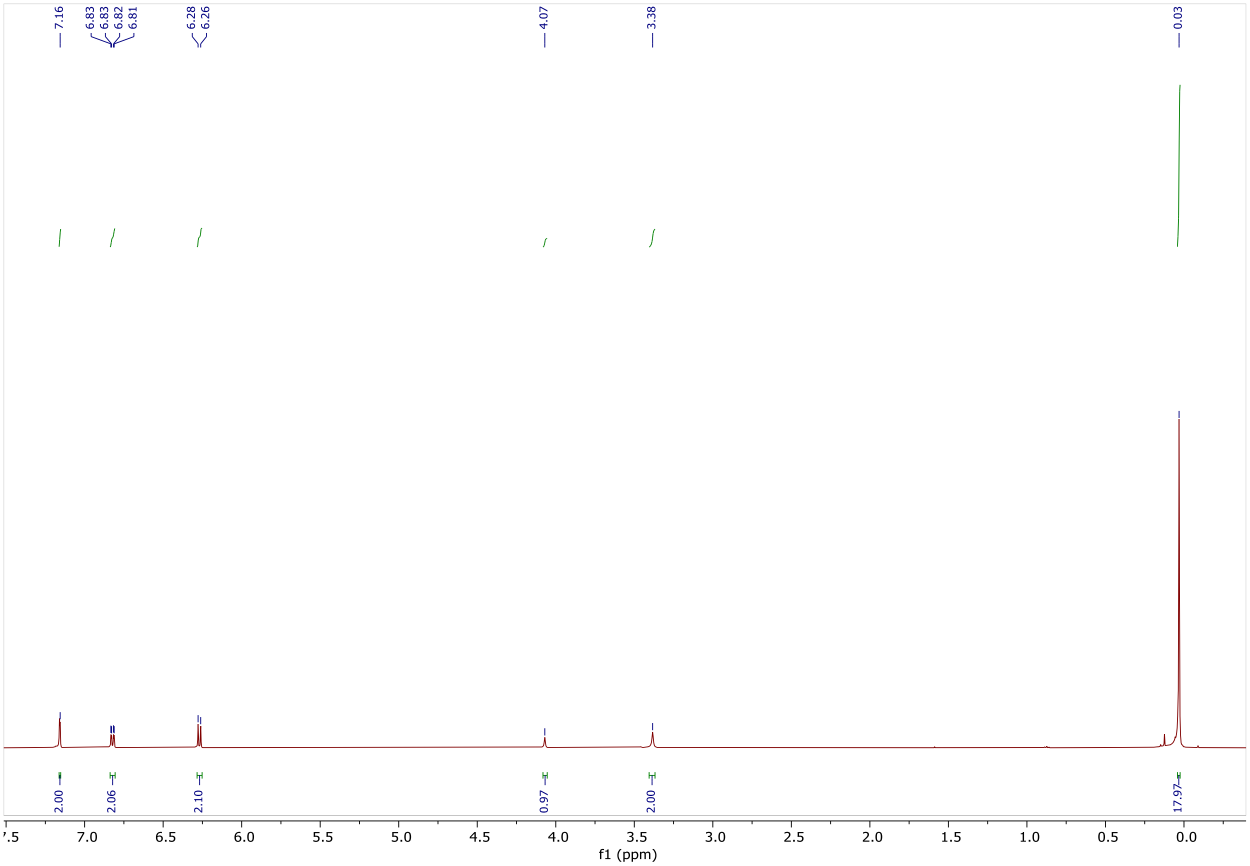


**Figure S34**. $^{1}H$ NMR (500 MHz, $C_6D_6$) of **1b**

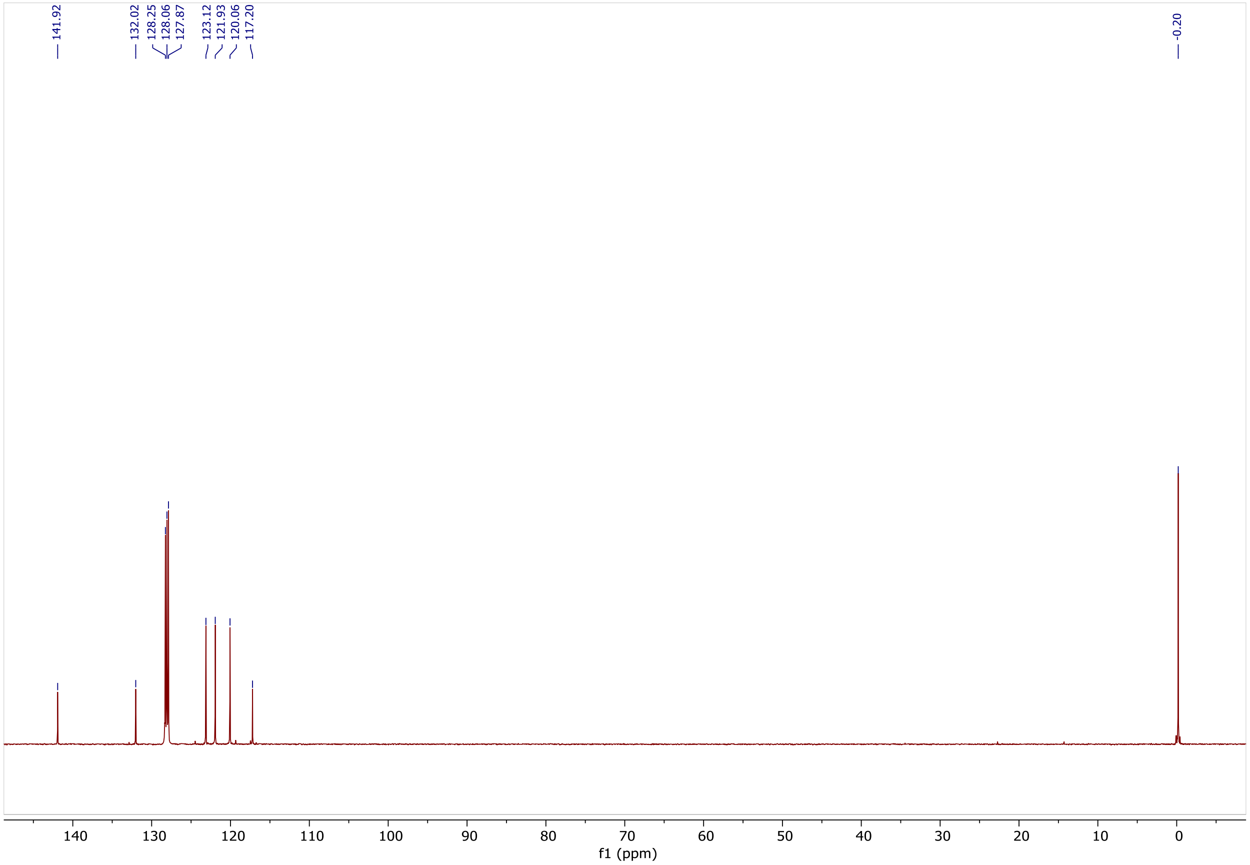


**Figure S35**. $^{13}C$ {$^{1}H$} NMR (126 MHz, $C_6D_6$) of **1b**

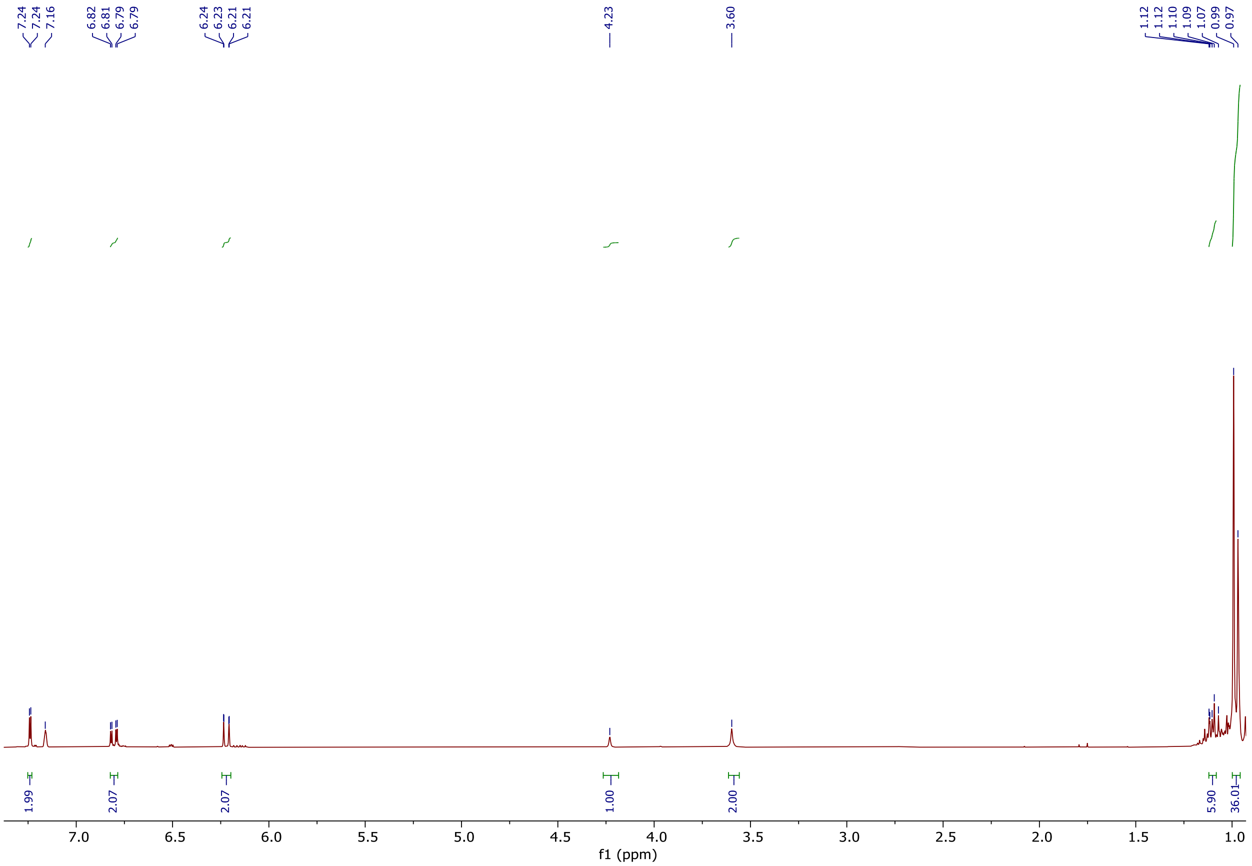


**Figure S36**. $^{1}$H NMR (300 MHz, $C_6D_6$) of **1c**

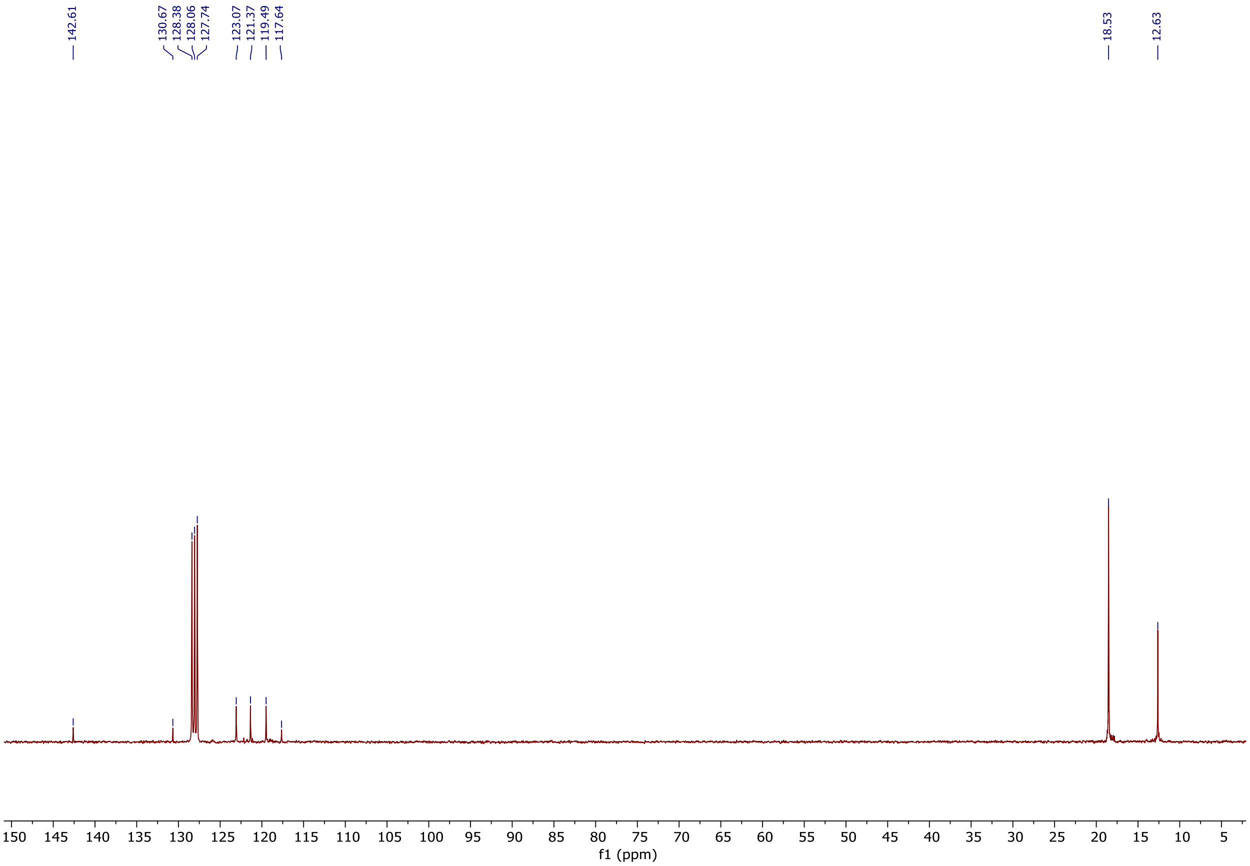


**Figure S37**. $^{13}C$ {$^{1}H$} NMR (75 MHz, $C_6D_6$) of **1c**

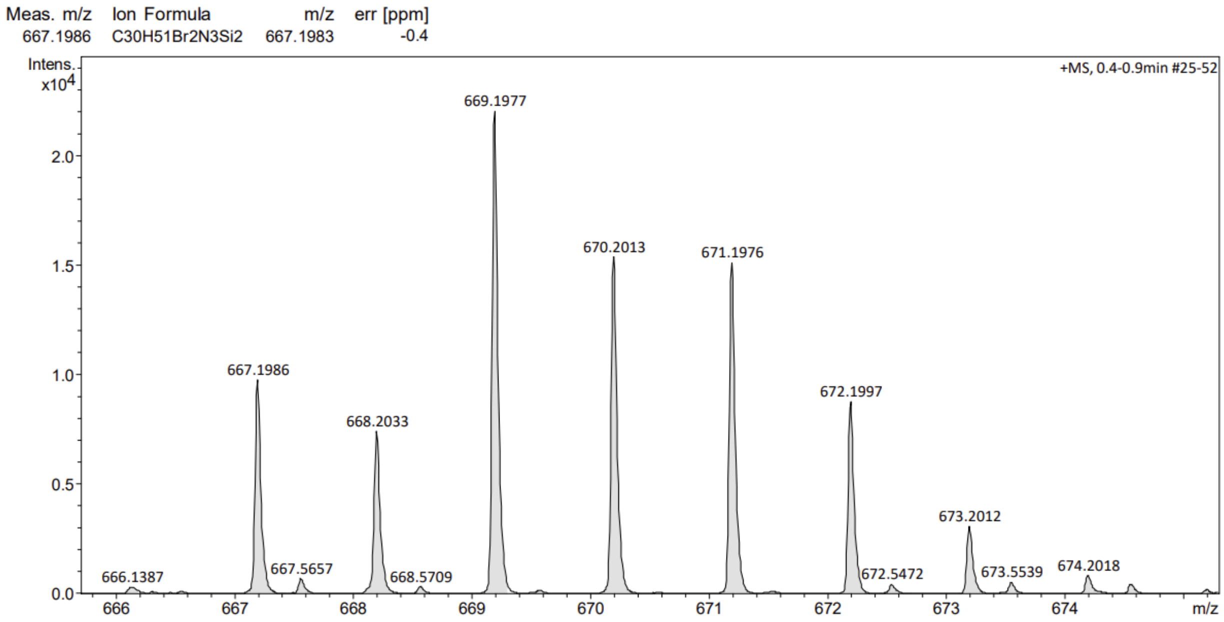


**Figure S38**. Mass spectrum of **1c**

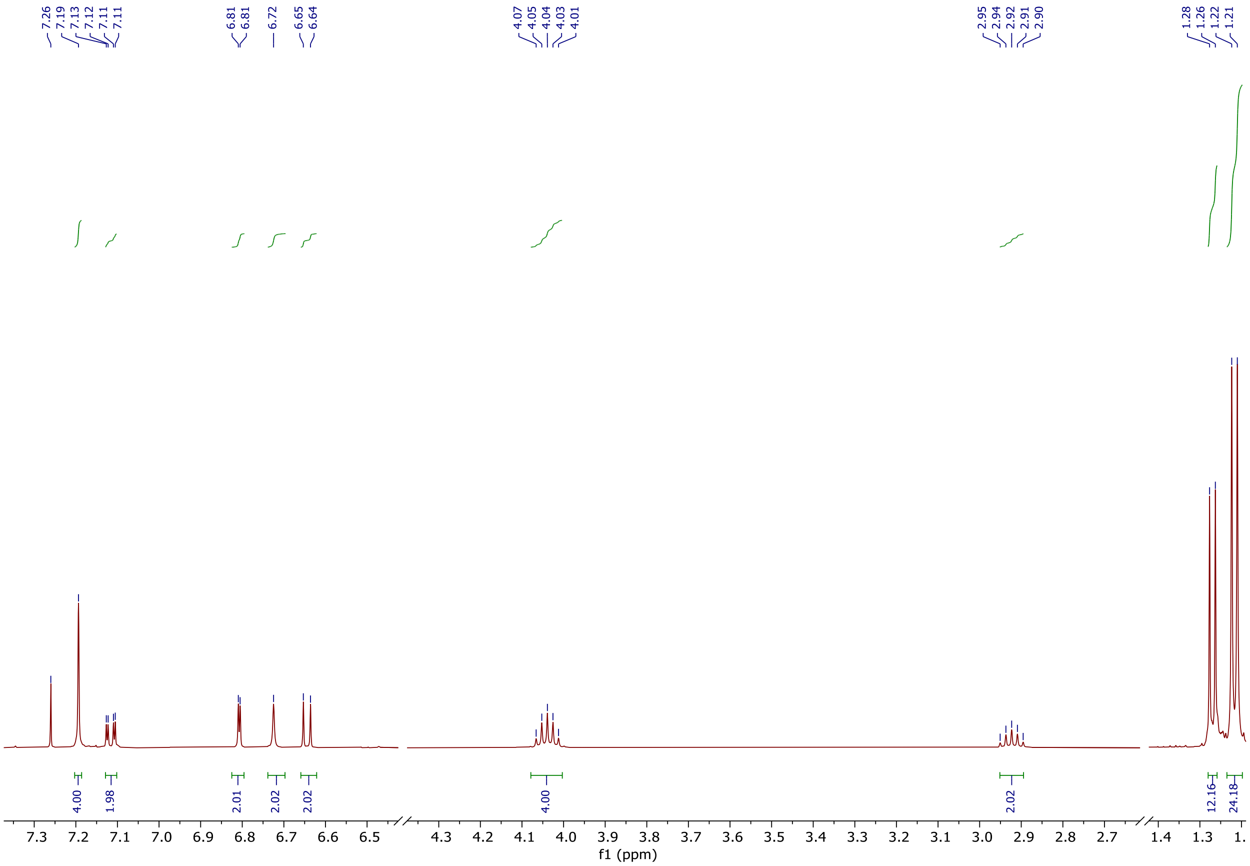


**Figure S39**. $^{1}H$ NMR (500 MHz, $CDCl_3$) of **1e**

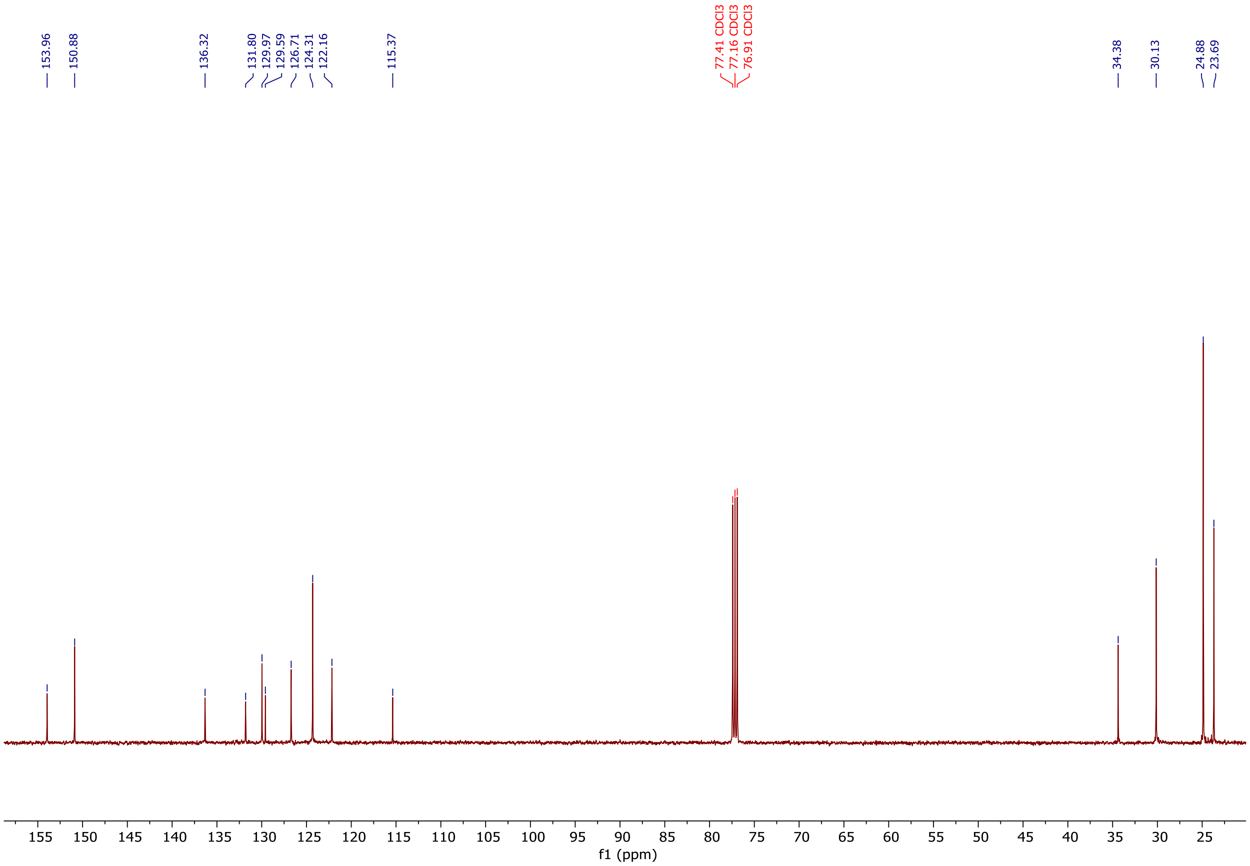


**Figure S40**. $^{13}C$ {$^1H$} NMR (126 MHz, $CDCl_3$) of **1e**

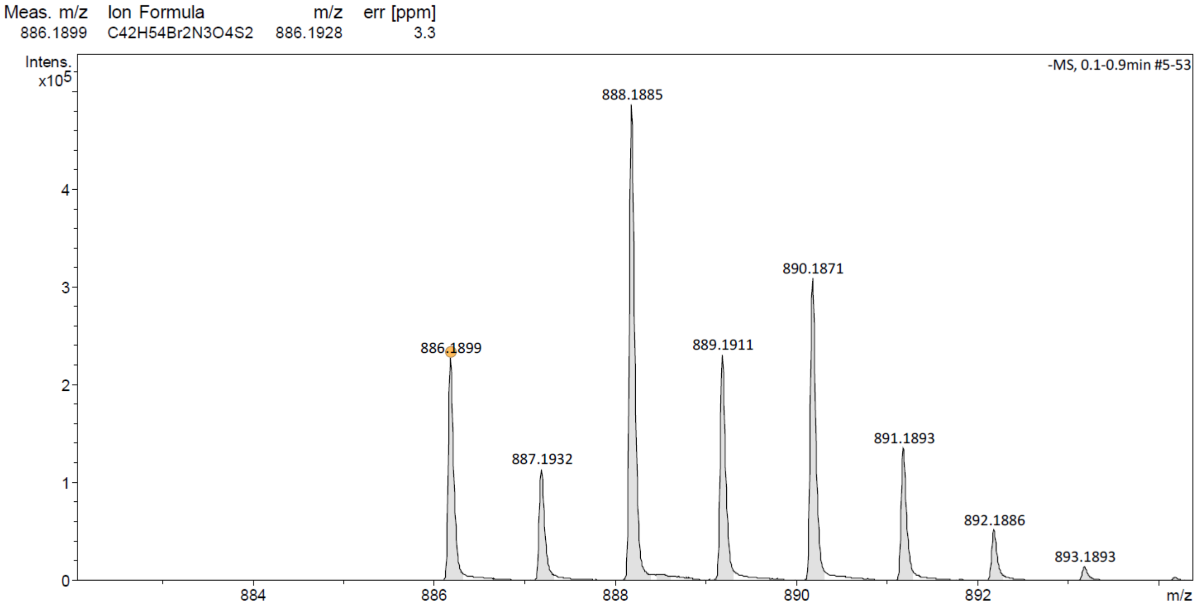


**Figure S41**. Mass spectrum of **1e**

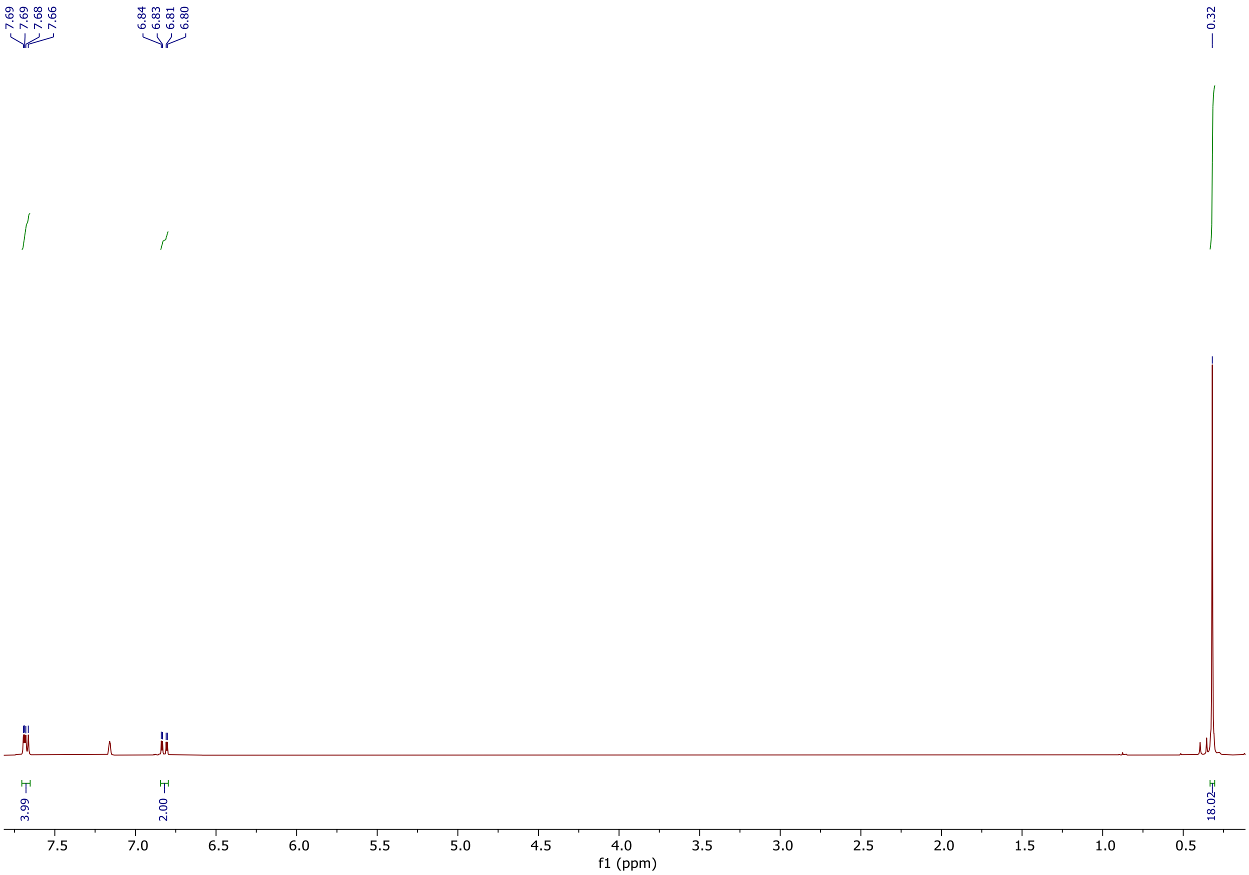


**Figure S42**. $^1$H NMR (300 MHz, $C_6D_6$) of **3b**

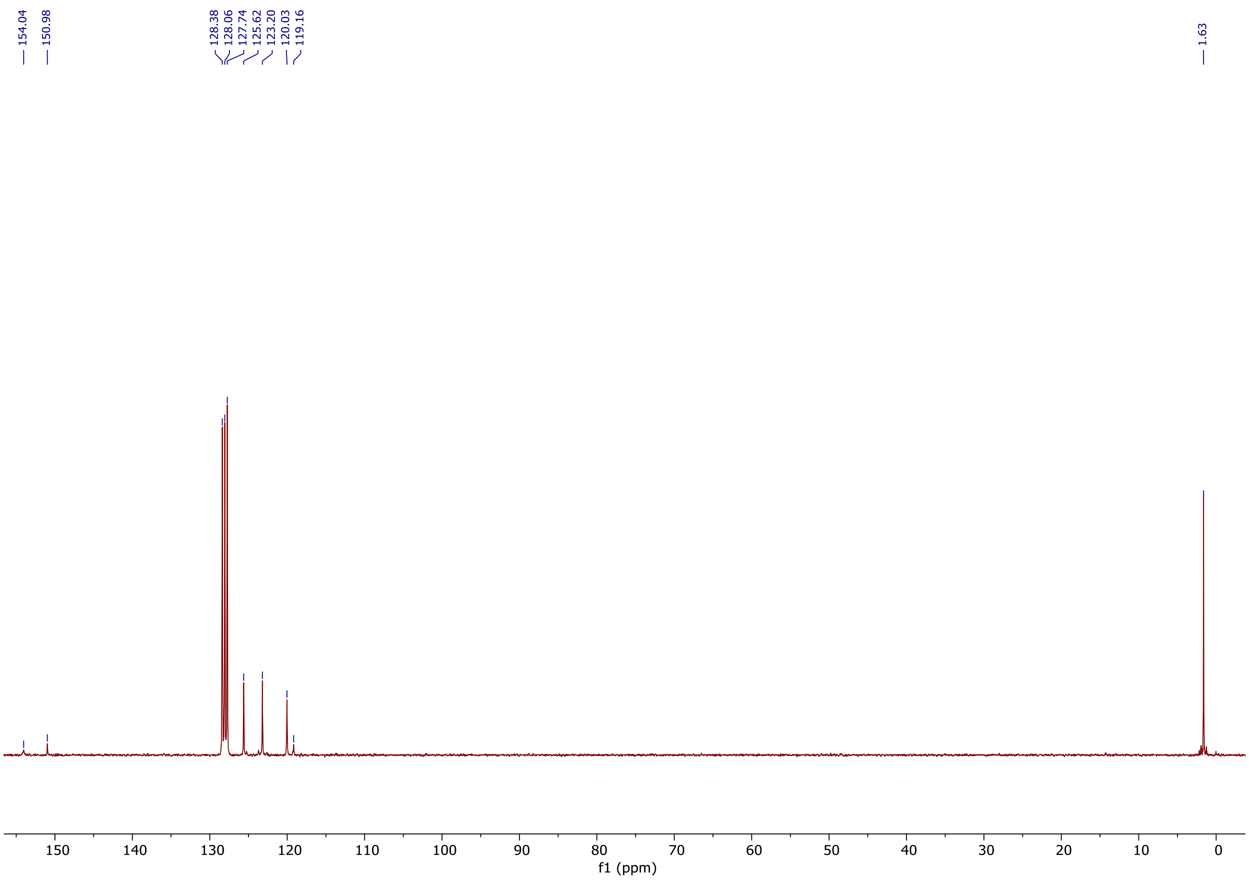


**Figure S43**. $^{13}C\ \{^{1}H\}$ NMR (75 MHz, $C_6D_6$) of **3b**

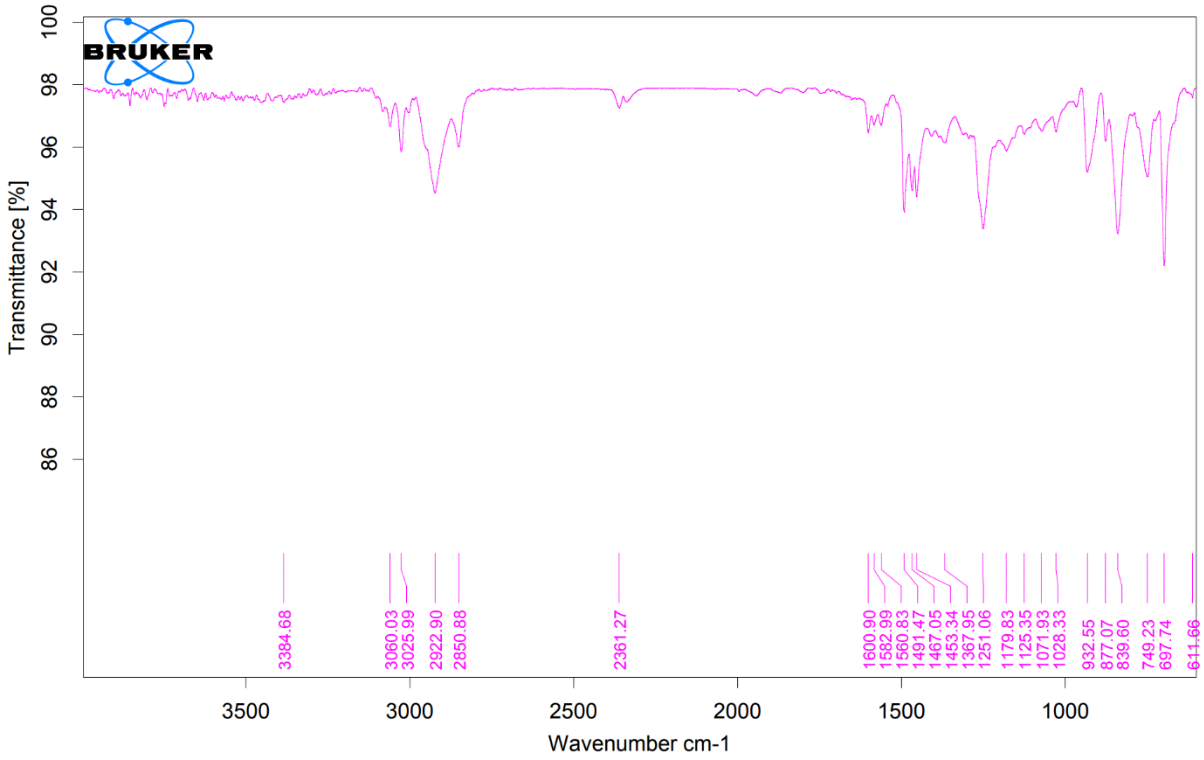


**Figure S44**. Infrared spectrum of **3b**

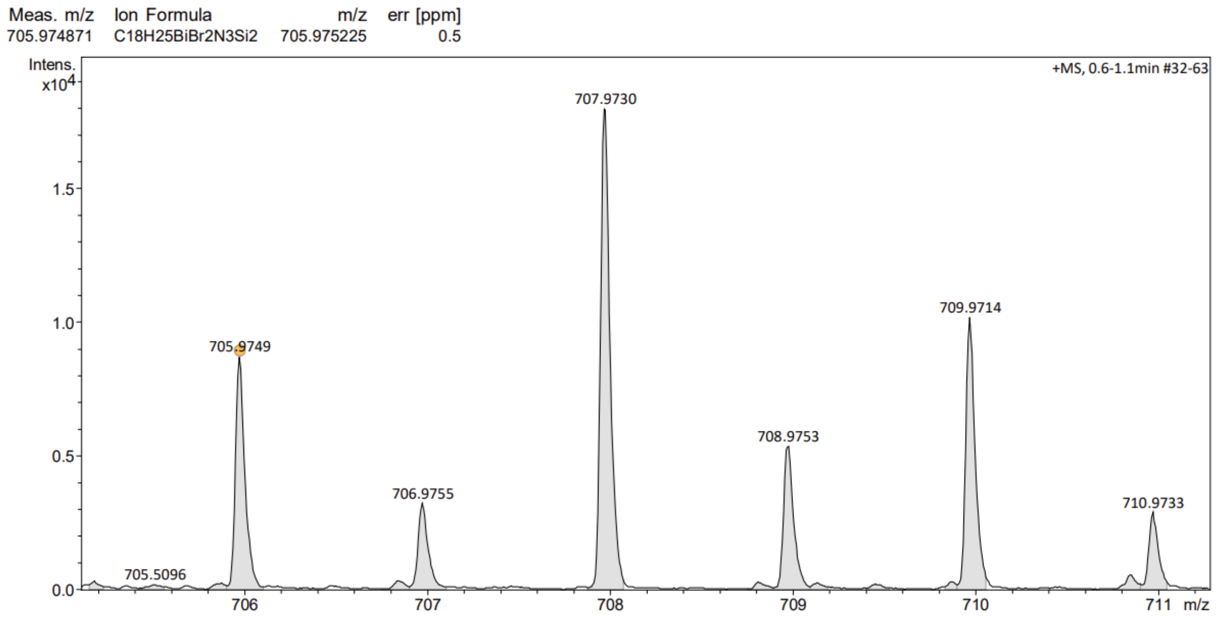


**Figure S45**. Mass spectrum of **3b**

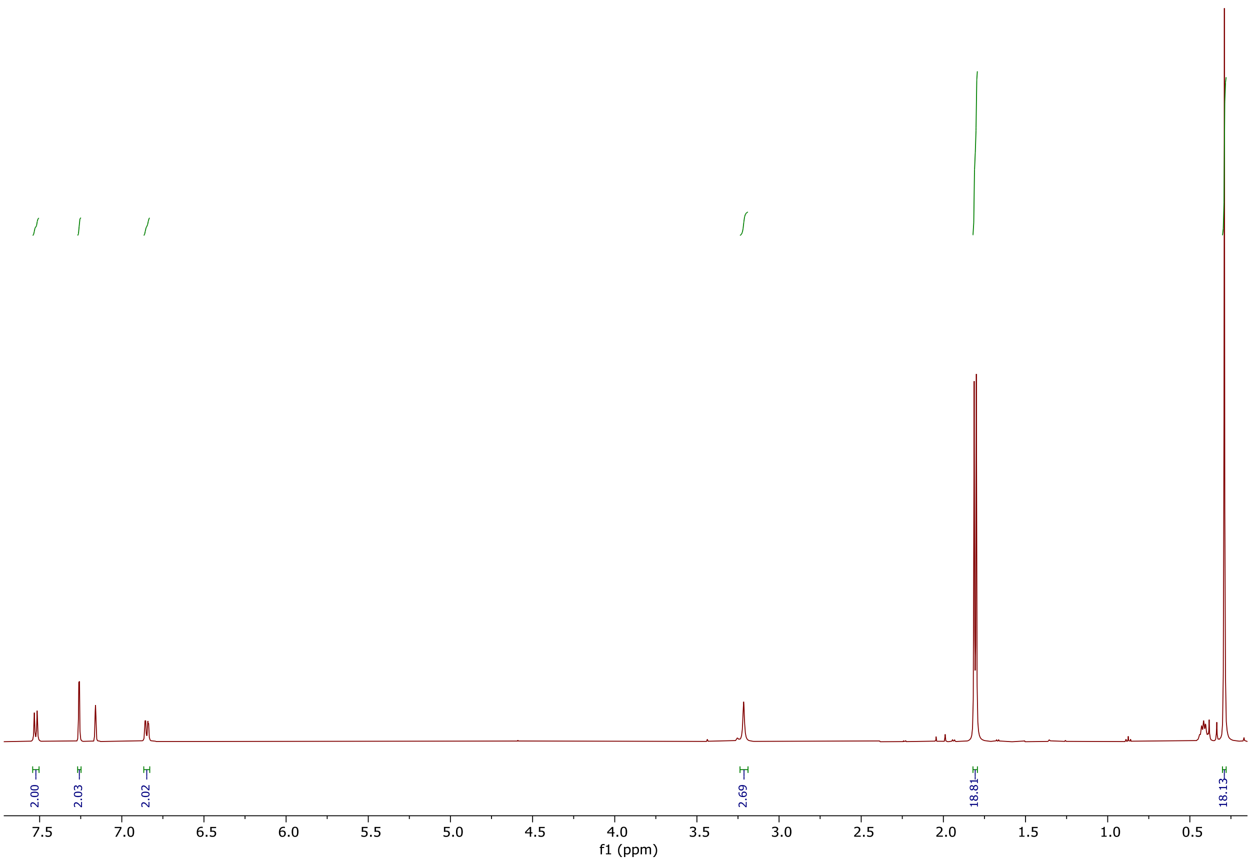


**Figure S46**. $^{1}H$ NMR (500 MHz, $C_6D_6$) of **2b**

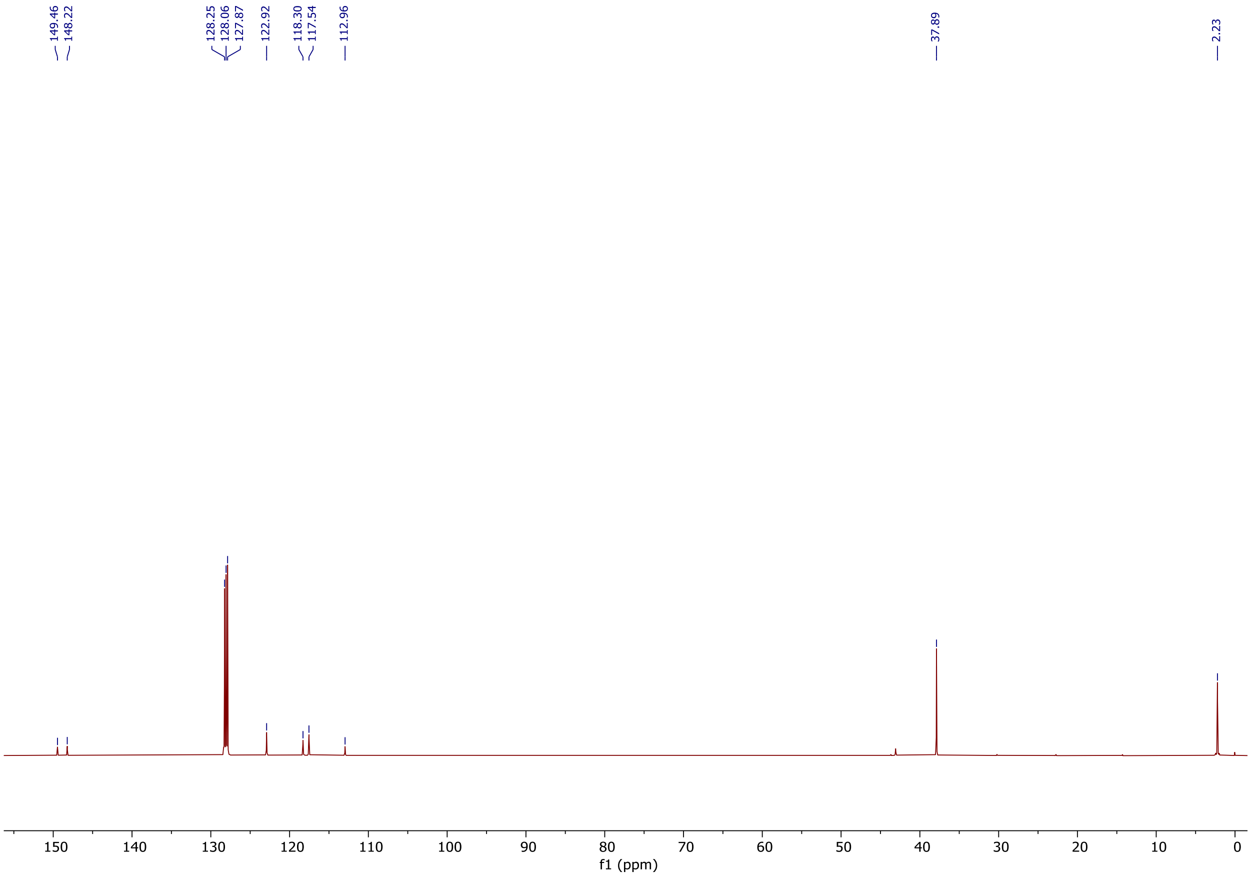


**Figure S47**. $^{13}C$ {$^{1}H$} NMR (126 MHz, $C_6D_6$) of **2b**

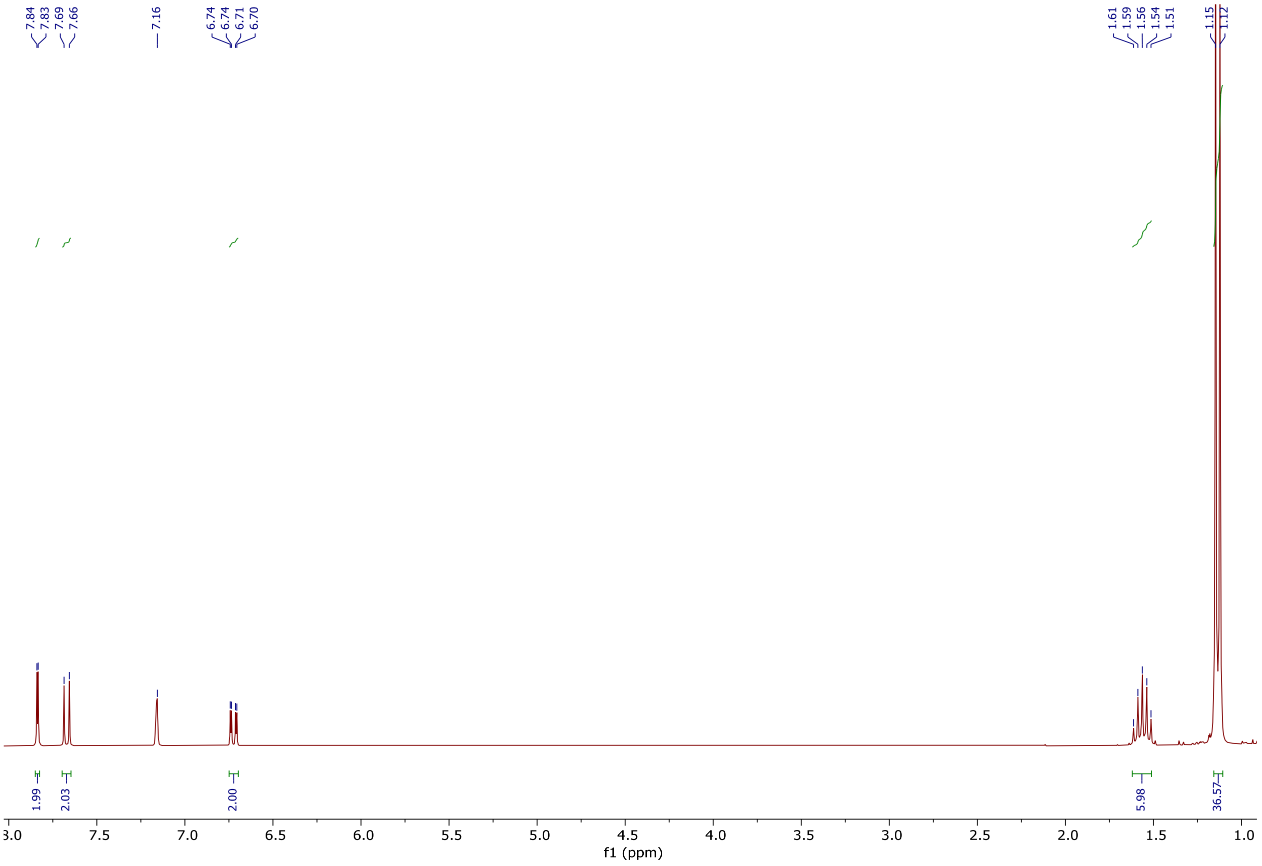

**Figure S48**. $^{1}H$ NMR (300 MHz, $C_6D_6$) of **3c**

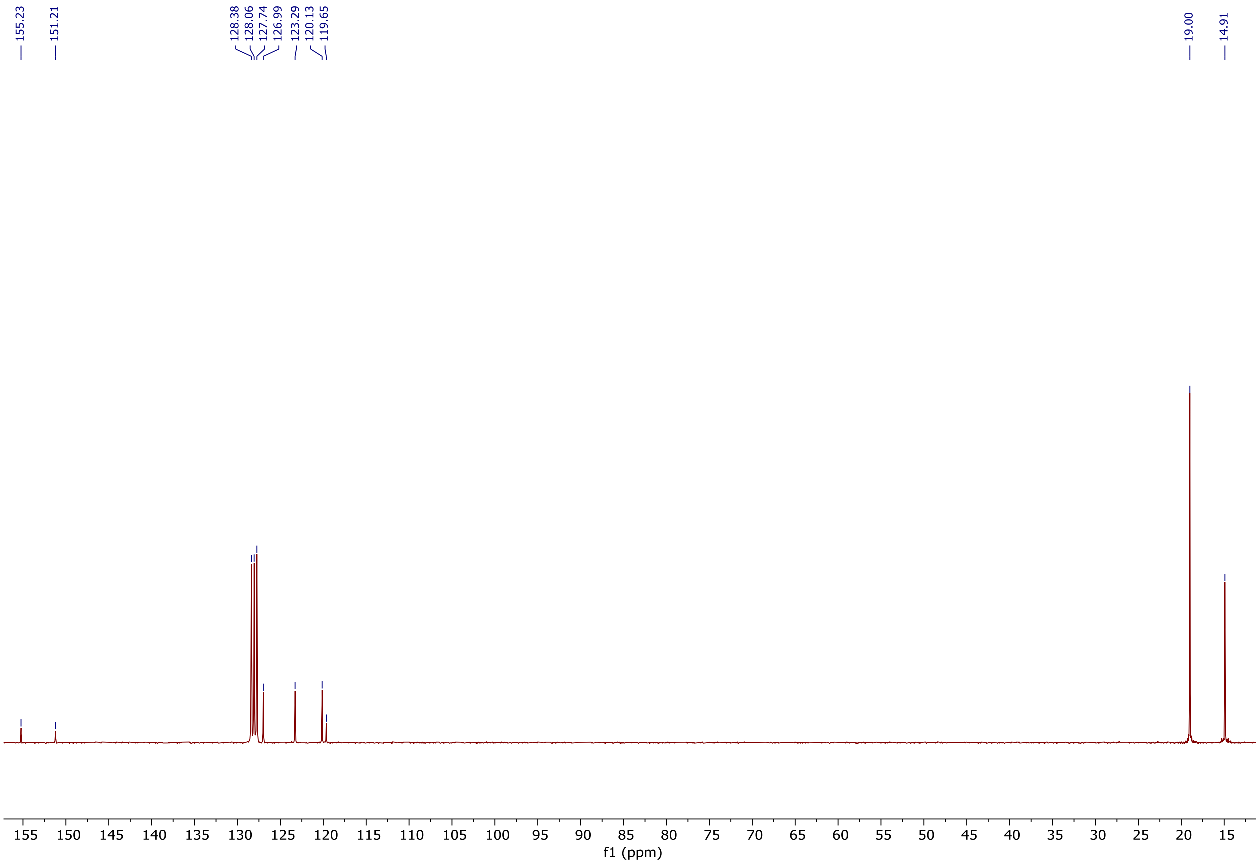


**Figure S49**. $^{13}C\{^1H\}$ NMR (75 MHz, $C_6D_6$) of **3c**

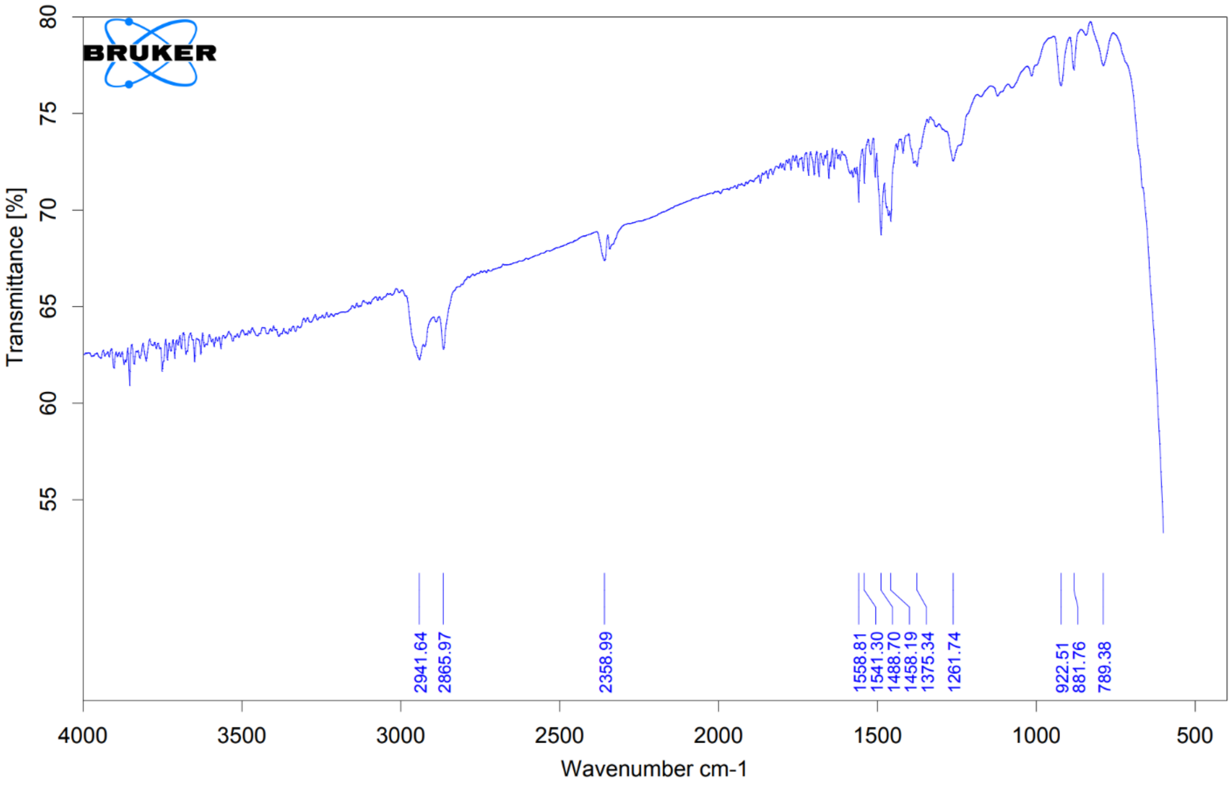


**Figure S50**. Infrared spectrum of **3c**

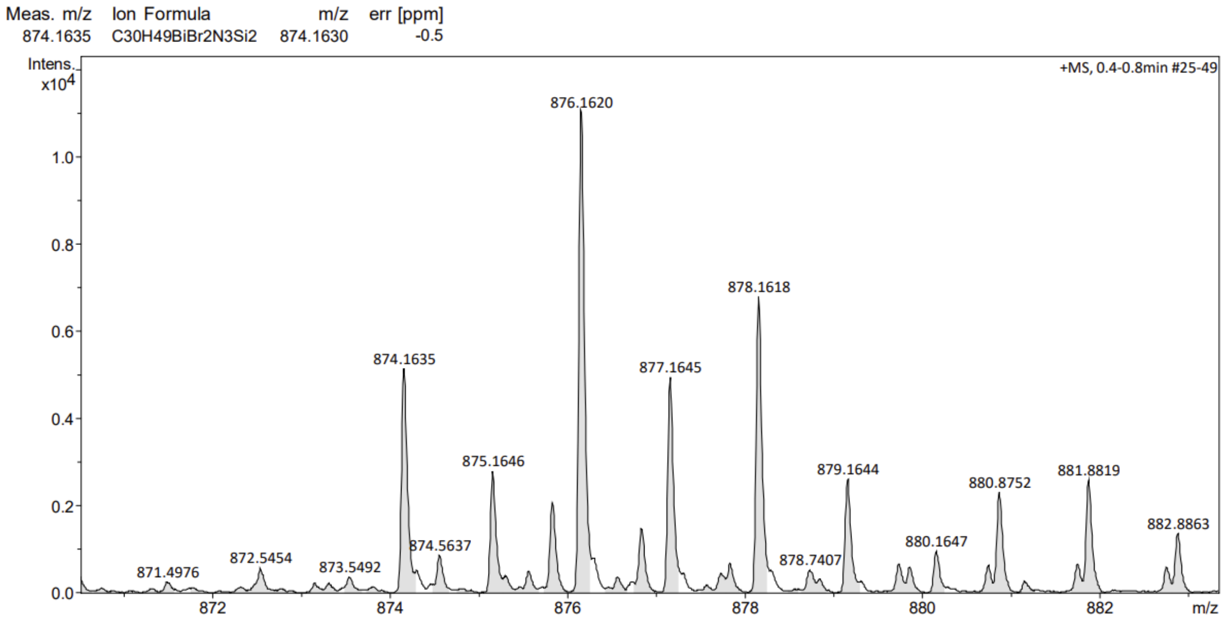


**Figure S51**. Mass spectrum of **3c**

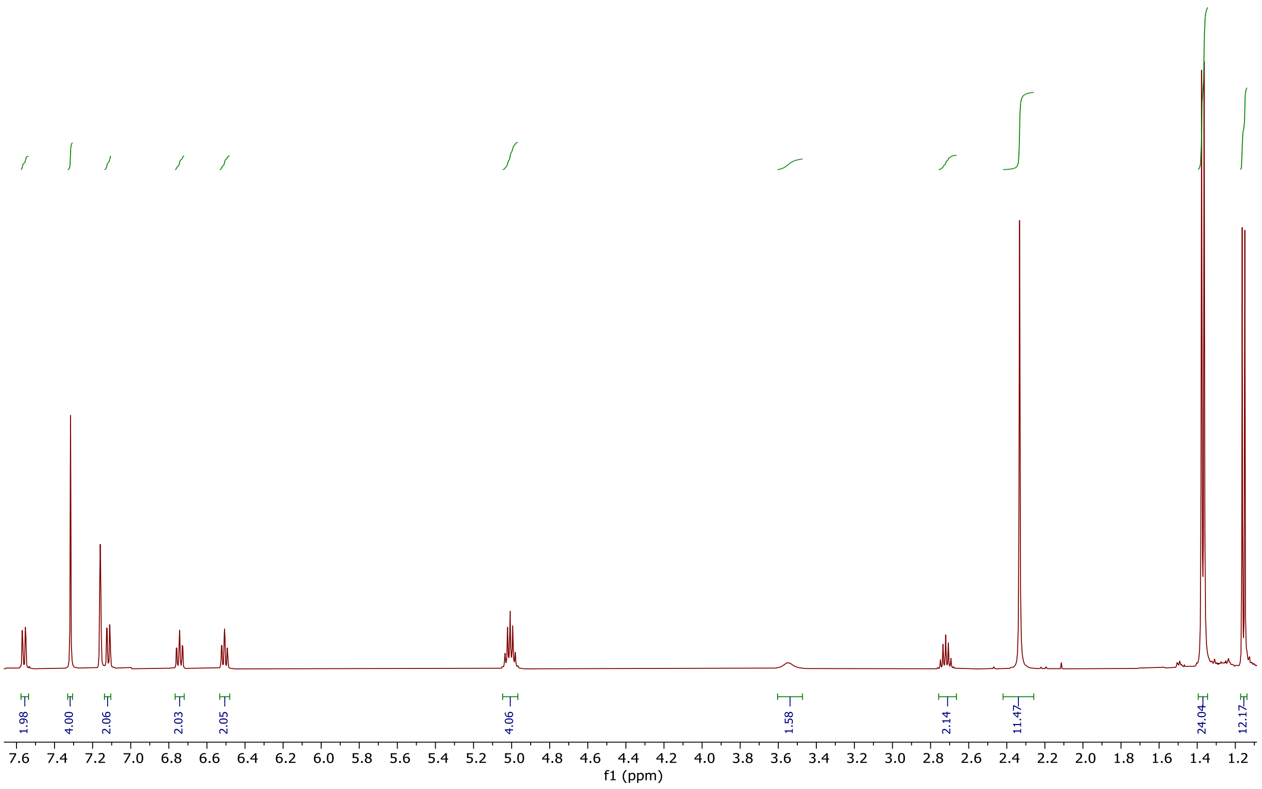


**Figure S52**. $^{1}H$ NMR (500 MHz, $C_6D_6$) of **2d**

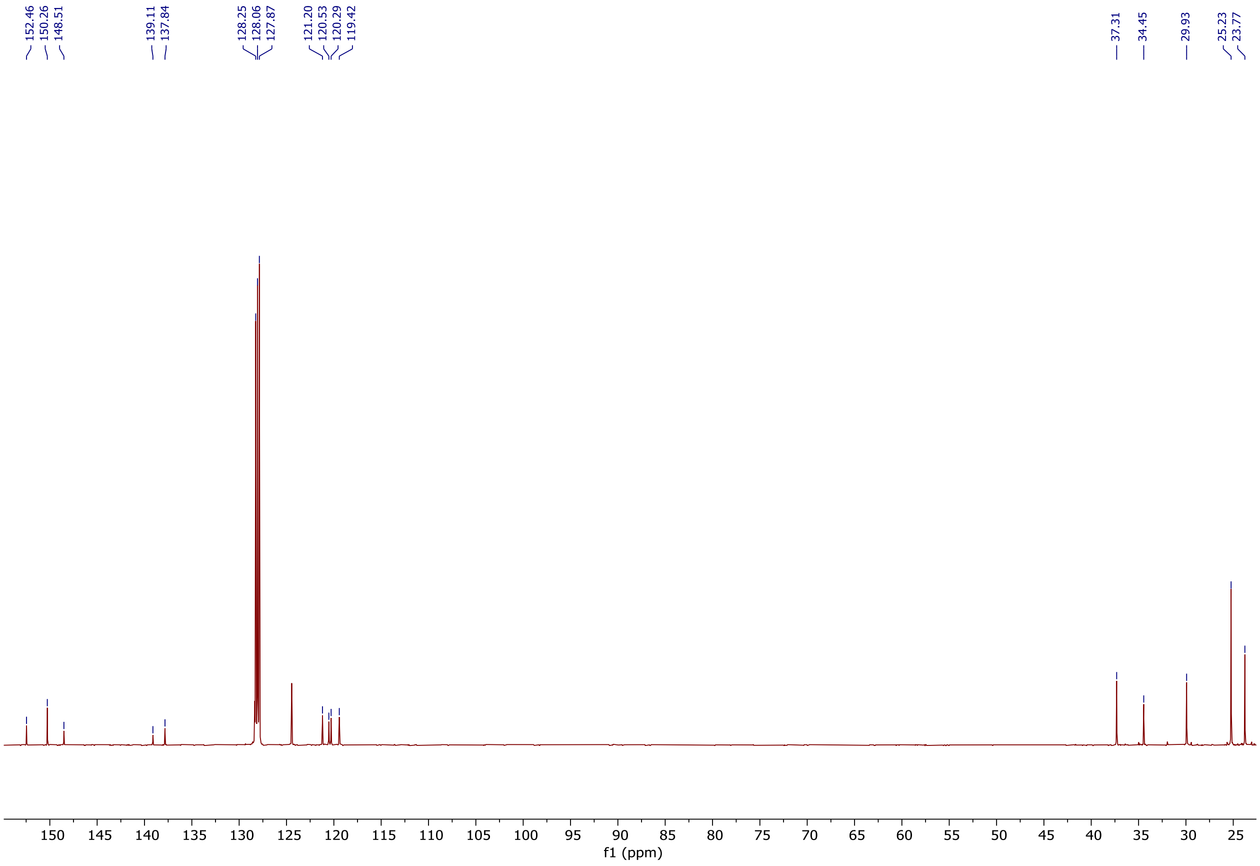


**Figure S53**. $^{13}C$ {$^{1}H$} NMR (126 MHz, $C_6D_6$) of **2d**

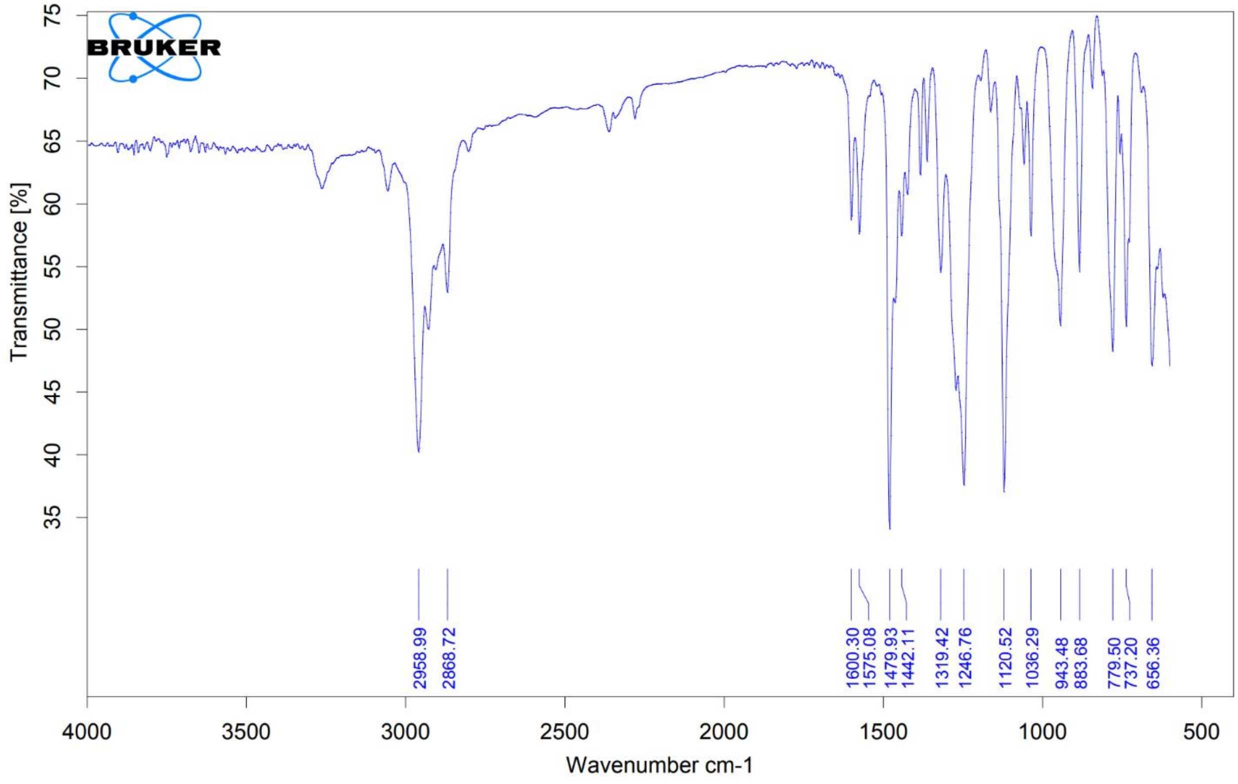


**Figure S54**. Infrared spectrum of **2d**

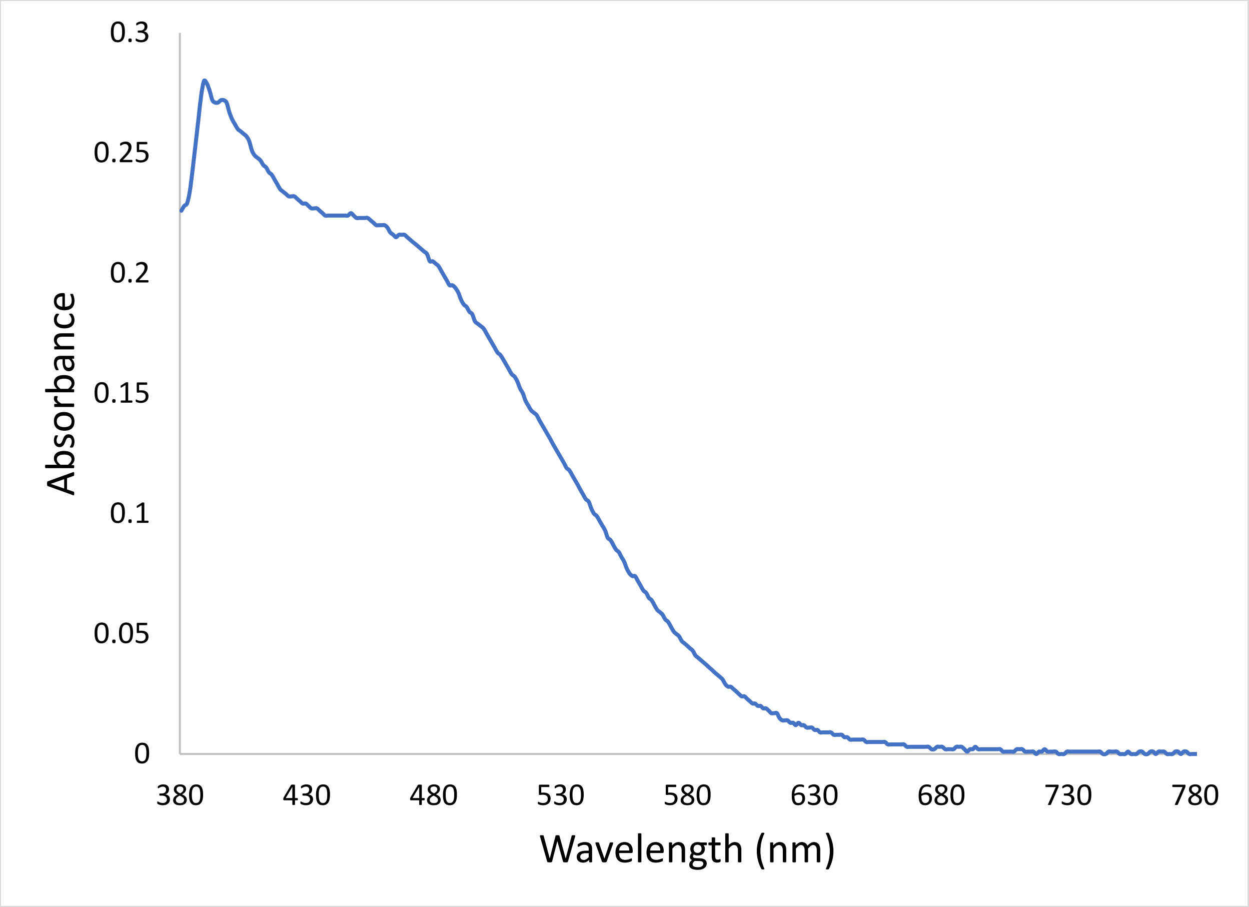


**Figure S55.** UV-Vis absorption spectrum of **2d**

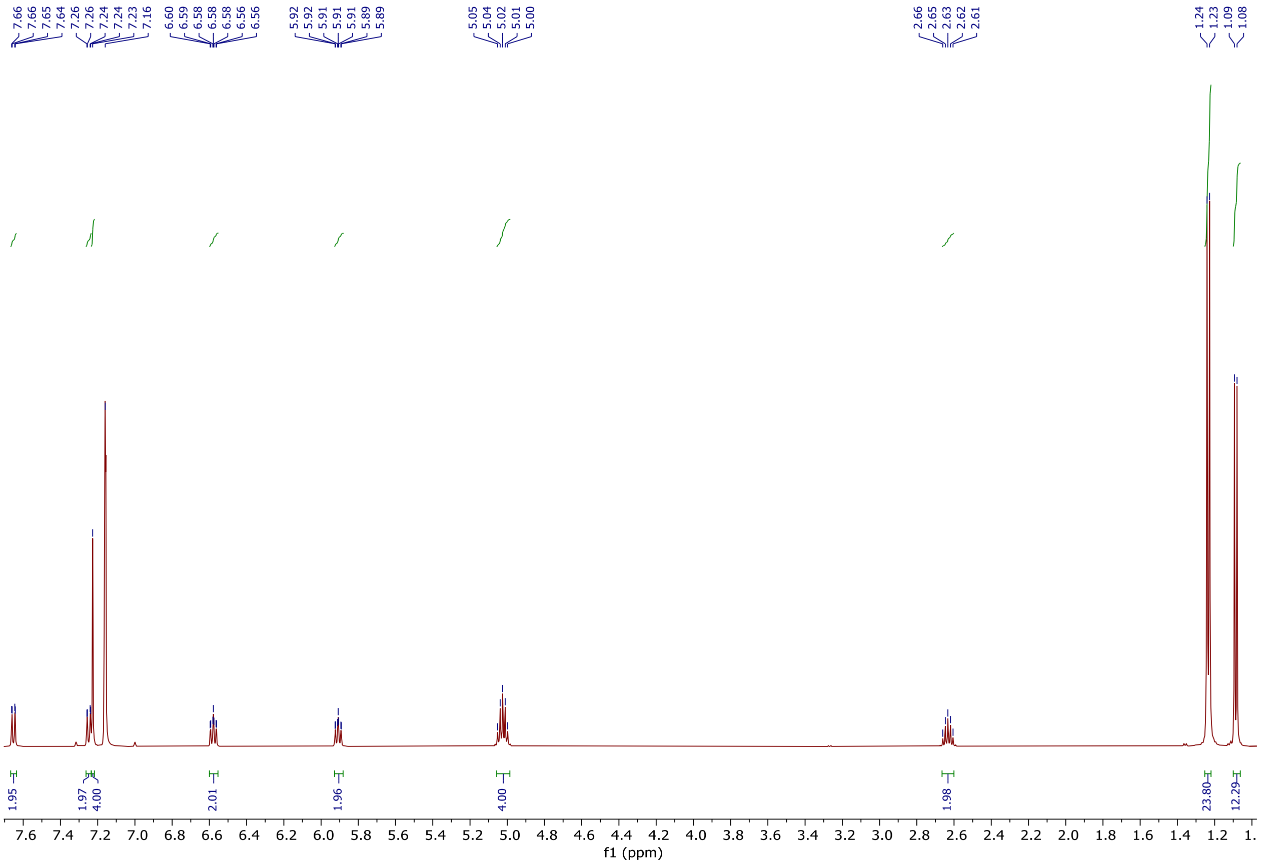

**Figure S56**. $^{1}H$ NMR (500 MHz, $C_6D_6$) of **3d**

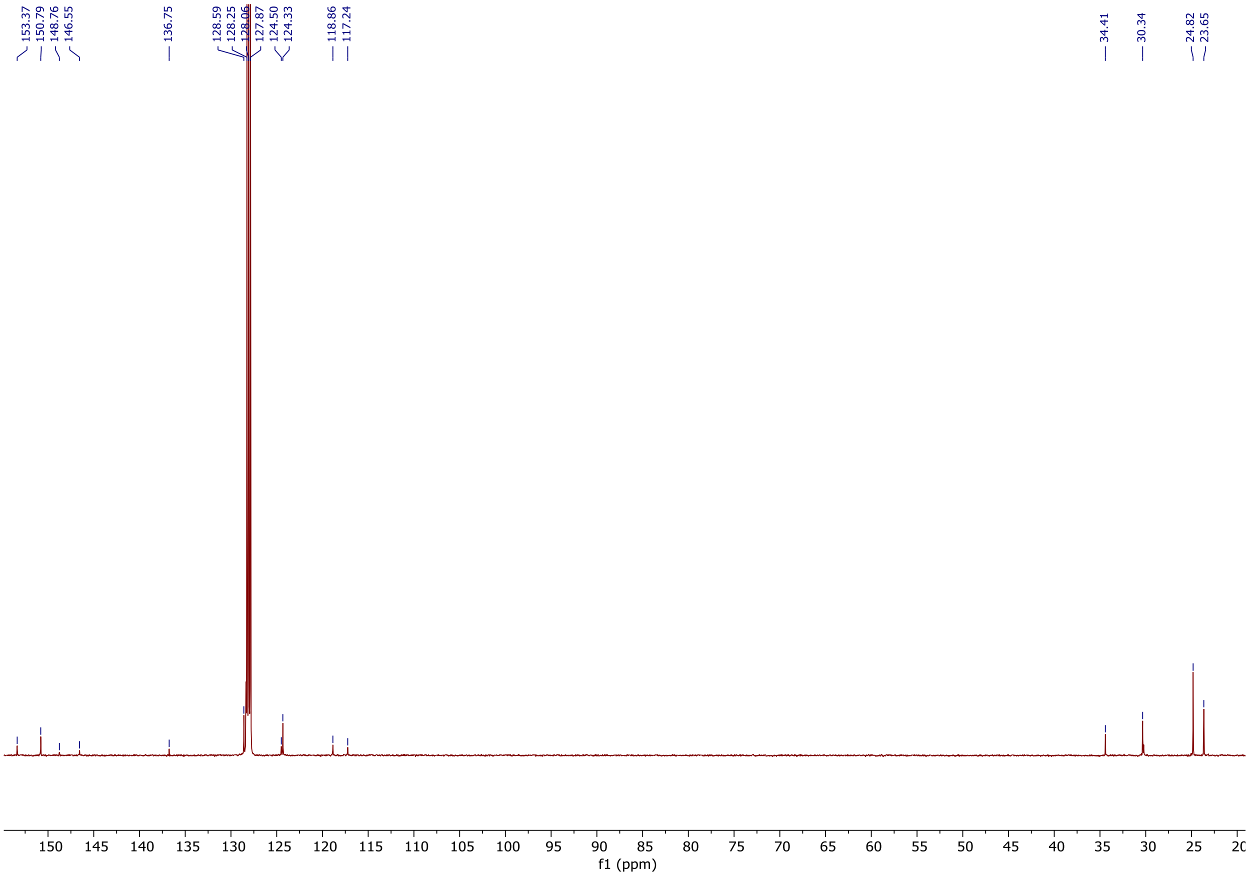


**Figure S57**. $^{13}C\{^{1}H\}$ NMR (126 MHz, $C_6D_6$) of **3d**

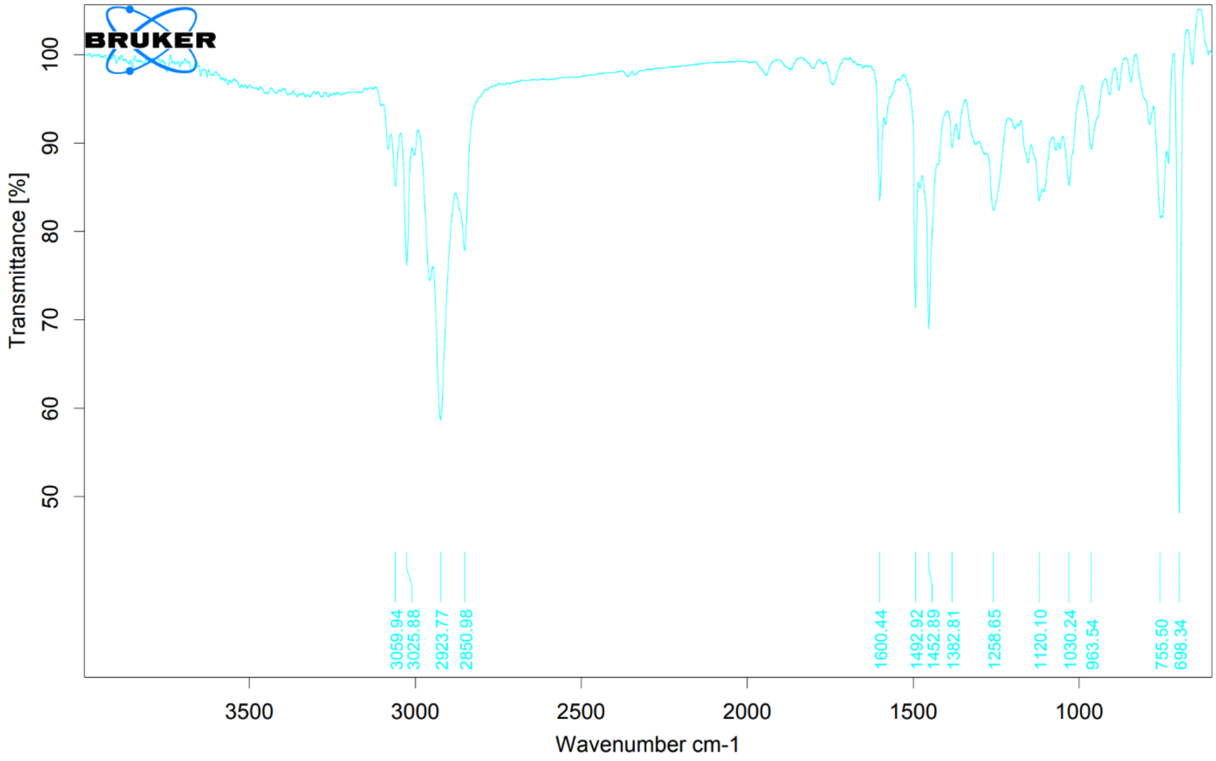


**Figure S58**. Infrared spectrum of **3d**

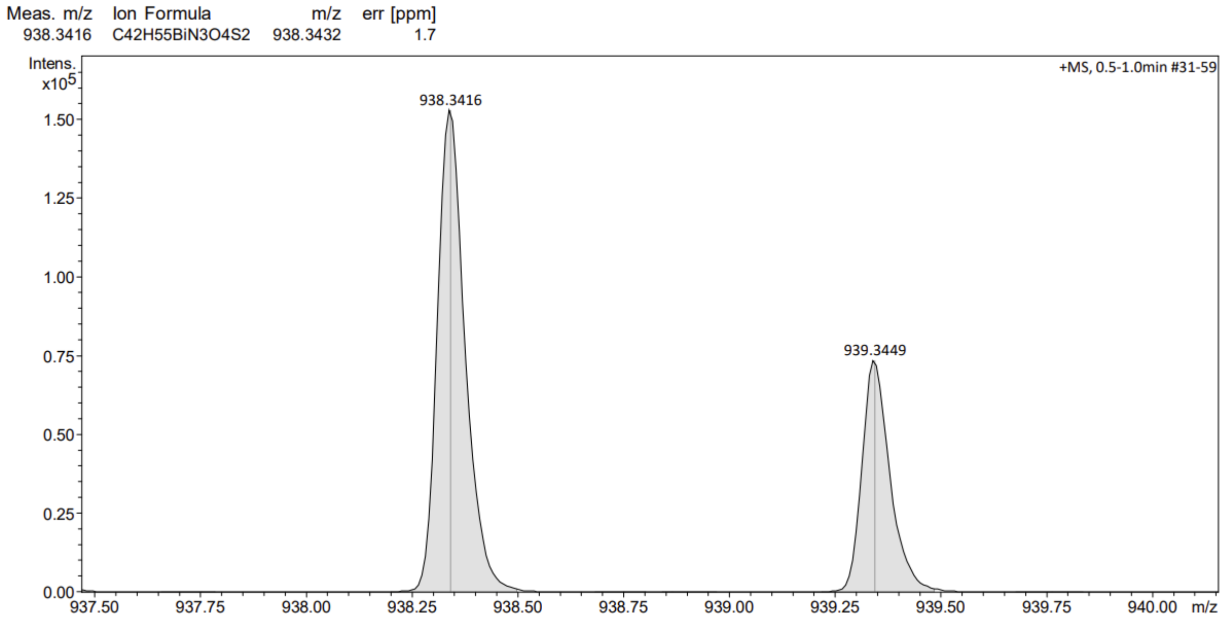


**Figure S59**. Mass spectrum of **3d**

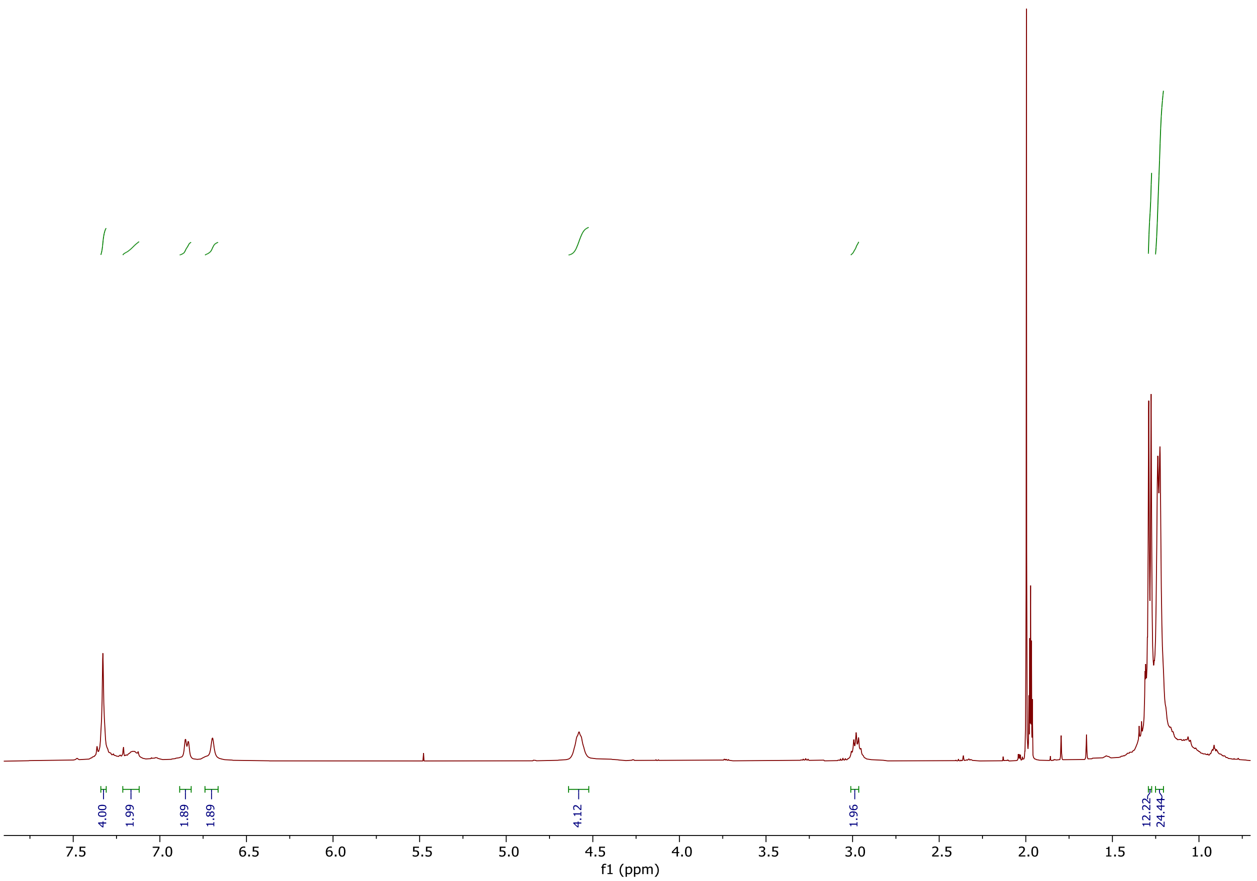


**Figure S60**. $^1$H NMR (500 MHz, $CD_3CN$) of **3e**

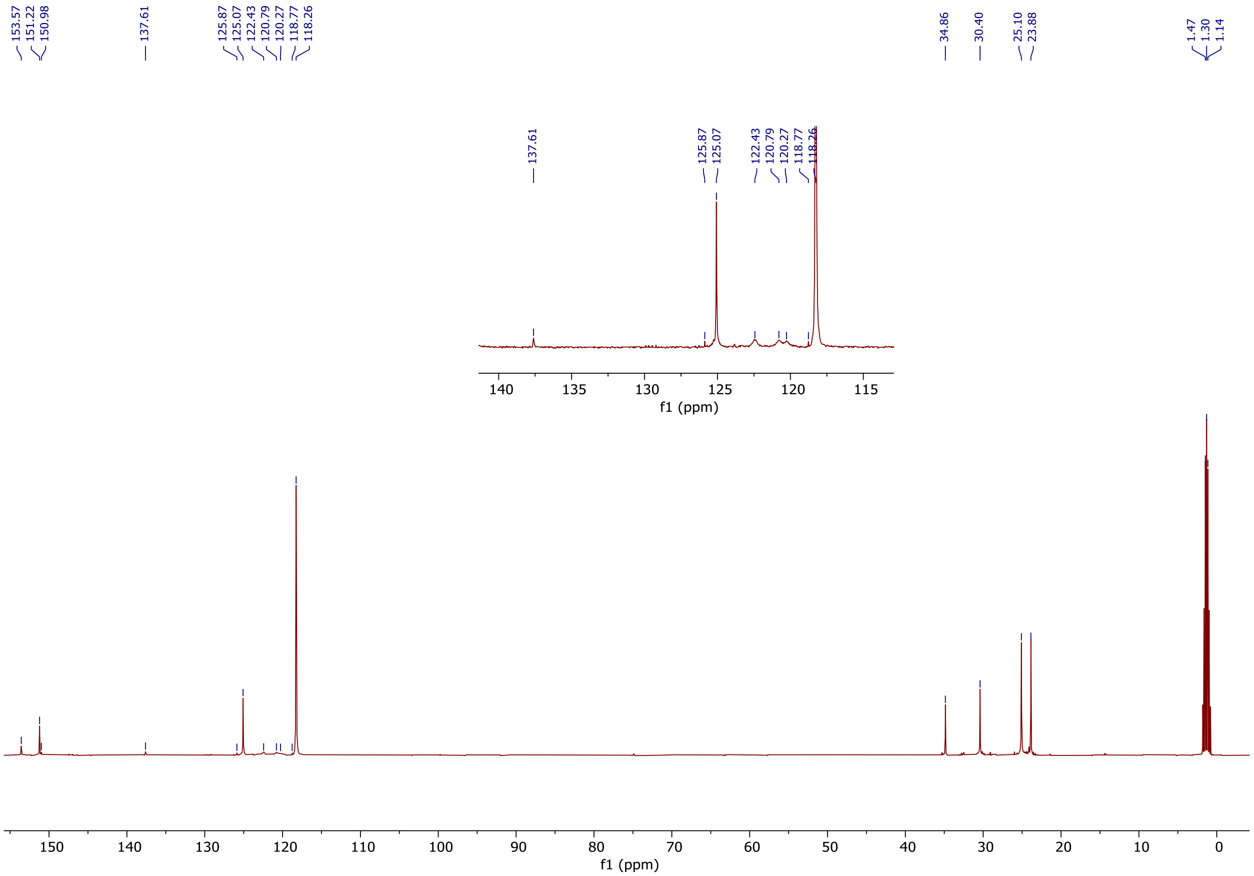


**Figure S61**. $^{13}C\{^1H\}$ NMR (126 MHz, $CD_3CN$) of **3e**

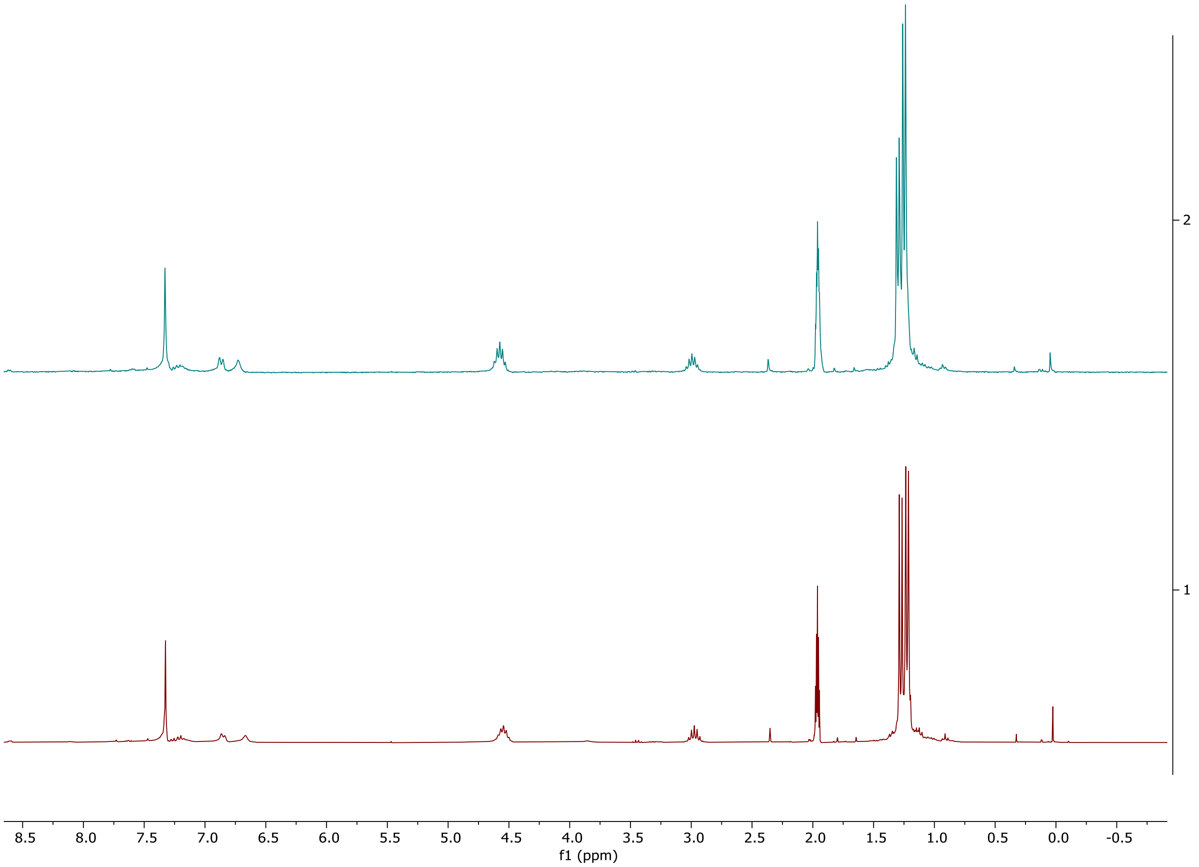


**Figure S62.** $^{1}H$ NMR (300 MHz, $CD_3CN$) of **3e**; attempting to improve signal by heating in NMR probe. Top spectrum = 340 K; bottom spectrum = 300 K.

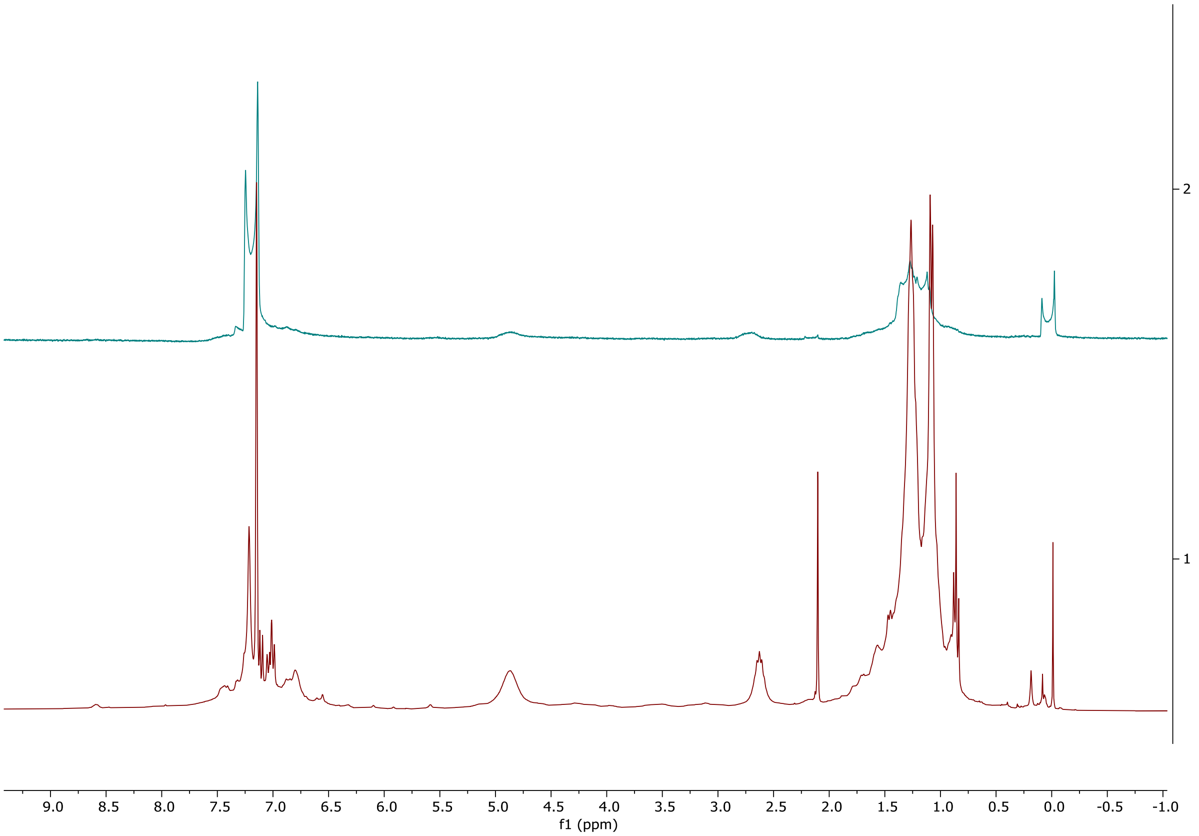


**Figure S63.** $^1$H NMR (300 MHz, $C_6D_6$) of **3e**; attempting to improve signal by heating in NMR probe. Top spectrum = 340 K; bottom spectrum = 300 K. Note extreme broadening observed in non-coordinating solvent.

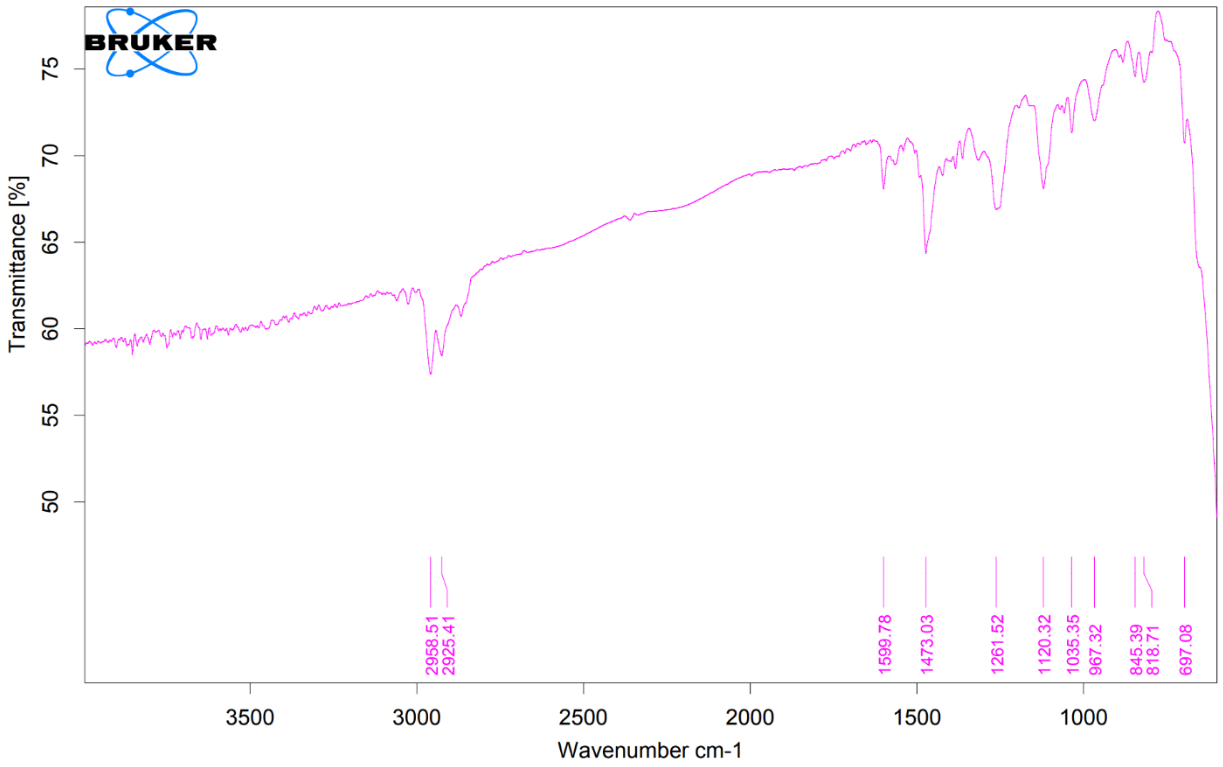


**Figure S64.** Infrared spectrum of **3e**

| Meas. m/z | Ion Formula | m/z | err [ppm] |
|---|---|---|---|
| 1094.1598 | C42H53BiBr2N3O4S2 | 1094.1643 | 4.1 |

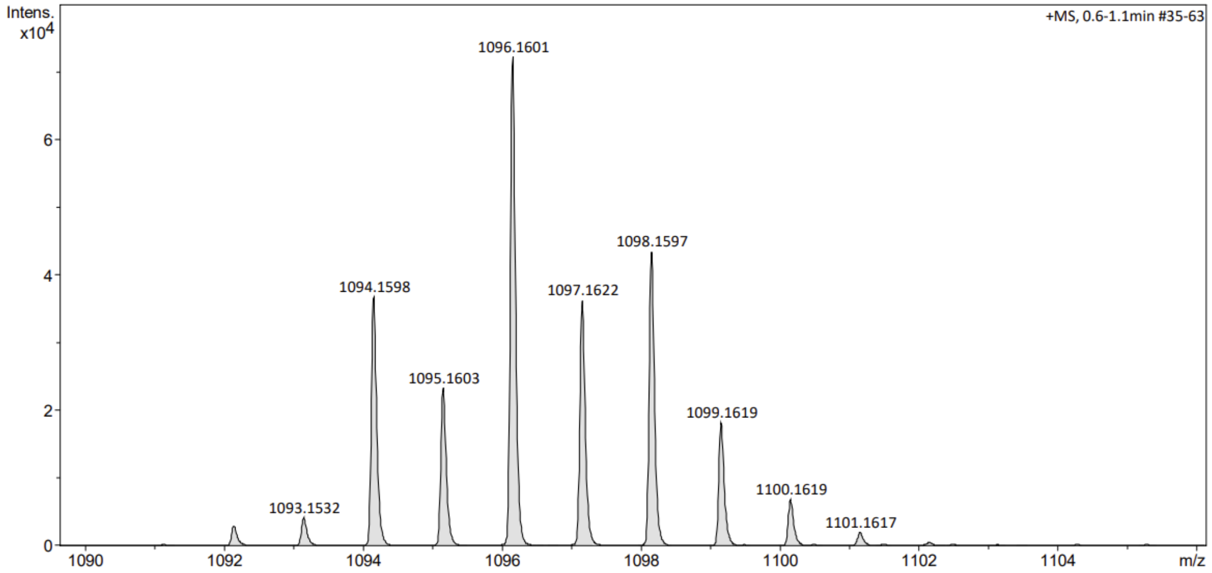


**Figure S65**. Mass spectrum of **3e**

# Polymerization Data:

## $^1$H-NMR

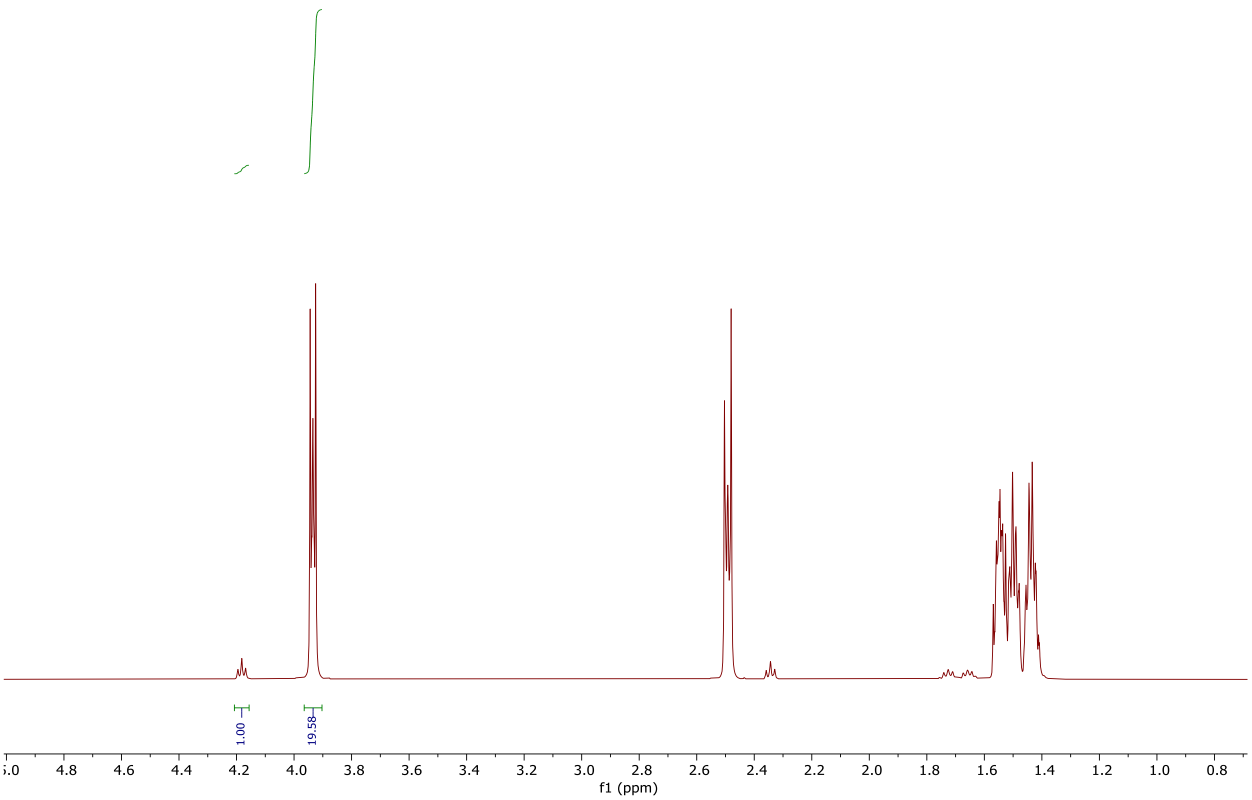


**Figure S66.** $^1$H NMR (300 MHz, $C_6H_6$) of ε-caprolactone polymerization in $C_6H_6$. Chemical shifts are uncorrected; peak further downfield corresponds to polycaprolactone while further upfield corresponds to the monomer. Conditions: **3a** catalyst, 0.02 mol % loading, benzene, 80 °C, 15.5 hours.

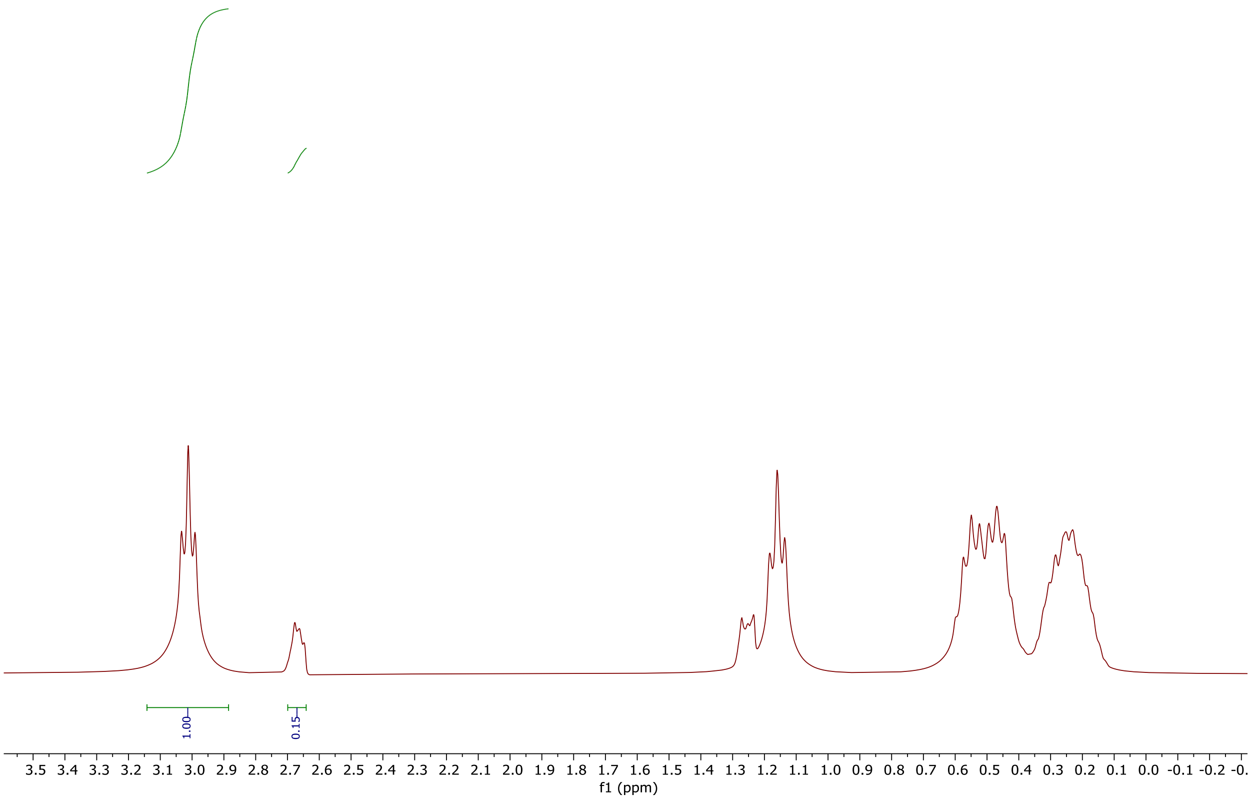


**Figure S67.** $^1$H NMR (300 MHz, $C_6H_6$) of ε-caprolactone polymerization in $C_6H_6$. Chemical shifts are uncorrected; peak further downfield corresponds to polycaprolactone while further upfield corresponds to the monomer. Conditions: **3b** catalyst, 0.02 mol % loading, benzene, 80 °C, 20 hours.

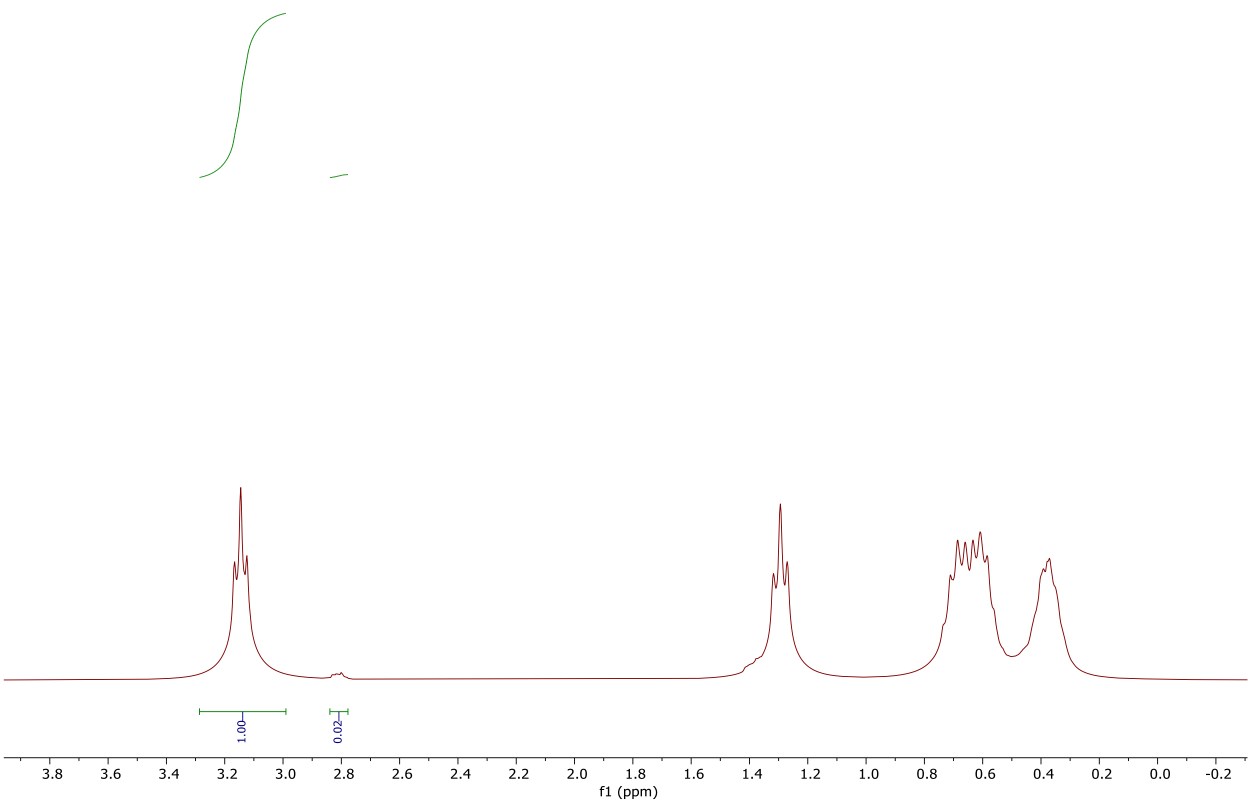


**Figure S68.** $^{1}H$ NMR (300 MHz, $C_6H_6$) of ε-caprolactone polymerization in $C_6H_6$. Chemical shifts are uncorrected; peak further downfield corresponds to polycaprolactone while further upfield corresponds to the monomer. Conditions: **3b** catalyst, 0.1 mol % loading, benzene, 80 °C, 19 hours.

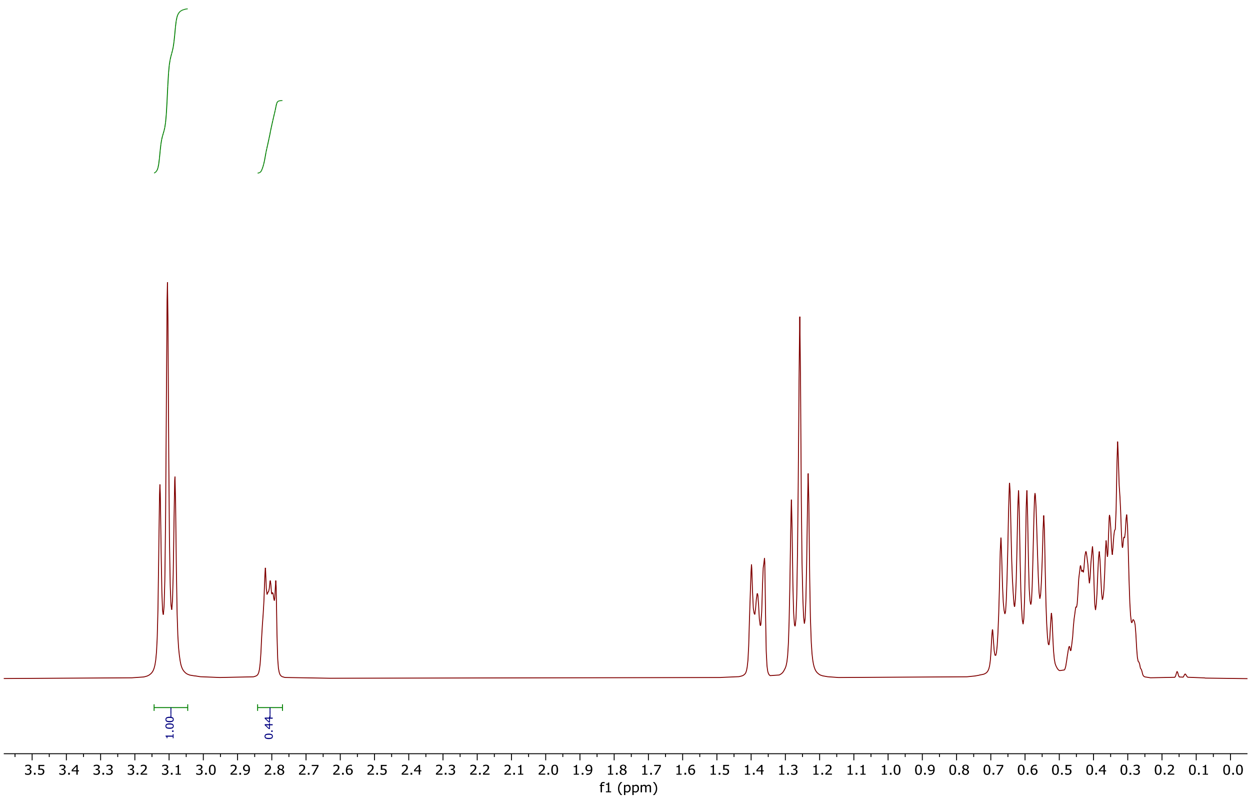


**Figure S69.** $^{1}H$ NMR (300 MHz, $C_6H_6$) of ε-caprolactone polymerization in $C_6H_6$. Chemical shifts are uncorrected; peak further downfield corresponds to polycaprolactone while further upfield corresponds to the monomer. Conditions: **3c** catalyst, 0.1 mol % loading, benzene, 80 $^{o}C$, 15 hours.

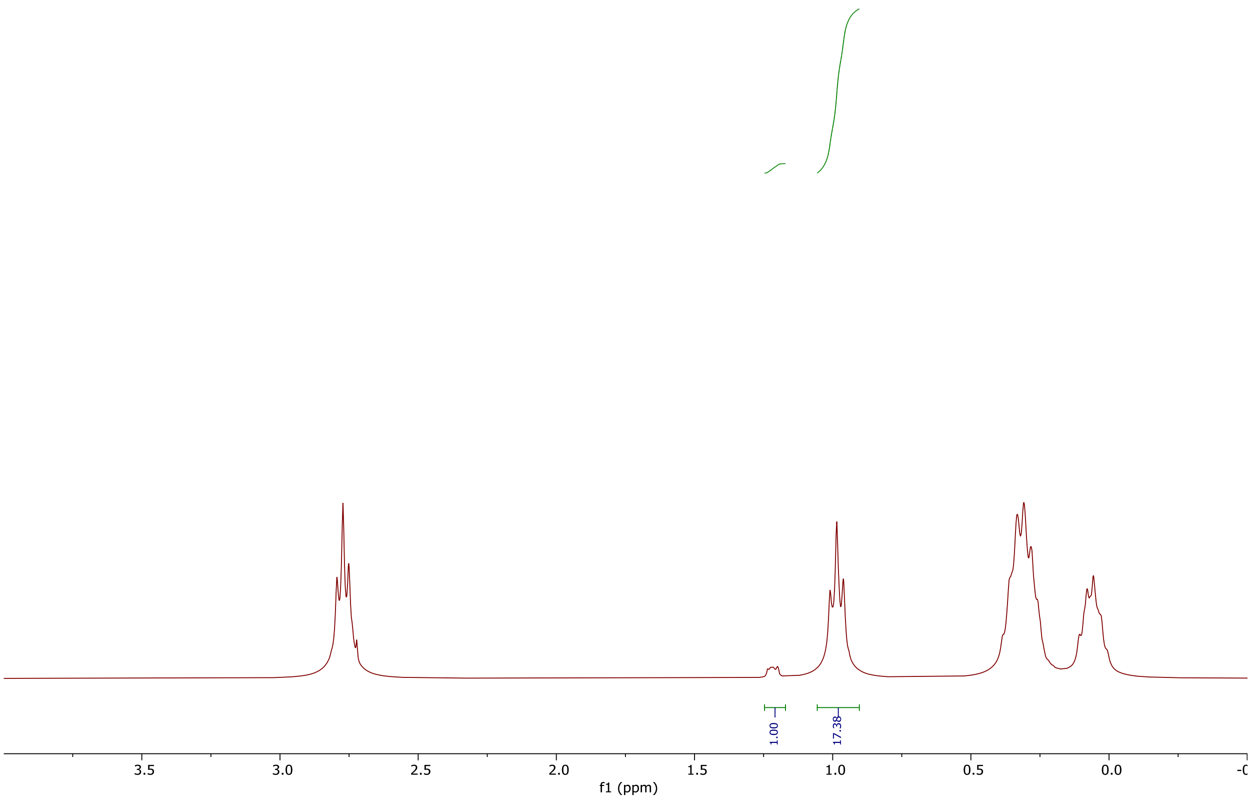


**Figure S70.** $^{1}H$ NMR (300 MHz, $C_6H_6$) of ε-caprolactone polymerization in *o*-DCB. Chemical shifts are uncorrected; peak further upfield corresponds to polycaprolactone while further downfield corresponds to the monomer (a different reference peak was chosen for this experiment due to better peak separation in *o*-DCB). Conditions: **3b** catalyst, 0.02 mol % loading, *ortho*-dichlorobenzene, 120 °C, 6 hours.

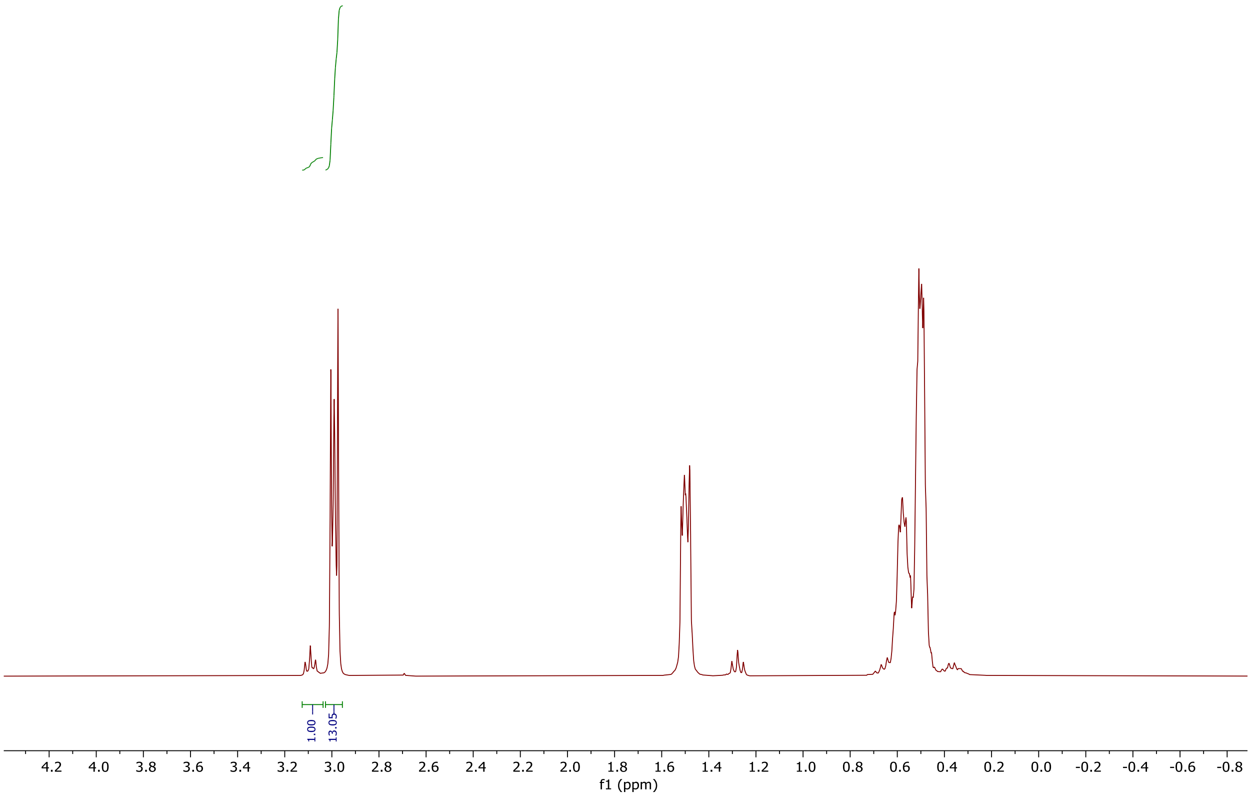


**Figure S71.** $^1$H NMR (300 MHz, $C_6H_6$) of ε-caprolactone polymerization in $C_6H_6$. Chemical shifts are uncorrected; peak further downfield corresponds to polycaprolactone while further upfield corresponds to the monomer. Conditions: **3d** catalyst, 0.1 mol % loading, benzene, 80 °C, 16 hours.

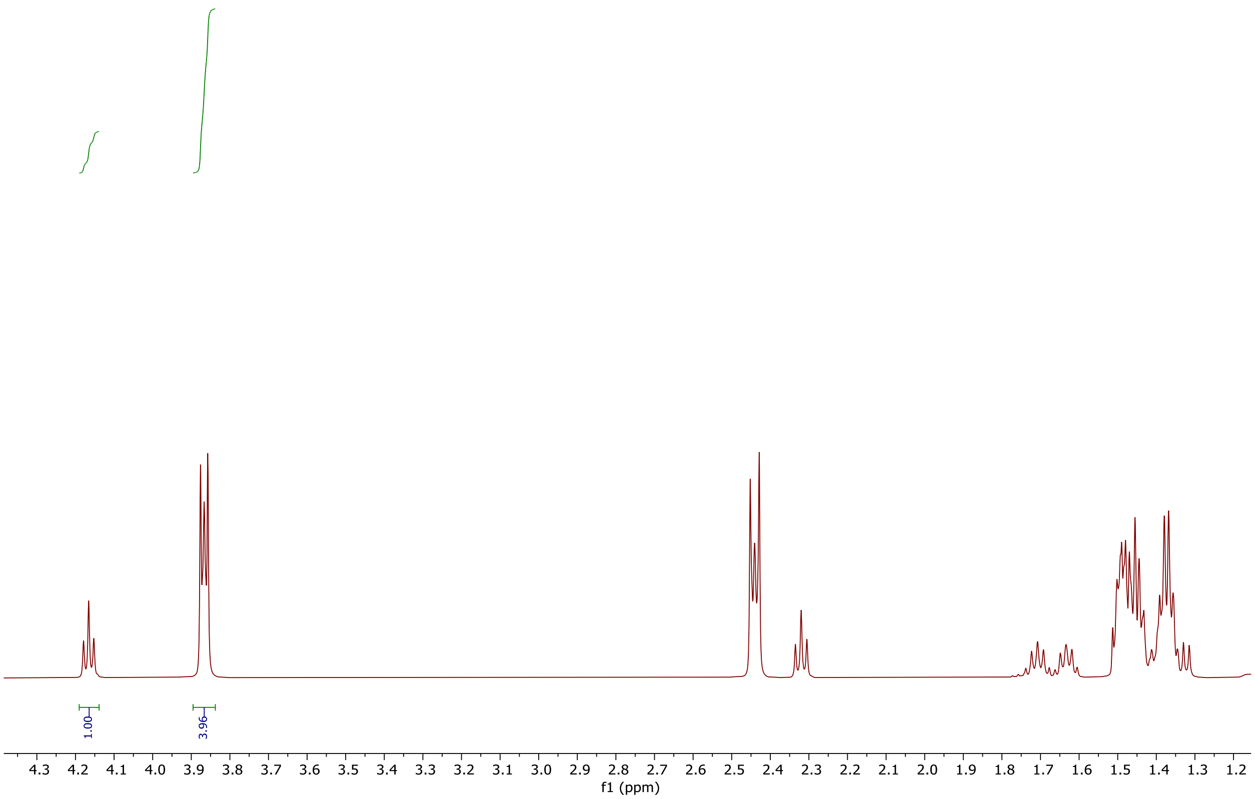


**Figure S72.** $^1$H NMR (300 MHz, $C_6H_6$) of ε-caprolactone polymerization in $C_6H_6$. Chemical shifts are uncorrected; peak further downfield corresponds to polycaprolactone while further upfield corresponds to the monomer. Conditions: **3b** catalyst, 1 mol % loading, benzene, 80 $^o$C, 16 hours.

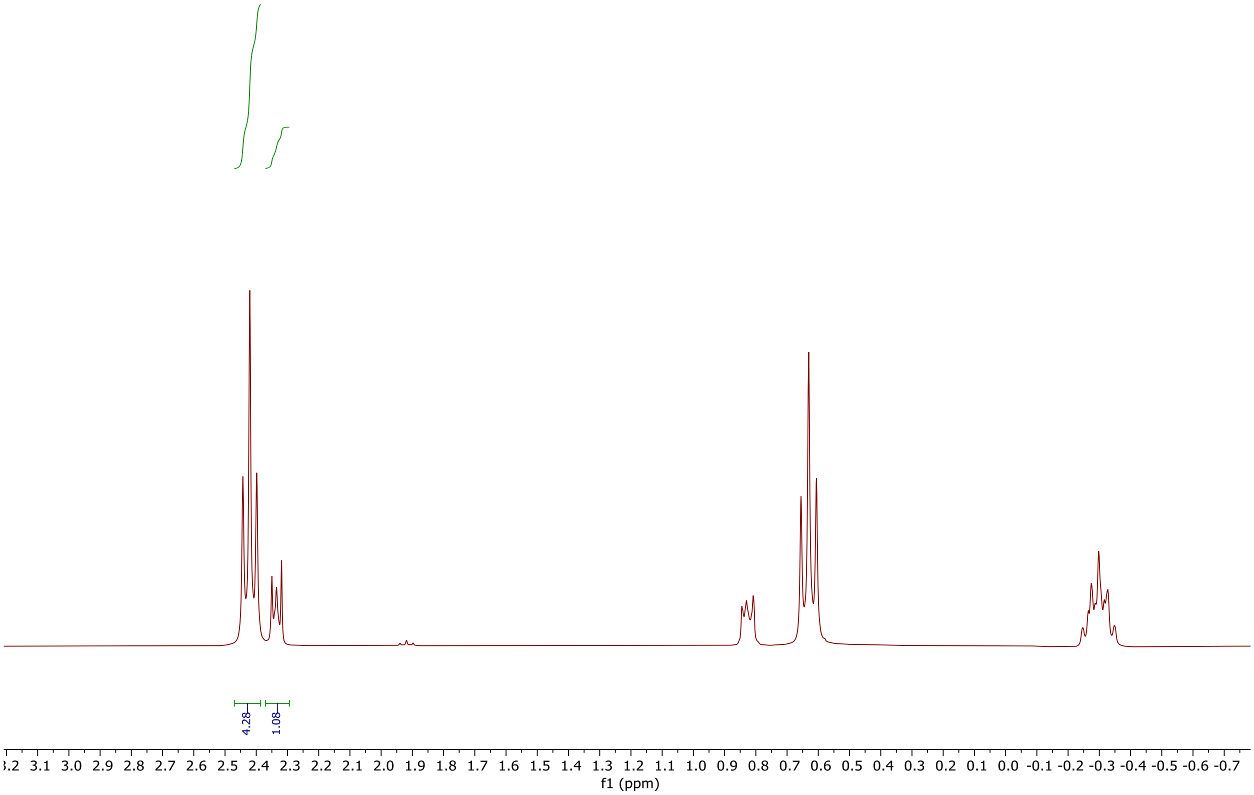


**Figure S73.** $^{1}H$ NMR (300 MHz, $C_6H_6$) of ε-caprolactone polymerization in $C_6H_6$. Chemical shifts are uncorrected; peak further downfield corresponds to polycaprolactone while further upfield corresponds to the monomer. Conditions: **3b** catalyst, 100:1 monomer:I, benzene, 80 °C, 40 hours.

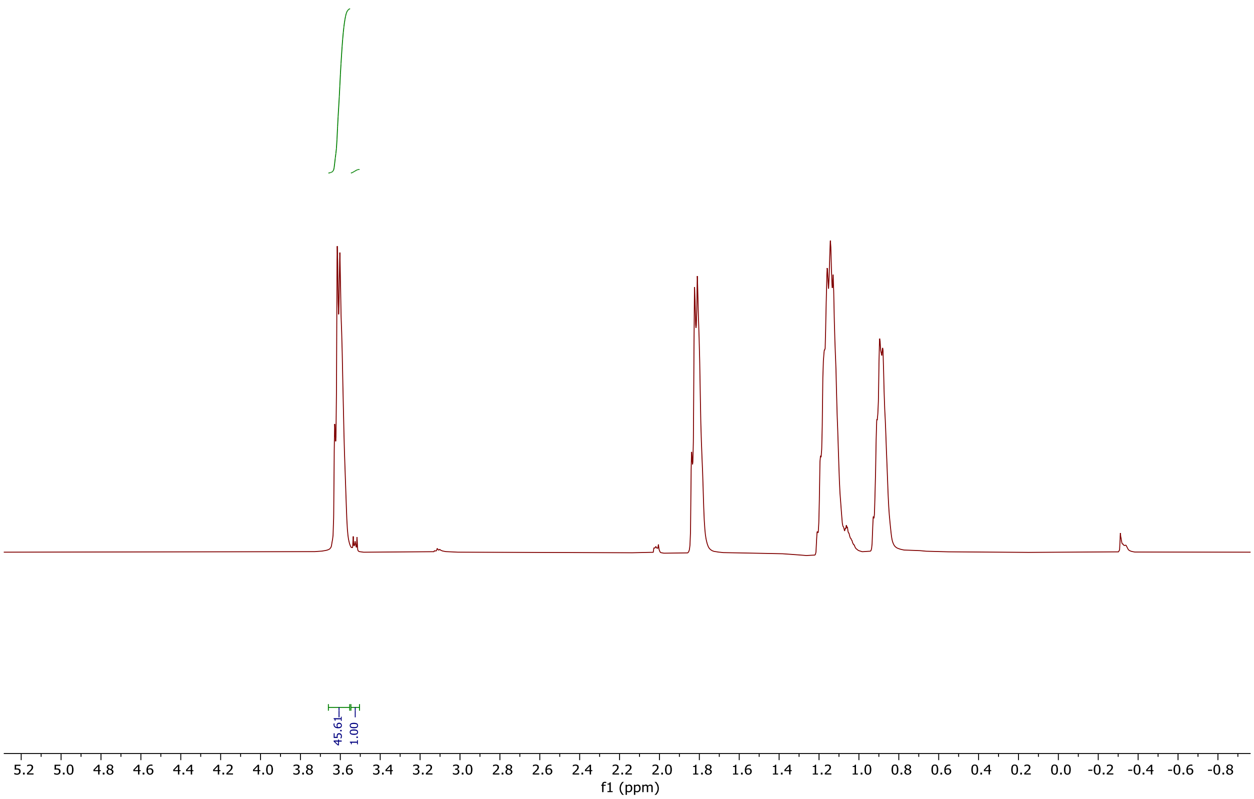


**Figure S74.** $^{1}H$ NMR (300 MHz, $C_6H_6$) of ε-caprolactone polymerization in $C_6H_6$. Chemical shifts are uncorrected; peak further downfield corresponds to polycaprolactone while further upfield corresponds to the monomer. Conditions: **3b** catalyst, 200:1 monomer:I, benzene, 80 °C, 16 hours.

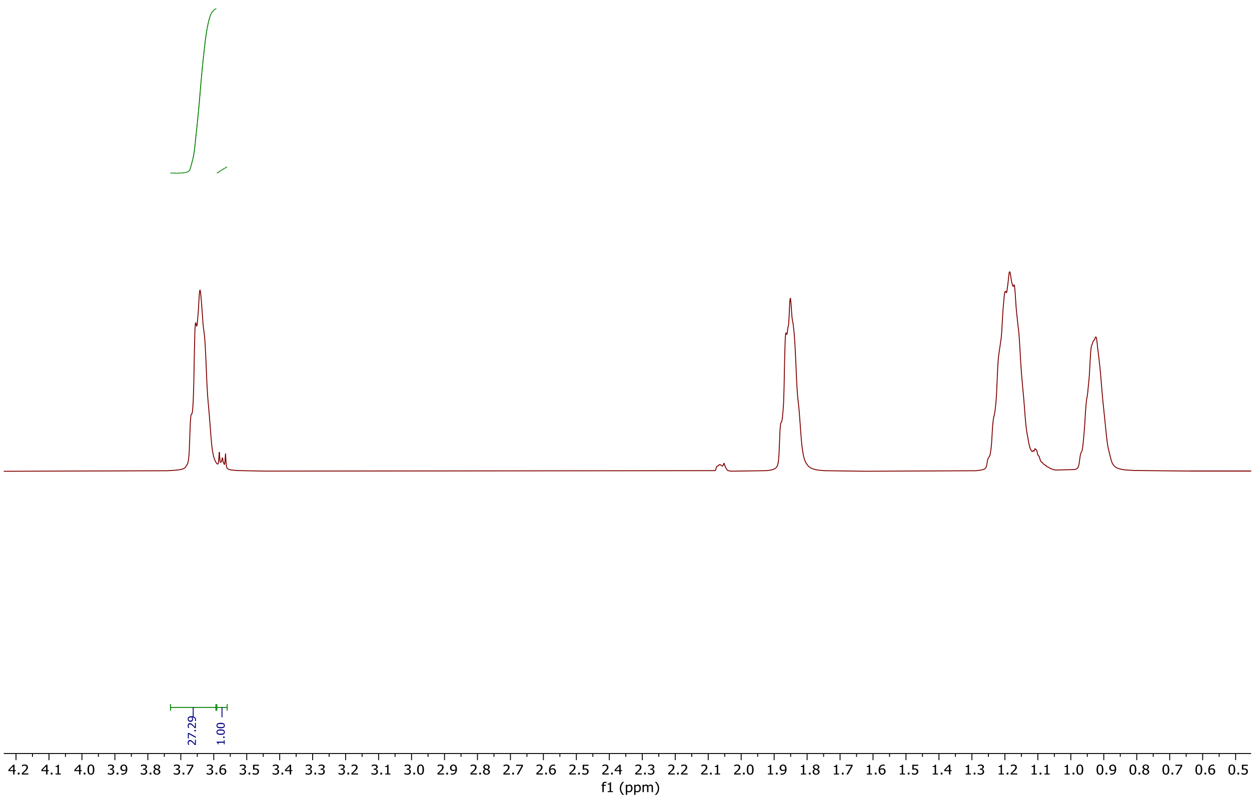


**Figure S75.** $^{1}H$ NMR (300 MHz, $C_6H_6$) of ε-caprolactone polymerization in $C_6H_6$. Chemical shifts are uncorrected; peak further downfield corresponds to polycaprolactone while further upfield corresponds to the monomer. Conditions: **3b** catalyst, 500:1 monomer:I, benzene, 80 $^{o}C$, 16 hours.

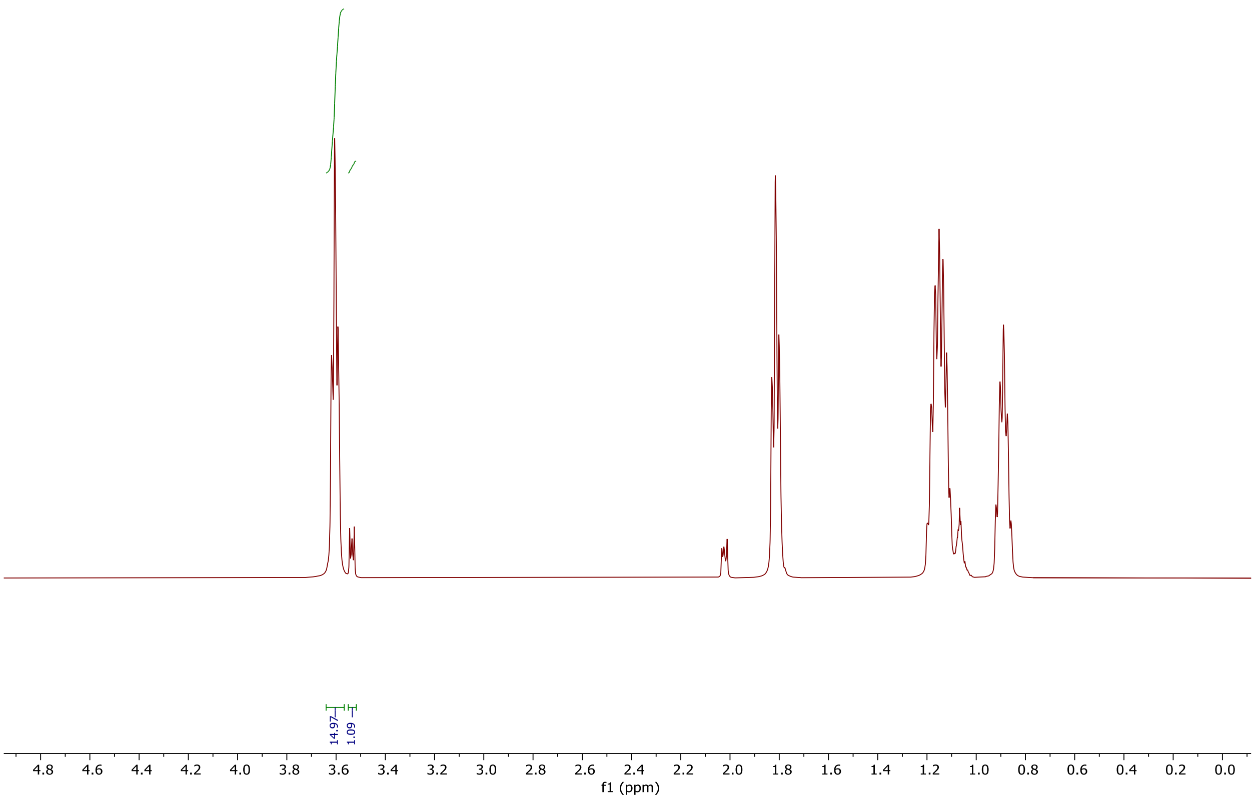


**Figure S76.** $^{1}$H NMR (300 MHz, $C_6H_6$) of ε-caprolactone polymerization in $C_6H_6$. Chemical shifts are uncorrected; peak further downfield corresponds to polycaprolactone while further upfield corresponds to the monomer. Conditions: **3b** catalyst, 1000:1 monomer:I, benzene, 80 $^{o}$C, 16 hours.

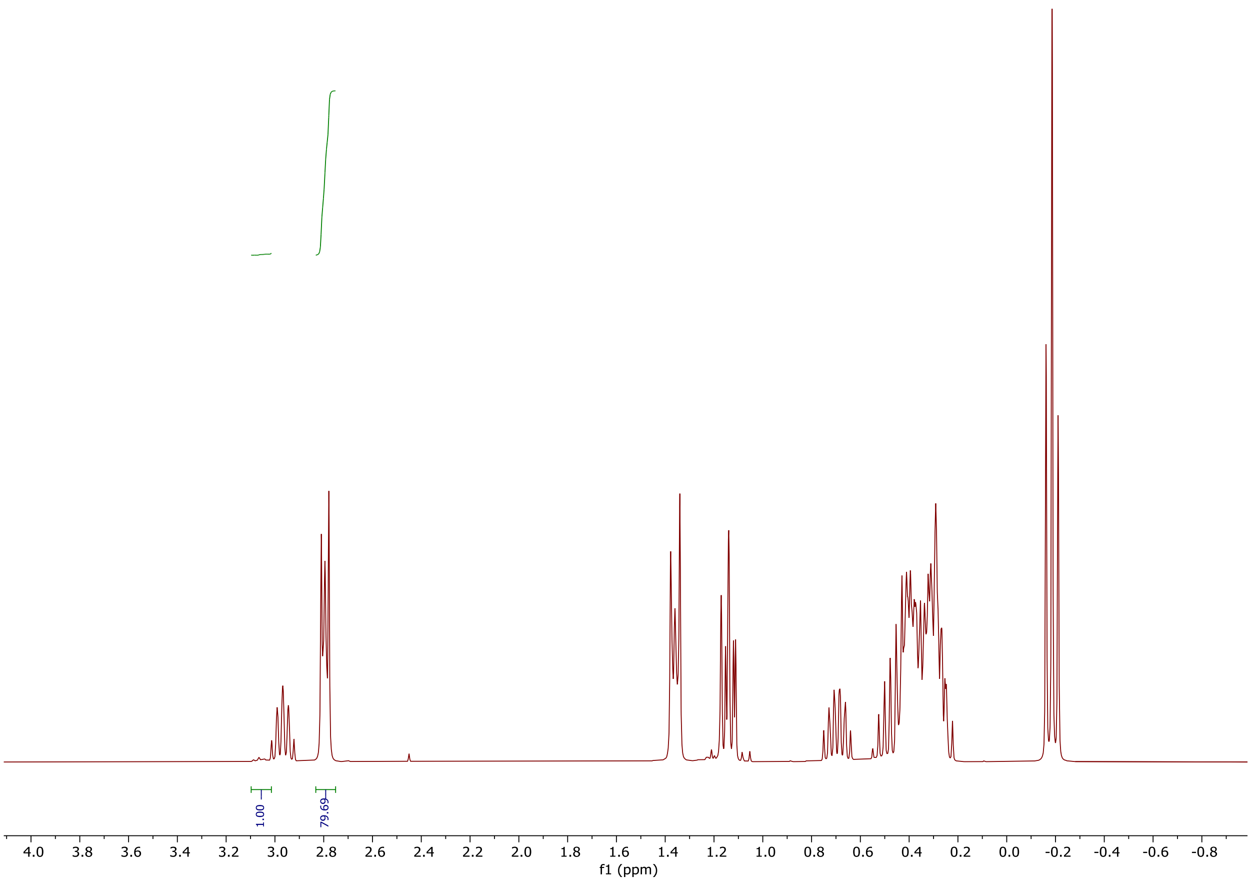


**Figure S77.** $^1$H NMR (300 MHz, $C_6H_6$) of ε-caprolactone polymerization in $C_6H_6$. Chemical shifts are uncorrected; peak further downfield corresponds to polycaprolactone while further upfield corresponds to the monomer. One equivalent of γ-caprolactone was added to compete for coordination. Note dramatically reduced TON (62 vs 4000 without γ-caprolactone). Conditions: **3b** catalyst, 0.02 mol % loading, benzene, 80 °C, 20 hours.

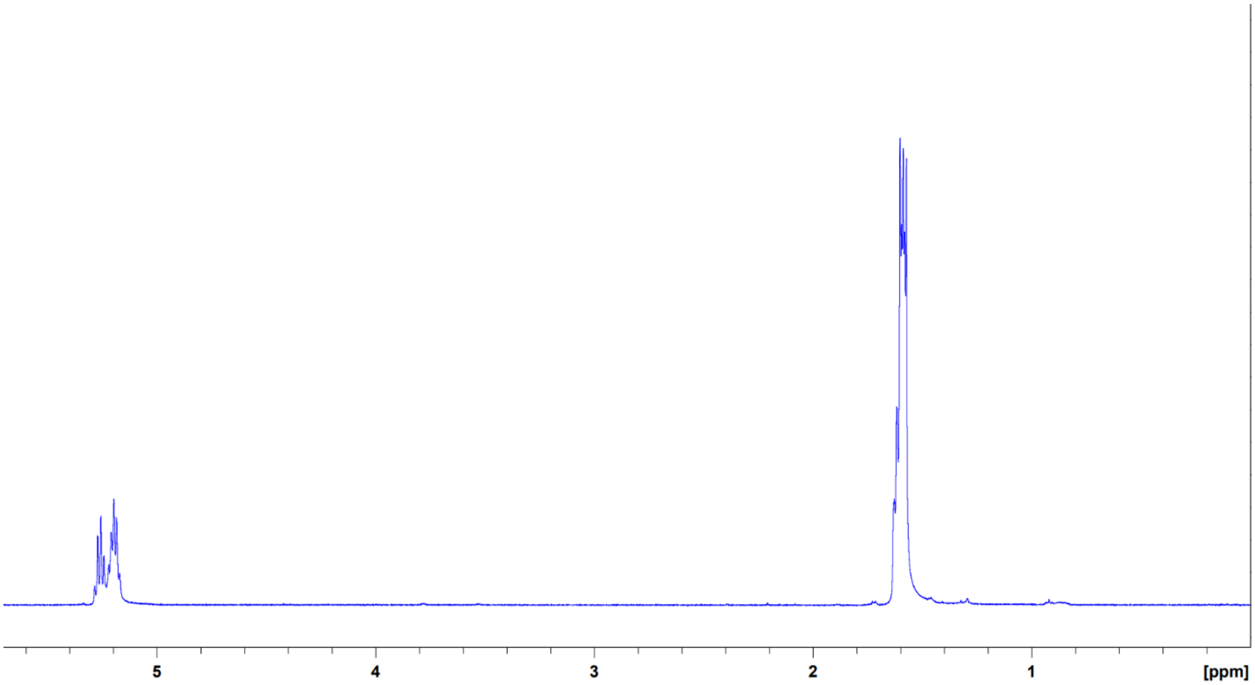


**Figure S78.** $^{1}H$ NMR (300 MHz, $C_6H_6$) of *rac*-lactide polymerization in $C_6H_6$. Chemical shifts are uncorrected; spectra of isolated polylactide. Conditions: **3b** catalyst, 0.05 mol % loading, neat, 120 $^{o}C$, 18 hours.

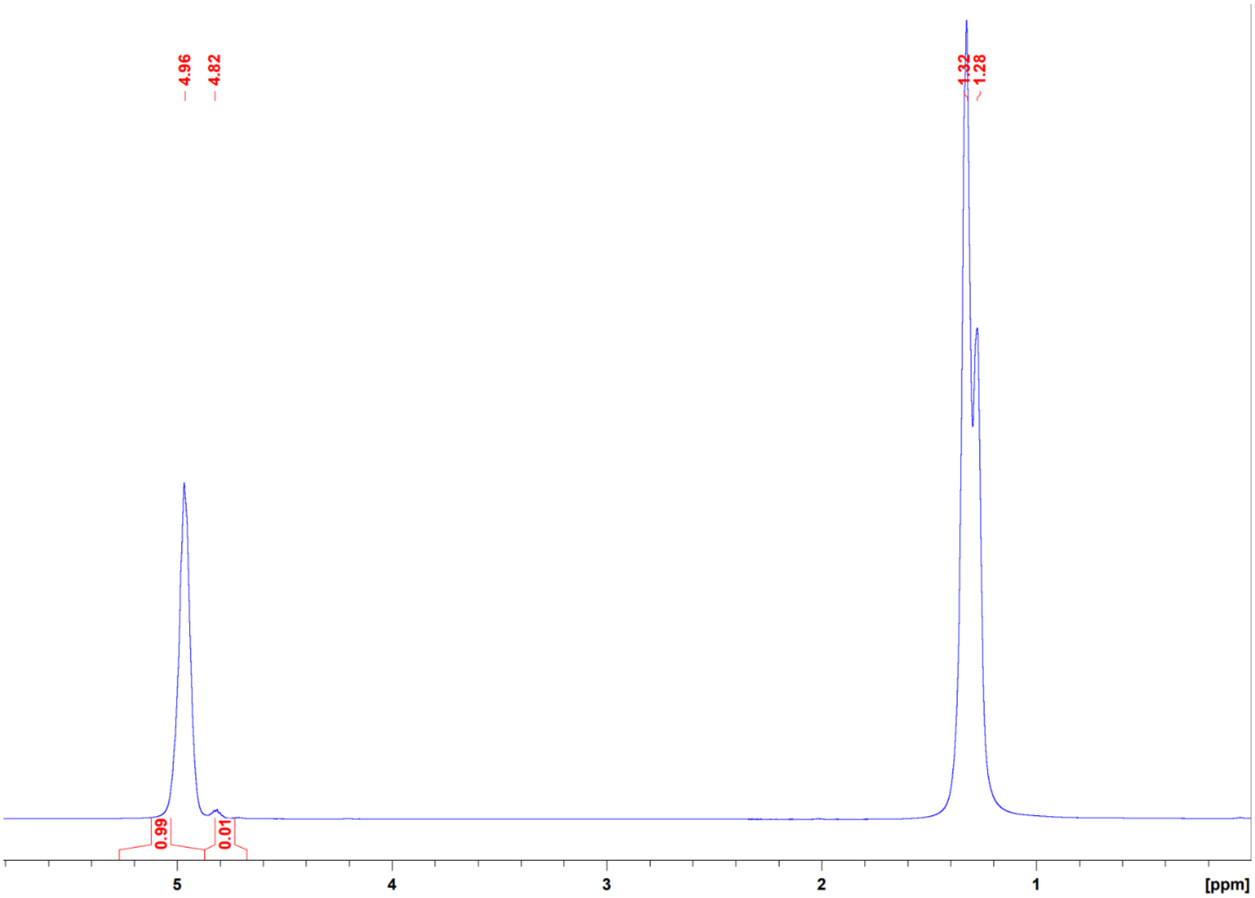


**Figure S79.** $^{1}$H NMR (300 MHz, $C_6H_6$) of *rac*-lactide polymerization in o-DCB. Chemical shifts are uncorrected; peak further downfield corresponds to polylactic acid, while peak further upfield corresponds to lactide. Conditions: **3b** catalyst, 0.1 mol % loading, o-DCB, 80 °C, 12 hours.

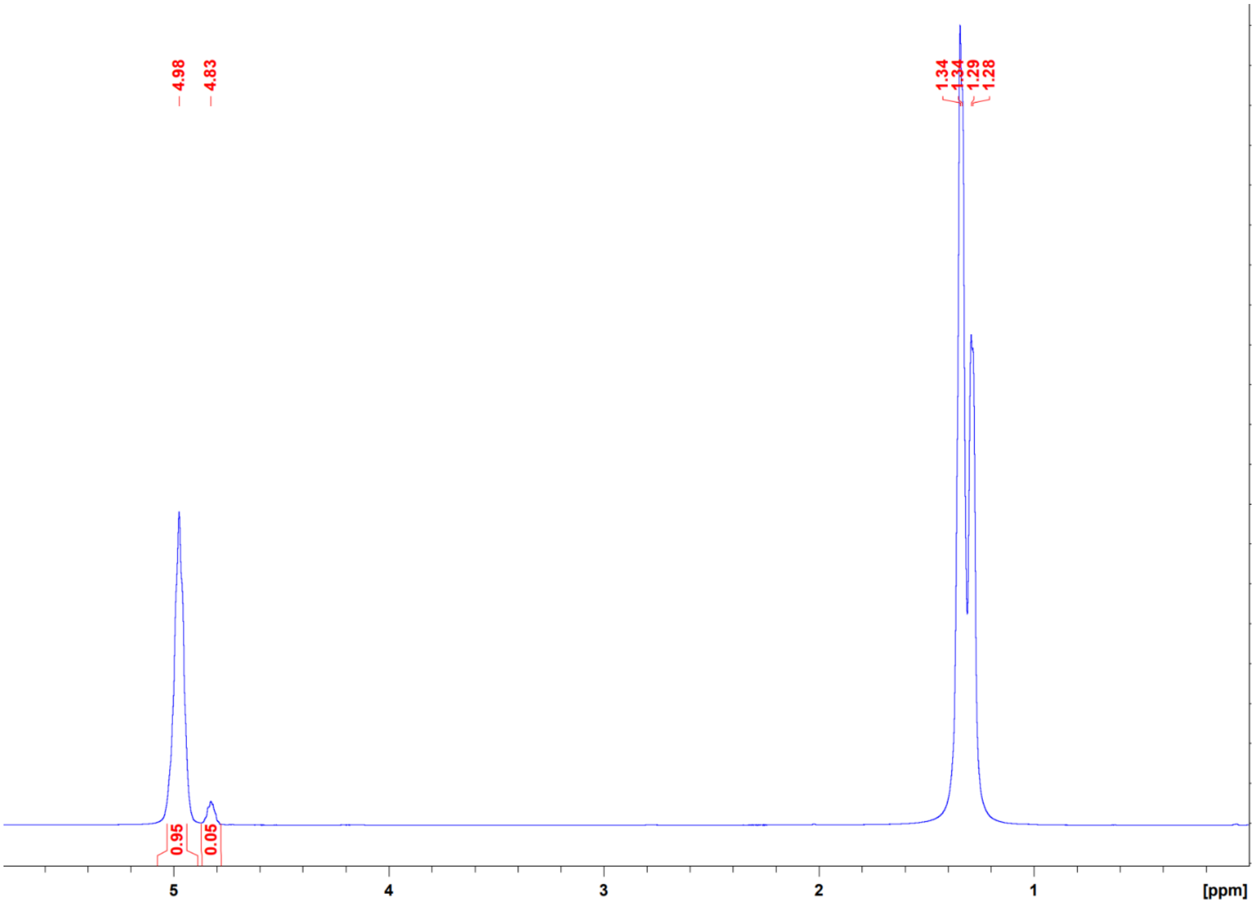


**Figure S80.** $^{1}$H NMR (300 MHz, $C_6H_6$) of *rac*-lactide polymerization in o-DCB. Chemical shifts are uncorrected; peak further downfield corresponds to polylactic acid, while peak further upfield corresponds to lactide. Conditions: **3b** catalyst, 0.05 mol % loading, o-DCB, 80 °C, 12 hours.

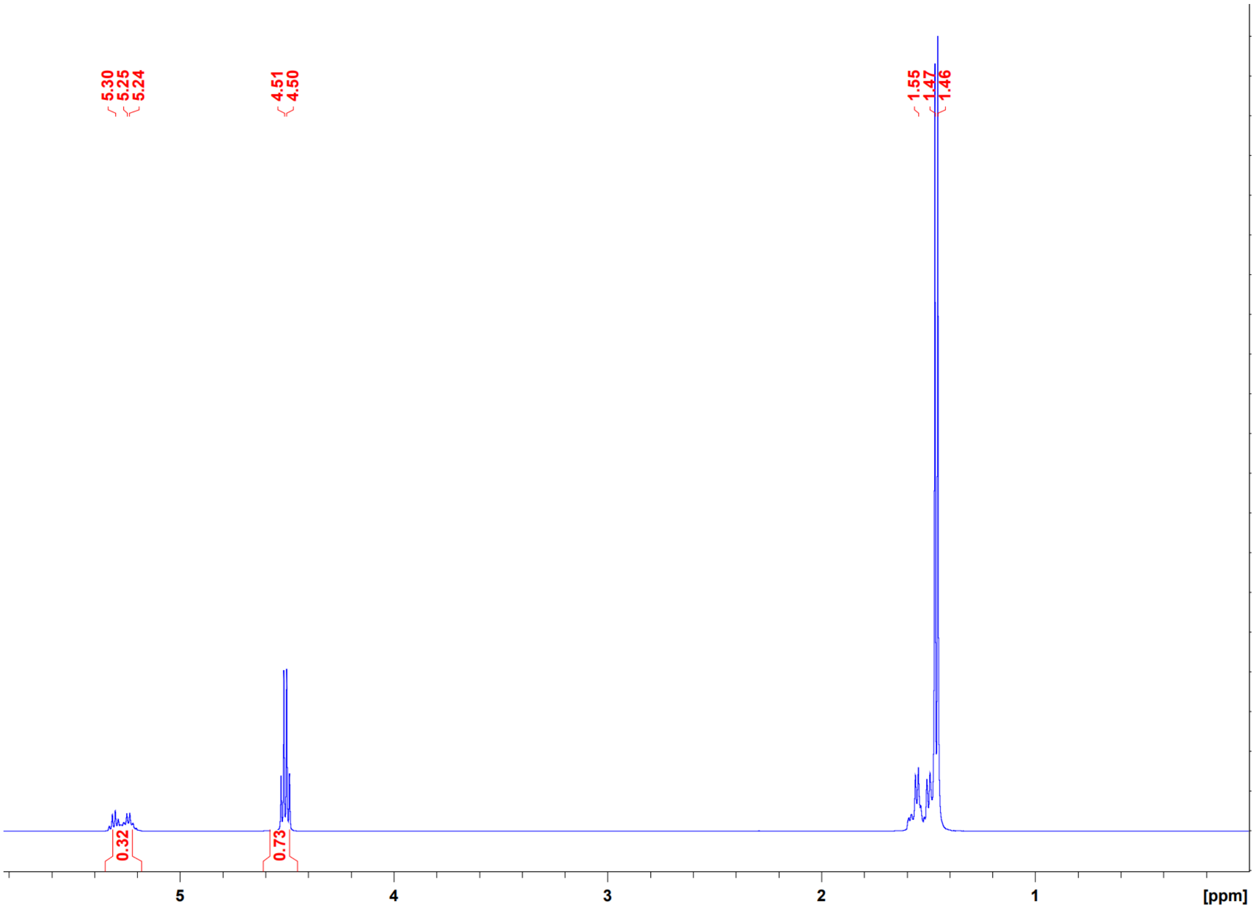


**Figure S81.** $^{1}H$ NMR (300 MHz, $C_6H_6$) of *rac*-lactide polymerization in $C_6H_6$. Chemical shifts are uncorrected; peak further downfield corresponds to polylactic acid, while peak further upfield corresponds to lactide. Conditions: **3b** catalyst, 0.02 mol % loading, $C_6H_6$, 80 $^{o}C$, 16 hours.

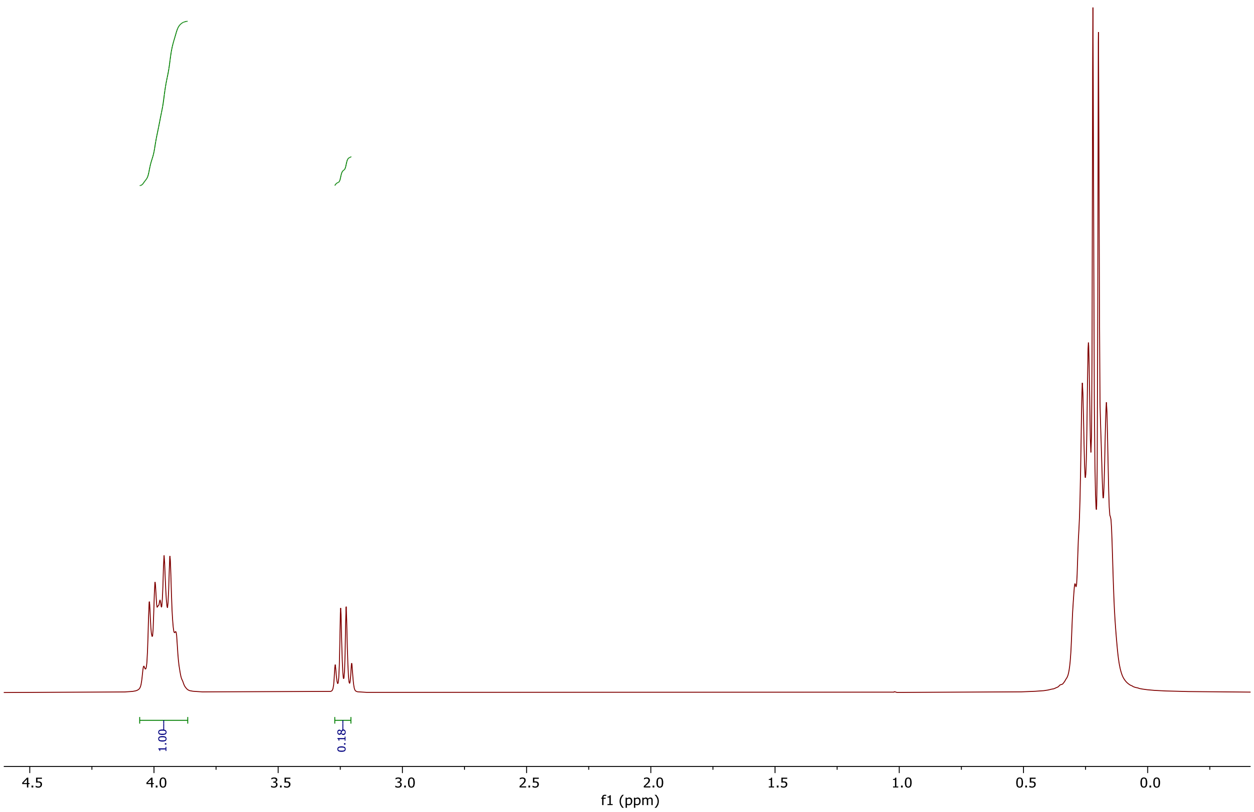


**Figure S82.** $^{1}$H NMR (300 MHz, $C_6H_6$) of *rac*-lactide polymerization in $C_6H_6$. Chemical shifts are uncorrected; peak further downfield corresponds to polylactic acid, while peak further upfield corresponds to lactide. Conditions: **3b** catalyst, 0.05 mol % loading, $C_6H_6$, 80 °C, 40 hours.

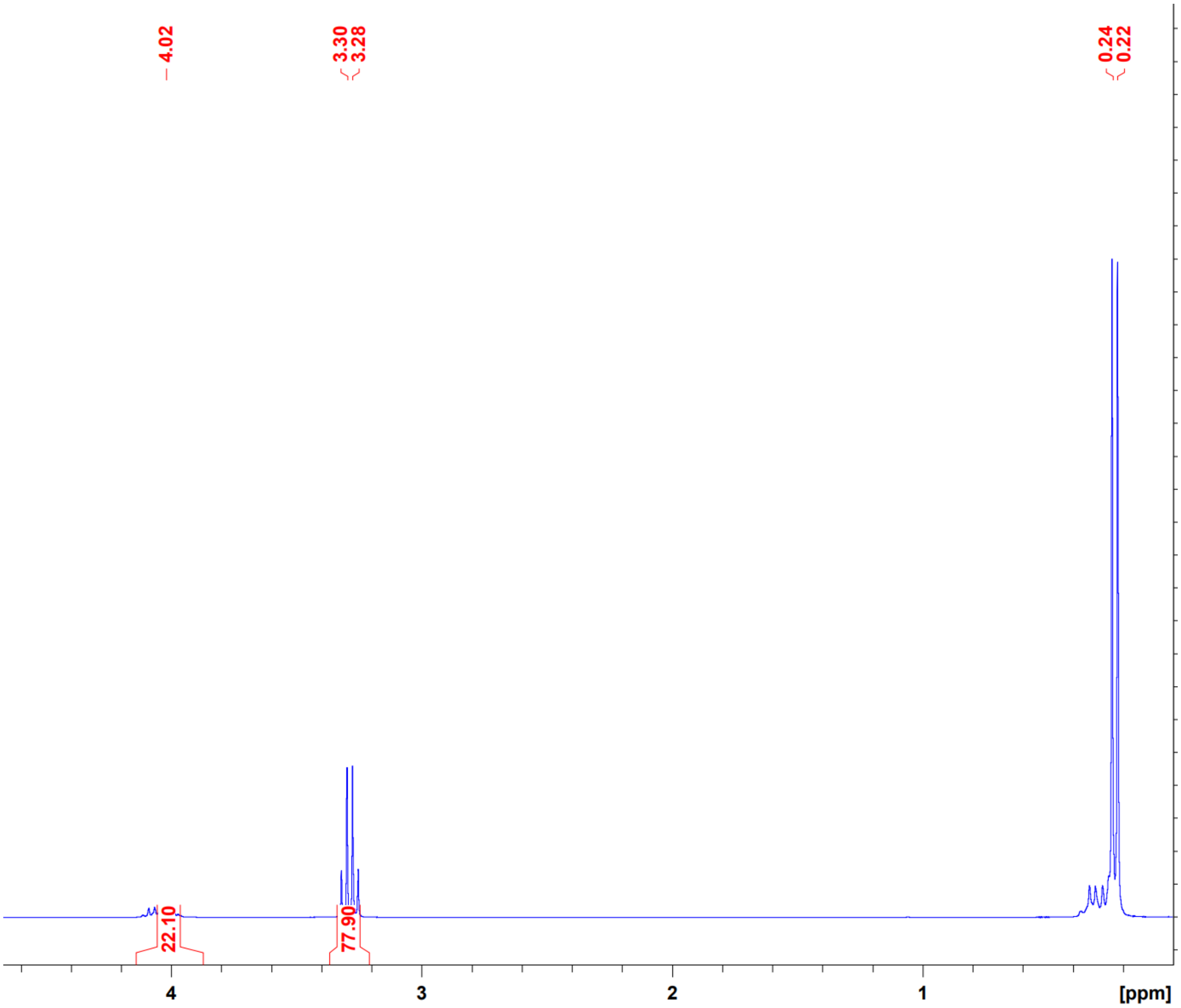


**Figure S83.** $^{1}H$ NMR (300 MHz, $C_6H_6$) of *rac*-lactide polymerization in $C_6H_6$. Chemical shifts are uncorrected; peak further downfield corresponds to polylactic acid, while peak further upfield corresponds to lactide. Conditions: **3d** catalyst, 0.05 mol % loading, $C_6H_6$, 80 °C, 40 hours.

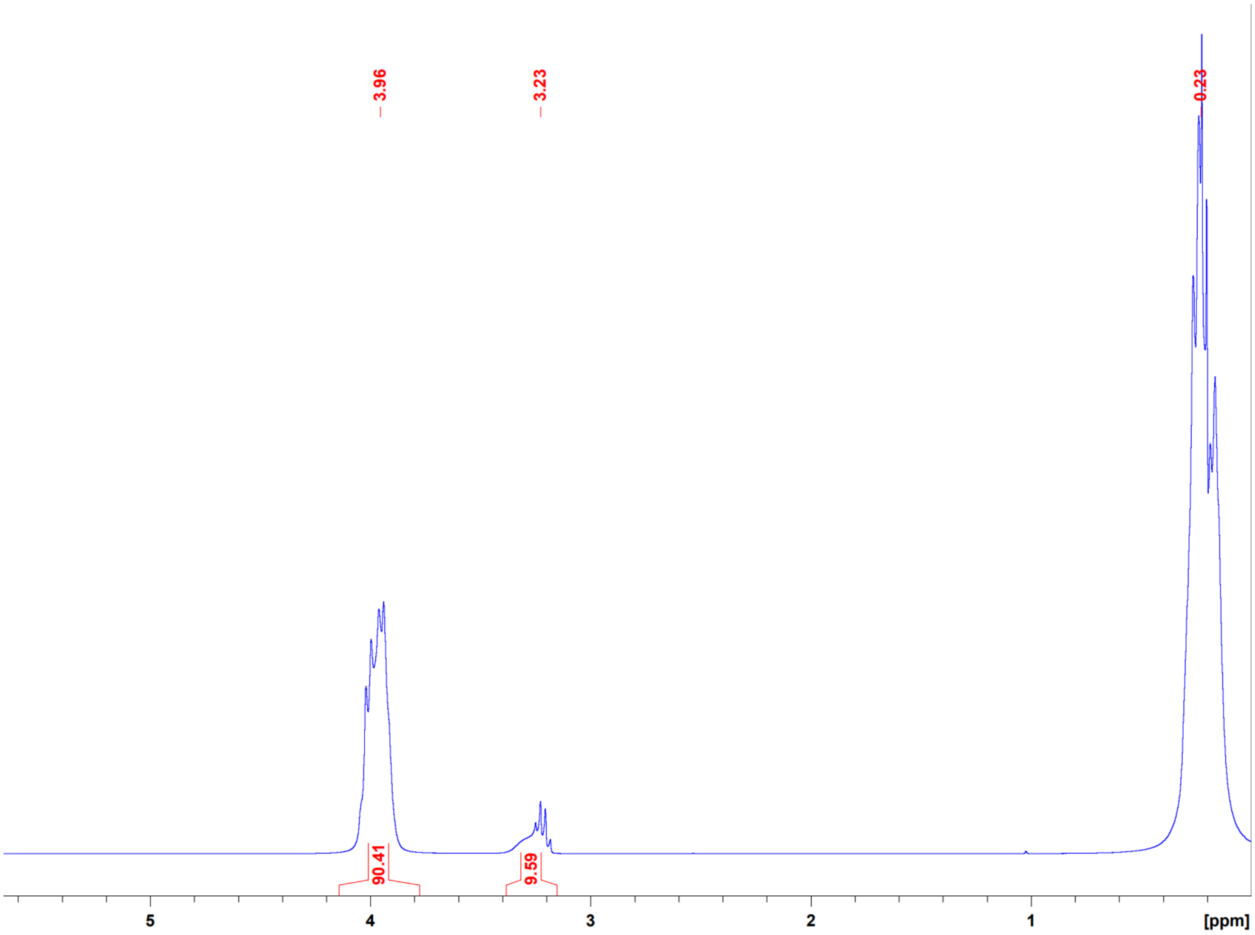


**Figure S84.** $^{1}H$ NMR (300 MHz, $C_6H_6$) of *rac*-lactide polymerization in $C_6H_6$. Chemical shifts are uncorrected; peak further downfield corresponds to polylactic acid, while peak further upfield corresponds to lactide. Conditions: **3c** catalyst, 0.05 mol % loading, $C_6H_6$, 80 $^{o}C$, 40 hours.

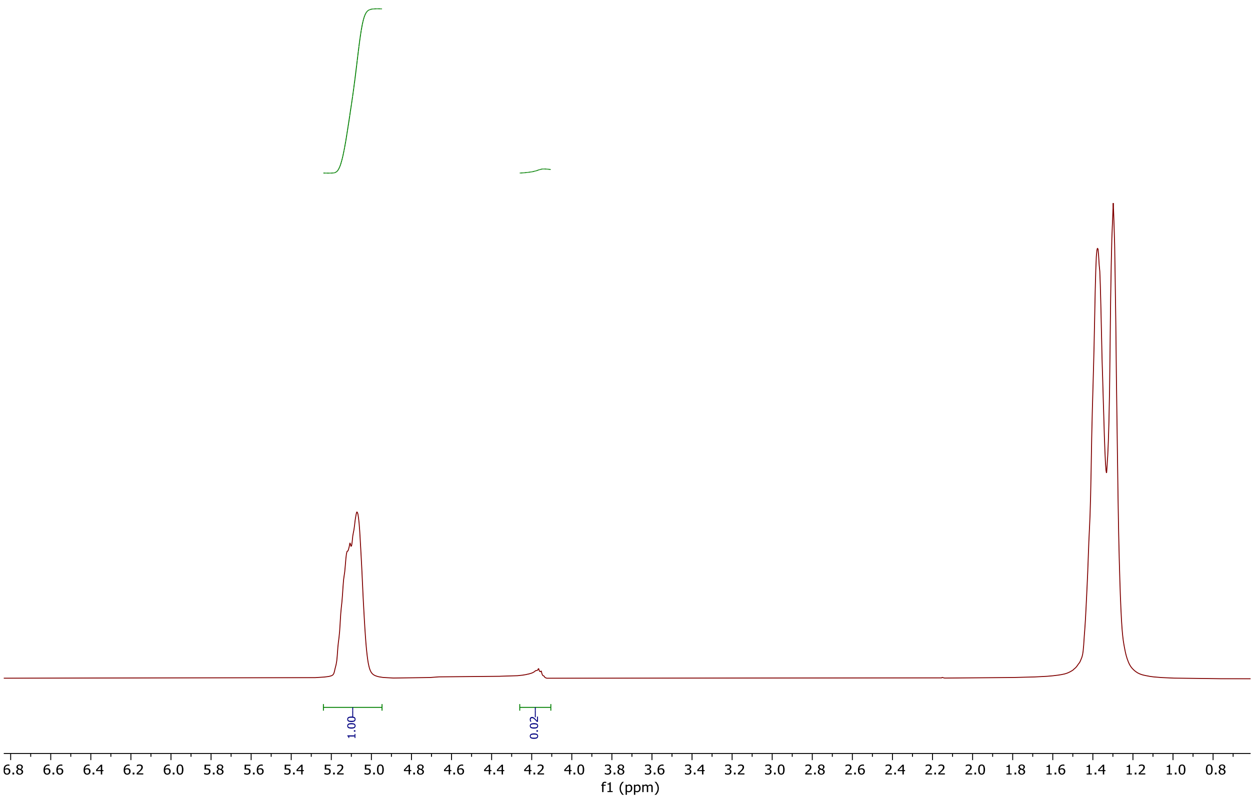


**Figure S85.** $^{1}$H NMR (300 MHz, $C_6H_6$) of *rac*-lactide polymerization in $C_6H_6$. Chemical shifts are uncorrected; peak further downfield corresponds to polylactic acid, while peak further upfield corresponds to lactide. Conditions: **3a** catalyst, 0.05 mol % loading, $C_6H_6$, 80 °C, 40 hours.

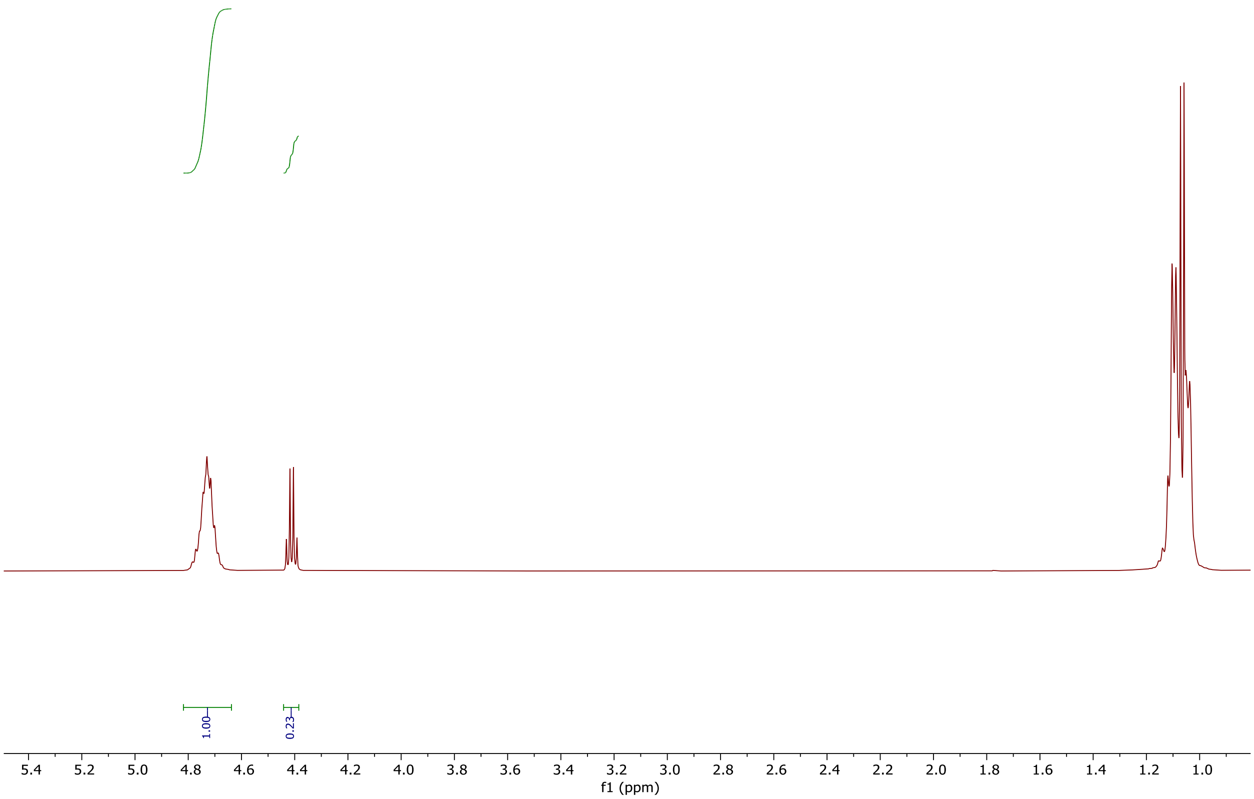


**Figure S86.** $^{1}H$ NMR (300 MHz, $C_6H_6$) of *rac*-lactide polymerization in $C_6H_6$. Chemical shifts are uncorrected; peak further downfield corresponds to polylactic acid, while peak further upfield corresponds to lactide. Conditions: **3a** catalyst, 100:1 monomer:I, $C_6H_6$, 80 $^{o}C$, 16 hours.

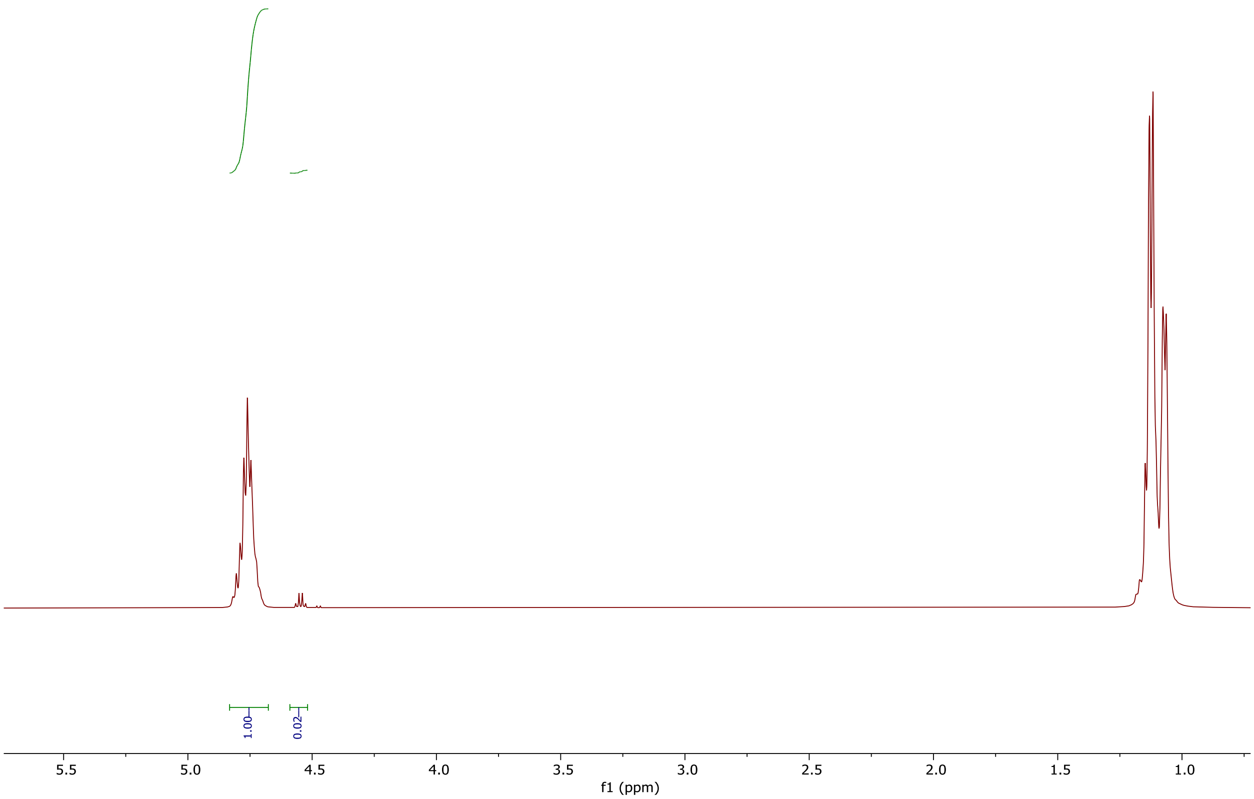


**Figure S87.** $^{1}H$ NMR (300 MHz, $C_6H_6$) of *rac*-lactide polymerization in $C_6H_6$. Chemical shifts are uncorrected; peak further downfield corresponds to polylactic acid, while peak further upfield corresponds to lactide. Conditions: **3a** catalyst, 200:1 monomer:I, $C_6H_6$, 80 $^{o}C$, 16 hours.

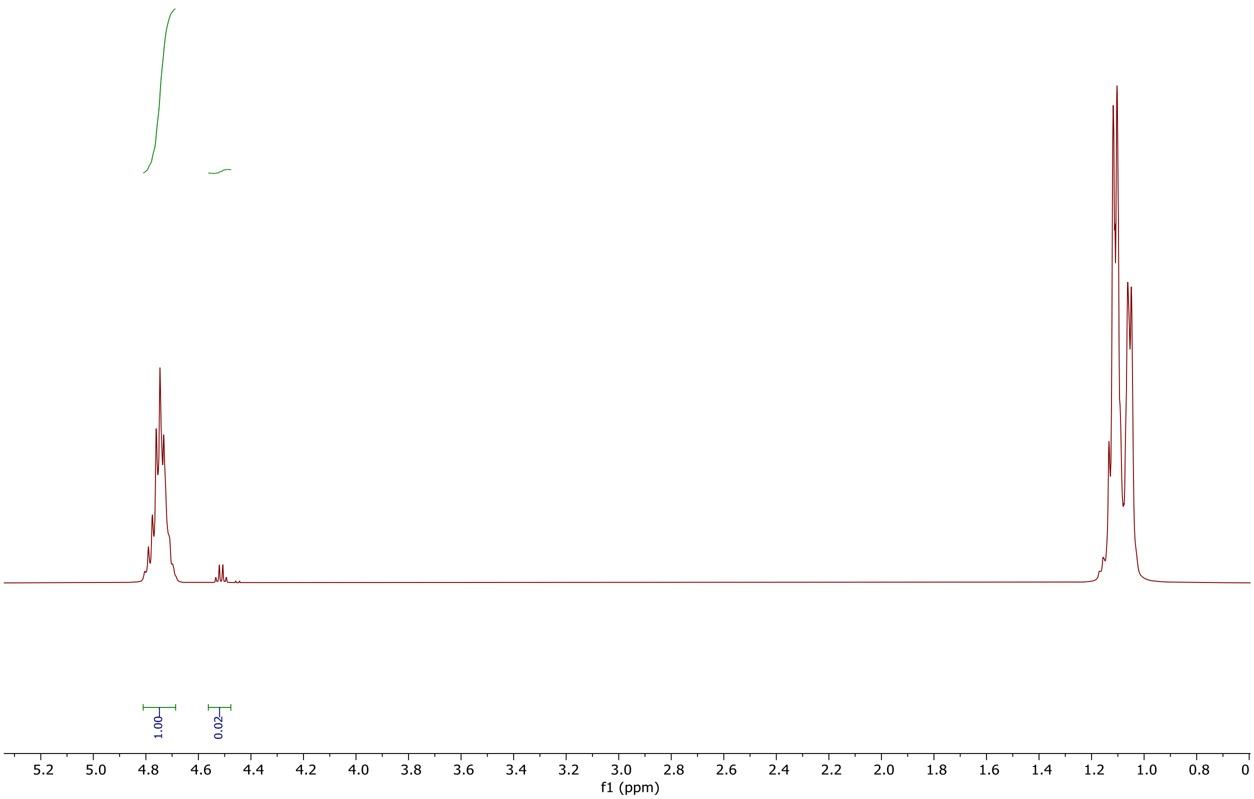


**Figure S88.** $^{1}H$ NMR (300 MHz, $C_6H_6$) of *rac*-lactide polymerization in $C_6H_6$. Chemical shifts are uncorrected; peak further downfield corresponds to polylactic acid, while peak further upfield corresponds to lactide. Conditions: **3a** catalyst, 500:1 monomer:I, $C_6H_6$, 80 $^{o}C$, 16 hours.

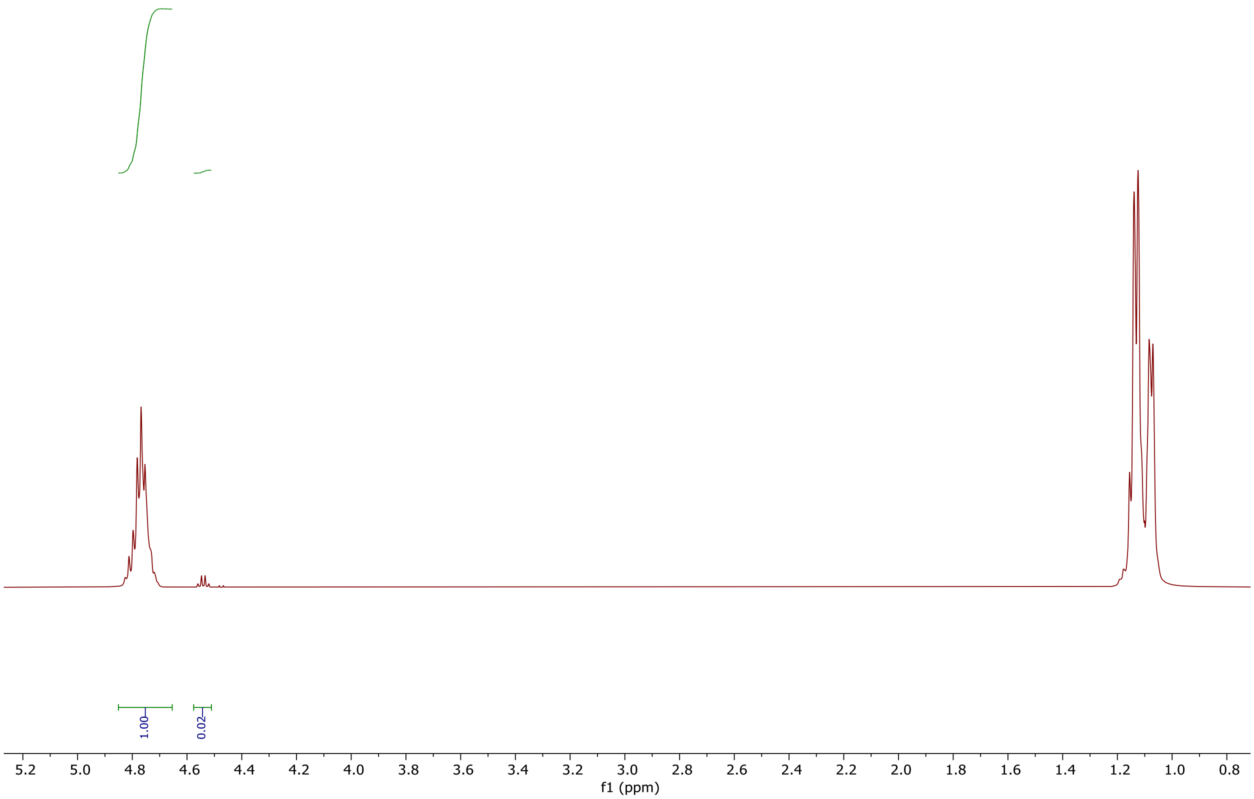


**Figure S89.** $^{1}$H NMR (300 MHz, $C_6H_6$) of *rac*-lactide polymerization in $C_6H_6$. Chemical shifts are uncorrected; peak further downfield corresponds to polylactic acid, while peak further upfield corresponds to lactide. Conditions: **3a** catalyst, 1000:1 monomer:I, $C_6H_6$, 80 °C, 16 hours.

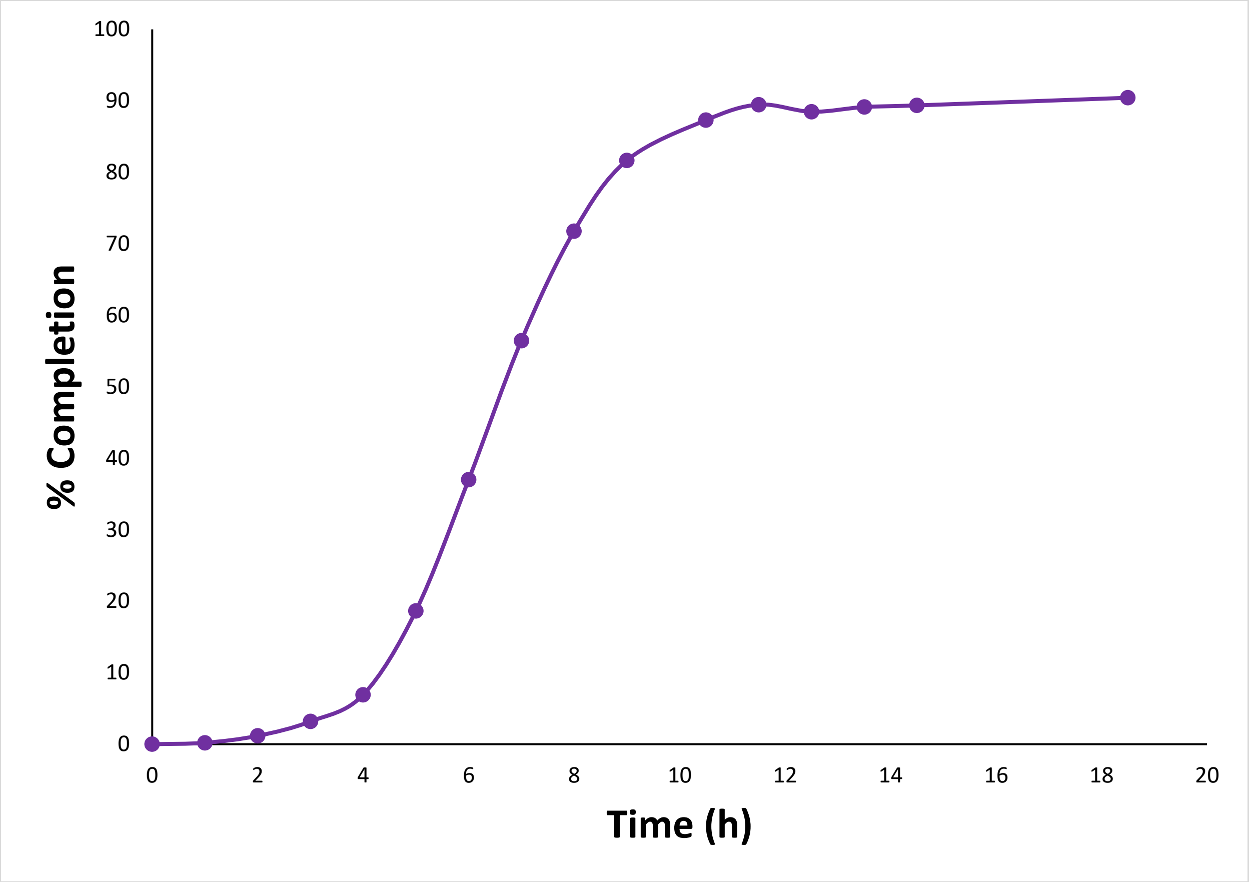


**Figure S90.** Kinetic study of polymerization completion versus time for ε-caprolactone polymerization. Percent completion was measured by integration in $^{1}$H NMR, recording spectra every hour when possible. Conditions: **3b** catalyst, 0.1 mol % loading, benzene, 80 $^{o}$C.

## Gel Permeation Chromatography Traces

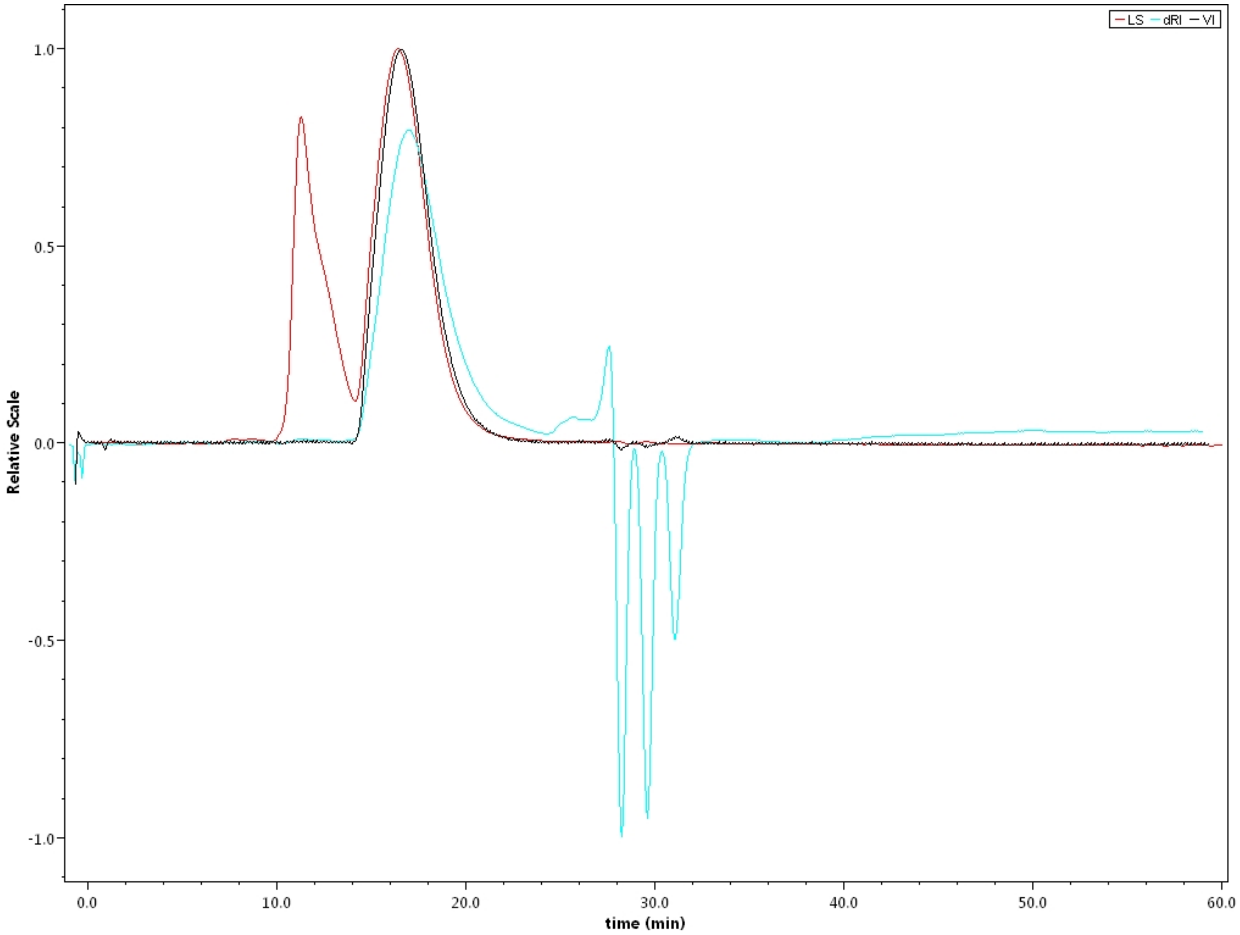


**Figure S91**: Gel permeation chromatogram (light scattering, refractive index, and viscometry traces) for polycaprolactone (Table 2, entry 1), $M_n$ = 62.85 kDa and $Đ$ = 1.31.

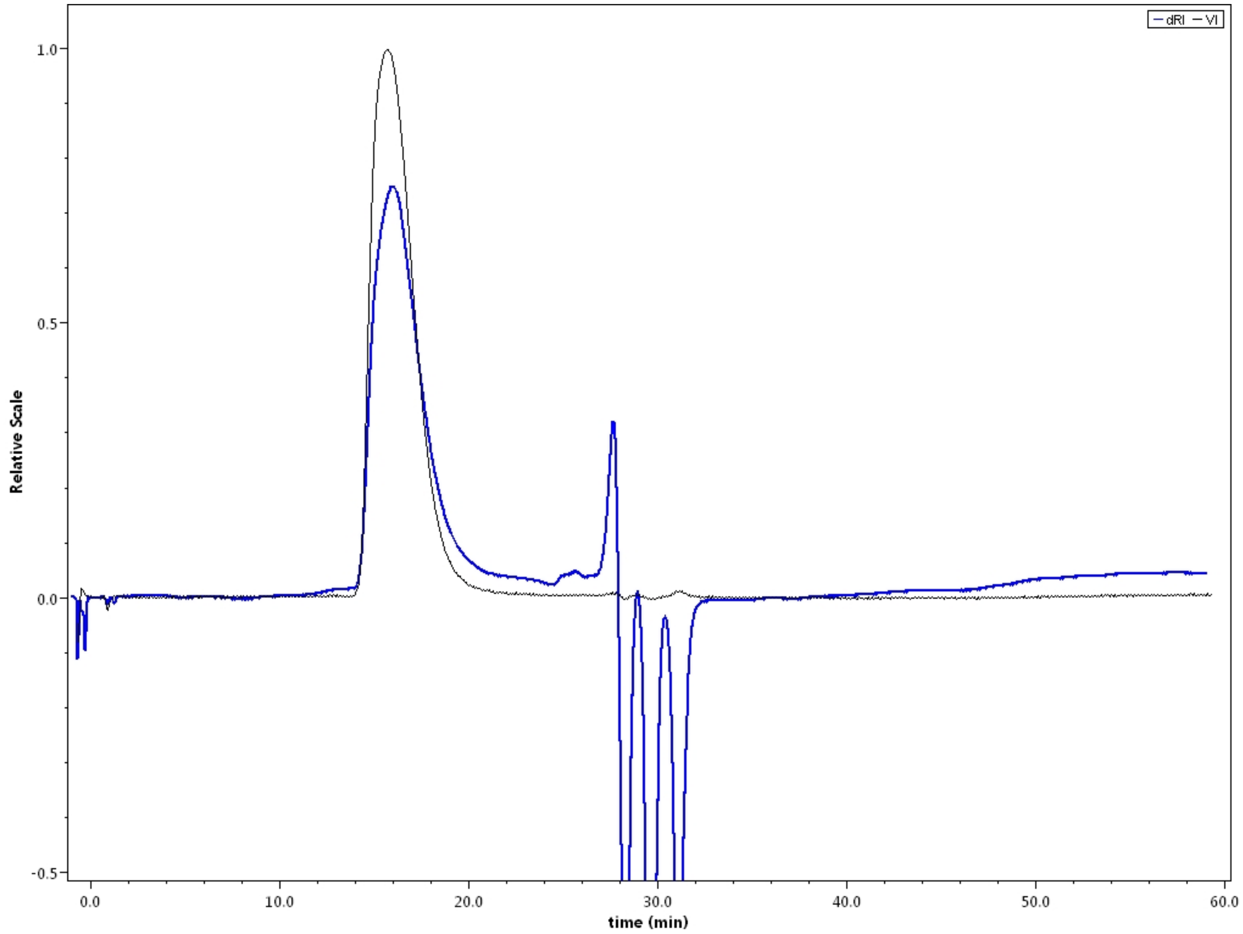


**Figure S92**: Gel permeation chromatogram (refractive index and viscometry traces) for polycaprolactone (Table 2, entry 2), $M_n$ = 232.6 kDa and $Đ$ = 1.15.

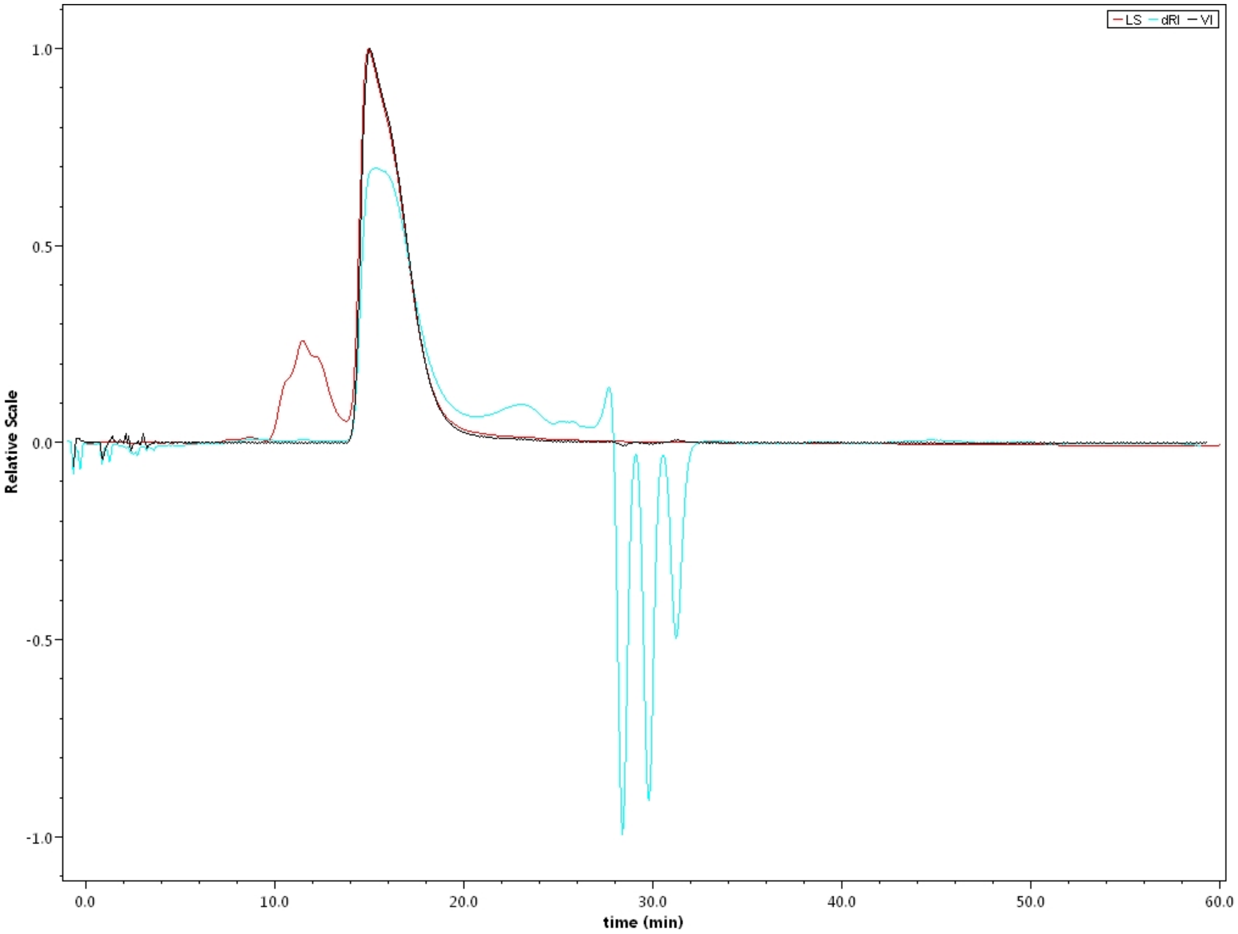


**Figure S93**: Gel permeation chromatogram (light scattering, refractive index, and viscometry traces) for polycaprolactone (Table 2, entry 3; Figure 8), $M_n$ of 289.6 kDa, *Đ* 1.09.

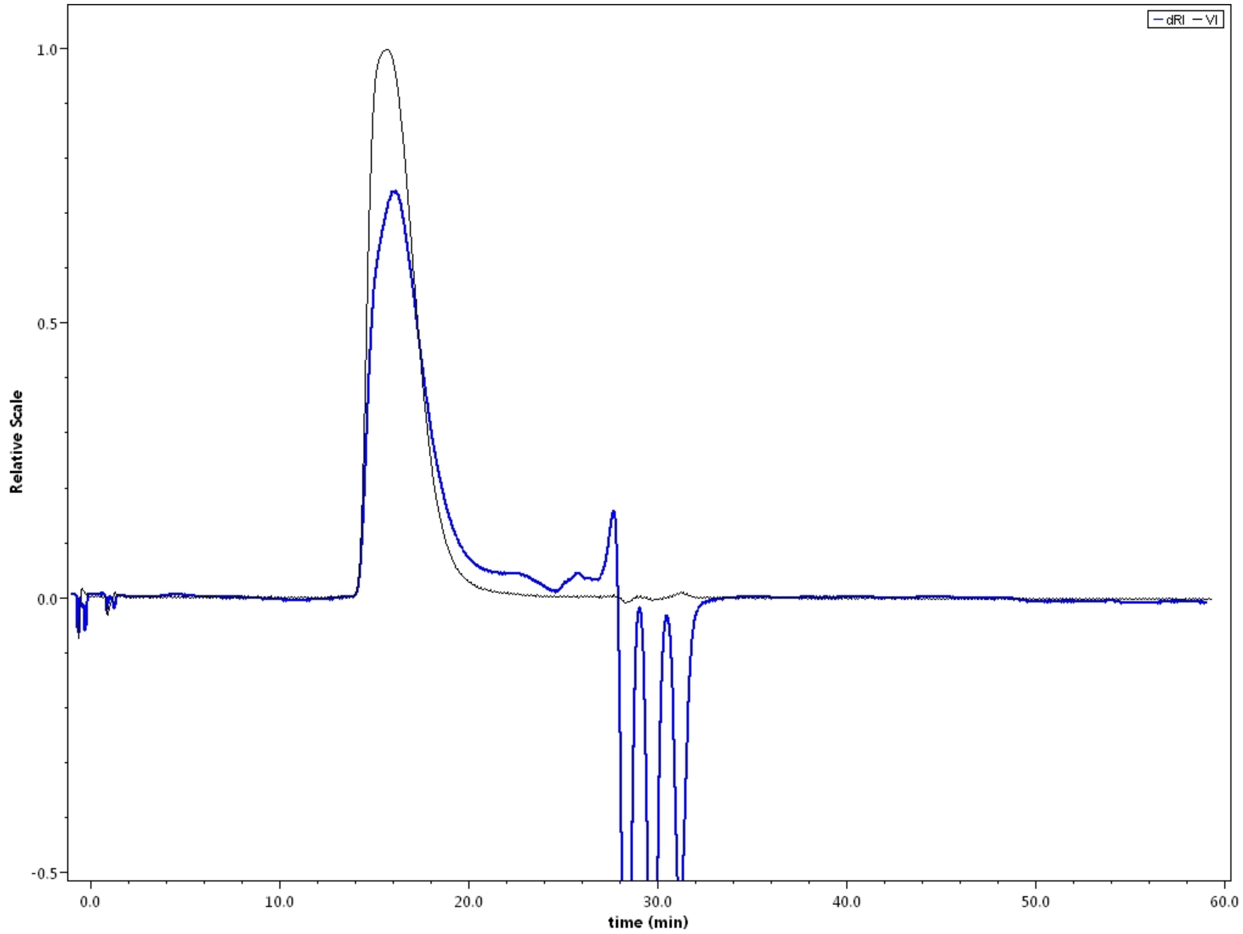


**Figure S94**: Gel permeation chromatogram (refractive index and viscometry traces) for polycaprolactone (Table 2, entry 4), $M_n$ of 177.7 kDa, *Đ* 1.17.

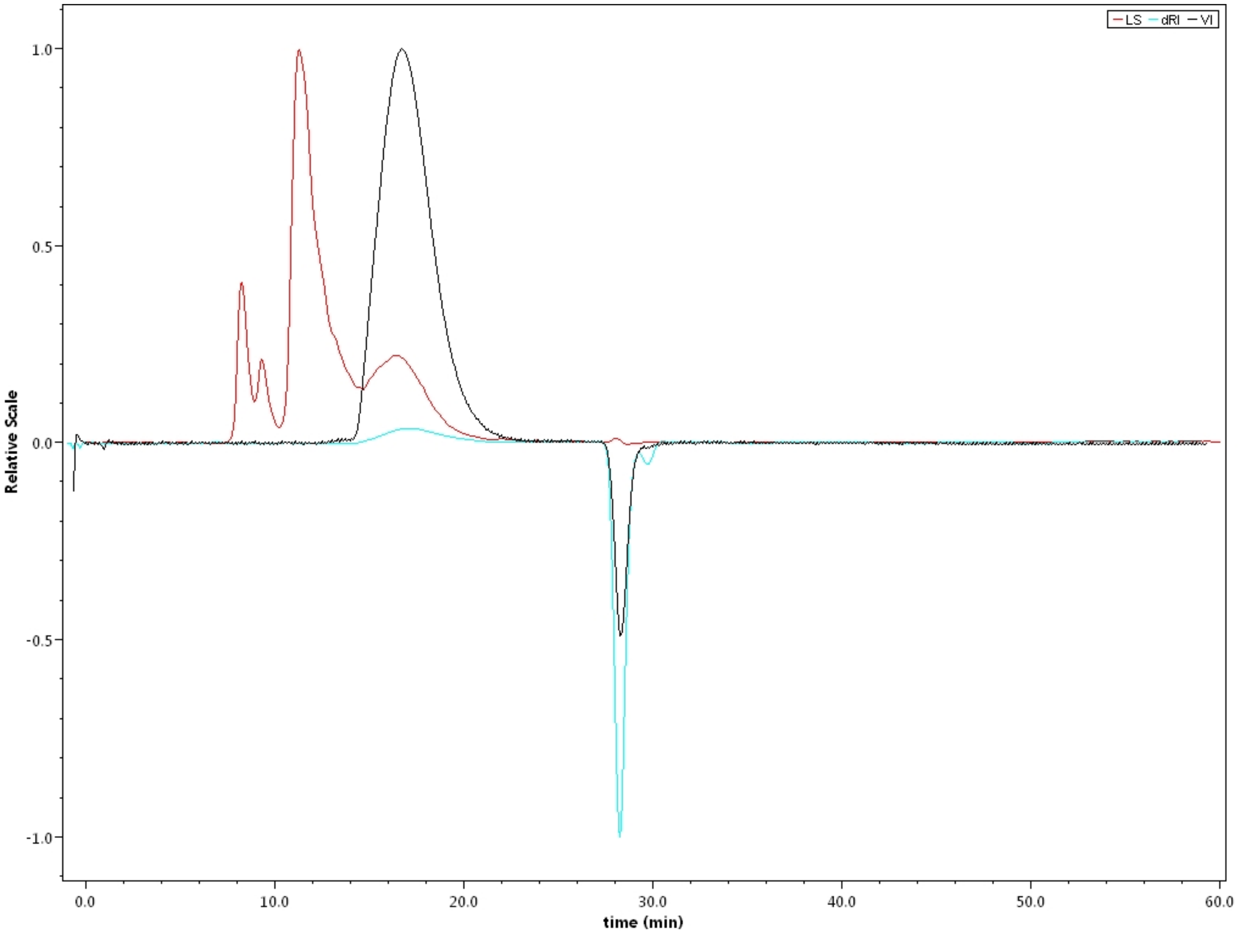


**Figure S95**: Gel permeation chromatogram (light scattering, refractive index, and viscometry traces) for polycaprolactone (Table 2, entry 5), $M_n$ of 99.24 kDa, *Đ* 1.33.

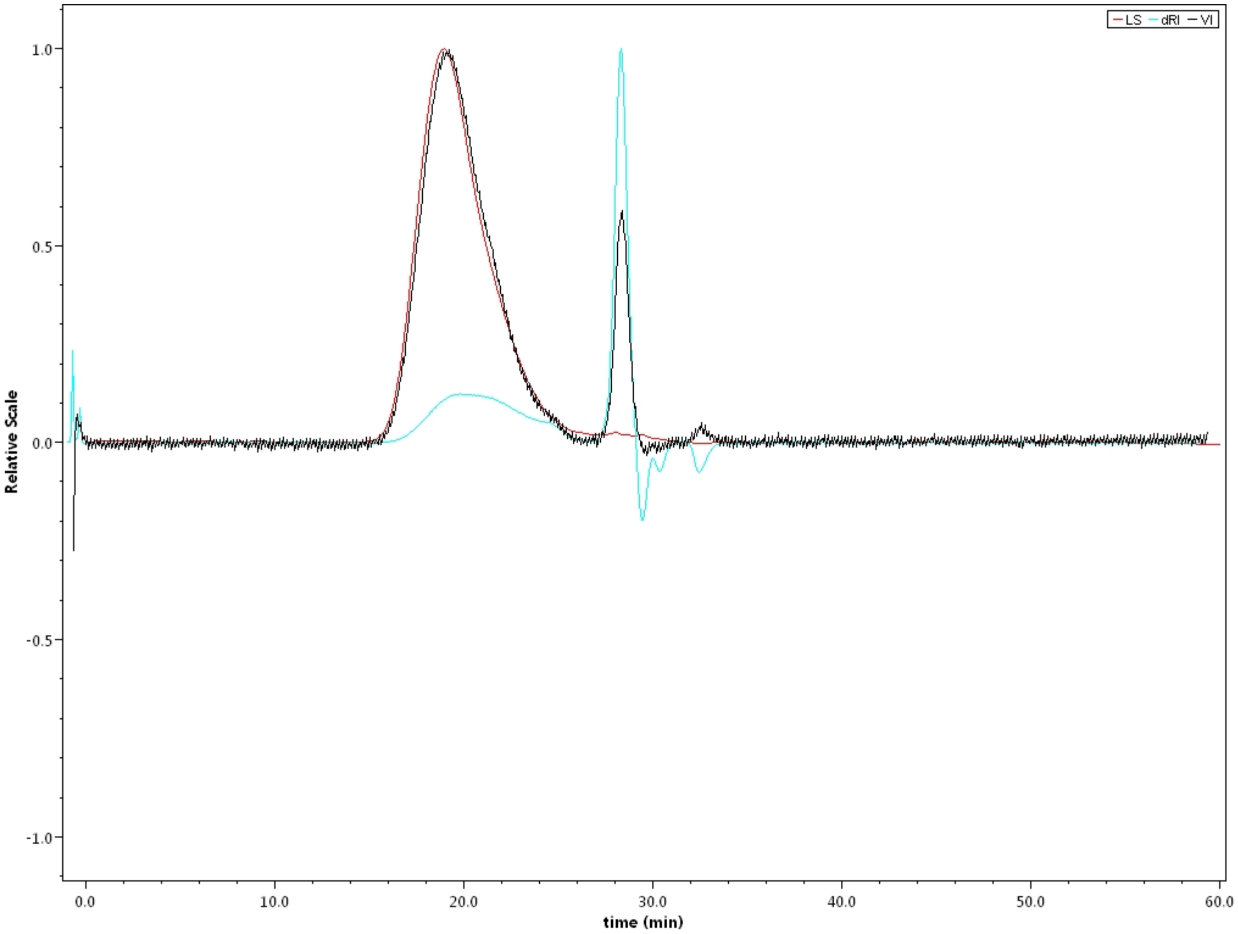


**Figure S96**: Gel permeation chromatogram (light scattering, refractive index, and viscometry traces) for polycaprolactone (Figure 8), $M_n$ of 12.63 kDa, *Đ* 1.48.

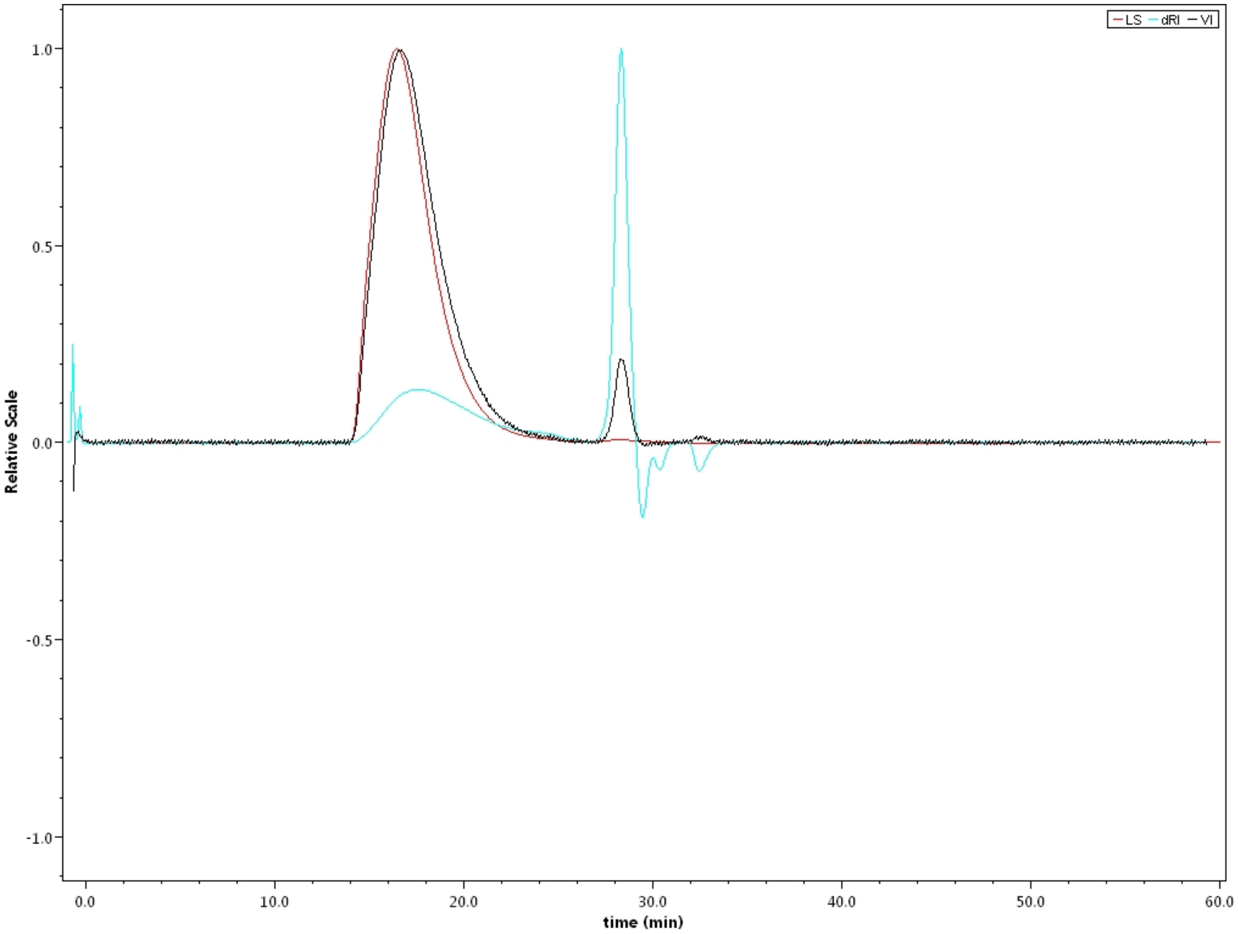


**Figure S97**: Gel permeation chromatogram (light scattering, refractive index, and viscometry traces) for polycaprolactone (Figure 8), $M_n$ of 18.67 kDa, *Đ* 2.45.

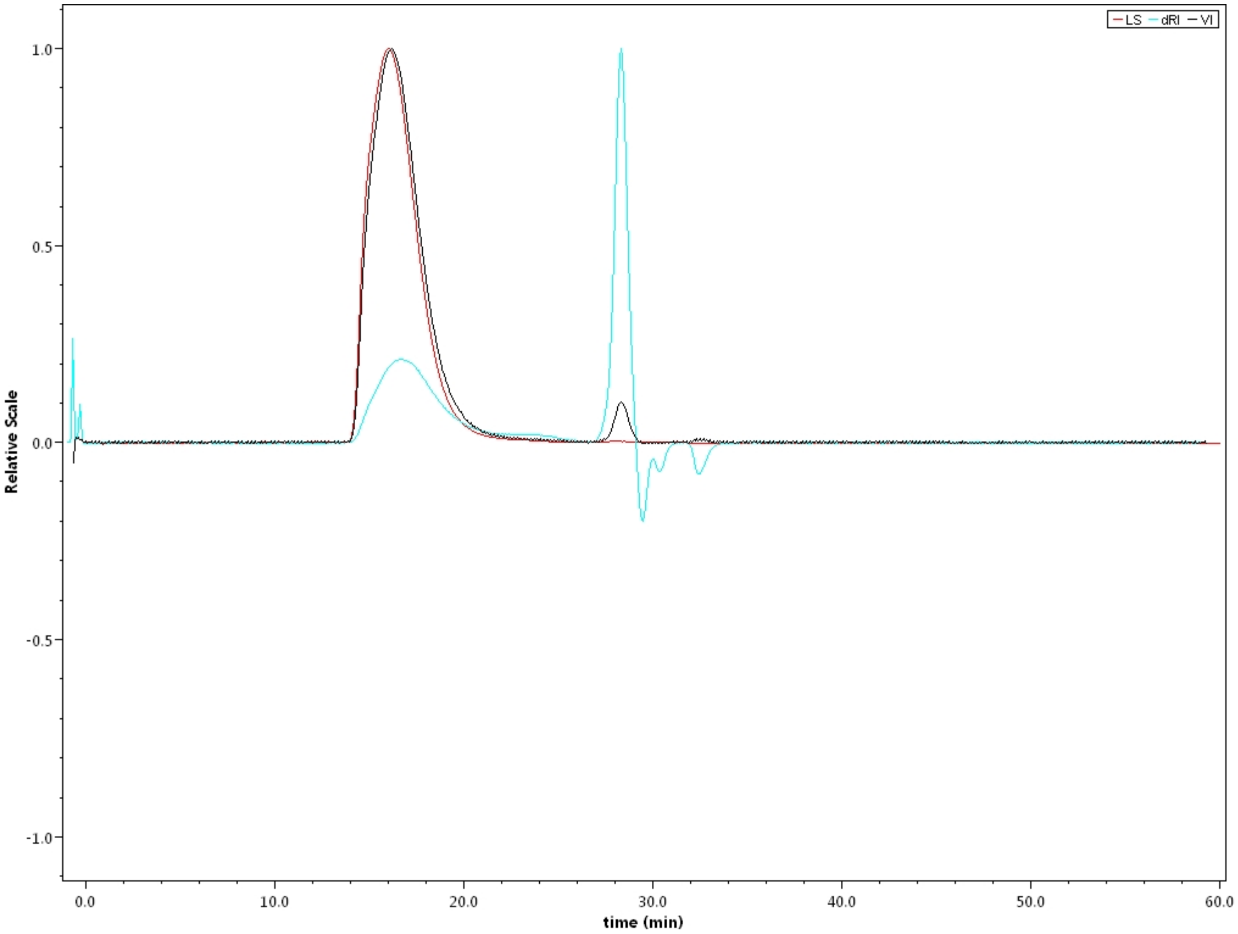


**Figure S98**: Gel permeation chromatogram (light scattering, refractive index, and viscometry traces) for polycaprolactone (Figure 8), $M_n$ of 48.59 kDa, $Đ$ 1.59.

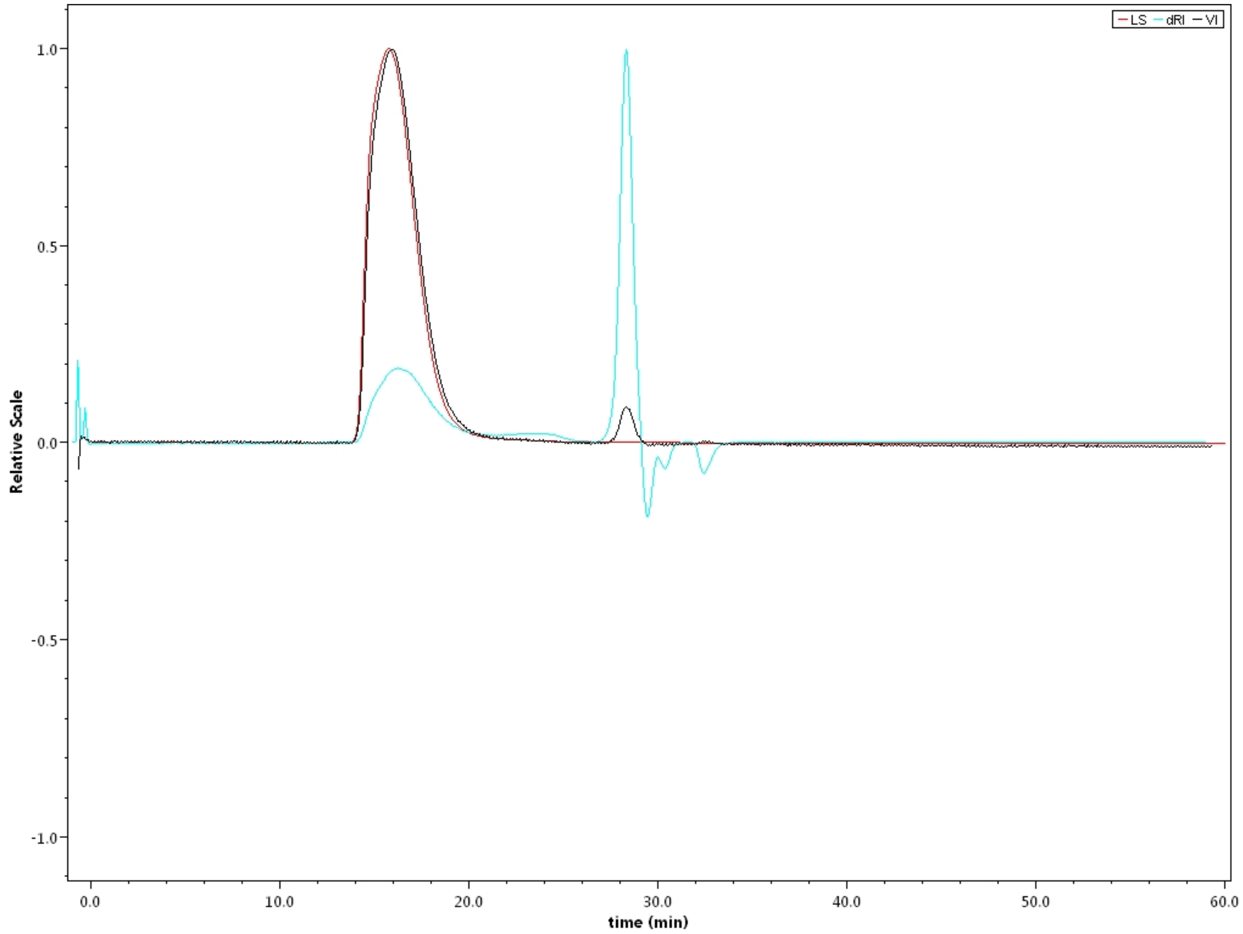


**Figure S99**: Gel permeation chromatogram (light scattering, refractive index, and viscometry traces) for polycaprolactone (Figure 8), $M_n$ of 84.42 kDa, *Đ* 1.44.

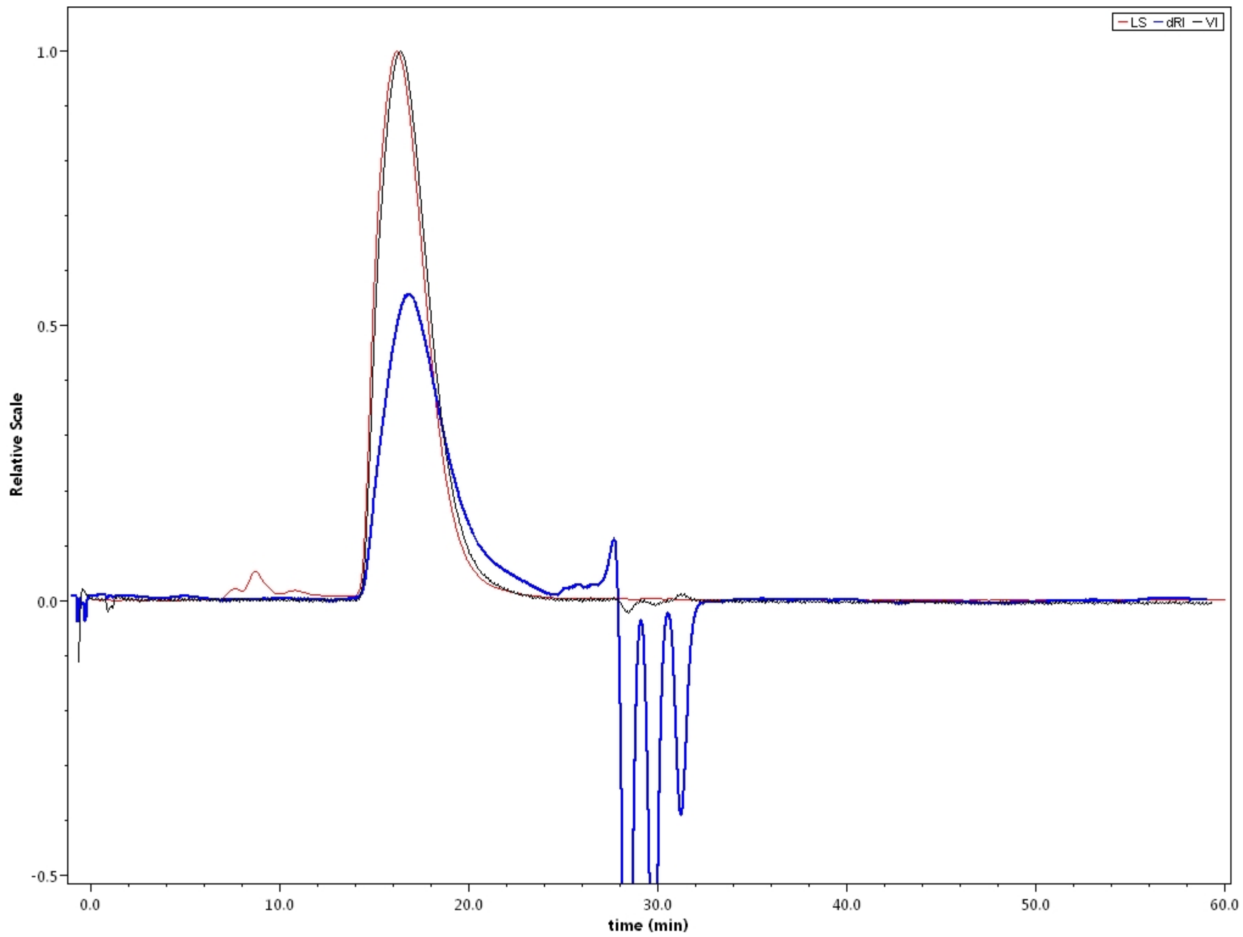


**Figure S100**: Gel permeation chromatogram (light scattering, refractive index and viscometry traces) for polycaprolactone (Table 2, entry 6), $M_n$ 82.31 kDa, *Đ* 1.37.

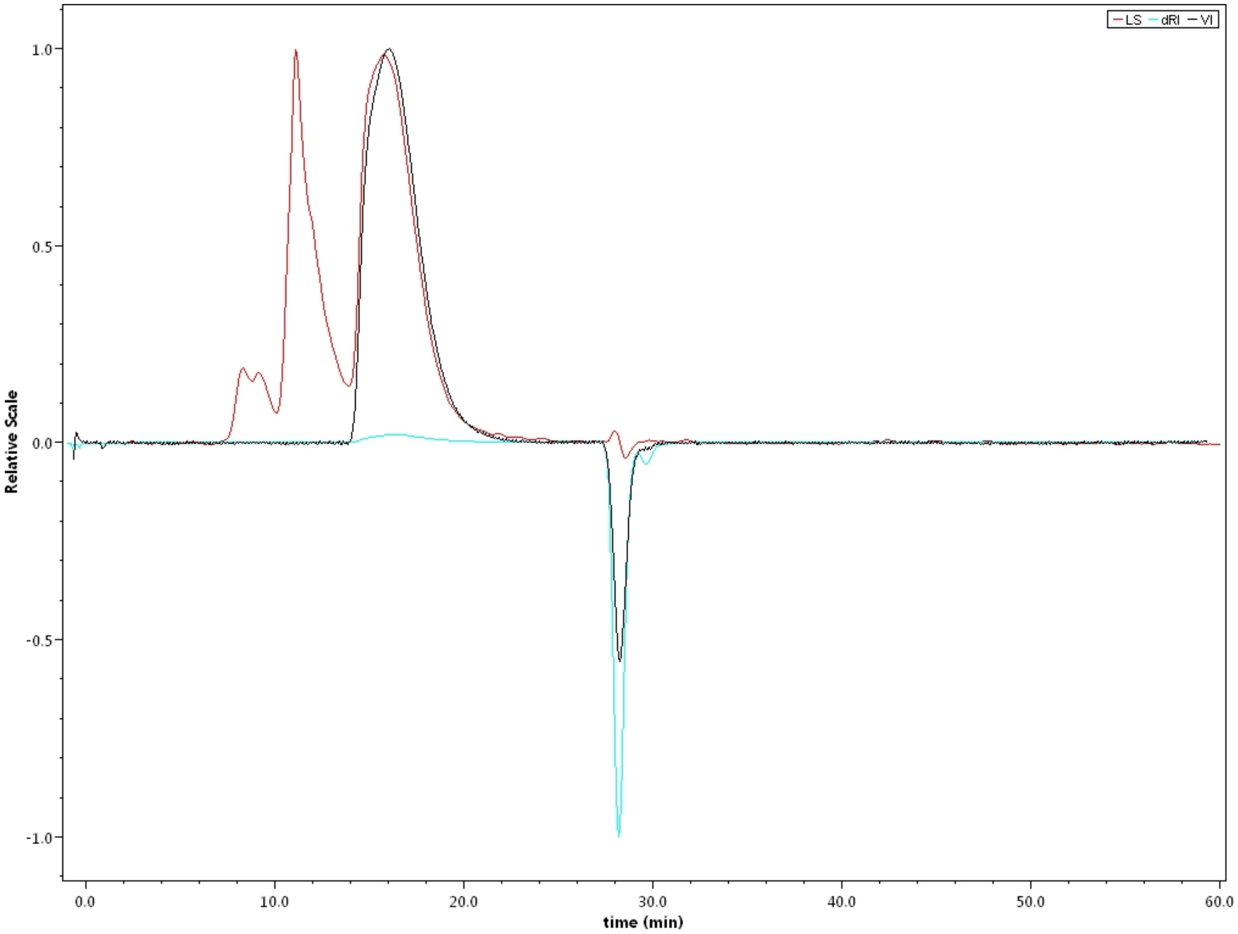


**Figure S101**: Gel permeation chromatogram (light scattering, refractive index and viscometry traces) for polylactide (Table 2, entry 8) $M_n$ of 176.2 kDa, *Đ* 1.31.

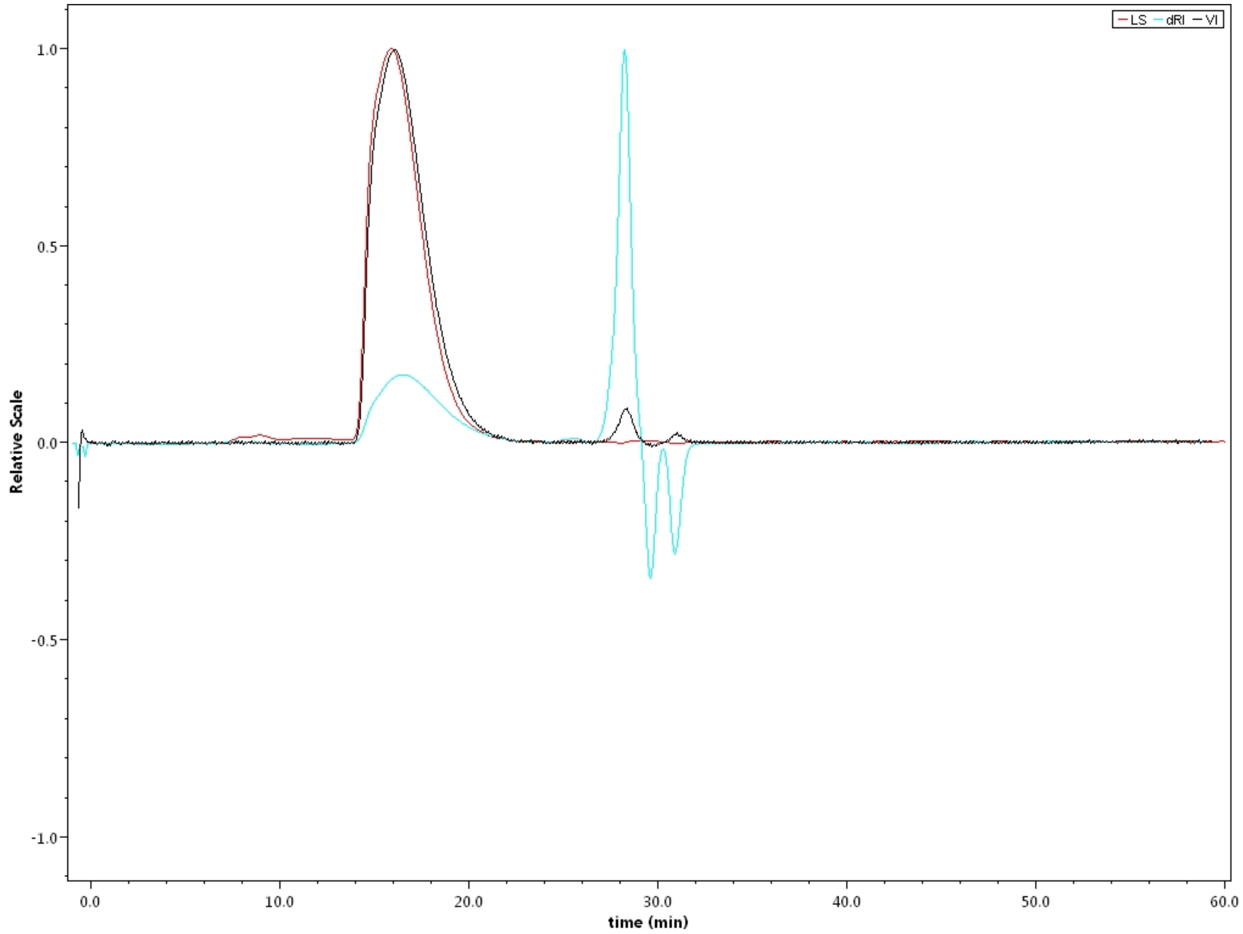


**Figure S102**: Gel permeation chromatogram (light scattering, refractive index and viscometry traces) for polylactide (Table 2, entry 9) $M_n$ of 105.9 kDa, *Đ* 1.44.

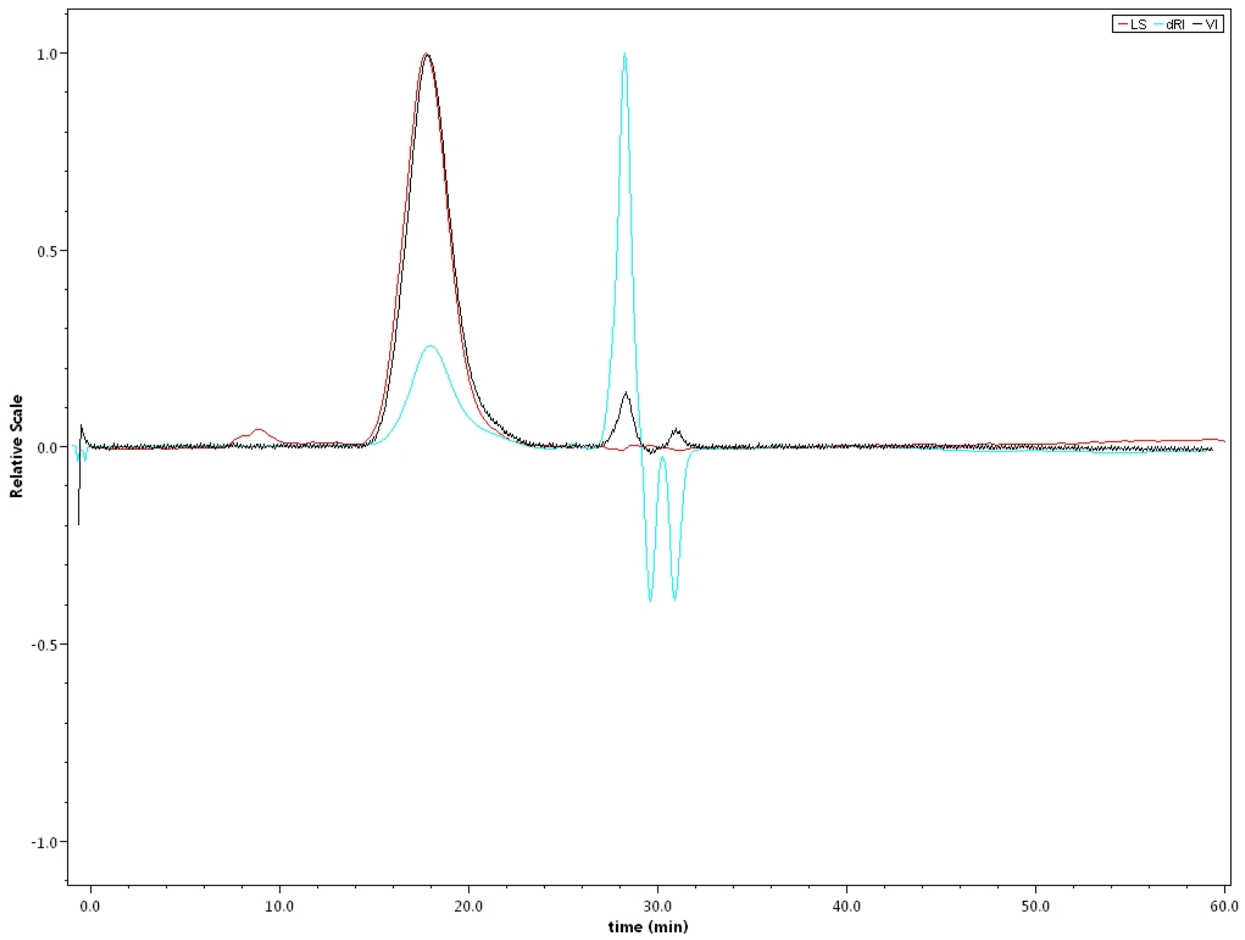


**Figure S103**: Gel permeation chromatogram (light scattering, refractive index and viscometry traces) for polylactide (Table 2, entry 8) $M_n$ of 55.43 kDa, *Đ* 1.15.

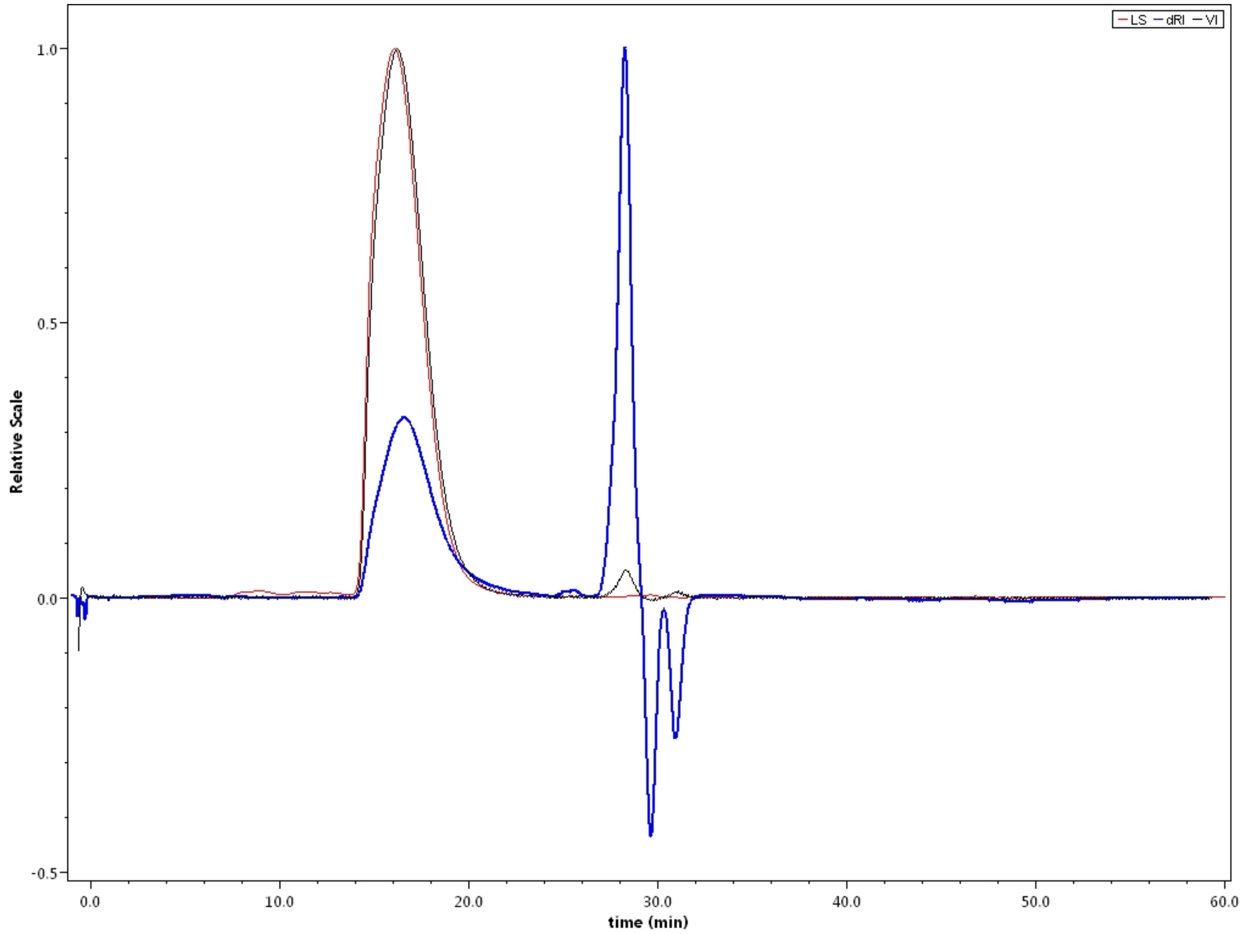


**Figure S104**: Gel permeation chromatogram (light scattering, refractive index and viscometry traces) for polylactide (Table 2, entry 11) $M_n$ of 118.2 kDa, *Đ* 1.31.

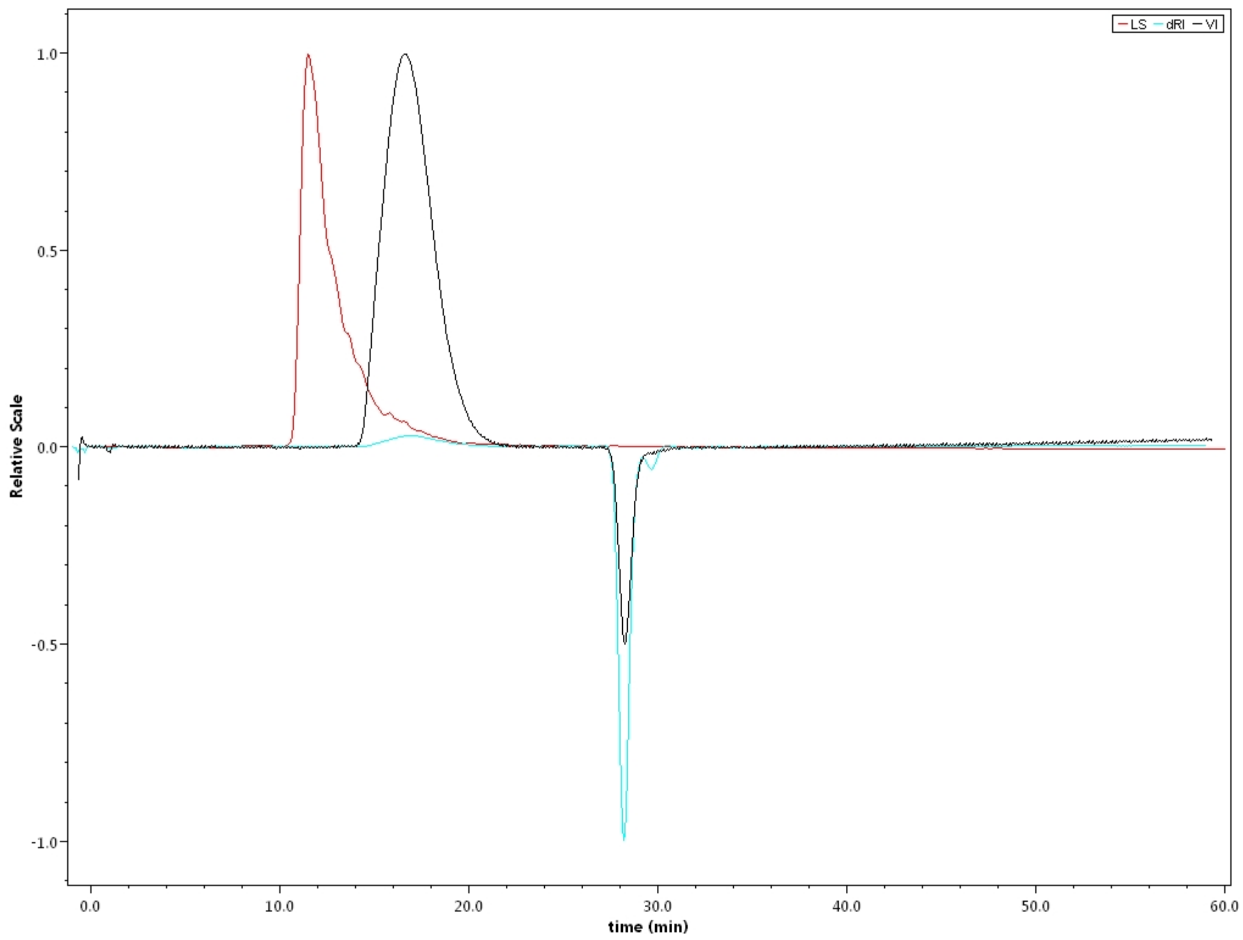


**Figure S105**: Gel permeation chromatogram (light scattering, refractive index and viscometry traces) for polylactide (Table 2, entry 12; Figure 8) $M_n$ of 228.9 kDa, *Đ* 2.2.

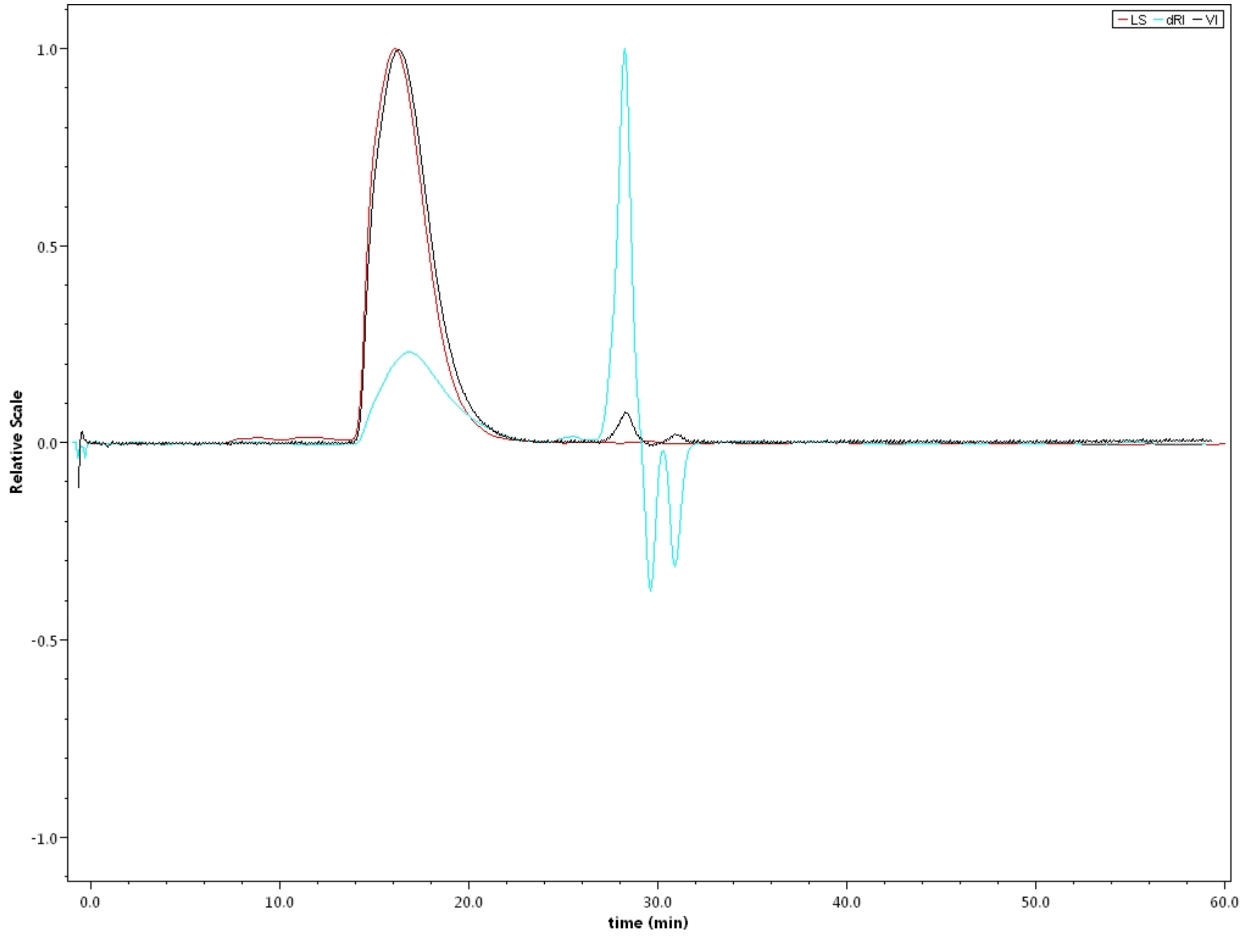


**Figure S106**: Gel permeation chromatogram (light scattering, refractive index and viscometry traces) for polylactide (Table 2, entry 13) $M_n$ of 84.91 kDa, *Đ* 1.54.

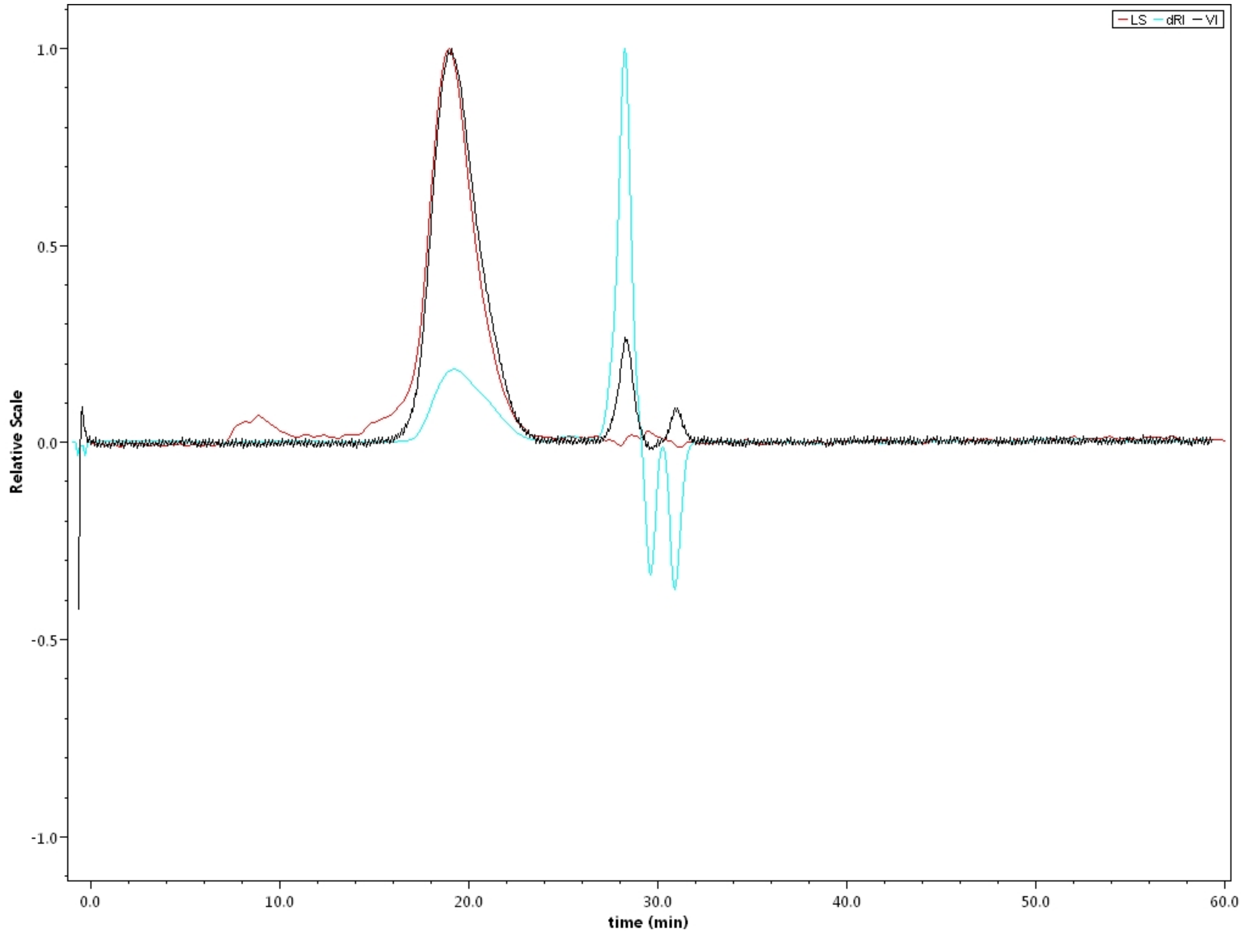


**Figure S107**: Gel permeation chromatogram (light scattering, refractive index and viscometry traces) for polylactide (Table 2, entry 14) $M_n$ of 31.33 kDa, *Đ* 1.14.

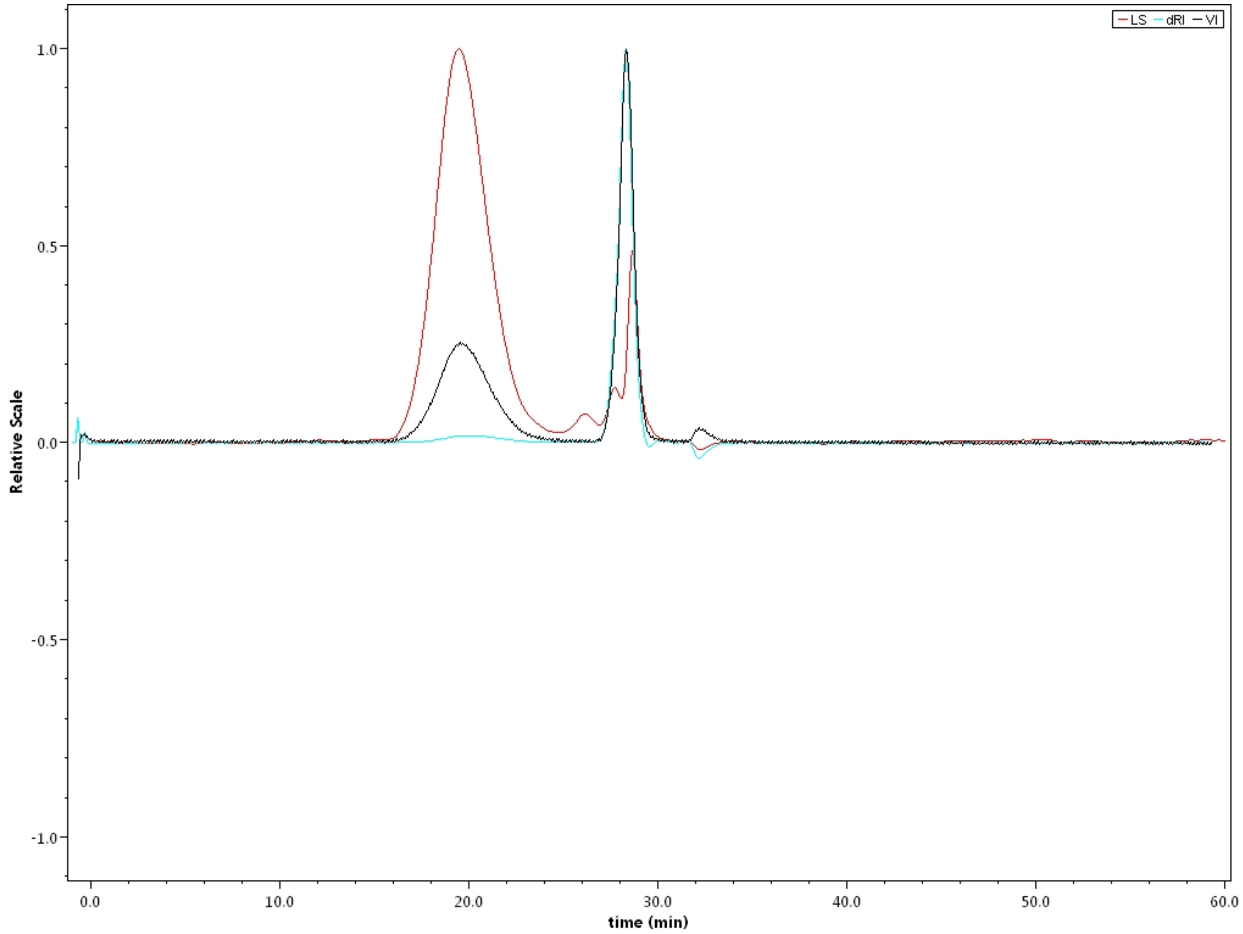


**Figure S108**: Gel permeation chromatogram (light scattering, refractive index and viscometry traces) for polylactide (Figure 8) $M_n$ of 21.11 kDa, *Đ* 1.16.

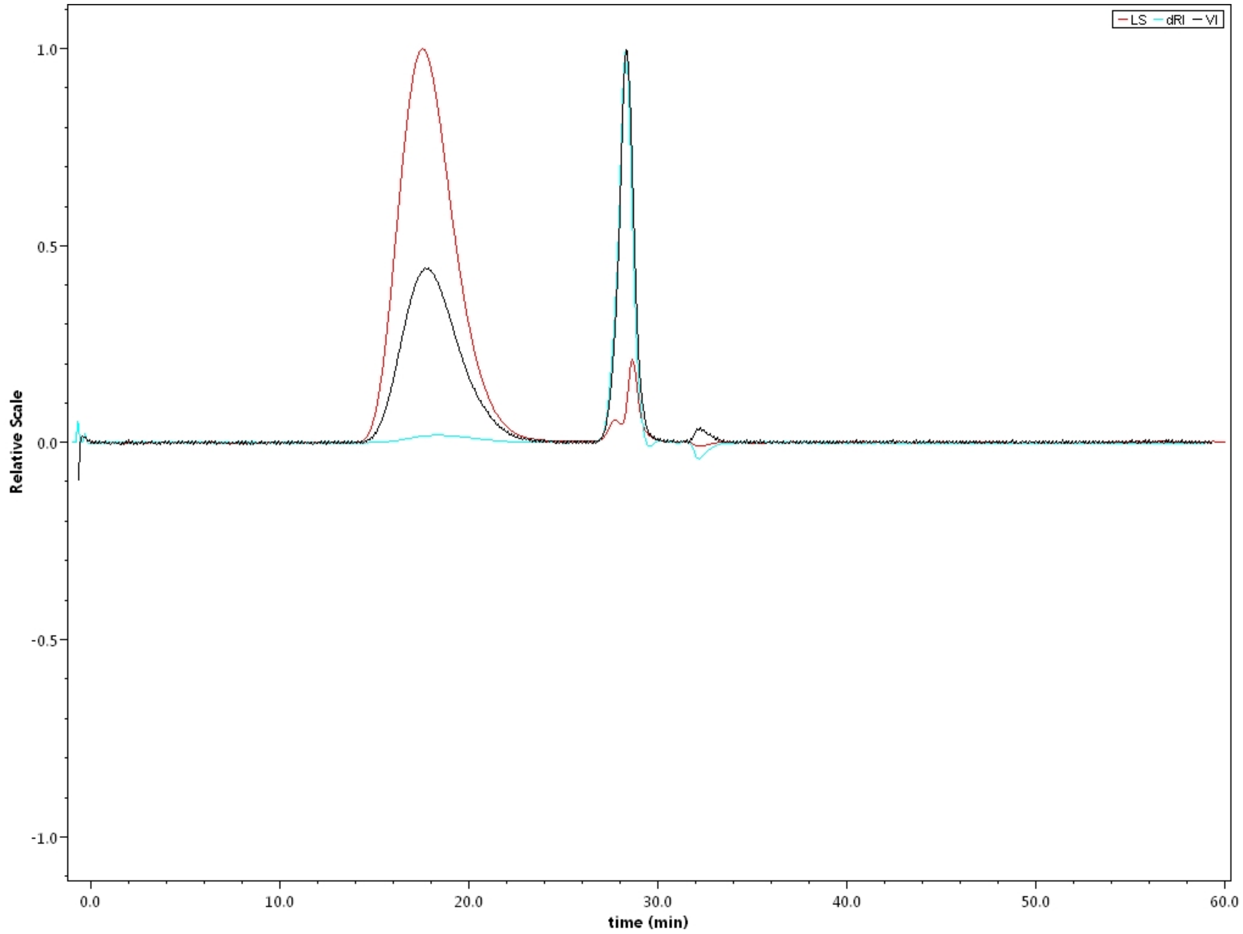


**Figure S109**: Gel permeation chromatogram (light scattering, refractive index and viscometry traces) for polylactide (Figure 8) $M_n$ of 29.51 kDa, *Đ* 1.37.

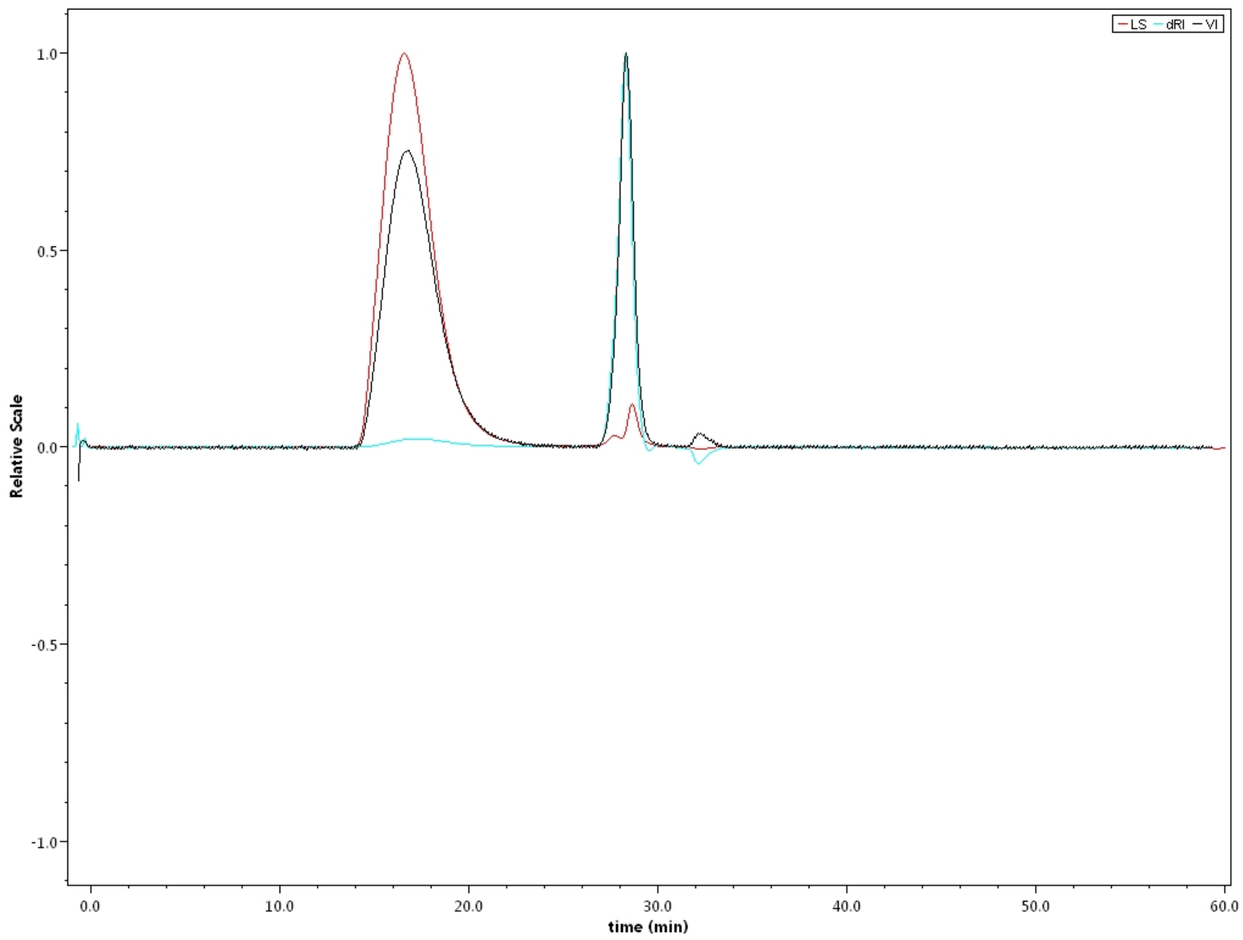


**Figure S110**: Gel permeation chromatogram (light scattering, refractive index and viscometry traces) for polylactide (Figure 8) $M_n$ of 41.87 kDa, *Đ* 1.50.

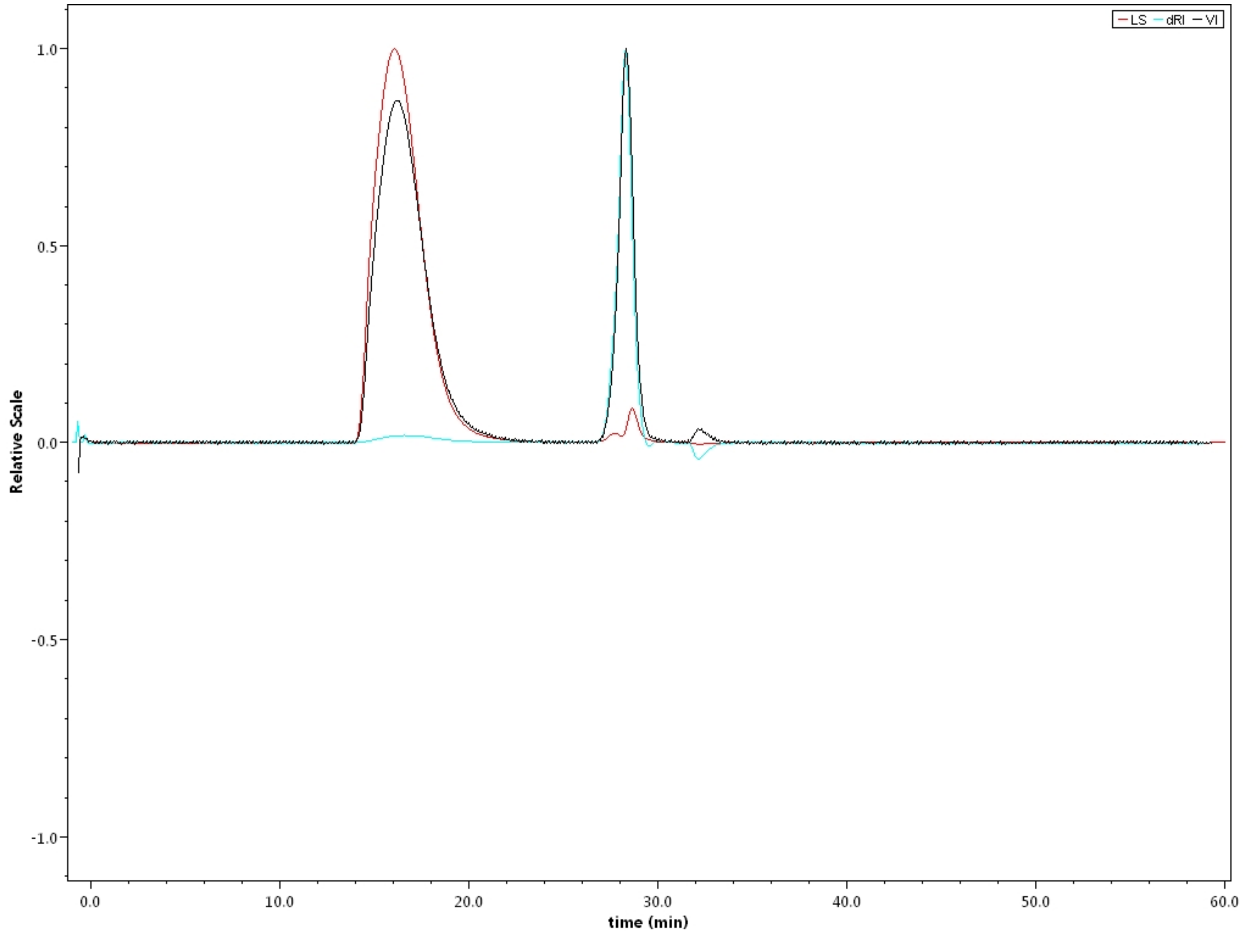


**Figure S111**: Gel permeation chromatogram (light scattering, refractive index and viscometry traces) for polylactide (Figure 8) $M_n$ of 72.85 kDa, *Đ* 1.51.

## MALDI-TOF Mass Spectrometry

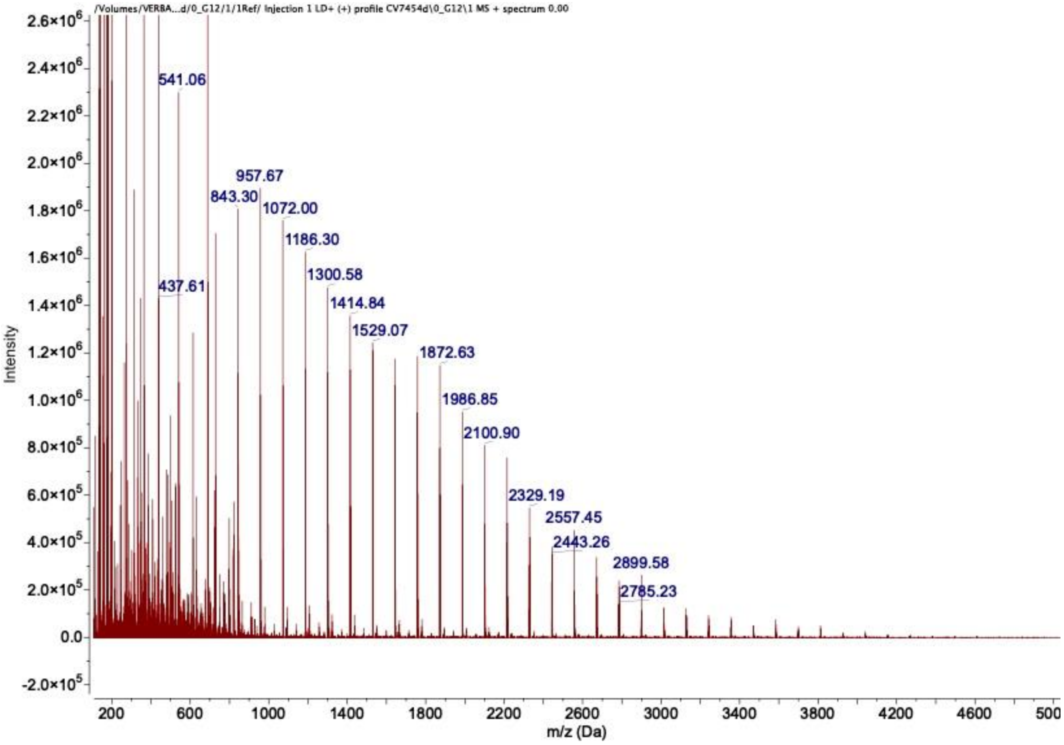


**Figure S112**: MALDI-TOF mass spectrum of polycaprolactone oligomer (Table 2, entry 9).

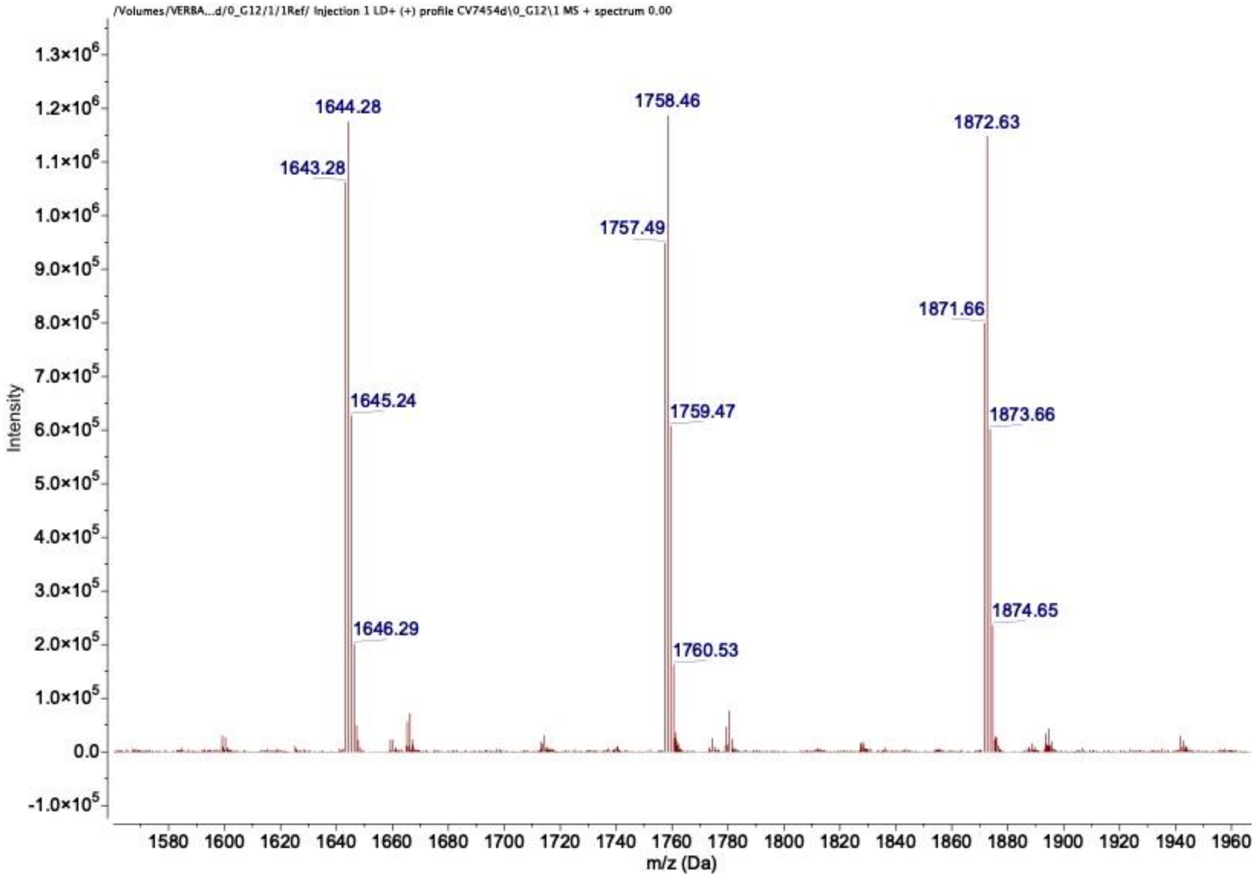


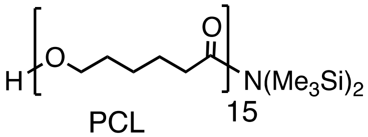


$$\frac{1872.63\ m/z}{114.14\ m/z\,(\text{PCL})} = 16.406, \text{if n} = 16 \text{ then end groups} = 0.406(114.14\,m/z) = 46.3\ m/z$$

$$\text{If n} = 15, \text{then end groups} = 1.406(114.14\ m/z) = \ 160.5\ m/z$$

$$160.5\ m/z\ \approx 160.4\ m/z\ (\text{N}(\text{Me}_3\text{Si})_2 + 1.01\ m/z\ (\text{H}^+)$$

**Figure S113**: Expansion of MALDI-TOF mass spectrum of polycaprolactone oligomer in the region of *m/z* 1560 to 1960. Masses correspond to polycaprolactone with degrees of polymerization of 13, 14 and 15 and having $(Me_3Si)_2N$ and H end groups. Shown below is structure of polycaprolactone oligomer based on MALDI-TOF mass spectrum and calculation of m/z of potential end groups.

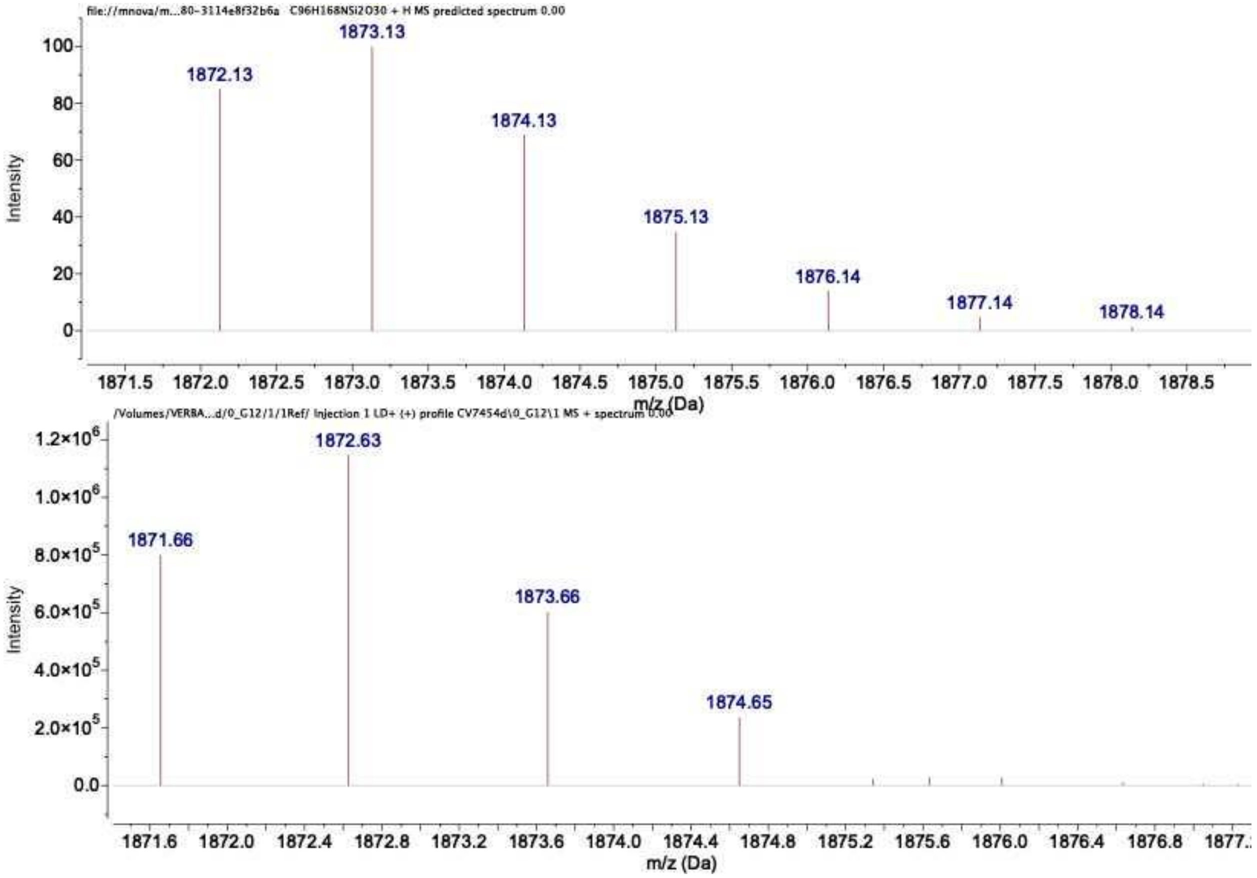


**Figure S114**: Further expansion of MALDI-TOF mass spectrum of polycaprolactone oligomer of *m/z* 1872.63 corresponding to a degree of polymerization of 15 caprolactone units (bottom) and predicted isotope pattern for polycaprolactone having $(Me_3Si)_2N$ and H end groups, $(Me_3Si)_2N$-$(C_6H_{10}O_2)_{15}$-H (top).

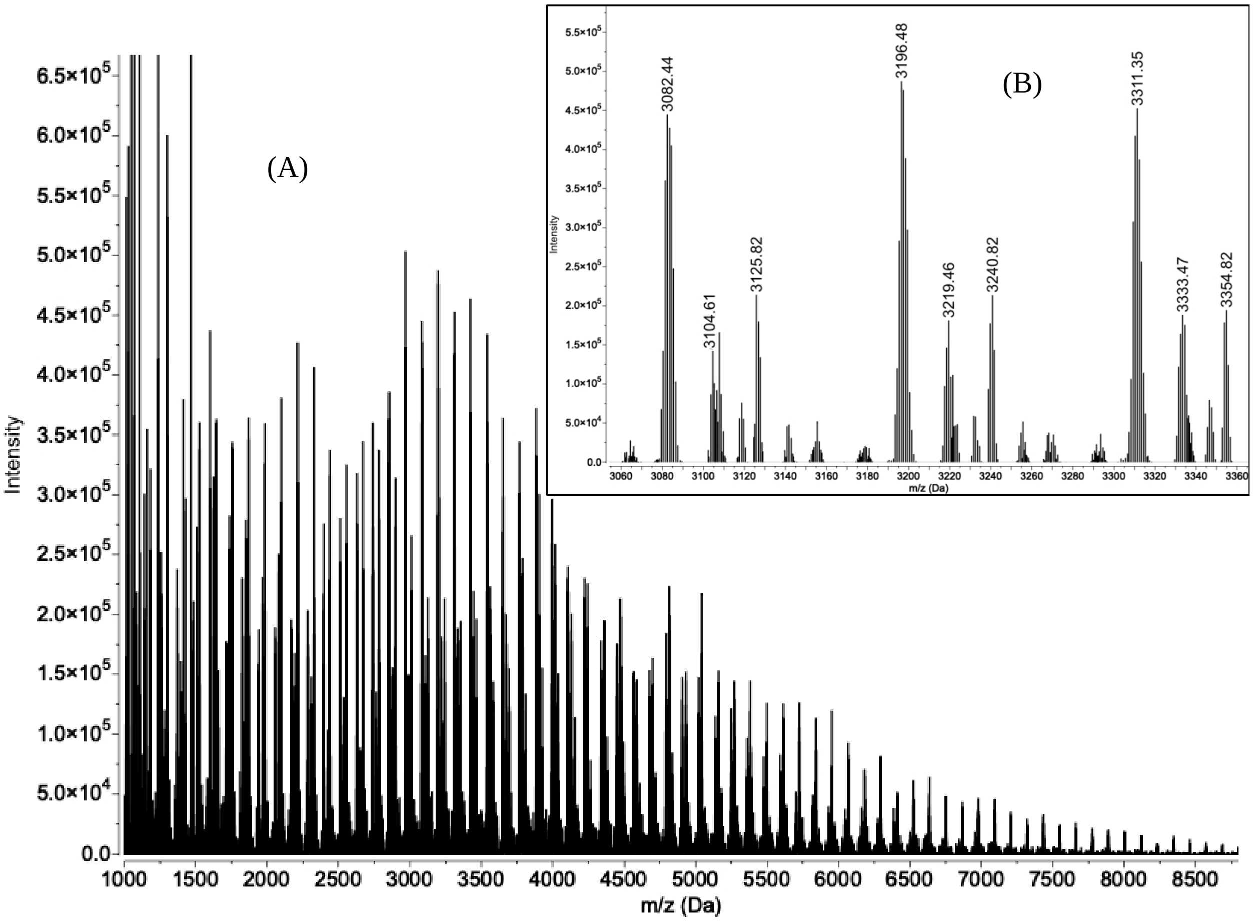


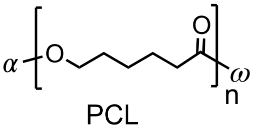


C)
PCL

$$\frac{3196.48\,m/z}{114.14\,m/z\ \text{(PCL)}} = 28.004, \qquad \text{if n} = 28 \text{ then } \alpha + \omega = 0.004(114.14\ \text{m}/z) = 0.5\,m/z$$

$$\text{if n} = 27 \text{ then } \alpha + \omega = 1.004(114.14\,m/z) = \ 114.6\ m/z$$

$$\text{if n} = 26 \text{ then } \alpha + \omega = 2.004(114.14\,m/z) = \ 228.8\ m/z$$

$$\text{if n} = 25 \text{ then } \alpha + \omega = 3.004(114.14\,m/z) = \ 342.9\ m/z$$

$$\text{if n} = 24 \text{ then } \alpha + \omega = 4.004(114.14\,m/z) = \ 457.0\ m/z$$

$$\text{if n} = 23 \text{ then } \alpha + \omega = 5.004(114.14\,m/z) = \ 571.2\ m/z$$

**Figure S115:** (A) MALDI-TOF mass spectrum produced by **3b** ε-caprolactone polymerization. (B) Expanded mass region (m/z 3060 – 3360, degree of polymerization 27, 28, 29) of the spectrum showing multiple mass distributions for the produced PCL. C) Structure of polycaprolactone oligomer based on MALDI-TOF mass spectrum with general end groups (α and ω) and calculation of m/z of potential end groups.

## Alternate X-Ray Crystal Structure Images

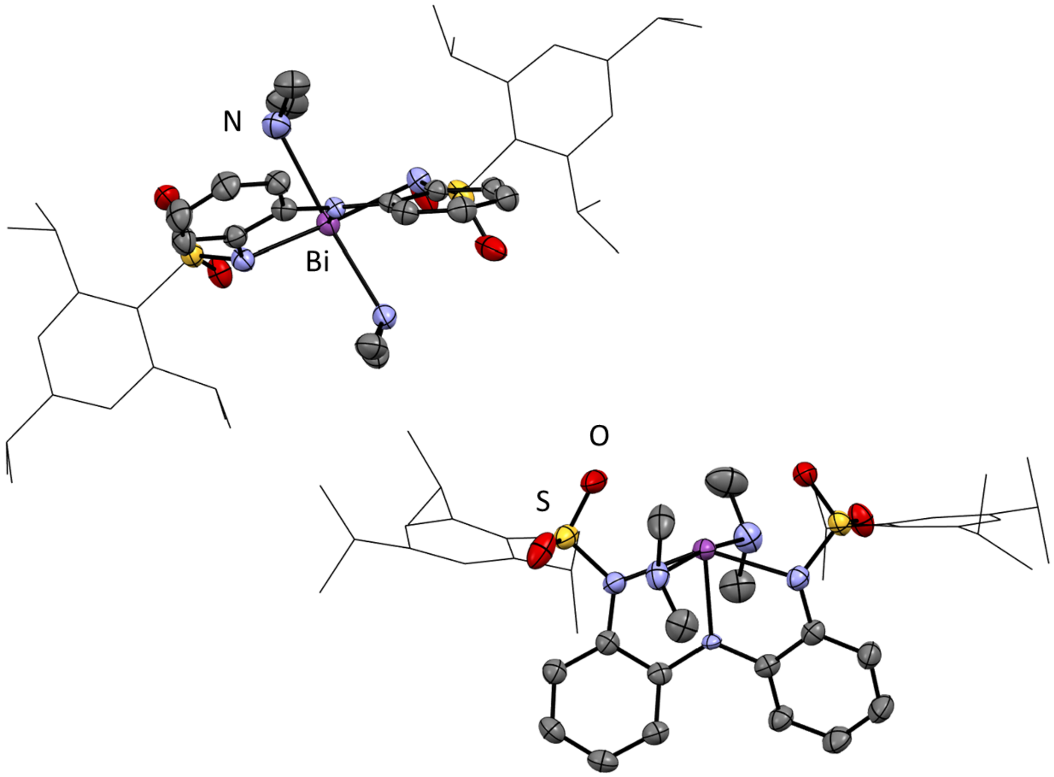


**Figure S116.** Alternate views of the molecular structure of **2d**. Ellipsoids have been drawn at the 50% probability level. Hydrogen atoms and solvent molecules have been removed for clarity. The Trip group has been shown in wireframe mode for clarity. Purple = bismuth, blue = nitrogen, red = oxygen, light yellow = silicon, yellow = sulfur, brown = bromine

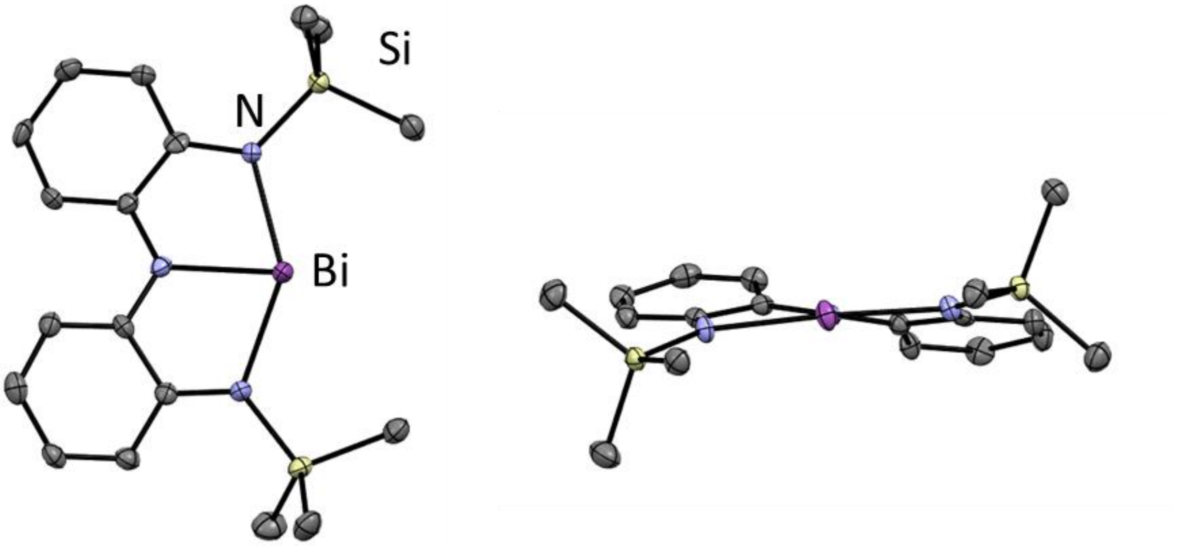


**Figure S117.** Alternate views of the molecular structure of **3a**. Ellipsoids have been drawn at the 50% probability level. Hydrogen atoms and solvent molecules have been removed for clarity. Purple = bismuth, blue = nitrogen, red = oxygen, light yellow = silicon, yellow = sulfur, brown = bromine

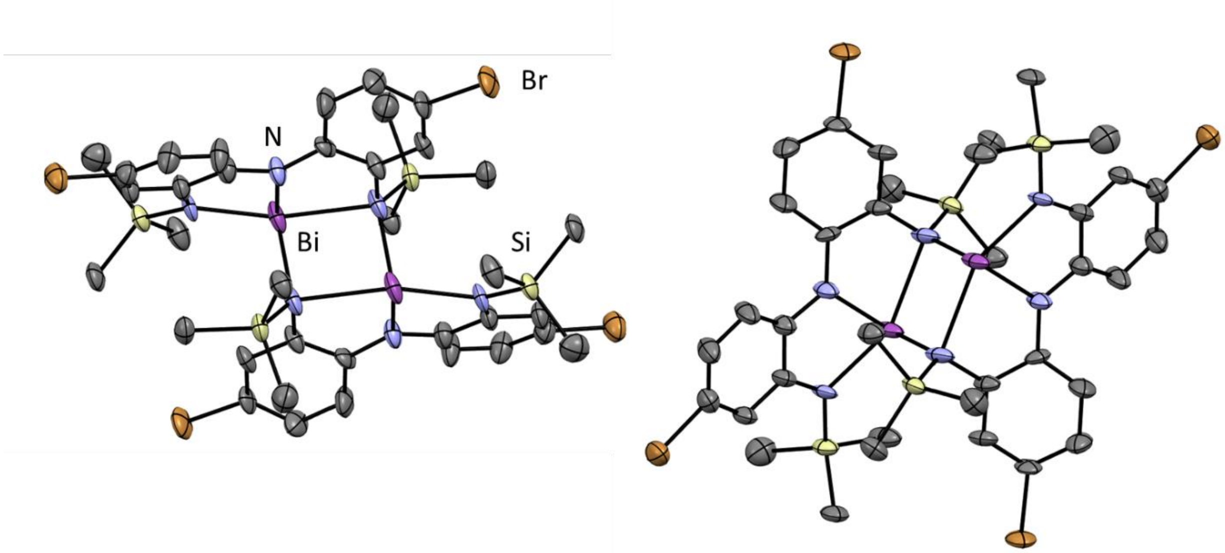


**Figure S118.** Alternate views of the molecular structure of **3b**. Ellipsoids have been drawn at the 50% probability level. Hydrogen atoms and solvent molecules have been removed for clarity. Purple = bismuth, blue = nitrogen, red = oxygen, light yellow = silicon, yellow = sulfur, brown = bromine

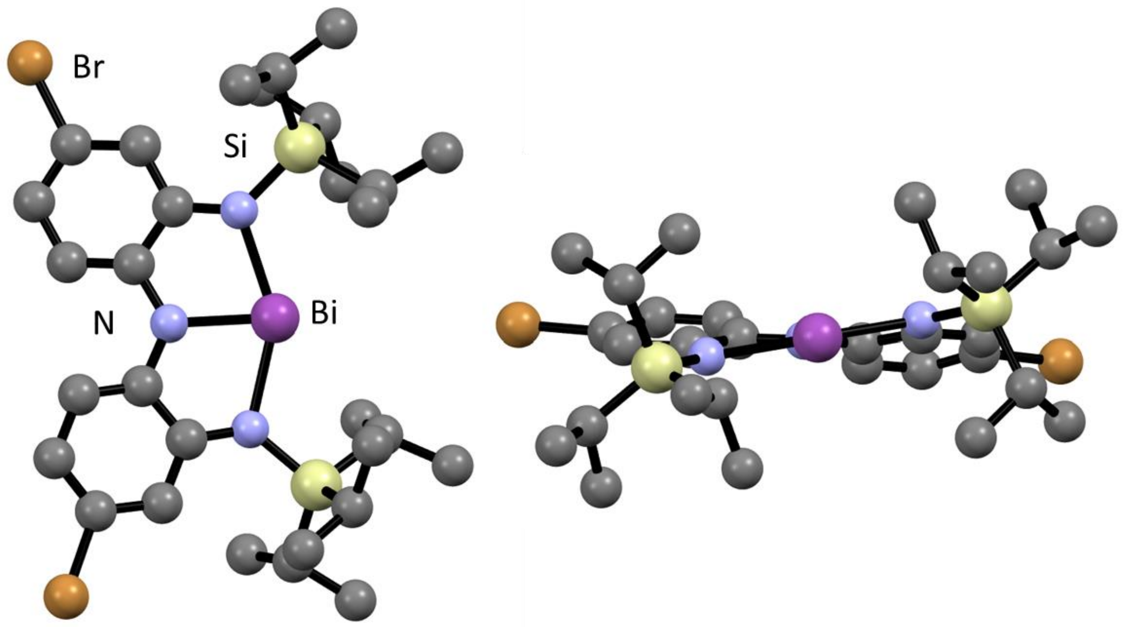


**Figure S119.** Alternate views of the molecular structure of **3c**. Structure shown in the ball and stick model. Hydrogen atoms and solvent molecules have been removed for clarity. Purple = bismuth, blue = nitrogen, red = oxygen, light yellow = silicon, yellow = sulfur, brown = bromine

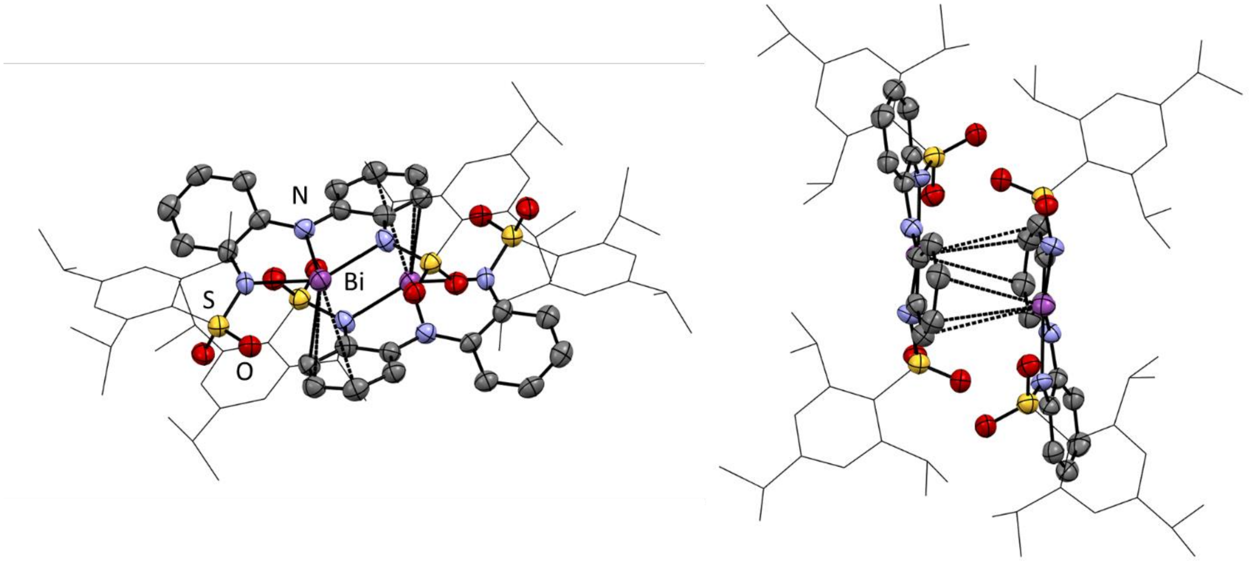


**Figure S120.** Alternate views of the molecular structure of **3d**. Ellipsoids have been drawn at the 50% probability level. Hydrogen atoms and solvent molecules have been removed for clarity. The Trip group has been shown in wireframe mode for clarity. Purple = bismuth, blue = nitrogen, red = oxygen, light yellow = silicon, yellow = sulfur, brown = bromine

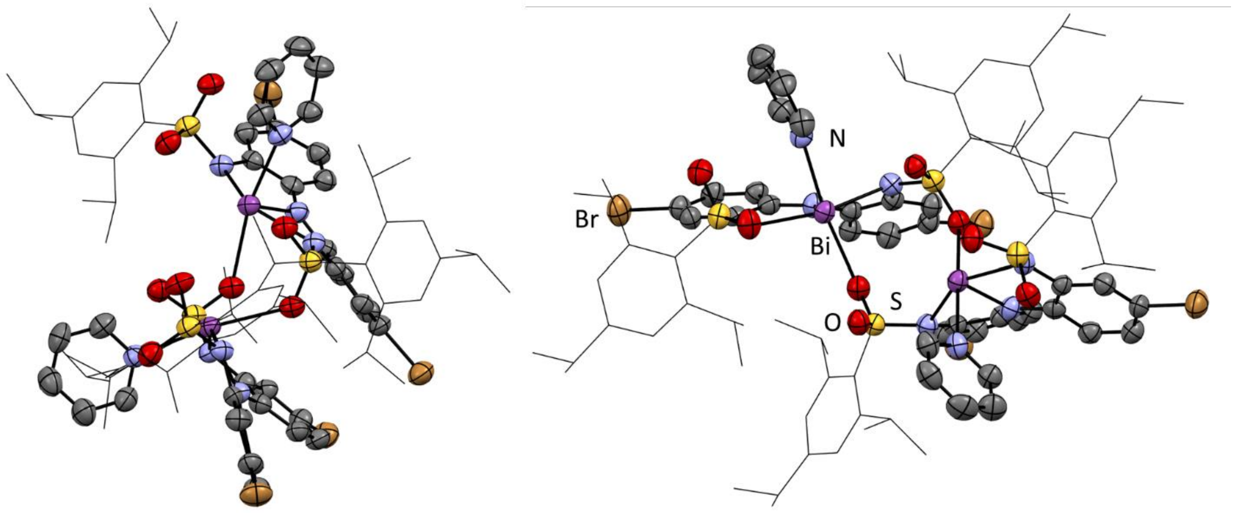


**Figure S121.** Alternate views of the molecular structure of **3e-py**. Ellipsoids have been drawn at the 50% probability level. Hydrogen atoms and solvent molecules have been removed for clarity. The Trip group has been shown in wireframe mode for clarity. Purple = bismuth, blue = nitrogen, red = oxygen, light yellow = silicon, yellow = sulfur, brown = bromine

## References:


(1) Lin, Y.-C.; Hatzakis, E.; McCarthy, S. M.; Reichl, K. D.; Lai, T.-Y.; Yennawar, H. P.; Radosevich, A. T. P–N Cooperative Borane Activation and Catalytic Hydroboration by a Distorted Phosphorous Triamide Platform. *Journal of the American Chemical Society* **2017**, *139* (16), 6008-6016. DOI: 10.1021/jacs.7b02512.

(2) Munhá, R. F.; Zarkesh, R. A.; Heyduk, A. F. Tuning the Electronic and Steric Parameters of a Redox-Active Tris(amido) Ligand. *Inorganic Chemistry* **2013**, *52* (19), 11244-11255. DOI: 10.1021/ic401496w.

(3) Cook, S. A.; Bogart, J. A.; Levi, N.; Weitz, A. C.; Moore, C.; Rheingold, A. L.; Ziller, J. W.; Hendrich, M. P.; Borovik, A. S. Mononuclear complexes of a tridentate redox-active ligand with sulfonamido groups: structure, properties, and reactivity. *Chemical Science* **2018**, *9* (31), 6540-6547, 10.1039/C7SC05445A. DOI: 10.1039/C7SC05445A.

(4) Schrock, R. R.; Lee, J.; Liang, L.-C.; Davis, W. M. The synthesis and structures of tantalum complexes that contain a triamido or a diamidoamine ligand. *Inorganica Chimica Acta* **1998**, *270* (1), 353-362. DOI: https://doi.org/10.1016/S0020-1693(97)05869-6.

(5) Kindervater, M. B.; Marczenko, K. M.; Werner-Zwanziger, U.; Chitnis, S. S. A Redox-Confused Bismuth(I/III) Triamide with a T-Shaped Planar Ground State. *Angewandte Chemie International Edition* **2019**, *58* (23), 7850-7855. DOI: https://doi.org/10.1002/anie.201903354.

(6) Clegg, W.; Compton, N. A.; Errington, R. J.; Fisher, G. A.; Green, M. E.; Hockless, D. C. R.; Norman, N. C. X-ray crystal structure of bismuth dimethylamide. *Inorganic Chemistry* **1991**, *30* (24), 4680-4682. DOI: 10.1021/ic00024a046.

(7) Vehkamäki, M.; Hatanpää, T.; Ritala, M.; Leskelä, M. Bismuth precursors for atomic layer deposition of bismuth-containing oxide films. *Journal of Materials Chemistry* **2004**, *14* (21), 3191-3197, 10.1039/B405891G. DOI: 10.1039/B405891G.

(8) Rodriguez, M. M.; Stubbert, B. D.; Scarborough, C. C.; Brennessel, W. W.; Bill, E.; Holland, P. L. Isolation and Characterization of Stable Iron(I) Sulfide Complexes. *Angewandte Chemie International Edition* **2012**, *51* (33), 8247-8250. DOI: https://doi.org/10.1002/anie.201202211.

(9) Erdmann, P.; Leitner, J.; Schwarz, J.; Greb, L. An Extensive Set of Accurate Fluoride Ion Affinities for p-Block Element Lewis Acids and Basic Design Principles for Strong Fluoride Ion Acceptors. *ChemPhysChem* **2020**, *21* (10), 987-994. DOI: https://doi.org/10.1002/cphc.202000244.
